# Hydrogen and Carbon Monoxide-Utilizing *Kyrpidia spormannii* Species From Pantelleria Island, Italy

**DOI:** 10.3389/fmicb.2020.00951

**Published:** 2020-05-19

**Authors:** Carmen Hogendoorn, Arjan Pol, Nunzia Picone, Geert Cremers, Theo A. van Alen, Antonina L. Gagliano, Mike S. M. Jetten, Walter D’Alessandro, Paola Quatrini, Huub J. M. Op den Camp

**Affiliations:** ^1^Department of Microbiology, Institute for Water and Wetland Research, Radboud University, Nijmegen, Netherlands; ^2^Istituto Nazionale di Geofisica e Vulcanologia, Palermo, Italy; ^3^Department of Biological, Chemical and Pharmaceutical Sciences and Technologies (STEBICEF), University of Palermo, Palermo, Italy

**Keywords:** *Kyrpidia spormannii*, H_2_, [NiFe]-hydrogenases, CO, thermoacidophilic, phylogeny

## Abstract

Volcanic and geothermal areas are hot and often acidic environments that emit geothermal gasses, including H_2_, CO and CO_2_. Geothermal gasses mix with air, creating conditions where thermoacidophilic aerobic H_2_- and CO-oxidizing microorganisms could thrive. Here, we describe the isolation of two *Kyrpidia spormannii* strains, which can grow autotrophically by oxidizing H_2_ and CO with oxygen. These strains, FAVT5 and COOX1, were isolated from the geothermal soils of the Favara Grande on Pantelleria Island, Italy. Extended physiology studies were performed with *K. spormannii* FAVT5, and showed that this strain grows optimally at 55°C and pH 5.0. The highest growth rate is obtained using H_2_ as energy source (μ_max_ 0.19 ± 0.02 h^–1^, doubling time 3.6 h). *K. spormannii* FAVT5 can additionally grow on a variety of organic substrates, including some alcohols, volatile fatty acids and amino acids. The genome of each strain encodes for two O_2_-tolerant hydrogenases belonging to [NiFe] group 2a hydrogenases and transcriptome studies using *K. spormannii* FAVT5 showed that both hydrogenases are expressed under H_2_ limiting conditions. So far no Firmicutes except *K. spormannii* FAVT5 have been reported to exhibit a high affinity for H_2_, with a K_s_ of 327 ± 24 nM. The genomes of each strain encode for one putative CO dehydrogenase, belonging to Form II aerobic CO dehydrogenases. The genomic potential and physiological properties of these *Kyrpidia* strains seem to be quite well adapted to thrive in the harsh environmental volcanic conditions.

## Introduction

Volcanic and geothermal areas represent the result of the dynamics involving the deeper layers of the earth and resulting in the emission of several gases from volcanic soils. In general, geothermal gases are mainly composed of H_2_O and CO_2_ as the most dominant species, and other minor species as the more reduced gases H_2_S, CH_4_, H_2_, CO, and NH_3_ (Oppenheimer et al., 2014). The abundance of the latter gases, and consequently their impact to the atmosphere, is strictly related to the energy of the system, the water-rock interactions, gas-gas interactions and also to the gas-biota interactions. In fact, the minor gas species represent the driving forces for the establishment of an active microbial community (Shock et al., 2010; Lindsay et al., 2019). Commonly, geothermal soils are characterized by high temperatures and often a low pH, which may be caused by the presence of H_2_S and CS_2_ in the emitted gases and their conversion to sulfuric acid by sulfide-oxidizing microbes (Chiodini et al., 2001; D’Alessandro et al., 2009; Smeulders et al., 2011, 2013; Quatrini and Johnson, 2018). Despite the harsh conditions, these hot and acidic terrestrial areas harbor distinctive microbial communities (Colman et al., 2019a, b; Lindsay et al., 2019). Analyses of soil microbial communities show that at low pH and high temperature, the dominant phyla differ from areas with moderate temperature and neutral pH (Strazzulli et al., 2017). Beside temperature and acidity, the available nutrients determine the microbial composition.

Gagliano et al. (2016) showed that 99% of the sequences obtained at the FAV1 site at the geothermal soils of the Favara Grande (Pantelleria Island) could be assigned to four main phyla: Proteobacteria, Firmicutes, Actinobacteria and Chloroflexi. At the FAV2 site 98% of the sequences were distributed in only three phyla Proteobacteria, Firmicutes and Actinobacteria. Especially Chloroflexi were much less represented than in FAV1. Apparently sites with low NH_4_^+^ concentration are dominated by a diverse methanotrophic community, whereas high NH_4_^+^ soils harbor thermo-acidophilic chemolithotrophs, despite CH_4_ being present. Alphaproteobacterial methanotrophs were isolated from the low NH_4_^+^ site (Gagliano et al., 2014). Previously, key players in the conversion of H_2_S, CS_2_, and CH_4_ from the Solfatara geothermal area near Naples, Italy, have been isolated and characterized (Pol et al., 2007; Smeulders et al., 2011, 2013; van Teeseling et al., 2014). Besides H_2_S, CS_2_ and CH_4_, H_2_ and CO are available and suitable electron donors for novel chemolithoautotrophs in geothermal soils. In this study, we focused on H_2_ and CO-utilizing bacteria from the Favara Grande geothermal site on Pantelleria Island, Italy. These hot and acidic soils are characterized by H_2_ (5-168,000 ppm) and CO (1.6-26 ppm) emissions (D’Alessandro et al., 2009) and *in situ* H_2_ consumption was already observed (Gagliano et al., 2016). Both H_2_ and CO have a high potential as electron donor for microbial growth.

H_2_ is an important electron donor in acidic and neutral hot springs (Spear et al., 2005) and can be used in a lithotrophic lifestyle under aerobic or anaerobic conditions. Oxidation of H_2_ is catalyzed by the enzyme hydrogenase, which can be classified as [NiFe]-, [FeFe]-, or [Fe]-hydrogenase depending on the metal ion in the active site (Lubitz et al., 2014). Hydrogenases are found among many different phyla, such as Proteobacteria, Firmicutes, Cyanobacteria, Chloroflexi, Verrucomicrobia, Aquificae, Euryarchaeota and Crenarchaeota (Greening et al., 2015). Aerobic H_2_-oxidizing microorganisms are referred to as “Knallgas” bacteria.

CO is a highly reactive gas, and often it is not detected due to field and laboratory equipment detection limits. Despite being a minor species in the hydrothermal gas mixture, CO analysis is very important because any variation in its amount may indicate some variations of the hydrothermal system. CO can be oxidized under aerobic and anaerobic conditions (Robb and Techtmann, 2018). Anaerobic CO-oxidizing bacteria have been detected in geothermal springs of neutral pH (Brady et al., 2015). In addition, anaerobic microbial CO consumption has been observed in acidic hot spring mud of pH 2.9 (Ikeda et al., 2015). In both cases, the microbial population was dominated by Firmicutes. Two distinct classes of CO dehydrogenases (CODH) catalyze CO transformation. The active site of the CODH from anaerobes contains a sulfur-coordinated Ni in a [Ni-4Fe-5S] cluster (Robb and Techtmann, 2018) and can be monofunctional producing CO_2_ or bifunctional producing acetyl-CoA and H_2_. Aerobic CO-oxidizers, or carboxydotrophs, possess heterotrimeric CO dehydrogenases with an active site containing a [Mo-Cu] cluster in the CoxL subunit. The other components are a flavoprotein (CoxM) and an iron-sulfur protein (CoxS). These enzyme complexes catalyze the unidirectional conversion of CO to CO_2_. The aerobic [Mo-Cu]-CO dehydrogenases can be categorized in two groups, Form I and Form II (King and Weber, 2007). Biochemical analysis showed that Form I has highest CO oxidation rates and is responsible for aerobic growth on CO. Form II is phylogenetically closer related to Form I CO dehydrogenase than to other enzymes of the molybdenum hydroxylases family. So far, no firm evidence is reported for CO oxidation by Form II enzymes, suggesting that Form II CO dehydrogenases may also catalyze the oxidation of a different substrate (Cunliffe, 2011).

In the upper layer of geothermal soils air is mixed with the geothermal gasses (Gagliano et al., 2016). Since soil porosity is higher than in the deeper soil horizon and the uprising hydrothermal flux lost part of its energy, this results in conditions where (micro)aerobic H_2_ and CO-oxidizing bacteria could thrive. Here, we used samples of the Favare grande soil to enrich H_2_-oxidizing microorganisms. After serial dilution to extinction of active enrichments we isolated and characterized two *Kyrpidia spormannii* species capable of using H_2_ and CO as sole energy source. We show that the genomic potential and physiological properties of the two *Kyrpidia* strains seem very suited to thrive in harsh volcanic conditions.

## Materials and Methods

### Geological Setting

Pantelleria Island is a quiescent volcano located in the Sicily Channel, and characterized by several hydrothermal manifestations as mofettes, fumaroles and passive degassing from geothermal soils. The main exhalative area is Favara Grande, where soil temperatures can reach 115°C at 5 cm of depth and soil pH may be down to 3. The geothermal field passively degases CO_2_, CH_4_, H_2_ in order of magnitude of percent per volume unit, and minor species as CO in the order of magnitude of ppm per volume unit (see Table 2 in D’Alessandro et al., 2009). Soil samples were taken in June 2017 at Favara Grande from two sites, FAV1 (23°21′80″N; 40°73′170″E) and FAV2 (23°21′77″N; 40°73160E) (Gagliano et al., 2016), using a core sampler (diameter 1.5 cm), divided into subsections of 5 cm and stored in sterile 50 ml tubes at room temperature.

### Enrichment and Isolation

The cultivation medium based on geochemical data (Gagliano et al., 2016) was composed of 0.5 mM MgCl_2_.H_2_O, 0.5 mM CaCl_2_.H_2_O, 1 mM Na_2_SO_4_, 2 mM K_2_SO_4_, 1 mM (NH_4_)_2_SO_2_ and 1 mM NaH_2_PO_4_.H_2_O. The final trace element concentrations were 1 μM CoCl_2_.6H_2_O, NaMoO_4_.2H_2_O, Na_2_SeO_3_, CeCl_3_.6H_2_O and ZnSO_4_.7H_2_O, 5 μM MnCl_2_.4H_2_O and FeSO_4_.7H_2_O and 10 μM CuSO_4_.5H_2_O and NiCl_2_.6H_2_O, with 50 μM NTA as complexing agent. The pH was set to 3.0 or 5.0 by adding 1M H_2_SO_4_ or 1M NaOH.

Within 6 h after taking the samples, the soil was mixed with sterile minimal medium of either pH 3 or pH 5. The mixtures were shaken for 10 min to extract the microorganisms from the soil, after which the suspensions were left settling for a few minutes. One ml of the liquid phase was transferred to a sterile 60 ml bottle, containing 10 ml sterile medium, either pH 3 or pH 5, and 87% (v/v) N_2_, 10% (v/v) CO_2_, 1.5% (v/v) air and either 1.5% (v/v) CO or H_2_. Immediately after this, 1 ml of the first batch is transferred to a new bottle (10x diluted). The bottles were stored at room temperature and after 24 h, the incubations were transferred to a shaking incubator operated at either 50 or 60°C and 50 rpm. Bottles that showed H_2_ or CO consumption were serial diluted to extinction to obtain pure cultures.

### Batch Cultivation

Growth experiments were performed in triplicate in 120 ml flasks with 20 ml medium. The headspace contained 10% (v/v) H_2_ or CO, 5% (v/v) O_2_, 5% (v/v) CO_2_ and 80% (v/v) N_2_. 2-(N-morpholino)ethanesulfonic acid (25 mM) was used to buffer the medium. Bottles were incubated at 55°C, unless stated otherwise, in a shaking incubator operating at 250 rpm. To test the growth on organic substrates, H_2_ or CO was replaced by the organic substrate (25 mM) or 1 g/l yeast extract. To test nitrogen fixation, medium without ammonium was used. To test for growth on urea, ammonium was replaced by 2 mM urea.

### Continuous Culture

Cultivation was performed in a 500 ml bioreactor (Applikon, Delft, Netherlands) with a working volume of 350 ml and the medium described above. The temperature was maintained at 55°C using a Peltier element. The pH was set to 5.0, measured by a pH electrode (Applikon, Delft, Netherlands) and maintained at pH 5.0 ± 0.1 by adding 0.2 M NaOH. The dissolved oxygen (DO) concentration was measured by a DO electrode (Applikon, Delft, Netherlands). The airflow was regulated to maintain a dissolved oxygen concentration of 5% air saturation. Temperature, pH and DO were controlled using the in-Control process controller (Applikon, Delft, Netherlands). The reactor was stirred at 1000 rpm using a stirrer with two Rushton impellers. The reactor was supplied with air (regulated), 9 ml.min^–1^ CO_2_-Argon (5%:95%, v/v), 2.5 ml.min^–1^ H_2_ and operated at a dilution rate of 0.045 h^–1^. Growth under this conditions is H_2_ limited since all other substrates are in excess.

### Gas Analysis

H_2_ and CO_2_ in the headspace of the bottles and the in- and outflow of the chemostat cultures were measured using a HP 5890 gas chromatograph (Agilent, Santa Clara, California) equipped with a Porapak Q column (1.8 m, ID 2 mm) and a thermal conductivity detector. For this analysis, 50–100 μl gas samples were injected. To determine the CO and O_2_ consumption, 25–100 μl gas was injected and measured on an Agilent series 6890 GC-MS (Agilent, Santa Clara, CA, United States) and analyzed as described before (Ettwig et al., 2008).

### Optical Density and Dry Weight

The optical density was measured using a Cary 50 UV-VIS spectrophotometer at a wavelength of 600 nm (Agilent, Santa Clara, California). Dry weight was determined as described before (Mohammadi et al., 2016).

### Membrane-Inlet Mass Spectrometry (MIMS)

Liquid concentrations of H_2_ were measured by Membrane Inlet Mass Spectrometry (HPR40, Positive Ion Counting detector, Hiden Analytical, Warrington, United Kingdom) in a 10 ml chamber. Briefly, the MIMS probe mounted with a 10 μm thin silicon (Hiden Analytical, Warrington, United Kingdom) or PTFE (Hansatech, Pentney, United Kingdom) membrane (8 mm^2^) was inserted inside of the bottom part just above the glass stirrer bar (1000 rpm). Liquid was equilibrated with the desired gas by bubbling via a metal capillary. The liquid volume was adjusted with a piston removing gas bubbles (8–9 ml). All additions were done via the metal capillary by gastight syringes (Hamilton, Reno, Nevada). Signal of mass 2 was not only derived from H_2_ but resulted also from water and gases such as CO_2_. Therefore, water vapor and other gases that pass the silicon/PTFE membrane were trapped using a coiled part inserted in liquid nitrogen before entering the mass spectrometer. In this way, the background mass 2 signal was minimized. For calibrations, known amounts of gas-saturated water were administered from 50 ml serum bottles containing 10 ml of water. The medium and pH set was identical to the growth medium.

### DNA Sequencing and Genome Reconstruction

For DNA isolation 2 ml cell suspension (OD_600_ 0.5–1.0) from the continuous culture was harvested by centrifugation (2 min, 14,000 × g) and resuspended in 100 μl sterile MQ water. DNA was extracted with the PowerSoil DNA isolation kit or the DNeasy Blood and Tissue kit according to the manufacturer’s instructions (Qiagen Benelux B.V, Venlo, The Netherlands). The quality and quantity of the DNA was analyzed using the Qubit (Thermo Fisher Scientific, Waltham, MA, United States) and the Agilent 2100 Bioanalyzer (Thermo Fisher Scientific, Waltham, MA, United States).

The genome was reconstructed using a combination of short-read Illumina sequencing and long-read Nanopore sequencing. For Illumina library preparation, the Nextera XT kit (Illumina, San Diego, CA, United States) was used according to the manufacturer’s instructions. Enzymatic tagmentation was performed starting with 1 ng of DNA, followed by incorporation of the indexed adapters and amplification of the library. After purification of the amplified library using AMPure XP beads (Beckman Coulter, Indianapolis, IN, United States), libraries were checked for quality and size distribution using the Agilent 2100 Bioanalyzer and the High sensitivity DNA kit. Quantitation of the library was performed by Qubit using the Qubit dsDNA HS Assay Kit (Thermo Fisher Scientific, Waltham, MA, United States). The libraries were pooled, denatured and sequenced with the Illumina Miseq sequence machine (San Diego, California). Paired end sequencing of 2 × 301 base pairs was performed using the MiSeq Reagent Kit v3 (Illumina, San Diego, CA, United States) according to the manufacturer’s protocol.

For Nanopore library preparation, 1–1.5 μg of DNA was used. The input DNA was checked for high molecular DNA and absence of degradation by agarose (0.5%) gel electrophoresis. For Nanopore sequencing the DNA Library construction was performed using the Ligation Sequencing Kit 1D (SQK-LSK108) in combination the Native barcoding Expansion Kit (EXP-NBD103 or EXP-NBD104) according to the manufacturers protocol (Oxford Nanopore Technologies, Oxford United Kingdom). The libraries were loaded and sequenced on a Flow Cell (R9.4.1) and run on a MinION device (Oxford Nanopore Technologies, Oxford, United Kingdom), according to the manufacturer’s instructions. Base calling after sequencing was done using the guppy_basecaller in combination with guppy_barcoder (Oxford Nanopore Technologies, Limited Version 2.3.7).

The genome was assembled from Nanopore reads using Canu (v1.8) (Koren et al., 2017). Assembled contigs were first polished with Racon (v1.3.1) (Vaser et al., 2017) followed by two iterations of Pilon (v1.23) polishing with Illumina reads (Walker et al., 2014). The genome was annotated using the MicroScope platform (Vallenet et al., 2013) and annotations were checked manually.

### Phylogenomic Tree Reconstruction

Bacterial genome-based phylogenetic analyses were performed using the up-to-date core gene (UBCG) set and pipeline for phylogenomic tree reconstruction (Na et al., 2018). Extracted genes of the genomes of the isolates and the reference genomes were aligned and concatenated using UBCG with default parameters. Maximum likelihood phylogenetic trees were made from the concatenated nucleotide alignment using RAxML version 8.2.10 (Stamatakis, 2014) on the CIPRES science gateway (Miller et al., 2012) with the GTR substitution and GAMMA rate heterogeneity models and 100 bootstrap iterations.

### RNA Sequencing and RNA-Seq Analysis

For transcriptome analysis, triplicate samples each of 10 ml cell suspension (OD_600_ 0.5) were taken from the continuous culture and harvested by centrifugation. mRNA was isolated using the RiboPure^TM^-Bacteria kit according to the manufacturer’s protocol (Thermo Fisher Scientific, Waltham, MA, United States). The quality and quantity of the RNA was analyzed using the Qubit (Thermo Fisher Scientific, Waltham, MA, United States) and the Agilent 2100 Bioanalyzer (Thermo Fisher Scientific, Waltham, MA, United States). The transcriptome libraries were constructed using the TruSeq^®^ Stranded mRNA Library Prep protocol (Illumina, San Diego, CA, United States) according to the manufacturer’s instructions. Total mRNA was used for library preparation and obtained libraries were checked qualitatively and quantitatively as described above. Pooled libraries were sequenced using the Illumina Miseq sequence machine (Illumina, San Diego, CA, United States). For sequencing the 151 bp sequence chemistry was performed using the MiSeq Reagent Kit v3 (Illumina, San Diego, CA, United States) according to the manufacturers protocol in one direction. CLCBio software (version 10.1.1, Qiagen, Aarhus, Denmark) was used to perform RNA-seq analysis. Gene expression levels were compared by calculating the reads per kilobase per million reads (RPKM) values for the CDSs and calculating the log_2_-fold to median (Mundinger et al., 2019).

### 16S rRNA Gene Analysis

The 16S rRNA gene was PCR amplified from isolated DNA using the primers 616F (AGAGTTTGATYMTGGCTCAG) and 1492R (GGTTACCTTGTTACGACTT) using the PCR program 5 min 94°C, 30 cycles 40 s at 96°C, 40 s 55°C, 40 s 72°C and finally 10 min 72°C. The amplicon was cloned into the pGEM-T Easy cloning vector (Promega) and transformed into competent *E. coli* cells. After growth of the cells, the 16S rRNA amplicon in the vector was PCR amplified, cleaned (GeneJET PCR purification kit, Thermo Fisher Scientific, Waltham, MA, United States) and sequenced using the Sanger sequencing platform (BaseClear B.V., Leiden, the Netherlands).

### Deposition of Cultures and Sequences

The two isolated strains were deposited to the DSMZ culture collection as *Kyrpidia spormannii* FAVT5 (DSM 109470) and *Kyrpidia spormannii* COOX1 (DSM 109471). The genomes of the two strains are available at the MaGe platform (accession numbers KFAV.1 and KSCOOX1.1)^1^.

## Results and Discussion

### Enrichment and Isolation

In this study, aerobic H_2_ and CO-oxidizing soil bacteria from the geothermal active area of Favara Grande on the island of Pantelleria, Italy were enriched, isolated and characterized. The primary enrichment minimal medium (low nutrient, autotrophic) was based upon geochemical analyses (Gagliano et al., 2016). The incubations were performed at two different pH values, pH 3 and pH 5, and at either 50 or 60°C with H_2_ or CO as electron donor. All enrichment cultures were checked for H_2_ or CO consumption, after which the most active cultures (pH 5, 60°C, from FAV1) were diluted to extinction using fresh medium. All bottles containing H_2_ in the headspace showed H_2_ consumption. After three transfers, a 16S rRNA gene analysis of these active cultures was performed and revealed that in all cultures the same microorganism was present and dominant (>95%). Therefore, only one culture was used to continue the isolation. After another three consecutive rounds of serial dilutions this resulted in the isolation of strain FAVT5. In contrast, only one of the enrichments with CO as electron donor showed activity, the one incubated at pH 5 and 60°C and inoculated with soil of FAV1. This culture was serial diluted to extinction for five consecutive rounds, resulting in the isolation of the strain COOX1.

### Genome Sequencing and Phylogeny

The genomes of both isolates, FAVT5 and COOX1, were sequenced and assembled using a combination of short read Illumina sequencing and long read Nanopore MinION sequencing. This resulted in closed genomes and the general features of both genomes are compiled in Table 1. The genomes are both 3.3 Mb in size and have a GC content of 59% and both contain five complete rRNA operons (16S, 23S, 5S) and 59 tRNAs with 1–5 copies per tRNA type. The 16S rRNA gene sequences in the genomes were identical to the sequences obtained by PCR analysis of the enrichments (see above).

**TABLE 1:** Genomic features of *K. spormannii* strains FAVT5 and COOX1.

Feature	Strain FAVT5	Strain COOX1
Genome size (bp)	3,312,578	3,310,423
DNA coding (bp)	2,933,288	2,945,945
DNA G+C (%)	58.96	58.95
Total genes	3,594	3,598
Protein coding genes	3,478	3,500
rRNA genes	15	15
tRNA genes	59	59
Pseudo genes	42	24
Genes assigned to COGs	2717	2724

**FIGURE 1:**
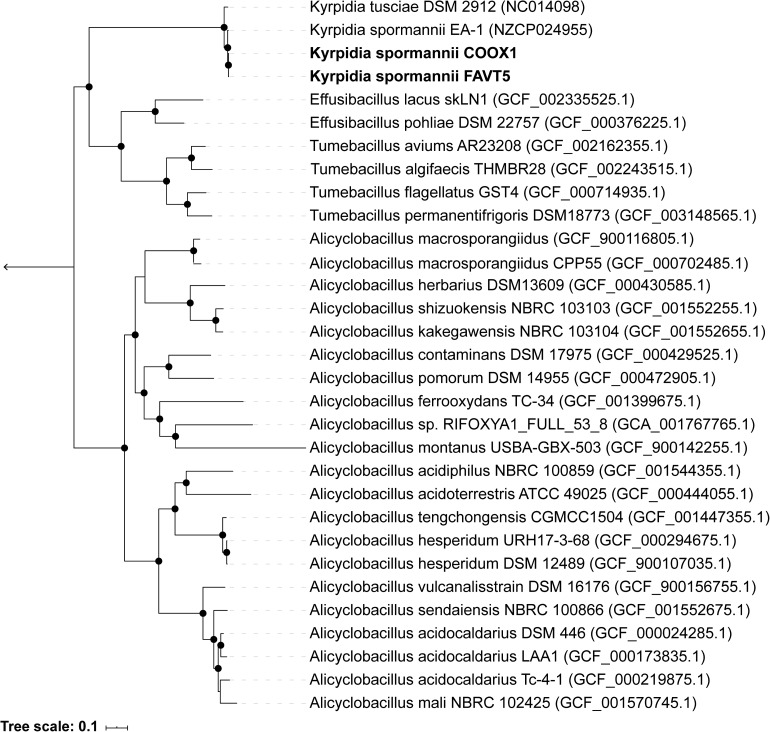
Up-to-date Bacterial Core Gene (UBCG) phylogenetic tree of the two isolates and members of the family Alicyclobacillaceae. *Hyphomicrobium denitrificans* was used to root the tree, but removed from the tree for clarity. The tree was constructed using RAxML. Bootstrap analysis was carried using 100 replications and percentage bootstrap values > 95% are indicated by a black dot at the nodes.

The 16S rRNA gene sequences (5 copies in each isolate) showed a high identity with each other (98.7–100%) (Supplementary Table S1 and Supplementary Figure S1), indicating that both strains belong to the same species. Some studies claim that 16S rRNA gene identity is not suitable for phylogeny and that better results can be obtained using genome-based phylogeny (Mahato et al., 2017). Therefore, an up-to-date bacterial core gene (UBCG) phylogenetic tree, based on 92 concatenated core gene alignments, was constructed. Our two isolates clustered within the genus *Kyrpidia* (Figure 1). The genus *Kyrpidia*, with thus far two cultured representatives, is the second genus in the parent family Alicyclobacillaceae (Klenk et al., 2011). *Kyrpidia* species, including our isolates, are Gram-stain-positive, aerobic, endospore-forming, non-motile rods (0.7–1.2 × 4–8 μm) (Reiner et al., 2018). To distinguish between species level, the average nucleotide identity (ANI) was calculated (Goris et al., 2007). These two isolates showed an ANI of 98.9% with each other and 97.4 and 97.5% with *Kyrpidia spormannii* EA-1 (Supplementary Table S2). ANI uses a species boundary of 95–96% identity (Chun et al., 2018), indicating that our two isolates are two novel strains of the species *Kyrpidia spormannii*, which we named *K. spormannii* strain FAVT5 and *K. spormannii* strain COOX1.

So far, two other *Kyrpidia* species have been isolated from geothermal areas. *K. spormannii* EA-1 was isolated from geothermal soils on the Azores, São Miguel, Portugal (Reiner et al., 2018) and *K. tusciae* was isolated from a geothermal pond in Tuscany, Italy (Bonjour and Aragno, 1984). 16S rRNA gene sequencing of environmental samples showed that members of the genus *Kyrpidia* were also found in sugarcane bagasse feedstock piles which are slightly acidic with a temperature of 49–52°C (Rattanachomsri et al., 2011). Other sequence matches were with biofilms of microbial fuel cells operated at 55°C and inoculated with biomass from a methanogenic anaerobic digester (Wrighton et al., 2008). Gagliano et al. (2016) performed 16S rRNA amplicon sequencing of the geothermal soils of our sampling site and this revealed the presence of three amplicon sequences related to *K. spormannii* within these soils (Supplementary Figure S1).

### Autotrophic Growth on H_2_

Since the two strains are phylogenetically very closely related and both grew on H_2_ and CO as sole source of energy, all physiological studies were performed with *K. spormannii* FAVT5. The optimal pH and temperature were determined during batch experiments with H_2_ as electron donor and CO_2_ as carbon source. The highest growth rate was achieved at pH 5, but growth occurred as low as pH 3. The strain has an optimal growth temperature of 55°C at which a maximal growth rate of 0.19 ± 0.02 h^–1^ was calculated (doubling time of 3.6 h) (Supplementary Figure S2). Spores were formed during the stationary phase (Supplementary Figure S3). To determine the stoichiometry of *K. spormannii* FAVT5, a H_2_-limited chemostat (D = 0.045 h^–1^) was started. Based on dry weight, 4.3 g DW was produced per mole H_2_ consumed and the following stoichiometry was obtained:

$$H_{2}+0.36O_{2}+0.14CO_{2}\to 0.14CH_{2}O+0.86H_{2}O$$

The yield of biomass on H_2_ is slightly lower compared to other “Knallgas” bacteria, such as *Ralstonia eutropha* (4.6 g DW/mol H_2_) (Morinaga et al., 1978) or *Hydrogenomonas eutropha* (5 g DW/mol H_2_) (Bongers, 1970), but higher than reported for *Methylacidiphilum fumariolicum* SolV (3.4 g DW/mol H_2_) (Mohammadi et al., 2016).

### Growth on Other Substrates

The *K. spormannii* FAVT5 repertoire of alternative electron donors besides H_2_ was tested. Compared to H_2_ the growth rate on CO was reduced to a doubling time of 15.7 h (μ_max_ = 0.04 ± 0.01 h^–1^). Furthermore, *K. spormannii* FAVT5 grew on the alcohols ethanol, propanol and butanol, but not on methanol (Table 2). In addition, the volatile fatty acids acetate, propionate and butyrate supported growth, but not formate. Growth on yeast extract and succinate was observed (Table 2). No growth was observed on the alkanes, methane, ethane, propane or butane. No growth occurred on oxaloacetate, citric acid, α-ketoglutarate, pyruvate, fumaric acid or malic acid. No growth was observed on the following sugars: glucose, galactose, fructose, maltose, ribose and lactose. This is in contrast to *K. spormannii* EA-1, since this strain could utilize pyruvate and sugars (Reiner et al., 2018). However, *K. tusciae* did not show growth on sugars and pyruvate either (Bonjour and Aragno, 1984). As for *K. tusciae*, the highest growth rate was obtained during growth on H_2_.

**TABLE 2:** Growth rates of *K. spormannii* strain FAVT5 on different electron donors, different nitrogen sources and different amino acids.

Energy source^a^	Growth rate μ (h^–1^)
H_2_	0.19 ± 0.02
CO	0.04 ± 0.01
Ethanol	0.05 ± 0.01
Propanol	0.16 ± 0.01
Butanol	0.18 ± 0.01
Acetate	0.16 ± 0.01
Propionate	0.09 ± 0.01
Butyrate	0.20 ± 0.01
Succinate	0.14 ± 0.01
Yeast extract	0.19 ± 0.01
**Nitrogen Source^b^**	
Ammonium	0.19 ± 0.02
Urea	0.09 ± 0.01
N_2_	0.14 ± 0.02
**Energy and Nitrogen Source**	
Alanine	0.06 ± 0.01
Valine	0.11 ± 0.01
Isoleucine	0.05 ± 0.01
Phenylalanine	0.06 ± 0.01

*^a^Ammonium was used as nitrogen source. ^b^Hydrogen was used as energy source. The values are the average ± standard deviation of three replicates.*

*K. spormannii* FAVT5 can use the following amino acids as energy and nitrogen source: alanine, valine, isoleucine, and phenylalanine. As nitrogen source, ammonium, urea and nitrogen gas can be used, but no growth was observed with nitrate or nitrite. Nitrogen fixation was tested under low O_2_ concentration (max. 5%, v/v). There is a difference in utilization of nitrogen sources between the different *Kyrpidia* species. *K. spormannii* EA-1 does not grow using urea or N_2_ as nitrogen source. The genome of this strain does not contain a urease gene, but the *nif*-genes could be identified (Reiner et al., 2018). The genome of *K. tusciae* contains an urease and growth on urea has been reported, but no N_2_ fixation has been observed (Bonjour and Aragno, 1984; Klenk et al., 2011).

### Kinetics of H_2_ Consumption

**FIGURE 2:**
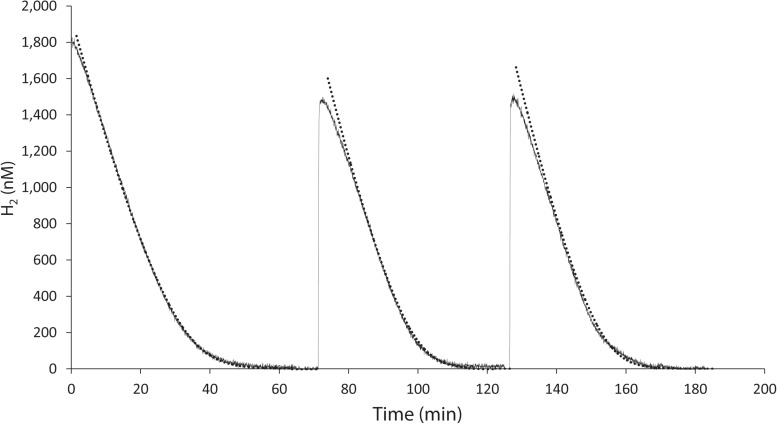
H_2_ uptake by *K. spormannii* strain FAVT in the MIMS-system (black line) and calculated hydrogen concentration based on Michaelis-Menten kinetics (dashed line).

*K. spormannii* FAVT5 was isolated from a volcanic soil, and soil microorganisms can have an extremely high affinity for H_2_ and oxidize atmospheric H_2_ (Novelli et al., 1999). Therefore, we tested the affinity for H_2_ of *K. spormannii* FAVT5. The kinetics were measured using Membrane-Inlet Mass Spectrometry (MIMS). For these experiments, the cells were grown in batch and during exponential phase transferred to the MIMS chamber. After addition of H_2_ to the chamber, H_2_ consumption started immediately and the maximal H_2_ oxidation rate was achieved within 1 min (Figure 2). The H_2_ depletion followed Michaelis-Menten kinetics and, resulted in a V_max_ of 60 ± 3 nmol H_2_/min/mg DW and the strain has a high affinity for H_2_, since a K_s_ of 327 ± 24 nM was found. These high affinities are also observed for different Actinobacteria, Acidobacteria, Chloroflexi and Verrucomicrobia (Constant et al., 2010; Greening et al., 2014; Myers and King, 2016; Mohammadi et al., 2016; Islam et al., 2019), however such a high affinity for H_2_ was not yet reported for any strain belonging to the phylum of Firmicutes.

### Hydrogenases in the Genomes

High affinity hydrogenases belong to either the group 1h or group 2a [NiFe]-hydrogenases (Constant et al., 2010; Greening et al., 2014; Myers and King, 2016; Mohammadi et al., 2016; Islam et al., 2019). The genomes of *K. spormannii* FAVT5 and *K. spormannii* COOX1 were checked for the presence of all known hydrogenase genes. Both strains possess two hydrogenases, all belonging to the oxygen tolerant group 2a [NiFe] hydrogenases (hydDB classification) (Greening et al., 2015). Group 2a hydrogenases are typically found in Actinobacteria (Greening et al., 2015) or Cyanobacteria to recycle H_2_ derived from nitrogen fixation (Tamagnini et al., 2007). Group 2a [NiFe] hydrogenases are classified as H_2_-uptake hydrogenases and some have a high affinity for H_2_. For example, the group 2a hydrogenase of the Actinobacteria *Mycobacterium smegmatis* mc^2^ has a K_m(app)_ of 180 nM for H_2_ (Greening et al., 2014), showing a bit higher affinity for H_2_ compared to *K. spormannii* FAVT5.

**FIGURE 3:**
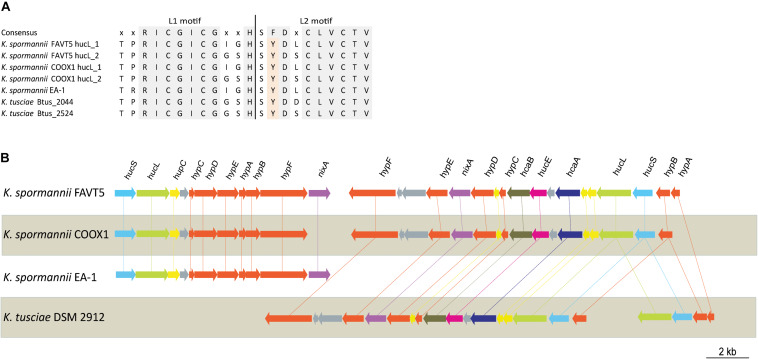
**(A)** Multiple sequence alignment of L1 and L2 motif of the [NiFe] hydrogenase large subunit of the different *Kyrpidia* species and strains. **(B)** Gene arrangement of the hydrogenases in different *Kyrpidia* isolates. Encoded proteins are colored as follows: green = large subunit, blue = small subunits, yellow = (putative) maturation proteins, orange = accessory proteins, purple = nickel transporter, pink = FeS cluster protein, dark purple = TTP repeat domain protein and dark green = NHL repeat domain protein.

The hydrogenase large subunits (HucL) of all *Kyrpidia* sp. contain the L1 and L2 motifs typical for group 2a [NiFe] hydrogenases. The two cysteine residues, that bind the metal ions, are well conserved. However in all *Kyrpidia* sp. the phenylalanine in the L2 motif is replaced by a tyrosine (Figure 3). Changes in neighboring residues are assumed to affect the catalytic behavior of the hydrogenases (Greening et al., 2015).

**FIGURE 4:**
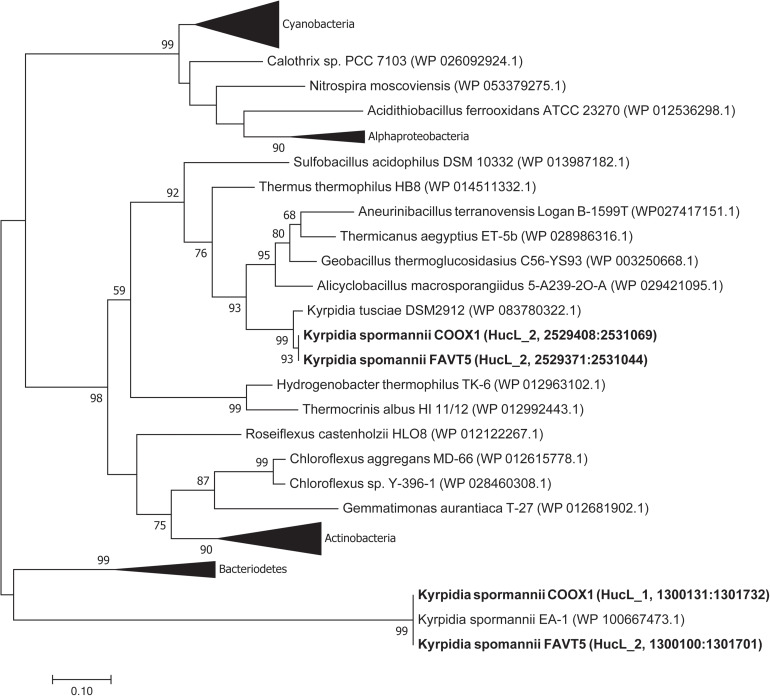
Phylogenetic tree of the group 2a [NiFe] hydrogenases. The tree was constructed using the Maximum Likelihood method (Saitou and Nei, 1987). Bootstrap percentage values above 50% (1000 replicates) are given at each node. Evolutionary distances were calculated using the Poisson correction method (Zuckerkandl and Pauling, 1965). The analysis involved 74 amino acid sequences and was performed using MEGA7 (Kumar et al., 2016).

Phylogenetic analysis of the large subunits of our strains reveals that one of the goup 2a [NiFe] hydrogenases clustered together with the [NiFe] hydrogenase of *K. tusciae*, whereas the other hydrogenases clustered outside the group 2a [NiFe] hydrogenase phylogenetic tree, together with the hydrogenase of *K. spormannnii* strain EA-1 (Figure 4). Based on amino acids, these latter hydrogenases showed little identity (55%) with the other [NiFe] hydrogenases belonging to class 2a. Also, the identity of the small subunit of this hydrogenase to other group 2a types was low (41%).

The two group 2a [NiFe] hydrogenase gene clusters are differently arranged. Figure 3 shows the hydrogenase gene arrangement of the *K. spormannnii* strains FAVT5, COOX1, EA-1 and *K. tusciae*, whereby the first gene cluster encodes for the distinct *Kyrpidia* group 2a [NiFe] hydrogenase and the second operon encodes a more conventional group 2a [NiFe] hydrogenase. The *hypABCDEF* genes coding for accessory proteins were found in both operons, although they are differently arranged. The gene encoding for HypD was only found in the first operon. This protein is needed to cleave off the C-terminus from the large subunit to ensure that the small subunit can bind. However, it is expected that KFAV_v1_2744 fulfills this job in the hydrogenases encoded by the second operon, since a Pfam motif search annotated the encoded protein as HycI maturation protein. In *K. spormannii* COOX1, the high affinity nickel transporter was only found in the conventional hydrogenase operon, whereas in *K. spormannii* FAVT5, both operons contain a high affinity nickel transporter. The FeS cluster was only found in the second/conventional operon. Hydrogenase type 2a operons often contain genes with a TTP repeat domain (*hcaA*) and a NHL repeat domain (*hcaB*) (Greening et al., 2015). The functions of these domains remain unclear, and these genes are only found in the second, conventional hydrogenase operon.

### CO Dehydrogenases in the Genome

**FIGURE 5:**
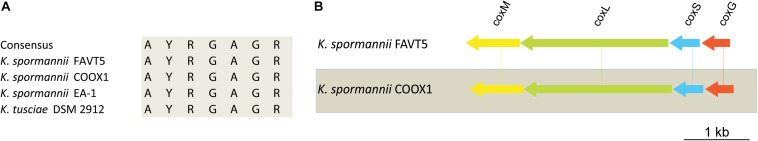
**(A)** Multiple sequence alignment of the active site of Form II CO dehydrogenase of the different *Kyrpidia* species. **(B)** Gene arrangement of the CO dehydrogenases in *K. spormannnii* FAVT5 and *K. spormannii* COOX1. Genes are color coded as follows: green = large subunit, blue = small subunit, yellow = medium subunit, orange = accessory protein.

*K. spormannii* COOX1 was isolated from a CO enrichment culture. Although originally isolated on H_2_, *K. spormannii* FAVT5 also grows on CO as sole energy source. A maximal growth rate of 0.04 ± 0.01 h^–1^ (Table 2) was observed, which is much lower compared to growth on H_2_. The genomes of both *K. spormannii* FAVT5 and *K. spormannii* COOX1 were analyzed for CO dehydrogenase genes. Both genomes encode for one candidate CO dehydrogenase gene cluster. The large subunits of all *Kyrpidia* strains contain the active site with the amino acid motive AYRGAGR, which groups them as a Form II CO dehydrogenases (Figure 5). BlastP searches with the *Oligotropha carboxidovorans* Type I CoxL (AEI08106) did not give a hit in the high quality closed genomes of strains FAVT5 and COOX1 and the very closely related *K. tusciae*. The *K. spormannii* EA-1genome encodes for both types of CoxL. The corresponding gene order in our strains, *coxSLM*, is characteristic for Form II CO dehydrogenases (King and Weber, 2007). This gene cluster is complemented with *coxG* (Figure 5), which encodes the pleckstrin homology (PH) domain protein. This domain binds to lipids, resulting in the hypothesis that CoxG is involved in anchoring the CO dehydrogenase to the cytoplasmic membrane to improve electron transfer to the respiratory chain (Pelzmann et al., 2014).

**FIGURE 6:**
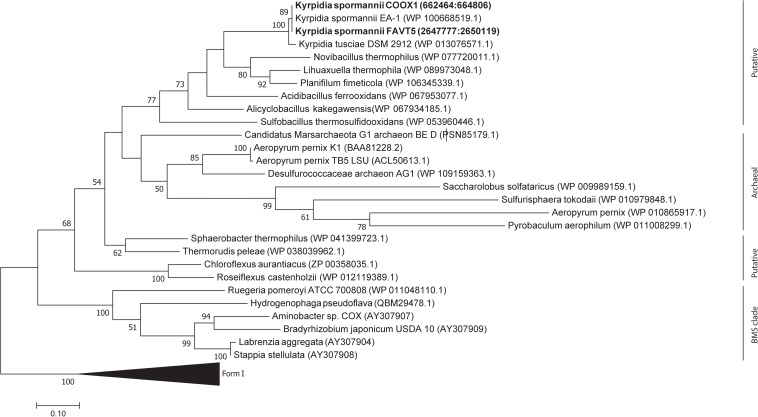
Phylogenetic tree of the CO dehydrogenase large subunit, form I and II. The tree was constructed using the Maximum Likelihood method. Bootstrap percentage values above 50% (1000 replicates) are given at each node. Evolutionary distances were calculated using the Poisson correction method (Zuckerkandl and Pauling, 1965) and the analysis was performed in MEGA7 (Kumar et al., 2016).

Phylogenetic analysis of the CoxL proteins of *K. spormannii* FAVT5 and *K. spormannii* COOX1 shows that these proteins cluster with Form II CO dehydrogenases (Figure 6). Most biochemical evidence for Form II CODH is obtained from members of the BMS (*Burkholderia*, *Mesorhizobium*, *Sinorhizobium*) clade, a group of Form II CODH found in α-Proteobacteria (Nunoura et al., 2005; Wu et al., 2017). It remains puzzling if the Form II enzymes are real CO dehydrogenases since the large subunit of type II-CODH does not possess the canonical motif bridging the active site of CODH (a cysteine residue is essential to accommodate the copper and molybdenum atoms of the active site). Recently, the CODH hydrogenase activity was observed for a putative Form II CODH from the thermophilic Archaeon *Aeropyrum pernix* TB5, which clusters outside the BMS clade (Nishimura et al., 2010). However, the enzyme was purified but no direct coupling of the purified protein with its encoding gene could be made. These strains oxidizes CO at heterotrophic and aerobic conditions and at high CO concentrations (>25%, v/v), however, growth on CO as sole electron source is impossible by this Archaeon, since it lacks a CO_2_ fixation pathway. This CO dehydrogenase could be purified and shows highest activity at 95°C and is oxygen stable (Nishimura et al., 2010). The CODH of the different *K. spormannii* isolates cluster outside the BMS clade as well and are phylogenetically closer to the archaeal CODH (Figure 6).

### Transcriptomics

**FIGURE 7:**
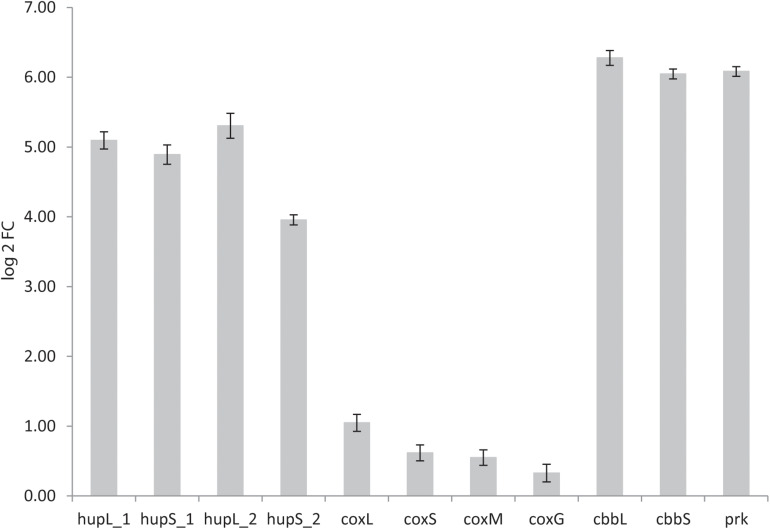
Log 2fold change of reads per kilobase per million reads (RPKM) values over the median RPKM. The values are the average of three biological replicates (chemostat samples taken on three different days). hupL_1, hydrogenase large subunit 1 (FAVT5_v1_1432); hupS_1, hydrogenase small subunit 1 (FAVT5_v1_1431); hupL_2, hydrogenase large subunit 2 (FAVT5_v1_2745); hupS_2, hydrogenase small subunit 2 (FAVT5_v1_2746); coxL, CO dehydrogenase large subunit; coxS, CO dehydrogenase small subunit; coxM, CO dehydrogenase medium subunit; coxG, pleckstrin homology (PH) domain protein; cbbL, ribulose bisphosphate carboxylase large chain; cbbS, ribulose bisphosphate carboxylase small chain; prk, phosphoribulokinase.

The genomes of both *K. spormannii* FAVT5 and *K. spormannii* COOX1 contain 2 group 2a [NiFe] hydrogenase gene clusters, one of which is phylogenetically distinct from other group 2a hydrogenases (Figure 4). To test whether both hydrogenases are being expressed, transcriptome analysis (RNAseq) was performed on strain FAVT5 cells from the H_2_-limited chemostat culture. The results of the transcriptome analysis were calculated as log2fold change of the RPKM-value compared to the median expression level (Mundinger et al., 2019). In this way, the genes that show expression values above the median get positive values and genes with RPKM values below the median will give a negative log2fold change. The results of the transcriptome studies revealed that *K. spormannii* FAVT5 expressed its two hydrogenases under H_2_-limiting conditions and their expression values largely exceeded the median RPKM values (Figure 7). The expression values of the large and small hydrogenase subunits were amongst the highest expressed genes (Supplementary Table S4). The expression values (RPKM) were between 25 and 52 times higher than the median values. The other genes in the hydrogenase gene clusters showed high expression values too (Supplementary Table S4). The reason for having two hydrogenases remains unclear, but it is likely that the enzymes function under different conditions (Sanchez-Perez et al., 2008) as in *Methylacidiphilum fumariolicum* SolV. In fact, this methanotroph can grow as “Knallgas”-bacterium and possesses two different hydrogenases, an oxygen-sensitive (group 1d) and an oxygen-insensitive hydrogenase (group 1h), giving it the possibility to oxidize H_2_ at higher oxygen concentrations (Mohammadi et al., 2016). Detailed transcriptomic studies combined with biochemical studies are needed to elucidate under which conditions the two different hydrogenases in the novel *Kyrpidia* isolates function.

The bioreactor was fed with CO_2_ as carbon source. The transcriptomics data also show high expression levels of the genes encoding the small (*cbbS*) and large (*cbbL*) subunits of the RuBisCO enzyme and the phosphoribulokinase gene (prk), key enzymes of the Calvin–Benson–Bassham (CBB) cycle.

Although the bioreactor was not fed with CO, the CO dehydrogenase gene cluster showed expression values higher than the mean. It is possible that *K. spormannii* FAVT5 transcribed the CO dehydrogenases constitutively or that the expression increases under nutrient limitation, as was observed with the Chloroflexi *Thermomicrobium roseum.* This strain upregulates the expression of its CO dehydrogenase under nutrient starvation to oxidize atmospheric CO for microbial persistence (Islam et al., 2019).

### Genome Data Supporting Alternative Substrate Utilization

The genome of *K. spormannii* strain FAVT5 contains a urease gene and the *nif*-genes for growth on urea and performing nitrogen fixation. The genomic analysis revealed multiple alcohol dehydrogenases and two acetaldehyde dehydrogenases (Supplementary Table S3) for the heterotrophic growth on alcohols. Growth on sugars was not observed, despite all genes for the glycolysis pathway being present. Since the *K. spormannii* FAVT5 genome does not encode any sugar transport proteins, it is more likely that the gluconeogenesis and glycolysis will be used for the production and consumption of storage material. During H_2_-limited growth, the genes for the glycolysis pathway were expressed, and especially the genes for the fructose 1,6-bisphosphatase class II, fructose 1,6-bisphosphate aldolase and glyceraldehyde-3-phosphate dehydrogenase showed high expression levels (Supplementary Table S4). Furthermore, all genes for the TCA cycle, the glyoxylate cycle and the non-oxidative part of pentose phosphate pathway were found in the genome (Supplementary Table S3). All of these pathways were expressed under H_2_-limited growth and high expression levels were observed for ribose 5-phosphate isomerase and transketolase genes (Supplementary Table S4). Despite any organic substrates being present, these pathways were expressed for the synthesis of different biomolecules, such as nucleotides, amino acids and lipids.

## Conclusion

This study shows that two different strains of the species *Kyrpidia spormannii* were isolated from a geothermal site using CO and H_2_ as energy source. The two isolates were genetically very similar and strain FAVT5, isolated on H_2_ as sole energy source, was also able to grow on CO. Therefore, all physiology experiment were performed with *K. spormannii* FAVT5. This strain grows on H_2_, CO and a variety of organic substrates with an optimum at 55 °C and pH 5.0. The affinity for H_2_ is high, namely 327 ± 24 nM, the first high H_2_ affinity reported for Firmicutes so far. The genome encodes for two group 2a [NiFe] hydrogenases of which one is distantly related to other group 2a hydrogenases. Both encoded hydrogenases are expressed under H_2_-limiting conditions in a chemostat. The genome encodes for a candidate CO dehydrogenase gene cluster and these genes are transcribed during H_2_ limited growth. The presence of *Kyrpidia spormannii* strains at Favara Grande and their capability to grow on CO are highly important.

## Data Availability Statement

The genomes of strains FAVT5 and COOX1 are available under accession numbers GCA_902829265 and GCA_902829275. The transcriptome sequencing data are available under Project number PRJNA616194.

## Author Contributions

CH, AP, MJ, and HO designed the projects and experiments. CH, AP, NP, AG, WD’A, PQ, and HO sampled the geothermal soils. CH and NP performed the enrichment and isolation experiments. CH and AP conducted the experiments. GC and TA sequenced the genome and transcriptome and analyzed the reconstructed the genome. CH, AP, and HO carried out the data analysis. CH and HO wrote the manuscript. All authors contributed to revision of the manuscript, and read and approved the submitted version.

## Conflict of Interest

The authors declare that the research was conducted in the absence of any commercial or financial relationships that could be construed as a potential conflict of interest.

## Footnotes

1 https://www.genoscope.cns.fr/agc/microscope/
