# Supplementary material for: Hydrogen and Carbon Monoxide-Utilizing Kyrpidia spormannii Species From Pantelleria Island, Italy
Source: Front Microbiol. 2020 May 19;11:951. doi: 10.3389/fmicb.2020.00951 (PMC7248562; doi:10.3389/fmicb.2020.00951)
Supplement: Supplementary file 1 [file Data_Sheet_1.PDF]

Supplementary data

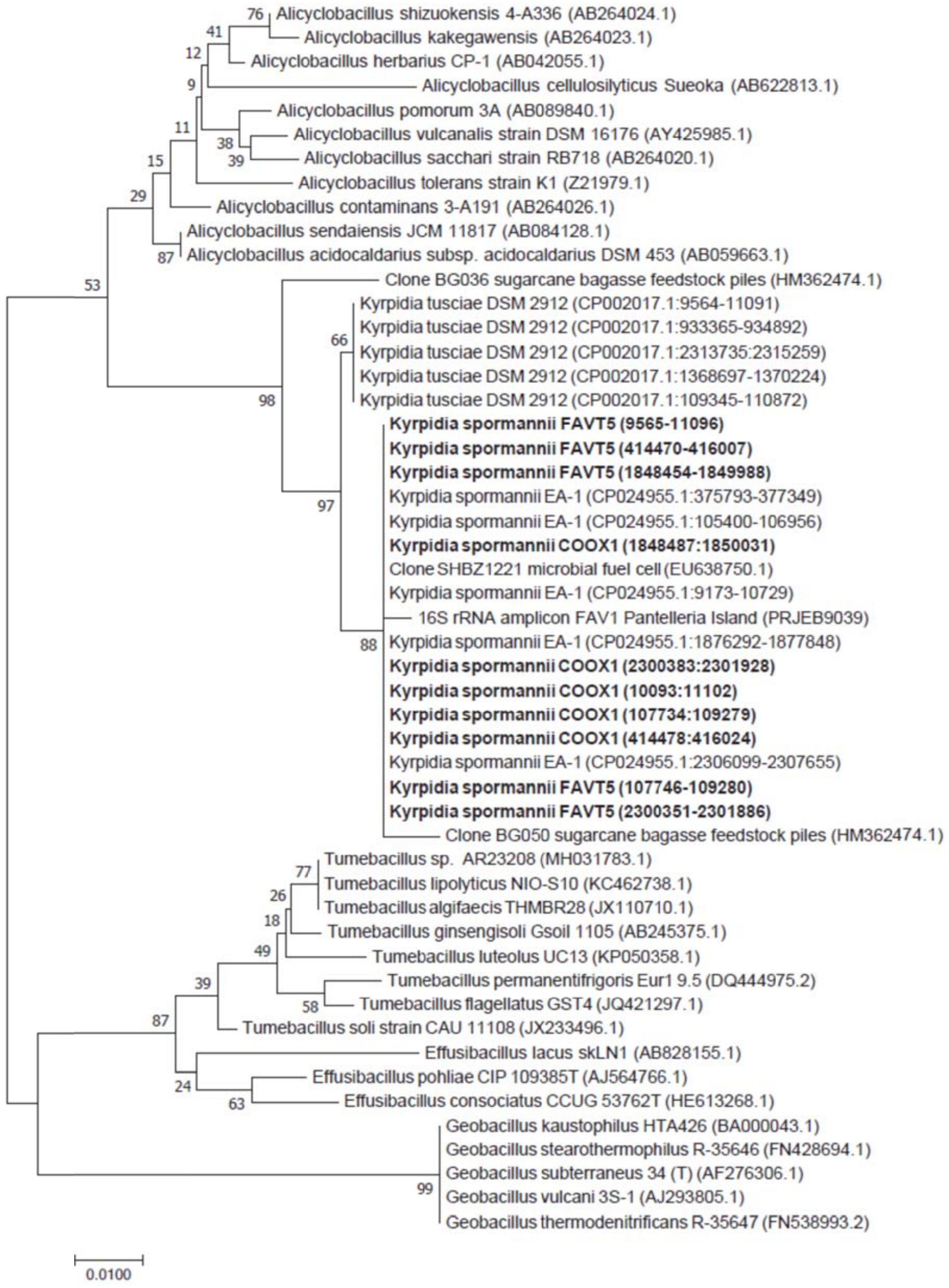

Figure S1: Phylogenetic tree of *Kyrpidia spormannii* strain FAVT5, *Kyrpidia spormannii* strain COOX1 and its relatives based on the 16S rRNA gene sequences. The tree was constructed using the Maximum Likelihood method based on the Tamura-Nei model (Tamura and Nei, 1993). Bootstrap percentage values (1000 replicates) are given at each node and the analysis was performed using MEGA7 (Kumar et al., 2016).

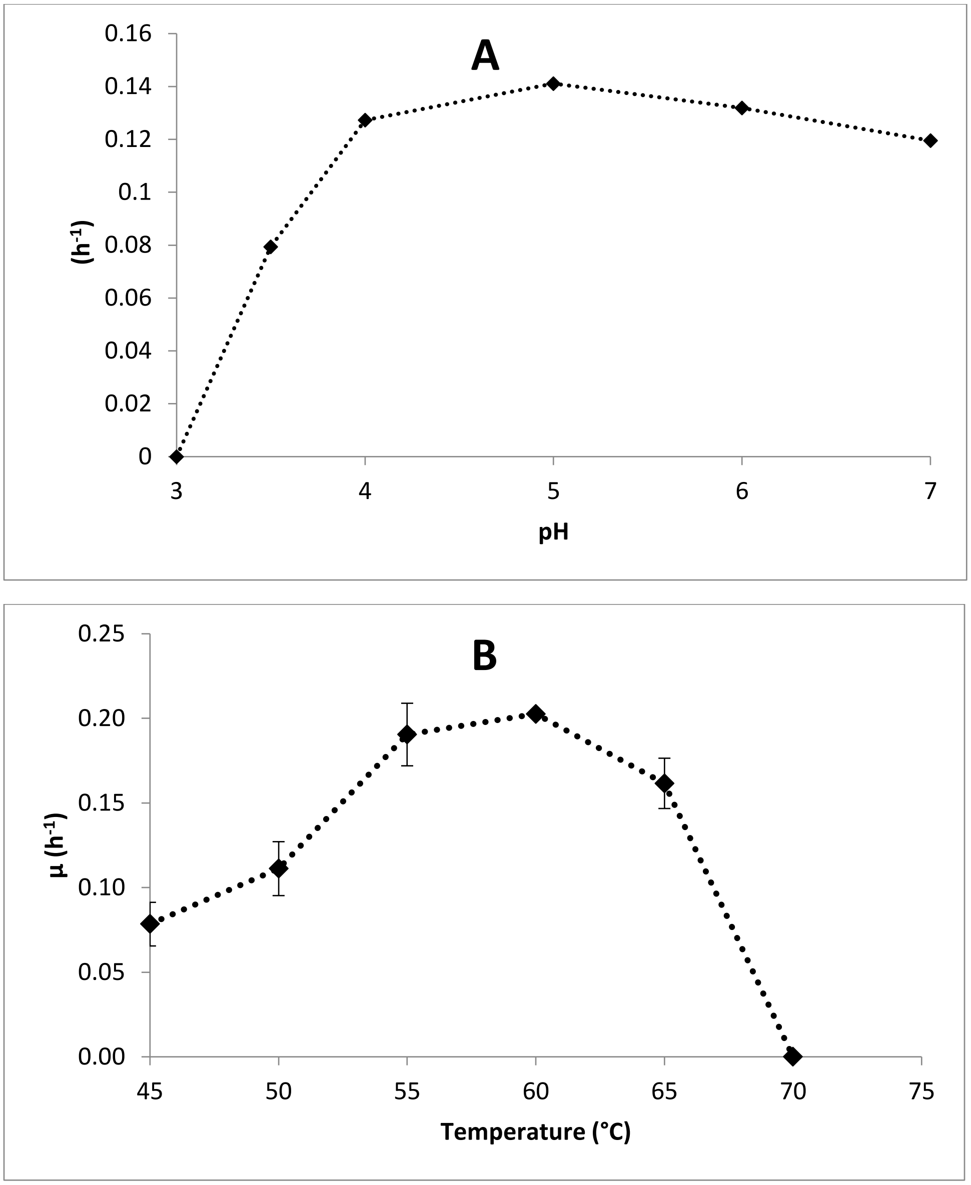

Figure S2. Growth rate of *Kyrpidia spormannii* FAVT5 at A. different pH at 50 °C and B. different temperature at pH 5.0.

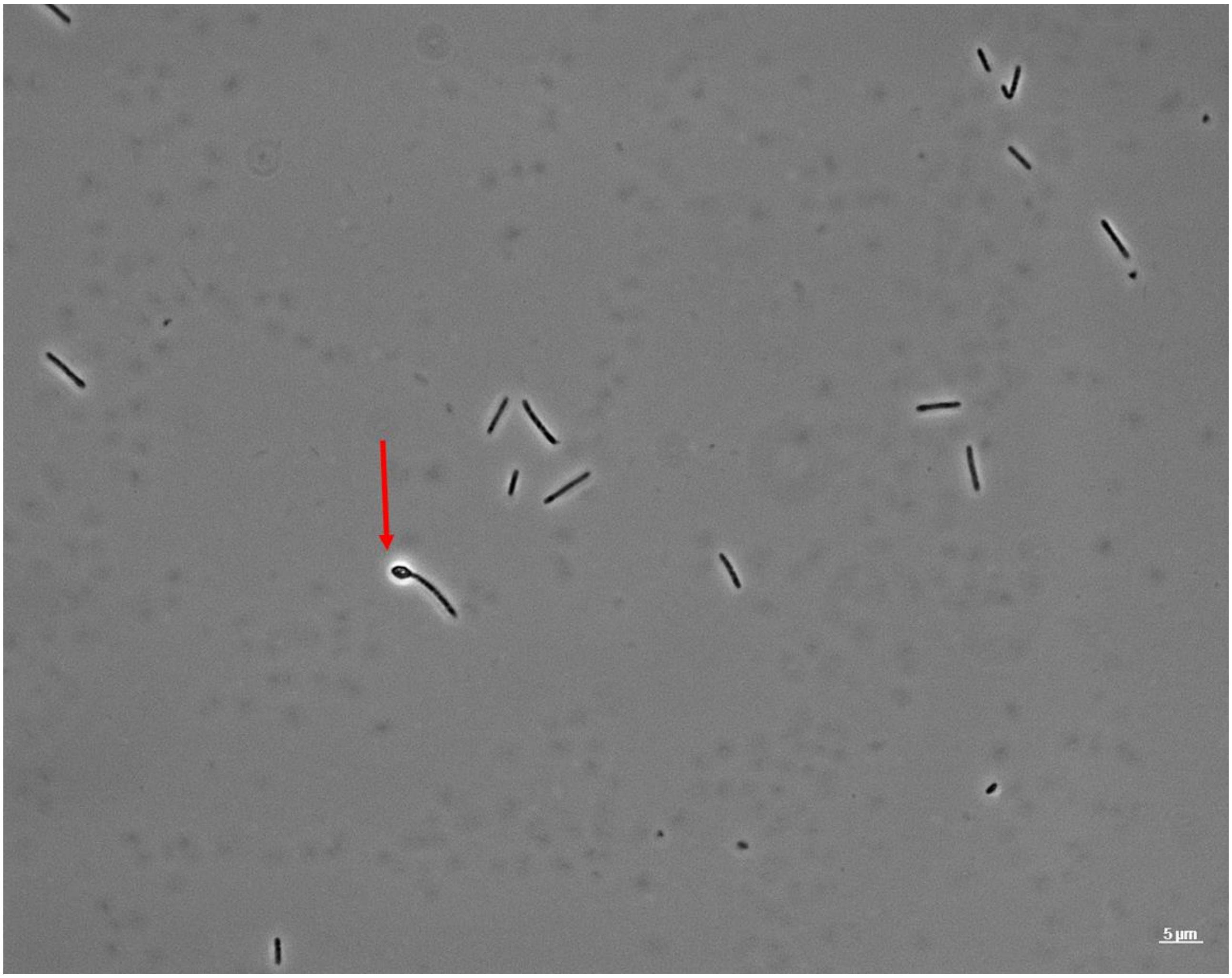

Figure S3: Phase contrast microscopy of a stationary phase spore-form forming cell of *Kyrpidia spormannii* FAVT5.

Table S1: Similarities of 16S rRNA genes between the strains *Kyrpidia spormannii* FAVT5, *Kyrpidia spormannii* COOX1, *Kyrpidia spormannii* EA-1 and *Kyrpidia tusciae*.

		1a	1b	1c	1d	1e	2a*	2b	2c	2d	2e
Kyrpidia spormannii FAVT5	1a										
	1b	99.6									
	1c	99.5	99.5								
	1d	99.3	99.5	99.4							
	1e	99.5	99.8	99.6	99.6						
Kyrpidia spormannii COOX1	2a*	100	100	99.54	99.8	99.9					
	2b	99.6	99.9	99.6	99.6	99.9	100.0				
	2c	99.6	99.6	99.9	99.4	99.7	99.9	99.7			
	2d	98.7	99.0	98.8	99.4	99.0	99.3	99.0	98.8		
	2e	99.6	99.8	99.6	99.6	100	99.9	99.87	99.7	99.0	
Kyrpidia spormannii EA-1	3a	99.6	99.7	99.7	99.5	99.6	99.9	99.81	99.74	99.0	99.8
	3b	99.6	99.9	99.7	99.7	99.9	100.0	99.94	99.81	99.1	99.9
	3c	99.6	99.7	99.7	99.5	99.8	99.9	99.81	99.71	99.0	99.8
	3d	99.6	99.7	99.7	99.5	99.8	99.9	99.81	99.74	99.0	99.8
	3e	99.7	99.8	99.7	99.6	99.9	100.0	99.87	99.81	99.0	99.9
Kyrpidia tusciae DSM 2912	3a	97.5	97.8	97.6	97.7	97.9	98.0	97.85	97.66	97.1	97.9
	3b	97.5	97.7	97.5	97.6	97.9	97.7	97.79	67.46	96.9	97.7
	3c	97.3	97.6	97.4	97.5	97.7	97.9	97.66	97.59	97.0	97.9
	3d	97.5	97.7	97.5	97.6	97.9	98.0	97.79	97.59	97.0	97.9
	3e	97.2	97.5	97.3	97.3	97.6	97.7	97.53	97.33	96.8	97.6

* partial 16S rRNA sequence

Table S2: Average nucleotide identity (ANIb) results showing the similarities between the strains *Kyrpidia spormannii* FAVT5, *Kyrpidia spormannii* COOX1, *Kyrpidia spormannii* EA-1 and *Kyrpidia tusciae*. Values are obtained using JSpeciesWS with standard settings.

		% similarity with strain based on ANI			
Strain no.	Strain name	1	2	3	4
1	*Kyrpidia spormannii* FAVT5		98.4	97.5	93.6
2	*Kyrpidia spormannii* COOX1	99.7		97.5	93.6
3	*Kyrpidia spormannii* EA-1	97.4	97.4		93.1
4	*Kyrpidia tusciae* DSM 2912	93.4	93.5	93.1	

Table S3: Genome annotation overview *Kyrpidia spormannii* FAVT5.

Gene	Product	EC no.	CDS[a]	Best BLAST hit in SwissProt[b]	Best BLAST hit in TrEMBL[c]
**Hydrogen metabolism**					
**Gene**	**Product**	**EC no.**	**CDS[a]**	**Best BLAST hit in SwissProt[b]**	**Best BLAST hit in TrEMBL[c]**
hucS_1	Hydrogenase small subunit	1.12.7.2	KFAV_v1_1431	Desulfomicrobium baculatum DSM 1743 (P13063, 29.45%)	Kyrpidia sp. EA-1 (A0A2K8NBH0, 100%)
hucL_1	Hydrogenase large subunit	1.12.99.6	KFAV_v1_1432	Desulfomicrobium baculatum (P13065, 29.76%)	Kyrpidia sp. EA-1 (A0A2K8N5M9, 100%)
	Putative hydrogenase maturation protease, HupD		KFAV_v1_1433	Bradyrhizobium diazoefficiens JCM 10833 / IAM 13628 / NBRC 14792 / USDA 110 (Q45251, 34.25%)	Kyrpidia sp. EA-1 (A0A2K8N7UO, 100%)
hypC	Hydrogenase maturation factor, HypC		KFAV_v1_1436	Azotobacter chroococcum mcd 1 (Q43951, 50.75%)	Kyrpidia sp. EA-1 (A0A2K8N8A3, 97.67%)
hypD	Hydrogenase maturation factor, HypD		KFAV_v1_1437	Bradyrhizobium diazoefficiens JCM 10833 / IAM 13628 / NBRC 14792 / USDA 110 (P31904, 51.08%)	Kyrpidia sp. EA-1 (A0A2K8N5T3, 99.19%)
hypE	Hydrogenase expression/formation protein, HypE		KFAV_v1_1438	Azotobacter chroococcum mcd 1 (P42034, 52.11%)	Kyrpidia sp. EA-1 (A0A2K8N5P9, 99.71%)
hypA	Hydrogenase maturation factor, HypA		KFAV_v1_1439	Desulfococcus oleovorans DSM 6200 / Hxd3 (A8ZZG8, 36.84%)	Kyrpidia sp. EA-1 (A0A2K8N6F6, 100%)
hypB	Hydrogenase maturation factor, HypB		KFAV_v1_1440	Azotobacter chroococcum mcd 1 (Q43949, 50%)	Kyrpidia sp. EA-1 (A0A2K8NAV7, 100%)
hypF	Carbamoyltransferase, HypF	6.2.-.-	KFAV_v1_1441	Cupriavidus necator ATCC 17699 / H16 / DSM 428 / Stanier 337 (O07451, 39.53%)	Kyrpidia sp. EA-1 (A0A2K8N5R1, 98.16%)
nixA	High-affinity nickel transport protein, nixA		KFAV_v1_1442	Helicobacter pylori J99 / ATCC 700824 (Q9ZM74, 49.54%)	Kyrpidia sp. EA-1 (A0A2K8NCR3, 98.56%)
hypF	Carbamoyltransferase, HypF	6.2.-.-	KFAV_v1_2731	Cupriavidus necator ATCC 17699 / H16 / DSM 428 / Stanier 337 (O07451, 42.09%)	Kyrpidia tusciae DSM 2912 / NBRC 15312
hypE	Hydrogenase expression/formation protein, HypE		KFAV_v1_2734	Azotobacter vinelandii ATCC 13705 / OP1 / DSM 366 / NCIB 11614 / LMG 3878 / UW (P40595, 49.7%)	Kyrpidia tusciae DSM 2912 / NBRC 15312
nixA	High-affinity nickel transport protein, nixA		KFAV_v1_2735	Helicobacter pylori J99 / ATCC 700824 (Q9ZM74, 51.69%)	Thermus thermophilus SG0.5JP17-16 (F6[illegible]
hypD	Hydrogenase maturation factor, HypD		KFAV_v1_2736	Bradyrhizobium diazoefficiens JCM 10833 / IAM 13628 / NBRC 14792 / USDA 110 (P31904, 56.03%)	Kyrpidia tusciae DSM 2912 / NBRC 15312
	Hydrogenase maturation factor		KFAV_v1_2737	Cupriavidus necator ATCC 17699 / H16 / DSM 428 / Stanier 337 (P31900, 29.41%)	Kyrpidia tusciae DSM 2912 / NBRC 15312
hypC	Hydrogenase maturation factor, HypC		KFAV_v1_2738	Azotobacter vinelandii ATCC 13705 / OP1 / DSM 366 / NCIB 11614 / LMG 3878 / UW (P31881, 48.24%)	Kyrpidia tusciae DSM 2912 / NBRC 15312
hcaB	NHL repeat protein, HcaB		KFAV_v1_2739	no hits	Kyrpidia tusciae DSM 2912 / NBRC 15312
hucE	Fe-S cluster biogenesis protein		KFAV_v1_2740	no hits	Kyrpidia tusciae DSM 2912 / NBRC 15312
hcaB	Tetratricopeptide repeat protein		KFAV_v1_2742	no hits	Kyrpidia tusciae DSM 2912 / NBRC 15312
	Hydrogenase maturation protease		KFAV_v1_2743	no hits	Kyrpidia tusciae DSM 2912 / NBRC 15312
	Hydrogenase maturation protease		KFAV_v1_2744	Escherichia coli K12 / TG1 / K12 / MG1655 / ATCC 47076 / K12 / W3110 / ATCC 27325 / DSM 5911 (P37182, 33.78%)	Kyrpidia tusciae DSM 2912 / NBRC 15312
hucL_2	Hydrogenase large subunit	1.12.99.6	KFAV_v1_2745	Desulfomicrobium baculatum (P13065, 31.44%)	Kyrpidia tusciae DSM 2912 / NBRC 15312
hucS_2	Hydrogenase small subunit	1.12.7.2	KFAV_v1_2746	Desulfovibrio gigas (P12943, 32.84%)	Kyrpidia tusciae DSM 2912 / NBRC 15312
hypB	Hydrogenase maturation factor, HypB		KFAV_v1_2747	Synechocystis sp. PCC 6803 / Kazusa (P74218, 51.44%)	Kyrpidia tusciae DSM 2912 / NBRC 15312
hypA	Hydrogenase maturation factor, HypA		KFAV_v1_2748	Chloroflexus aggregans MD-66 / DSM 9485 (B8G3M6, 47.32%)	Geobacillus galactosidasius (A0A226QL8[illegible]
**CO metabolism**					
**Gene**	**Product**	**EC no.**	**CDS[a]**	**Best BLAST hit in SwissProt[b]**	**Best BLAST hit in TrEMBL[c]**
coxS	Carbon monoxide dehydrogenase small chain	1.2.5.3	KFAV_v1_2877	Hydrogenophaga pseudoflava (P19915, 62.33%)	Kyrpidia sp. EA-1 (A0A2K8NBC5, 100%)
coxM	Carbon monoxide dehydrogenase medium chain	1.2.5.3	KFAV_v1_2875	Hydrogenophaga pseudoflava (P19914, 40.86%)	Kyrpidia sp. EA-1 (A0A2K8NAW8, 100%)
coxL	Carbon monoxide dehydrogenase large chain	1.2.5.3	KFAV_v1_2876	Hydrogenophaga pseudoflava (P19913, 43.22%)	Kyrpidia sp. EA-1 (A0A2K8N8V7, 100%)
coxG	Putative carbon monoxide dehydrogenase subunit G		KFAV_v1_2878	no hits	Kyrpidia sp. EA-1 (A0A2K8N8Z6, 97.97%)
coxL	Carbon monoxide dehydrogenase large chain	1.2.5.3	KFAV_v1_2838	Oligotropha carboxidovorans ATCC 49405 / DSM 1227 / KCTC 32145 / OM5 (P19919, 35.92%)	Kyrpidia sp. EA-1 (A0A2K8N9N0, 99.36%)
coxS	Carbon monoxide dehydrogenase small chain	1.2.5.3	KFAV_v1_2839	Hydrogenophaga pseudoflava (P19915, 52.35%)	Kyrpidia sp. EA-1 (A0A2K8N847, 100%)
coxM	Carbon monoxide dehydrogenase medium chain	1.2.5.3	KFAV_v1_2540	no hits	Kyrpidia sp. EA-1 (A0A2K8N831, 98.89%)
coxE	Putative CO dehydrogenase accessory protein		KFAV_v1_2541	no hits	Kyrpidia sp. EA-1 (A0A2K8NA63, 99.55%)
**Carbon fixation**					
	**Calvin cycle**				
cbbL	Ribulose bisphosphate carboxylase large chain	4.1.1.39	KFAV_v1_3127	Methylacidiphilum infernorum V4 (B3DVG5, 79.24%)	Kyrpidia sp. EA-1 (A0A2K8N9E7, 99.58%)
cbbS	Ribulose bisphosphate carboxylase small subunit	4.1.1.39	KFAV_v1_3128	Guillardia theta (P14960, 57.25%)	Kyrpidia tusciae DSM 2912 / NBRC 15312
cbbX	RuBisCO accessory protein		KFAV_v1_3129	Cyanidioschyzon merolae 10D (O22025, 68.31%)	Kyrpidia sp. EA-1 (A0A2K8N9D1, 99.33%)
pgk	phosphoglycerate kinase	2.7.2.3	KFAV_v1_3121	Carboxydothermus hydrogenoformans ATCC BAA-161 / DSM 6008 / Z-2901 (Q3AFD1, 62.24%)	Kyrpidia sp. EA-1 (A0A2K8N9I4, 98.73%)
gapA	glyceraldehyde-3-phosphate dehydrogenase	1.2.1.12	KFAV_v1_3125	Geobacillus stearothermophilus (P00362, 59.39%)	Kyrpidia sp. EA-1 (A0A2K8NAX0, 99.13%)
fbaA	fructose-1,6-bisphosphate aldolase	4.1.2.13	KFAV_v1_3123	Thermus caldophilus (Q70312, 48.83%)	Kyrpidia sp. EA-1 (A0A2K8NA10, 98.95%)
glpX	fructose 1,6-bisphosphatase class II	3.1.3.11	KFAV_v1_3126	Bacillus subtilis 168 / BD99 / MS119 / 168 / 168 / 168 / Marburg / ATCC 6051 / DSM 10 / JCM 1465 / NBRC 13719 / NCIMB 3610 / VKM B-501 (Q03224, 60.95%)	Kyrpidia sp. EA-1 (A0A2K8N9D2, 98.6%)
tkt	transketolase	2.2.1.1	KFAV_v1_3131	Geobacillus stearothermophilus DSM 13240 (A0A0I9QGZ2, 62.03%)	Kyrpidia sp. EA-1 (A0A2K8N9J4, 96.74%)
rpiB	D-ribose 5-phosphate isomerase	5.3.1.6	KFAV_v1_3133	Escherichia coli K12 / TG1 / K12 / MG1655 / ATCC 47076 / K12 / W3110 / ATCC 27325 / DSM 5911 (P37351, 50.68%)	Kyrpidia sp. EA-1 (A0A2K8N9H0, 98.7%)
prk	phosphoribulokinase	2.7.1.19	KFAV_v1_3124	Synechocystis sp. PCC 6803 / Kazusa (P37101, 36.65%)	Kyrpidia sp. EA-1 (A0A2K8N9C1, 98.71%)
pgk	phosphoglycerate kinase	2.7.2.3	KFAV_v1_0607	Brevibacillus brevis 47 / JCM 6285 / NBRC 100599 (COZ6L, 62.28%)	Kyrpidia sp. EA-1 (A0A2K8N5B1, 99.75%)
gapA	glyceraldehyde-3-phosphate dehydrogenase	1.2.1.12	KFAV_v1_0606	Geobacillus stearothermophilus (P00362, 72.46%)	Kyrpidia sp. EA-1 (A0A2K8NAP9, 99.42%)
tpi	triosephosphate isomerase	5.3.1.1	KFAV_v1_0608	Symbiobacterium thermophilum T / IAM 14863 (Q67SW4, 55.12%)	Kyrpidia sp. EA-1 (A0A2K8N3B4, 96.27%)
rpe	ribulose-5-phosphate 3-epimerase	5.1.3.1	KFAV_v1_1991	Bacillus subtilis 168 (O34557, 59.43%)	Kyrpidia sp. EA-1 (A0A2K8N8Z8, 100%)
fbaA	fructose-1,6-bisphosphate aldolase	4.1.2.13	KFAV_v1_3548	Geobacillus stearothermophilus (P94453, 57.89%)	Kyrpidia sp. EA-1 (A0A2K8NAB0, 99.65%)
glpX	fructose 1,6-bisphosphatase class II	3.1.3.11	KFAV_v1_3542	Bacillus subtilis 168 / BD99 / MS119 / 168 / 168 / 168 / Marburg / ATCC 6051 / DSM 10 / JCM 1465 / NBRC 13719 / NCIMB 3610 / VKM B-501 (Q03224, 67.08%)	Kyrpidia sp. EA-1 (A0A2K8NCR0, 99.69%)
tkt	fragment of transketolase (part 2)	2.2.1.1	KFAV_v1_2895	Geobacillus stearothermophilus DSM 13240 (A0A0I9QGZ2, 50.52%)	Kyrpidia sp. EA-1 (A0A2K8N8X8, 100%)
tkt	fragment of transketolase (part 1)	2.2.1.1	KFAV_v1_2896	Geobacillus stearothermophilus DSM 13240 (A0A0I9QGZ2, 65.11%)	Kyrpidia sp. EA-1 (A0A2K8N8X3, 98.53%)
rpiB	D-ribose 5-phosphate isomerase	5.3.1.6	KFAV_v1_3818	Listeria innocua serovar 6a ATCC BAA-680 / CLIP 11262 (Q92EU5, 47.95%)	Kyrpidia tusciae DSM 2912 / NBRC 15312
**Carbon metabolism**					
	**Alcohol oxidation**				
**Gene**	**Product**	**EC no.**	**CDS[a]**	**Best BLAST hit in SwissProt[b]**	**Best BLAST hit in TrEMBL[c]**
adh	alcohol dehydrogenase	1.1.1.1	KFAV_v1_0360	Cupriavidus necator ATCC 17699 / H16 / DSM 428 / Stanier 337 (Q0KDL6, 38.32%)	Kyrpidia sp. EA-1 (A0A2K8N2R4, 99.71%)
adh	alcohol dehydrogenase	1.1.1.1	KFAV_v1_0757	no hits	Kyrpidia tusciae DSM 2912 / NBRC 15312
adh	alcohol dehydrogenase	1.1.1.1	KFAV_v1_1314	Geobacillus stearothermophilus (P42327, 58.28%)	Kyrpidia sp. EA-1 (A0A2K8N5H2, 99.7%)
adh	alcohol dehydrogenase	1.1.1.1	KFAV_v1_1858	Geobacillus stearothermophilus (P12311, 38.55%)	Kyrpidia sp. EA-1 (A0A2K8N6J4, 99.72%)
adh	alcohol dehydrogenase	1.1.1.1	KFAV_v1_2105	Pseudomonas putida G1 / ATCC 17453 / G1 / ATCC 17453 (P09347, 33.91%)	Kyrpidia sp. EA-1 (A0A2K8N6J4, 99.72%)
adh	alcohol dehydrogenase	1.1.1.1	KFAV_v1_2351	Bacillus subtilis 168 (O06012, 38.89%)	Kyrpidia sp. EA-1 (A0A2K8NAB4, 98.27%)
	putative alcohol dehydrogenase		KFAV_v1_1321	Staphylococcus aureus MRSA252 (Q6GEP3, 39.18%)	Kyrpidia tusciae DSM 2912 / NBRC 15312
	putative alcohol dehydrogenase		KFAV_v1_2460	Escherichia coli K12 / TG1 / K12 / MG1655 / ATCC 47076 / K12 / W3110 / ATCC 27325 / DSM 5911 (P7731066, 34.43.68%)	Pseudonocardia dioxanivorans ATCC 554[illegible]
mhpF	acetaldehyde dehydrogenase II	1.2.1.10	KFAV_v1_2446	Mycobacterium gilvum PYR-GCK (A4T7U3, 65.32%)	Kyrpidia tusciae DSM 2912 / NBRC 15312
aldE	acetaldehyde dehydrogenase	1.1.1.1, 1.2	KFAV_v1_2839	Escherichia coli O157:H7 / EDL933 / ATCC 700927 / EHEC (P0A9Q8, 49.54%)	Kyrpidia sp. EA-1 (A0A2K8NA97, 96.22%)
	**Glycolysis / Gluconeogenesis /**				
pgcA	phosphoglucomutase	5.4.2.2	KFAV_v1_0460	Bacillus subtilis 168 (P18159, 46.99%)	Kyrpidia sp. EA-1 (A0A2K8NAK3, 99.65%)
pgi	glucose-6-phosphate isomerase	5.3.1.9	KFAV_v1_1500	Geobacillus stearothermophilus T521 (P13376, 43.32%)	Kyrpidia sp. EA-1 (A0A2K8NAU6, 97.73%)
pfkA	6-phosphofructokinase	2.7.1.11	KFAV_v1_2636	Geobacillus kaustophilus HTA426 (Q5KWB1, 65.2%)	Kyrpidia sp. EA-1 (A0A2K8NAD6 , 99.693[illegible]
glpX	fructose 1,6-bisphosphatase class II	3.1.3.11	KFAV_v1_3126	Bacillus subtilis 168 / BD99 / MS119 / 168 / 168 / 168 / Marburg / ATCC 6051 / DSM 10 / JCM 1465 / NBRC 13719 / NCIMB 3610 / VKM B-501 (Q03224, 60.95%)	Kyrpidia sp. EA-1 (A0A2K8N9D2, 98.6%)
glpX	fructose 1,6-bisphosphatase class II	3.1.3.11	KFAV_v1_3542	Bacillus subtilis 168 / BD99 / MS119 / 168 / 168 / 168 / Marburg / ATCC 6051 / DSM 10 / JCM 1465 / NBRC 13719 / NCIMB 3610 / VKM B-501 (Q03224, 67.08%)	Kyrpidia sp. EA-1 (A0A2K8NCR0, 99.69%)
fbaA	fructose-1,6-bisphosphate aldolase	4.1.2.13	KFAV_v1_3548	Geobacillus stearothermophilus (P94453, 57.89%)	Kyrpidia sp. EA-1 (A0A2K8NAB0, 99.65%)

tpi	triosephosphate isomerase	5.3.1.1	KFAV_v1_0608	Symbiobacterium thermophilum T / IAM 14863 (Q67SW4, 55.12%)	Kyrpidia sp. EA-1 (A0A2K8N3B4, 96.27%)
gapA	glyceraldehyde-3-phosphate dehydrogenase	1.2.1.12	KFAV_v1_3125	Geobacillus stearothermophilus (P00362, 59.39%)	Kyrpidia sp. EA-1 (A0A2K8NAX0, 99.13%)
gapA	glyceraldehyde-3-phosphate dehydrogenase	1.2.1.12	KFAV_v1_0606	Geobacillus stearothermophilus (P00362, 72.46%)	Kyrpidia sp. EA-1 (A0A2K8NAP9, 99.42%)
pgk	phosphoglycerate kinase	2.7.2.3	KFAV_v1_0607	Brevibacillus brevis 47 / JCM 6285 / NBRC 100599 (COZ6L, 62.28%)	Kyrpidia sp. EA-1 (A0A2K8N5B1, 99.75%)
pgm	phosphoglycerate mutase	5.4.2.12	KFAV_v1_0609	Geobacillus kaustophilus HTA426 (Q5KVE6, 65.34%)	Kyrpidia sp. EA-1 (A0A2K8N3B9, 99.8%)
	**Glycon storage**				
ykcC	putative glycosyltransferase	2.4.-.-	KFAV_v1_0322	Bacillus subtilis 168 (O34319, 56.95%)	Geobacillus thermoleovorans (A0A1C3D9
	putative glycosyltransferase		KFAV_v1_0392	Stackebrandtia nassauensis DSM 44728 / NRRL B-16338 / NBRC 102104 / LLR-40K-21 (D3Q051, 26.49%)	Deinococcus hopiensis KR-140 (A0A1W1U
	putative glycosyltransferase		KFAV_v1_0393	Haloferax volcanii ATCC 29605 / DSM 3757 / JCM 8879 / NBRC 14742 / NCIMB 2012 / VKM B-1768 / DS2 (D4GYG7, 23.71%)	Thermus parvatiensis RL (H7GFQ3, 50.55
	putative glycosyltransferase	2.4.-.-	KFAV_v1_0413	Bacillus subtilis 168 (P71053, 28.28%)	Kyrpidia sp. EA-1 (A0A2K8NB68, 92.88%)
	putative glycosyltransferase	2.4.-.-	KFAV_v1_1173	No hits	Kyrpidia sp. EA-1 (A0A2K8N6Z6, 99.45%)
	putative glycosyltransferase	2.4.-.-	KFAV_v1_1943	No hits	Kyrpidia sp. EA-1 (A0A2K8N6R4, 99.45%)
	**TCA cycle**				
citZ	citrate synthase II	2.3.3.16	KFAV_v1_2570	Haloferax volcanii ATCC 29605 / DSM 3757 / JCM 8879 / NBRC 14742 / NCIMB 2012 / VKM B-1768 / DS2 (D4GS06, 56.68%)	Kyrpidia sp. EA-1 (A0A2K8N851, 99.73%)
citA	citrate synthase I	2.3.3.16	KFAV_v1_2602	Bacillus subtilis 168 (P39119, 57.5%)	Kyrpidia sp. EA-1 (A0A2K8NAT2, 100%)
citB	aconitate hydratase	4.2.1.3	KFAV_v1_2761	Bacillus subtilis 168 (P09339, 63.25%)	Kyrpidia sp. EA-1 (A0A2K8N990, 99.67%)
icd	isocitrate dehydrogenase	1.1.1.42	KFAV_v1_2634	Bacillus subtilis 168 (P39126, 75.95%)	Kyrpidia sp. EA-1 (A0A2K8N8B9, 99.77%)
icd	putative isocitrate dehydrogenase, E1 subunit	1.1.1.41	KFAV_v1_2765	Thermus thermophilus HB8 / ATCC 27634 / DSM 579 (P33197, 30%)	Kyrpidia sp. EA-1 (A0A2K8N8K6, 99.45%)
odhA	2-oxoglurarate dehydrogenase	1.2.4.2	KFAV_v1_2024	Geobacillus kaustophilus HTA426 (Q5L172, 54.64%)	Kyrpidia sp. EA-1 (A0A2K8N6Y9, 99.15%)
odhB	2-oxoglutarate dehydrogenase, E2 subunit	2.3.1.61	KFAV_v1_2025	Bacillus subtilis 168 (P16263, 52.06%)	Kyrpidia sp. EA-1 (A0A2K8N926, 98.85%)
sucD	succinyl-CoA synthetase (alpha subunit)	6.2.1.4	KFAV_v1_1927	Bacillus subtilis 168 (P80865, 79.93%)	Kyrpidia sp. EA-1 (A0A2K8NB08, 99.67%)
sucC	succinyl-CoA synthetase (beta subunit)	6.2.1.4	KFAV_v1_1928	Geobacillus thermodenitrificans NG80-2 (A4IM83, 77.92%)	Kyrpidia sp. EA-1 (A0A2K8N6T1, 99.74%)
sdhA	succinate dehydrogenase (ubiquinone) flavoprotein subunit	1.3.5.1	KFAV_v1_1119	Bacillus subtilis 168 (P08065, 75.81%)	Kyrpidia sp. EA-1 (A0A2K8N4L4, 99.83%)
sdhC	succinate dehydrogenase (cytochrome b558 subunit)	1.3.5.1	KFAV_v1_1118	Bacillus subtilis 168 (P08064, 52.19%)	Kyrpidia sp. EA-1 (A0A2K8N788, 99.02%)
sdhB	succinate dehydrogenase (iron-sulfur protein)	1.3.5.1	KFAV_v1_1120	Bacillus subtilis 168 (P08066, 77.92%)	Kyrpidia sp. EA-1 (A0A2K8N4K4, 98.8%)
fumA	fumarate hydratase, class I	4.2.1.2	KFAV_v1_2862	Geobacillus stearothermophilus (Q04718, 70.18%)	Kyrpidia sp. EA-1 (A0A2K8N8U0, 99.61%)
mdh	malate dehydrogenase (NAD-dependent)	1.1.1.37	KFAV_v1_2633	Anoxybacillus flavithermus DSM 21510 / WK1 (B7GGT8, 79.29%)	Kyrpidia sp. EA-1 (A0A2K8N9V6, 100%)
mdh	malate dehydrogenase (NAD-dependent)	1.1.1.37	KFAV_v1_2833	Desulforudis audaxviator MP104C (B1I3T1, 60.73%)	Kyrpidia sp. EA-1 (A0A2K8NB92 , 68.05%)
	**Glyoxylate cycle**				
aceB	malate synthase A	2.3.3.9	KFAV_v1_2384	Myxococcus xanthus DK 1622 (P95329, 60.3%)	Kyrpidia sp. EA-1 (A0A2K8N7T4, 100%)
aceB	malate synthase A	2.3.3.9	KFAV_v1_2385	Myxococcus xanthus DK 1622 (P95329, 63.71%)	Kyrpidia tusciae DSM 2912 / NBRC 15312
mdh	malate dehydrogenase (NAD-dependent)	1.1.1.37	KFAV_v1_2633	Anoxybacillus flavithermus DSM 21510 / WK1 (B7GGT8, 79.29%)	Kyrpidia sp. EA-1 (A0A2K8N9V6, 100%)
mdh	malate dehydrogenase (NAD-dependent)	1.1.1.37	KFAV_v1_2833	Desulforudis audaxviator MP104C (B1I3T1, 60.73%)	Kyrpidia sp. EA-1 (A0A2K8NB92 , 68.05%)
citB	aconitate hydratase	4.2.1.3	KFAV_v1_2761	Bacillus subtilis 168 (P09339, 63.25%)	Kyrpidia sp. EA-1 (A0A2K8N990, 99.67%)
aceA	isocitrate lyase	4.1.3.1	KFAV_v1_2386	Bacillus halodurans ATCC BAA-125 / DSM 18197 / FERM 7344 / JCM 9153 / C-125 (Q9K9H0 , 77.2%)	Kyrpidia sp. EA-1 (A0A2K8N7U7, 99.76%)
	**Pentose phosphate pathway (non oxidative part)**				
rpiB	D-ribose 5-phosphate isomerase	5.3.1.6	KFAV_v1_3133	Escherichia coli K12 / TG1 / K12 / MG1655 / ATCC 47076 / K12 / W3110 / ATCC 27325 / DSM 5911 (P37351, 50.68%)	Kyrpidia sp. EA-1 (A0A2K8N9H0, 98.7%)
rpiB	D-ribose 5-phosphate isomerase	5.3.1.6	KFAV_v1_3518	Bacillus subtilis 168 (P39156, 60.54%)	Kyrpidia tusciae DSM 2912 / NBRC 15312
tkt	transketolase	2.2.1.1	KFAV_v1_3131	Geobacillus stearothermophilus DSM 13240 (A0A0I9QGZ2, 62.03%)	Kyrpidia sp. EA-1 (A0A2K8N9J4, 96.74%)
tal	transaldolase	2.2.1.2	KFAV_v1_3547	Brevibacillus brevis 47 / JCM 6285 / NBRC 100599 (C0Z838, 68.72%)	Kyrpidia sp. EA-1 (A0A2K8NAY1, 98.14%)
	**Acetate metabolims**				
actP	Cation acetate symporter		KFAV_v1_1055	Klebsiella pneumoniae 342 (B5XXT7, 50.29%)	Kyrpidia sp. EA-1 (A0A2K8N5B5, 99%)
actP	Cation acetate symporter		KFAV_v1_1109	Yersinia pseudotuberculosis serotype O:1b IP 31758 (A7FNG3, 43.15%)	Kyrpidia sp. EA-1 (A0A2K8N4J1, 99.03%)
actP	Cation acetate symporter		KFAV_v1_2440	Escherichia coli O81 ED1a (B7MSH6, 47.66%)	Kyrpidia sp. EA-1 (A0A2K8N5B5, 66.87%)
acs	acetyl-CoA synthetase	6.2.1.1	KFAV_v1_0685	Bacillus subtilis 168 (P39062, 63.88%)	Kyrpidia sp. EA-1 (A0A2K8N3K9,99.65%)
acs	acetyl-CoA synthetase	6.2.1.1	KFAV_v1_1065	Bacillus subtilis 168 (P39062, 71.6%)	Kyrpidia sp. EA-1 (A0A2K8N4E4,100%)
acs	acetyl-CoA synthetase	6.2.1.1	KFAV_v1_1113	Gemmatimonas aurantiaca T-27 / DSM 14586 / JCM 11422 / NBRC 100505 (C1AA44, 63.25%)	Kyrpidia sp. EA-1 (A0A2K8N684,99.38%)
acs	acetyl-CoA synthetase	6.2.1.1	KFAV_v1_1272	Thermus thermophilus HB8 / ATCC 27634 / DSM 579 (Q5SIW6, 50.08%)	Kyrpidia sp. EA-1 (A0A2K8N507,99.08%)
acs	acetyl-CoA synthetase	6.2.1.1	KFAV_v1_2679	Thermus thermophilus HB8 / ATCC 27634 / DSM 579 (Q5SIW6, 42.97%)	Kyrpidia sp. EA-1 (A0A2K8ND91,99.24%)
**Nitrogen metabolism**					
	**Nitrogen fixation**				
**Gene**	**Product**	**EC no.**	**CDS[a]**	**Best BLAST hit in SwissProt[b]**	**Best BLAST hit in TrEMBL[c]**
nifH	Nitrogenase iron protein	1.18.6.1	KFAV_v1_1746	Cyanothece sp. PCC 7425 / ATCC 29141 (B8HWE3, 76.82%)	Kyrpidia sp. EA-1 (A0A2K8N8F4, 100%)
nifD	Nitrogenase molybdenum-iron protein alpha chain	1.18.6.1	KFAV_v1_1747	Azotobacter vinelandii ATCC 13705 / OP1 / DSM 366 / NCIB 11614 / LMG 3878 / UW / ATCC 13705 / OP1 / DSM 366 / NCIB 11614 / LMG 3878 / UW / ATCC 13705 / OP1 / DSM 366 / NCIB 11614 / LMG 3878 / UW / CA (P07328, 67.15%)	Kyrpidia sp. EA-1 (A0A2K8N6A0, 98.96%)
nifK	Nitrogenase molybdenum-iron protein beta chain	1.18.6.1	KFAV_v1_1748	Nostoc sp. PCC 7120 / SAG 25.82 / UTEX 2576 (P00468, 58.25%)	Kyrpidia sp. EA-1 (A0A2K8N8X2 , 94.07%)
nifE	Nitrogenase iron-molybdenum cofactor biosynthesis protein		KFAV_v1_1749	Cyanothece sp. PCC 8801 (O07355, 56.43%)	Kyrpidia sp. EA-1 (A0A2K8N6G5 , 98.297
nifN	Putative nitrogenase iron-molybdenum cofactor biosynthesis protein		KFAV_v1_1750	Cyanothece sp. PCC 8801 (O07356, 40.49%)	Kyrpidia sp. EA-1 (A0A2K8N6C7, 98.97%)
nifW	Putative nitrogenase stabilizing/protective protein		KFAV_v1_1759	Nostoc sp. PCC 7120 / SAG 25.82 / UTEX 2576 (Q44149, 42.7%)	Kyrpidia sp. EA-1 (A0A2K8N7V5, 99.12%)
	**Denitrification**				
	Putative nitric-oxide reductase	1.7.2.5	KFAV_v1_0646	Pseudomonas aeruginosa ATCC 15692 / DSM 22644 / CIP 104116 / JCM 14847 / LMG 12228 / 1C / PRS 101 / PAO1 (Q59647, 32.69%)	Kyrpidia tusciae DSM 2912 / NBRC 15312
**Sulfur metabolism**					
	**Assimilatory sulfate reduction**				
**Gene**	**Product**	**EC no.**	**CDS[a]**	**Best BLAST hit in SwissProt[b]**	**Best BLAST hit in TrEMBL[c]**
cysH	Phosphoadenosine phosphosulfate reductase	1.8.4.8	KFAV_v1_0202	Geobacillus thermodenitrificans NG80-2 (A4IKB7, 54.07%)	Kyrpidia sp. EA-1 (A0A2K8N589, 98.73%)
cysI	Sulfite reductase	1.8.1.2	KFAV_v1_2243	Paenibacillus sp. JDR-2 (C6CXN6, 46.03%)	Kyrpidia tusciae DSM 2912 / NBRC 15312
**Respiration**					
	**Complex I NADH dehydrogenase**				
**Gene**	**Product**	**EC no.**	**CDS[a]**	**Best BLAST hit in SwissProt[b]**	**Best BLAST hit in TrEMBL[c]**
nuoM	NADH-quinone oxidoreductase subunit M		KFAV_v1_3132	no hits	Kyrpidia tusciae DSM 2912 / NBRC 15312
nuoA	NADH-quinone oxidoreductase subunit A	1.6.5.11	KFAV_v1_3134	Bacillus cytotoxicus DSM 22905 / CIP 110041 / 391-98 / NVH 391-98 (A7GV51, 54.55%)	Kyrpidia sp. EA-1 (A0A2K8NA20, 99.18%)
nuoB	NADH-quinone oxidoreductase subunit B	1.6.5.11	KFAV_v1_3135	Geobacillus kaustophilus HTA426 (Q5KUJ6, 78.79%)	Kyrpidia sp. EA-1 (A0A2K8N9D3, 98.87%)
nuoC	NADH-quinone oxidoreductase subunit C	1.6.5.11	KFAV_v1_3136	Roseiflexus castenholzii DSM 13941 / HLO8 (A7NIW0, 49.3%)	Kyrpidia tusciae DSM 2912 / NBRC 15312
nuoD	NADH-quinone oxidoreductase subunit D	1.6.5.11	KFAV_v1_3137	Bacillus thuringiensis subsp. konkukian 97-27 (Q6HAY8, 95.3%)	Kyrpidia sp. EA-1 (A0A2K8N9E1, 99.46%)
nuoH	NADH-quinone oxidoreductase subunit H	1.6.5.11	KFAV_v1_3138	Brevibacillus brevis 47 / JCM 6285 / NBRC 100599 (C0Z769, 60.62%)	Kyrpidia sp. EA-1 (A0A2K8N9F8, 99.4%)
nuoI	NADH-quinone oxidoreductase subunit I	1.6.5.11	KFAV_v1_3139	Solibacter usitatus Ellin6076 (Q01ZR8, 38.1%)	Kyrpidia sp. EA-1 (A0A2K8NBH4, 99.31%)
nouJ	NADH-quinone oxidoreductase subunit J	1.6.5.11	KFAV_v1_3140	no hits	Kyrpidia sp. EA-1 (A0A2K8NBC1, 98.01%)
nouK	NADH-quinone oxidoreductase subunit K	1.6.5.11	KFAV_v1_3141	Geobacillus sp. WCH70 (C5D981, 54.55%)	Kyrpidia sp. EA-1 (A0A2K8N9E4, 98.25%)
nouL	NADH-quinone oxidoreductase subunit L	1.6.5.11	KFAV_v1_3142	Thermus thermophilus HB8 / ATCC 27634 / DSM 579 (Q56227, 41.36%)	Kyrpidia sp. EA-1 (A0A2K8NC48, 99.05%)
nuoN	NADH-quinone oxidoreductase subunit N	1.6.5.11	KFAV_v1_3143	Anoxybacillus flavithermus DSM 21510 / WK1 (B7GME1, 40.28%)	Kyrpidia sp. EA-1 (A0A2K8N9K3, 99.39%)
nuoN	NADH-quinone oxidoreductase subunit N	1.6.5.11	KFAV_v1_3492	Brevibacillus brevis 47 / JCM 6285 / NBRC 100599 (C0Z763, 42.77%)	Kyrpidia sp. EA-1 (A0A2K8NA61, 99.8%)

nuoM	NADH-quinone oxidoreductase subunit M		KFAV_v1_3493	Pseudomonas aeruginosa ATCC 15692 / DSM 22644 / CIP 104116 / JCM 14847 / LMG 12228 / 1C / PRS 101 / PAO1 (Q9I0J0, 34.64%)	Kyrpidia sp. EA-1 (A0A2K8NCJ8, 99.21%)
nuoL	NADH-quinone oxidoreductase subunit L	1.6.5.11	KFAV_v1_3494	no hits	Kyrpidia sp. EA-1 (A0A2K8NA75, 99.21%)
nouK	NADH-quinone oxidoreductase subunit K	1.6.5.11	KFAV_v1_3495	Brevibacillus brevis 47 / JCM 6285 / NBRC 100599 (C0Z766, 69.39%)	Kyrpidia tusciae DSM 2912 / NBRC 15312
nouJ	NADH-quinone oxidoreductase subunit J	1.6.5.11	KFAV_v1_3496	no hits	Kyrpidia sp. EA-1 (A0A2K8NA99, 98.88%)
nuoI	NADH-quinone oxidoreductase subunit I	1.6.5.11	KFAV_v1_3497	Carboxydothermus hydrogenoformans ATCC BAA-161 / DSM 6008 / Z-2901 (Q3AC82, 42.2%)	Kyrpidia sp. EA-1 (A0A2K8NA95, 100%)
nuoH	NADH-quinone oxidoreductase subunit H	1.6.5.11	KFAV_v1_3498	Brevibacillus brevis 47 / JCM 6285 / NBRC 100599 (C0Z769, 62.42%)	Kyrpidia sp. EA-1 (A0A2K8NAU1, 99.4%)
nuoD	NADH-quinone oxidoreductase subunit D	1.6.5.3	KFAV_v1_3499	Geobacillus thermodenitrificans NG80-2 (A4ITI4, 71.07%)	Kyrpidia sp. EA-1 (A0A2K8NA62, 100%)
nuoC	NADH-quinone oxidoreductase subunit C	1.6.5.11	KFAV_v1_3500	Chlorobium phaeobacteroides DSM 266 (A1BF34, 37.41%)	Kyrpidia sp. EA-1 (A0A2K8NBR6, 98.52%)
nuoB	NADH-quinone oxidoreductase subunit B	1.6.5.11	KFAV_v1_3501	Bacillus weihenstephanensis KBAB4 (A9VS97, 77.3%)	Kyrpidia tusciae DSM 2912 / NBRC 15312
nuoA	NADH-quinone oxidoreductase subunit A	1.6.5.11	KFAV_v1_3502	Bacillus weihenstephanensis KBAB4 (A9VS98, 63.87%)	Kyrpidia sp. EA-1 (A0A2K8NA70, 99.17%)
	**Complex II succinate dehydrogenase**				
sdhA	succinate dehydrogenase (ubiquinone) flavoprotein subunit	1.3.5.1	KFAV_v1_1119	Bacillus subtilis 168 (P08065, 75.81%)	Kyrpidia sp. EA-1 (A0A2K8N4L4, 99.83%)
sdhC	succinate dehydrogenase (cytochrome b558 subunit)	1.3.5.1	KFAV_v1_1118	Bacillus subtilis 168 (P08064, 52.19%)	Kyrpidia sp. EA-1 (A0A2K8N788, 99.02%)
sdhB	succinate dehydrogenase (iron-sulfur protein)	1.3.5.1	KFAV_v1_1120	Bacillus subtilis 168 (P08066, 77.92%)	Kyrpidia sp. EA-1 (A0A2K8N4K4, 98.8%)
	**Cytochromes**				
	Putative cytochrome c oxidase subunit IIa		KFAV_v1_0030	no hits	no hits
cbaA	Cytochrome c oxidase subunit 1	1.9.3.1	KFAV_v1_0031	Thermus thermophilus VK1 (Q56408, 50.93%)	Kyrpidia sp. EA-1 (A0A2K8N1Y7, 99.64%)
cbaB	Cytochrome c oxidase subunit 2	1.9.3.1	KFAV_v1_0032	Thermus thermophilus (P98052, 42.5%)	Kyrpidia sp. EA-1 (A0A2K8NAH9, 99.35%)
	Putative cytochrome c oxidase subunit IIa		KFAV_v1_0853	Thermus thermophilus HB8 / ATCC 27634 / DSM 579 (P82543,39.39%)	Kyrpidia tusciae DSM 2912 / NBRC 15312
cbaB	Cytochrome c oxidase subunit 2	1.9.3.1	KFAV_v1_0854	Thermus thermophilus (P98052, 44%)	Kyrpidia tusciae DSM 2912 / NBRC 15312
cbaA	Cytochrome c oxidase subunit 1	1.9.3.1	KFAV_v1_0855	Thermus thermophilus (Q56408, 50.47%)	Kyrpidia sp. EA-1 (A0A2K8N6E8, 81.8%)
	Cytochrome c class I		KFAV_v1_0301	Synechocystis sp. PCC 6803 / Kazusa (P42351, 55.56%)	Kyrpidia tusciae DSM 2912 / NBRC 15312
	Cytochrome c class I		KFAV_v1_0302	Pseudaminobacter salicylatoxidans KCT001 (Q9K4M4, 24.59)	Kyrpidia sp. EA-1 (A0A2K8N6E8, 81.8%)
ccdA	Cytochrome c-type biogenesis protein		KFAV_v1_0743	Bacillus subtilis 168 (P45706, 47.41%)	Kyrpidia sp. EA-1 (A0A2K8NAN4, 99.57%)
cydA	cytochrome bb' ubiquinol oxidase (subunit I)	1.10.3.-	KFAV_v1_0816	Bacillus subtilis 168 (P94364, 47.5%)	Kyrpidia sp. EA-1 (A0A2K8N6Q1, 99.34%)
cydB	Cytochrome d ubiquinol oxidase subunit II	1.10.3.-	KFAV_v1_0817	Escherichia coli K12 (P0ABK2, **32.97%)**	Kyrpidia sp. EA-1 (A0A2K8NCG7, 97.08%)
cydA	cytochrome bb' ubiquinol oxidase (subunit I)	1.10.3.-	KFAV_v1_1027	Bacillus subtilis 168 (P94364, 30.93%)	Kyrpidia sp. EA-1 (A0A2K8N4A5, 99.34%)
	Putative Cytochrome C		KFAV_v1_1028	Bacillus subtilis 168 (C0SP90, 24.87%)	Kyrpidia sp. EA-1 (A0A2K8N4B4, 99.35%)
ctaC	cytochrome caa3 oxidase (subunit II)	1.9.3.1	KFAV_v1_0861	Bacillus pseudofirmus OF4 (Q04441, 45.03%)	Kyrpidia sp. EA-1 (A0A2K8N514, 97.31%)
ctaD	cytochrome caa3 oxidase (subunit I)	1.9.3.1	KFAV_v1_0862	Bacillus sp. PS3 (P16262, 60.83%)	Kyrpidia sp. EA-1 (A0A2K8N425, 91.07%)
ctaE	cytochrome caa3 oxidase (subunit III)	1.9.3.1	KFAV_v1_0863	Bacillus sp. PS3 (Q03439, 60.11%)	Kyrpidia tusciae DSM 2912 / NBRC 15312
ctaF	cytochrome caa3 oxidase (subunit IV)	1.9.3.1	KFAV_v1_0864	Bacillus subtilis 168 (P24013, 47.06%)	Kyrpidia tusciae DSM 2912 / NBRC 15312
qoxD	cytochrome aa3-600 quinol oxidase (subunit IV)	1.10.3.12	KFAV_v1_2235	Bacillus subtilis 168 (P34959, 32.56%)	Kyrpidia sp. EA-1 (A0A2K8N7H8, 96.61%)
ctaE	cytochrome caa3 oxidase (subunit III)	1.9.3.1	KFAV_v1_2236	Bacillus sp. PS3 (Q03439, 53.33%)	Kyrpidia sp. EA-1 (A0A2K8N9J8, 99.39%)
qoxB	cytochrome aa3-600 quinol oxidase (subunit I)	1.10.3.12	KFAV_v1_2237	Bacillus subtilis 168 (P34956, 61.67%)	Kyrpidia sp. EA-1 (A0A2K8N7H3, 98.41%)
qoxA	Quinol oxidase subunit 2	1.10.3.12	KFAV_v1_2238	Bacillus cereus ATCC 14579 / DSM 31 / JCM 2152 / NBRC 15305 / NCIMB 9373 / NRRL B-3711 (Q81HT3, 49.2%)	
qcrA	menaquinol:cytochrome c oxidoreductase (iron-sulfur subunit)	1.10.2.-	KFAV_v1_1708	Bacillus subtilis 168 (P46911, 45.96%)	Kyrpidia sp. EA-1 (A0A2K8N7R5, 100%)
qcrB	menaquinol:cytochrome c oxidoreductase (cytochrome b subunit)		KFAV_v1_1709	Geobacillus thermodenitrificans K1041 (Q45658, 74.88%)	Kyrpidia tusciae DSM 2912 / NBRC 15312
qcrC	Menaquinol-cytochrome c reductase cytochrome b/c subunit		KFAV_v1_1710	Geobacillus thermodenitrificans K1041 (Q45659, 45.19%)	Kyrpidia sp. EA-1 (A0A2K8N673 5, 100%)
qcrA	menaquinol:cytochrome c oxidoreductase (iron-sulfur subunit)	1.10.2.-	KFAV_v1_2630	Bacillus subtilis 168 (P46911, 38.65%)	Kyrpidia tusciae DSM 2912 / NBRC 15312
qcrC	Cytochrome bc1 complex cytochrome c subunit	1.10.2.2	KFAV_v1_1767	Mycobacterium bovis ATCC BAA-935 / AF2122/97 (P63888, 58.06%)	Kyrpidia tusciae DSM 2912 / NBRC 15312
CcmE	Cytochrome c biogenesis protein		KFAV_v1_2897	Nitrosospira multiformis (Q2Y9Q8, 30.77%)	Kyrpidia sp. EA-1 (A0A2K8N9J1, 100%)
	Cytochrome C biogenesis protein		KFAV_v1_2898	Pseudomonas aeruginosa ATCC 15692 / DSM 22644 / CIP 104116 / JCM 14847 / LMG 12228 / 1C / PRS 101 / PAO1 (Q9I3N2, 37.46%)	Kyrpidia sp. EA-1 (A0A2K8N8U6, 99.4%)
	Cytochrome c-type biogenesis protein		KFAV_v1_2899	Escherichia coli K12 (P0ABM9 , 34.78%)	

[a] *K. Spormannii* FAVT5 protein identifier

[b] Organism with highest scoring BLAST hit to *K. Spormannii* FAVT5 protein in SwissProt database. In parentheses: SwissProt accession number of best hit, amino acid identity.

[c] Organism with highest scoring BLAST hit to *K. Spormannii* FAVT5 protein in TrEMBL database. In parentheses: TrEMBL accession number of best hit, amino acid identity.

Table S4: Transcriptomic results of Kyrpidia spormannii FAVT5 hydrogen-limited chemostat culture.

Name	Label	Product	RPKM_1	RPKM_2	RPKM_3	RPKM_4
KFAV_v1_0616	KFAV_v1_0616	protein of unknown function	71862	118857	108282	83254
KFAV_v1_3431	KFAV_v1_3431	conserved exported protein of unknown function	19424	24821	24523	22169
cspC_1	KFAV_v1_1621	cold-shock protein	9951	9471	10692	10063
cbbL	KFAV_v1_3127	Ribulose bisphosphate carboxylase large subunit	8870	8733	9284	9637
codY_2	KFAV_v1_2579	GTP-sensing transcriptional pleiotropic repressor CodY	8464	8611	7530	9359
prk	KFAV_v1_3124	Phosphoribulokinase	8245	7745	7353	8619
cbbS	KFAV_v1_3128	Ribulose bisphosphate carboxylase small subunit	7985	7725	7267	8159
cspB	KFAV_v1_1420	major cold-shock protein, RNA helicase co-factor, RNA co-chaperone	7848	7700	7233	7707
cbbX	KFAV_v1_3129	RuBisCO accessory protein	7607	7672	7095	7355
gapA_2	KFAV_v1_3125	glyceraldehyde-3-phosphate dehydrogenase	7017	6728	6858	6631
fbaA_1	KFAV_v1_3123	fructose-1,6-bisphosphate aldolase	6607	6725	6853	6552
trxBB_2	KFAV_v1_3130	ferredoxin-NADP+ reductase (flavodoxin)	6552	6552	6526	6289
KFAV_v1_1783	KFAV_v1_1783	conserved protein of unknown function	5937	6468	6068	5856
acoA_1	KFAV_v1_2222	acetoin dehydrogenase E1 component (TPP-dependent alpha subunit)	5066	6064	6026	5569
acoB	KFAV_v1_2221	acetoin dehydrogenase E1 component (TPP-dependent beta subunit)	4943	5921	5923	5432
KFAV_v1_2292	KFAV_v1_2292	SPW repeat-containing protein	4799	5223	5324	5199
fla	KFAV_v1_3233	Flagellin	4764	5031	5250	4953
tkt_3	KFAV_v1_3131	Transketolase	4685	4513	5210	4903
hupL_2	KFAV_v1_2745	Hydrogenase large subunit, group 2a	4273	4347	5202	4849
KFAV_v1_3458	KFAV_v1_3458	conserved protein of unknown function	4252	4301	5177	4846
acoC	KFAV_v1_2220	acetoin dehydrogenase E2 component (dihydrolipoamide acetyltransferase)	4115	4284	4898	4823
KFAV_v1_0697	KFAV_v1_0697	conserved protein of unknown function	3963	3980	4252	4791
hupL_1	KFAV_v1_1432	Hydrogenase large subunit, group 2a	3848	3908	4133	4216
nuoD_1	KFAV_v1_3137	NADH-quinone oxidoreductase subunit D	3836	3752	4023	3993
KFAV_v1_1502	KFAV_v1_1502	conserved protein of unknown function	3783	3746	4019	3930
nuoA_1	KFAV_v1_3134	NADH-quinone oxidoreductase subunit A	3774	3617	3809	3507
hupS_1	KFAV_v1_1431	Hydrogenase small subunit	3331	3594	3562	3495
yadR	KFAV_v1_1533	putative chaperone involved in Fe-S cluster assembly and activation; hesB-like	3318	3571	3406	3457
KFAV_v1_1629	KFAV_v1_1629	protein of unknown function	3277	3412	3349	3406
hucE	KFAV_v1_2740	Fe-S cluster biogenesis protein	3270	3333	3224	3390
qcrB	KFAV_v1_1709	menaquinol:cytochrome c oxidoreductase (cytochrome b subunit)	3242	3326	3194	3380
hypC_2	KFAV_v1_2738	Hydrogenase maturation factor, HypC	3136	3321	3140	3327
qcrC_1	KFAV_v1_1710	Menaquinol-cytochrome c reductase cytochrome b/c subunit	3133	3148	3119	3282
tufA	KFAV_v1_0160	elongation factor Tu	3110	3146	3049	3264
nuoM_1	KFAV_v1_3132	NADH-quinone oxidoreductase subunit M	3097	3116	2958	3247
acpA	KFAV_v1_1976	acyl carrier protein	2999	3035	2938	3177
KFAV_v1_0343	KFAV_v1_0343	protein of unknown function	2943	2953	2887	3176
nuoB_1	KFAV_v1_3135	NADH-quinone oxidoreductase subunit B	2932	2903	2862	3148
hypB_2	KFAV_v1_2747	Hydrogenase maturation factor, HypB	2851	2861	2782	3114
glpX_1	KFAV_v1_3126	fructose 1,6-bisphosphatase class II	2835	2724	2743	3108
qcrA_1	KFAV_v1_1708	menaquinol:cytochrome c oxidoreductase (iron-sulfur subunit)	2783	2708	2706	3072
ctaE_1	KFAV_v1_0863	cytochrome caa3 oxidase (subunit III)	2709	2694	2632	3026
qcrC_2	KFAV_v1_1767	Cytochrome bc1 complex cytochrome c subunit	2705	2694	2562	3018
hcaA	KFAV_v1_2742	Tetratricopeptide repeat protein	2695	2638	2548	2867
tadA	KFAV_v1_0036	tRNA specific adenosine A34 deaminase	2575	2637	2494	2812
KFAV_v1_1430	KFAV_v1_1430	conserved protein of unknown function	2565	2625	2481	2808
nixA_2	KFAV_v1_2735	High-affinity nickel transport protein, nixA	2555	2486	2471	2765
KFAV_v1_2339	KFAV_v1_2339	conserved exported protein of unknown function	2541	2482	2463	2709

KFAV_v1_2741	KFAV_v1_2741	conserved protein of unknown function	2446	2375	2460	2678
hpf	KFAV_v1_3078	ribosome-associated sigma 54 modulation protein; ribosome dimerisation factor	2373	2248	2449	2605
KFAV_v1_2737	KFAV_v1_2737	Hydrogenase maturation factor	2347	2234	2413	2603
fusA	KFAV_v1_0159	elongation factor G	2338	2224	2321	2511
nuoI_1	KFAV_v1_3139	NADH-quinone oxidoreductase subunit I	2335	2220	2313	2458
rpmEA	KFAV_v1_3538	ribosomal protein L31	2323	2210	2313	2363
nuoC_1	KFAV_v1_3136	NADH-quinone oxidoreductase subunit C	2270	2190	2195	2361
KFAV_v1_2732	KFAV_v1_2732	Glutaredoxin	2263	2178	2057	2344
rplA	KFAV_v1_0150	ribosomal protein L1 (BL1)	2213	2133	2037	2290
rpiB_1	KFAV_v1_3133	ribose 5-phosphate isomerase	2212	2132	2036	2244
KFAV_v1_2749	KFAV_v1_2749	conserved protein of unknown function	2200	2126	1985	2244
atpB	KFAV_v1_3511	ATP synthase (subunit a, component F0)	2195	2069	1971	2207
ctaD	KFAV_v1_0862	cytochrome caa3 oxidase (subunit I)	2171	1990	1951	2168
rplS	KFAV_v1_1963	ribosomal protein L19	2110	1980	1936	2095
nuoH_1	KFAV_v1_3138	NADH-quinone oxidoreductase subunit H	2104	1941	1934	2086
yqeY	KFAV_v1_1248	conserved protein of unknown function with tRNA aminoacid amidase domain	2094	1902	1914	1997
qcrA_2	KFAV_v1_2630	menaquinol:cytochrome c oxidoreductase (iron-sulfur subunit)	2074	1886	1851	1956
hypA_2	KFAV_v1_2748	Hydrogenase maturation factor, HypA	2031	1876	1819	1937
sigA	KFAV_v1_1270	RNA polymerase major sigma-43 factor (sigma-A)	2007	1852	1816	1935
KFAV_v1_2100	KFAV_v1_2100	Thiol-disulfide oxidoreductase	1951	1839	1775	1872
hupS_2	KFAV_v1_2746	Hydrogenase small subunit	1902	1833	1746	1829
ctaC	KFAV_v1_0861	cytochrome caa3 oxidase (subunit II)	1834	1826	1742	1820
rpmB	KFAV_v1_1990	50S ribosomal protein L28	1827	1799	1721	1815
ctaF	KFAV_v1_0864	cytochrome caa3 oxidase (subunit IV)	1820	1760	1716	1813
rplK	KFAV_v1_0149	ribosomal protein L11 (BL11)	1817	1744	1706	1789
KFAV_v1_3384	KFAV_v1_3384	Type IV pilin	1770	1691	1689	1780
rpsO	KFAV_v1_1861	ribosomal protein S15 (BS18)	1755	1676	1684	1773
rplB	KFAV_v1_0165	ribosomal protein L2 (BL2)	1738	1644	1645	1766
rpmA	KFAV_v1_1164	ribosomal protein L27 (BL24)	1731	1609	1645	1743
KFAV_v1_1736	KFAV_v1_1736	Ferredoxin	1707	1608	1609	1738
cysK	KFAV_v1_2095	cysteine synthase	1702	1590	1605	1722
fixA	KFAV_v1_1950	Protein FixA	1697	1561	1594	1647
KFAV_v1_0865	KFAV_v1_0865	conserved membrane protein of unknown function	1684	1556	1590	1643
KFAV_v1_2548	KFAV_v1_2548	conserved protein of unknown function	1672	1552	1575	1636
KFAV_v1_1610	KFAV_v1_1610	conserved protein of unknown function	1663	1511	1557	1635
KFAV_v1_1946	KFAV_v1_1946	conserved protein of unknown function	1663	1501	1546	1627
dnaG	KFAV_v1_1269	DNA primase	1648	1493	1537	1618
KFAV_v1_1981	KFAV_v1_1981	Metal-binding protein	1637	1480	1526	1608
sodA	KFAV_v1_2618	superoxide dismutase (Mn[2+]-dependent)	1625	1479	1519	1587
rulS	KFAV_v1_0156	K-turn RNA binding protein; alternative ribosomal protein L7A	1616	1473	1504	1561
hcaB	KFAV_v1_2739	NHL repeat protein	1590	1434	1475	1500
paaK	KFAV_v1_2132	phenylacetyl-CoA ligase	1580	1428	1464	1436
KFAV_v1_1509	KFAV_v1_1509	conserved protein of unknown function	1560	1428	1455	1409
cbaB_2	KFAV_v1_0854	Cytochrome c oxidase subunit 2	1556	1407	1446	1396
nusG	KFAV_v1_0148	RNA polymerase elongation pause factor	1511	1407	1445	1388
KFAV_v1_1932	KFAV_v1_1932	conserved protein of unknown function	1507	1369	1433	1379
etfA_2	KFAV_v1_1949	Electron transfer flavoprotein subunit alpha	1498	1363	1423	1361
KFAV_v1_0213	KFAV_v1_0213	conserved protein of unknown function	1479	1360	1390	1331
hypD_2	KFAV_v1_2736	protein required for maturation of hydrogenases	1464	1341	1389	1327
rpfA	KFAV_v1_1645	RNA degradation presenting factor (ribosomal protein S1 homolog)	1450	1327	1372	1326
nuoN_1	KFAV_v1_3143	NADH-quinone oxidoreductase subunit N	1427	1310	1370	1321
KFAV_v1_0867	KFAV_v1_0867	Aldo/keto reductase	1398	1295	1370	1315

KFAV_v1_2744	KFAV_v1_2744	Hydrogenase maturation protease	1395	1293	1356	1314
KFAV_v1_2733	KFAV_v1_2733	Phosphoheptose isomerase	1389	1289	1352	1296
KFAV_v1_2338	KFAV_v1_2338	conserved exported protein of unknown function	1362	1284	1347	1295
atpD_1	KFAV_v1_3504	fragment of ATP synthase (subunit beta, component F1) (part 2)	1329	1284	1325	1295
veg	KFAV_v1_0064	conserved hypothetical protein	1319	1272	1320	1247
rpsL	KFAV_v1_0157	ribosomal protein S12 (BS12)	1316	1260	1318	1247
KFAV_v1_0287	KFAV_v1_0287	protein of unknown function	1315	1258	1291	1244
rpsG	KFAV_v1_0158	ribosomal protein S7 (BS7)	1313	1246	1287	1218
hupN_1	KFAV_v1_0083	HU-related DNA-binding protein; phage SPbeta	1312	1225	1255	1213
rbcR	KFAV_v1_3122	RuBisCO operon transcriptional regulator	1308	1216	1243	1210
adk	KFAV_v1_0183	adenylate kinase	1308	1211	1200	1208
KFAV_v1_3011	KFAV_v1_3011	Cytochrome C551	1303	1209	1162	1207
actP_1	KFAV_v1_1055	Cation acetate symporter	1301	1189	1150	1199
fixX	KFAV_v1_1947	Ferredoxin-like protein	1291	1171	1132	1199
KFAV_v1_1707	KFAV_v1_1707	protein of unknown function	1284	1142	1131	1188
ahpA_2	KFAV_v1_2101	biofilm-specific peroxidase; 2-cys peroxiredoxin	1273	1130	1123	1186
rplW	KFAV_v1_0164	ribosomal protein L23	1271	1116	1122	1159
tig	KFAV_v1_1125	prolyl isomerase (trigger factor)	1262	1100	1093	1145
KFAV_v1_1462	KFAV_v1_1462	conserved exported protein of unknown function	1231	1087	1080	1138
rpsS	KFAV_v1_0166	ribosomal protein S19 (BS19)	1229	1084	1071	1132
rny	KFAV_v1_1807	endoribonuclease Y	1226	1064	1069	1126
atpH	KFAV_v1_3508	ATP synthase subunit delta	1210	1064	1064	1122
nuoK_1	KFAV_v1_3141	NADH-quinone oxidoreductase subunit K	1206	1059	1045	1122
cbaA_2	KFAV_v1_0855	Cytochrome c oxidase subunit 1	1193	1054	1045	1109
rplR	KFAV_v1_0178	ribosomal protein L18	1190	1054	1039	1104
rpsH	KFAV_v1_0176	ribosomal protein S8 (BS8)	1187	1047	1030	1091
atpA	KFAV_v1_3507	ATP synthase (subunit alpha, component F1)	1172	1045	1030	1074
KFAV_v1_3276	KFAV_v1_3276	conserved protein of unknown function	1168	1034	1026	1063
moaA	KFAV_v1_2870	GTP 3',8-cyclase	1150	1018	1026	1063
hypB_1	KFAV_v1_1440	Hydrogenase maturation factor, HypB	1142	1015	1012	1062
rplL	KFAV_v1_0152	ribosomal protein L12 (BL9)	1134	1013	1010	1055
rplNA	KFAV_v1_0172	ribosomal protein L14	1127	1012	1005	1045
yabR	KFAV_v1_0091	putative RNA degradation protein; polyribonucleotide nucleotidyltransferase or phosphorylase	1126	994	1003	1041
fixC	KFAV_v1_1948	Protein FixC	1125	986	1003	1030
rpsI	KFAV_v1_0195	ribosomal protein S9	1103	983	994	1021
rpoA	KFAV_v1_0191	RNA polymerase (alpha subunit)	1094	981	992	1019
KFAV_v1_1702	KFAV_v1_1702	Ethanolamine utilization protein EutJ	1073	979	978	1016
KFAV_v1_1245	KFAV_v1_1245	conserved protein of unknown function	1068	976	968	1015
atpD_2	KFAV_v1_3505	fragment of ATP synthase (subunit beta, component F1) (part 1)	1065	976	968	1007
mapA	KFAV_v1_0184	methionine aminopeptidase	1060	974	952	985
rplQ	KFAV_v1_0192	ribosomal protein L17 (BL15)	1058	943	952	979
secY	KFAV_v1_0182	preprotein translocase subunit	1055	940	938	973
hypE_1	KFAV_v1_1438	Hydrogenase expression/formation protein, HypE	1055	929	922	972
rpsT	KFAV_v1_2151	ribosomal protein S20 (BS20)	1052	912	918	969
rplM	KFAV_v1_0194	ribosomal protein L13	1046	907	912	968
atpF	KFAV_v1_3509	ATP synthase (subunit b, component F0)	1034	904	906	959
KFAV_v1_0589	KFAV_v1_0589	conserved protein of unknown function	1033	901	903	958
KFAV_v1_1755	KFAV_v1_1755	conserved protein of unknown function	1025	891	900	949
metK	KFAV_v1_3261	S-adenosylmethionine synthetase	1008	877	896	949
rpsK	KFAV_v1_0189	ribosomal protein S11 (BS11)	1005	865	894	948
KFAV_v1_3386	KFAV_v1_3386	conserved protein of unknown function	994	862	887	929
KFAV_v1_1433	KFAV_v1_1433	Putative hydrogenase maturation protease (HupD)	993	860	882	925

nuoL_1	KFAV_v1_3142	NADH-quinone oxidoreductase subunit L	992	836	881	919
leuA	KFAV_v1_2490	2-isopropylmalate synthase	976	831	867	915
rpsJ	KFAV_v1_0161	ribosomal protein S10 (BS13); transcription antitermination factor	972	824	861	910
rplC	KFAV_v1_0162	ribosomal protein L3 (BL3)	968	817	858	908
gcvH_1	KFAV_v1_1652	glycine cleavage system protein H (lipoyl acceptor protein)	963	813	857	903
KFAV_v1_1163	KFAV_v1_1163	Ribosomal-processing cysteine protease Prp	955	811	854	898
rpsQ	KFAV_v1_0171	ribosomal protein S17 (BS16)	952	809	848	892
clpX	KFAV_v1_1127	protein unfolding ATPase required for presentation of proteins to proteases; Maxwell's demon	949	809	846	886
KFAV_v1_2604	KFAV_v1_2604	Phage shock protein A	946	807	841	874
KFAV_v1_2573	KFAV_v1_2573	Sec-independent protein translocase protein TatA (fragment)	938	807	838	872
rpsB	KFAV_v1_1882	ribosomal protein S2	938	793	836	872
cmk	KFAV_v1_1643	cytidylate kinase	929	792	832	871
hemQ	KFAV_v1_2103	iron-coproporphyrin (coproheme) decarboxylase	920	780	831	864
groEL	KFAV_v1_0243	chaperonin large subunit	920	774	830	863
rpsE	KFAV_v1_0179	ribosomal protein S5	917	774	828	854
KFAV_v1_2531	KFAV_v1_2531	protein of unknown function	903	772	826	847
perR	KFAV_v1_1461	transcriptional regulator (Fur family)	902	769	825	844
KFAV_v1_1412	KFAV_v1_1412	conserved protein of unknown function	902	768	824	837
rpmD	KFAV_v1_0180	ribosomal protein L30 (BL27)	901	763	817	832
hypE_2	KFAV_v1_2734	Hydrogenase expression/formation protein, HypE	899	763	814	831
rpsC	KFAV_v1_0168	ribosomal protein S3 (BS3)	891	761	811	827
KFAV_v1_3232	KFAV_v1_3232	protein of unknown function	885	759	794	823
KFAV_v1_0185	KFAV_v1_0185	conserved protein of unknown function	885	759	792	823
purA	KFAV_v1_3584	adenylosuccinate synthetase	883	757	791	823
atpG	KFAV_v1_3506	ATP synthase (subunit gamma, component F1)	882	755	783	822
yitA	KFAV_v1_0204	putative sulfate adenylyltransferase	882	746	779	816
KFAV_v1_0852	KFAV_v1_0852	protein of unknown function	878	738	770	816
KFAV_v1_2743	KFAV_v1_2743	Hydrogenase maturation protease	877	732	751	812
pyrR	KFAV_v1_2058	transcriptional attenuator and uracil phosphoribosyltransferase activity	874	732	747	807
ppa	KFAV_v1_1053	Inorganic pyrophosphatase	869	732	744	796
KFAV_v1_2871	KFAV_v1_2871	Nucleotidyltransferase family protein	869	732	743	795
KFAV_v1_2900	KFAV_v1_2900	conserved protein of unknown function	868	732	741	793
rpsD	KFAV_v1_0190	30S ribosomal subunit protein S4	866	726	740	789
hypC_1	KFAV_v1_1436	Hydrogenase maturation factor, Hup	863	718	738	784
coxL_2	KFAV_v1_2876	Carbon monoxide dehydrogenase large chain	861	718	735	782
KFAV_v1_0568	KFAV_v1_0568	Diguanylate cyclase	860	711	734	780
KFAV_v1_1398	KFAV_v1_1398	conserved protein of unknown function	858	710	725	775
phoP_1	KFAV_v1_2730	Alkaline phosphatase synthesis transcriptional regulatory protein PhoP	857	710	719	772
rplE	KFAV_v1_0174	ribosomal protein L5 (BL6)	847	710	715	766
KFAV_v1_1532	KFAV_v1_1532	conserved protein of unknown function	840	710	713	766
KFAV_v1_2226	KFAV_v1_2226	YhfH family protein	837	710	713	765
KFAV_v1_0510	KFAV_v1_0510	conserved protein of unknown function	834	706	712	759
cysH	KFAV_v1_0202	Phosphoadenosine phosphosulfate reductase	829	703	711	758
KFAV_v1_1421	KFAV_v1_1421	Sugar ABC transporter substrate-binding protein	829	701	709	753
pgk_2	KFAV_v1_3121	phosphoglycerate kinase	826	698	709	739
rplF	KFAV_v1_0177	ribosomal protein L6 (BL8)	824	695	703	737
rplP	KFAV_v1_0169	ribosomal protein L16	824	694	701	734
KFAV_v1_0424	KFAV_v1_0424	transposase	811	688	700	734
rplU	KFAV_v1_1162	ribosomal protein L21 (BL20)	796	684	698	729
KFAV_v1_2553	KFAV_v1_2553	protein of unknown function	791	679	697	725
rplX	KFAV_v1_0173	ribosomal protein L24 (BL23)	788	675	696	723
KFAV_v1_2593	KFAV_v1_2593	conserved protein of unknown function	786	666	678	721

codY_1	KFAV_v1_1919	transcriptional regulator, GTP and BCAA-dependent	782	661	678	714
KFAV_v1_3387	KFAV_v1_3387	conserved protein of unknown function	782	661	676	709
leuB	KFAV_v1_2489	3-isopropylmalate dehydrogenase	779	660	676	707
rplV	KFAV_v1_0167	ribosomal protein L22 (BL17)	777	657	675	706
KFAV_v1_1054	KFAV_v1_1054	conserved protein of unknown function	774	654	675	701
KFAV_v1_0302	KFAV_v1_0302	Cytochrome c class I	769	650	669	698
nuoJ_1	KFAV_v1_3140	NADH-quinone oxidoreductase subunit J	768	646	669	698
KFAV_v1_1434	KFAV_v1_1434	conserved protein of unknown function	762	645	669	689
yeeD	KFAV_v1_3519	conserved hypothetical protein	760	645	665	686
KFAV_v1_2869	KFAV_v1_2869	conserved protein of unknown function	760	643	661	685
hslR	KFAV_v1_0084	ribosomal RNA binding protein involved in 50S recycling; heat shock protein	754	638	661	685
pdxT	KFAV_v1_0017	glutamine amidotransferase for pyridoxal phosphate synthesis; pyridoxal 5'-phosphate synthase complex, glutamine amidotransferase subunit PdxT	753	634	655	682
rplD	KFAV_v1_0163	ribosomal protein L4	750	634	654	675
hypD_1	KFAV_v1_1437	protein required for maturation of hydrogenases	749	633	650	670
KFAV_v1_3250	KFAV_v1_3250	conserved protein of unknown function	747	618	645	668
rplJ	KFAV_v1_0151	ribosomal protein L10 (BL5)	746	617	637	668
KFAV_v1_1671	KFAV_v1_1671	conserved protein of unknown function	746	616	635	666
phoU	KFAV_v1_2753	Phosphate-specific transport system accessory protein PhoU homolog	741	616	623	661
cstA	KFAV_v1_1609	carbon starvation protein	738	612	621	657
ilvD	KFAV_v1_2366	dihydroxy-acid dehydratase	733	610	621	653
rpsR	KFAV_v1_3590	ribosomal protein S18	725	610	620	650
trxA	KFAV_v1_1096	Thioredoxin 1	720	607	620	644
KFAV_v1_1503	KFAV_v1_1503	YlbF family regulator	717	607	617	641
speD	KFAV_v1_2552	S-adenosylmethionine decarboxylase	717	600	617	637
ndoA	KFAV_v1_0224	endoribonuclease toxin	708	599	614	634
rpsU	KFAV_v1_1247	ribosomal protein S21	706	592	610	632
rplY	KFAV_v1_0075	50S ribosomal protein L25	705	591	606	632
clpP_1	KFAV_v1_1126	ATP-dependent Clp protease proteolytic subunit; Maxwell's demon	704	589	605	631
secE	KFAV_v1_0147	Protein translocase subunit SecE	703	589	603	631
KFAV_v1_3230	KFAV_v1_3230	transposase (fragment)	700	582	602	626
hypF_2	KFAV_v1_2731	Carbamoyltransferase, HypF	697	580	598	625
KFAV_v1_2874	KFAV_v1_2874	ATPase	692	580	596	622
KFAV_v1_2495	KFAV_v1_2495	protein of unknown function	686	579	595	618
ilvC	KFAV_v1_2491	acetohydroxy-acid isomeroreductase (NADP-dependent)	675	576	592	615
KFAV_v1_0799	KFAV_v1_0799	conserved protein of unknown function	673	573	592	615
KFAV_v1_2304	KFAV_v1_2304	DNA-binding response regulator	664	569	592	615
clpY	KFAV_v1_1920	two-component ATP-dependent protease (ATPase and chaperone)	661	568	586	615
glmM	KFAV_v1_0207	phosphoglucosamine mutase	659	564	582	614
KFAV_v1_3388	KFAV_v1_3388	conserved protein of unknown function	659	562	581	612
rpsM	KFAV_v1_0188	ribosomal protein S13	658	560	579	611
KFAV_v1_0696	KFAV_v1_0696	ABC transporter ATP-binding protein	658	560	578	606
KFAV_v1_3513	KFAV_v1_3513	conserved protein of unknown function	657	551	570	601
KFAV_v1_2922	KFAV_v1_2922	conserved protein of unknown function	653	550	570	595
sucD	KFAV_v1_1927	succinyl-CoA synthetase (alpha subunit)	648	550	569	594
pyrAA_2	KFAV_v1_2054	pyrimidine-specific carbamoyl-phosphate synthetase (small subunit, glutaminase subunit)	647	549	569	593
KFAV_v1_2217	KFAV_v1_2217	conserved protein of unknown function	642	549	569	593
rplT	KFAV_v1_2527	ribosomal protein L20	642	547	565	592
KFAV_v1_1393	KFAV_v1_1393	Glutaredoxin	639	547	560	592
ilvB	KFAV_v1_2493	acetohydroxy-acid synthase (large subunit)	639	545	556	580
carD	KFAV_v1_0137	RNA polymerase-binding transcription factor CarD	638	542	556	578
coxS_2	KFAV_v1_2877	Carbon monoxide dehydrogenase small chain	638	539	546	574
glnH	KFAV_v1_0127	glutamine transporter subunit ; periplasmic binding component of ABC superfamily	637	539	546	573

KFAV_v1_2547	KFAV_v1_2547	protein of unknown function	636	536	545	572
ysxA	KFAV_v1_1152	conserved nucleotide-related metabolism protein	631	535	543	572
yumB	KFAV_v1_2310	NADH dehydrogenase-like protein YumB	624	533	541	570
KFAV_v1_2362	KFAV_v1_2362	conserved protein of unknown function	623	528	539	568
yjnA	KFAV_v1_0203	putative membrane transporter protein YjnA	622	528	539	567
fabG_4	KFAV_v1_1977	beta-ketoacyl-acyl carrier protein reductase	622	528	539	567
rimO	KFAV_v1_1813	Ribosomal protein S12 methylthiotransferase RimO	613	526	538	566
bioB	KFAV_v1_2810	biotin synthase	612	524	536	563
KFAV_v1_0353	KFAV_v1_0353	conserved protein of unknown function	606	523	536	561
infC	KFAV_v1_2529	initiation factor IF-3	605	521	534	561
cshA	KFAV_v1_1810	ATP-dependent RNA helicase; cold shock	603	520	533	554
ftsZ	KFAV_v1_1557	cell-division initiation protein	603	519	529	552
coxM_2	KFAV_v1_2875	Carbon monoxide dehydrogenase medium chain	601	510	528	550
KFAV_v1_2898	KFAV_v1_2898	Cytochrome C biogenesis protein	598	510	525	547
KFAV_v1_0718	KFAV_v1_0718	conserved membrane protein of unknown function	598	508	524	547
glmU	KFAV_v1_0073	bifunctional glucosamine-1-phosphate N-acetyltransferase/UDP-N-acetylglucosamine pyrophosphorylase	598	508	522	545
argG	KFAV_v1_0543	argininosuccinate synthase	597	505	522	544
fumA	KFAV_v1_2862	fumarate hydratase class I	592	504	517	544
lexA	KFAV_v1_1766	transcriptional repressor of the SOS regulon	588	498	517	543
KFAV_v1_2800	KFAV_v1_2800	SCO1 protein homolog	588	495	513	541
KFAV_v1_1187	KFAV_v1_1187	conserved protein of unknown function	587	495	509	539
ilvE_2	KFAV_v1_2494	putative branched-chain-amino-acid aminotransferase	587	490	509	538
KFAV_v1_0505	KFAV_v1_0505	protein of unknown function	587	490	509	538
KFAV_v1_1215	KFAV_v1_1215	conserved protein of unknown function	585	488	507	537
KFAV_v1_1612	KFAV_v1_1612	conserved protein of unknown function	585	487	507	536
KFAV_v1_3583	KFAV_v1_3583	protein of unknown function	583	486	501	534
KFAV_v1_2532	KFAV_v1_2532	conserved protein of unknown function	572	485	500	531
hypA_1	KFAV_v1_1439	Hydrogenase maturation factor, HypA	572	483	498	526
ndoAI	KFAV_v1_0223	antitoxin EndoAI	571	482	495	526
KFAV_v1_1510	KFAV_v1_1510	conserved protein of unknown function	571	477	495	526
dnaH	KFAV_v1_0052	regulatory subunit of the DNA replication complex	569	476	493	525
KFAV_v1_1246	KFAV_v1_1246	conserved protein of unknown function	562	472	492	523
gapB	KFAV_v1_1514	glyceraldehyde-3-phosphate dehydrogenase (NADP-dependent, gluconeogenesis)	560	470	491	522
infA	KFAV_v1_0186	initiation factor IF-I	554	468	490	521
csoR	KFAV_v1_1400	repressor of copper utilisation proteins - Cu(I)	550	466	489	520
sufC	KFAV_v1_1038	sulfur mobilizing ABC protein, ATPase	549	466	489	518
hypF_1	KFAV_v1_1441	Carbamoyltransferase, hypF	548	465	484	517
KFAV_v1_0423	KFAV_v1_0423	Integrase (fragment)	547	465	484	514
KFAV_v1_0051	KFAV_v1_0051	conserved protein of unknown function	547	464	484	511
copZ	KFAV_v1_1401	copper insertion chaperone and transporter component	547	462	482	511
KFAV_v1_0727	KFAV_v1_0727	Iron sulfur domain-containing, CDGSH-type	546	461	481	511
KFAV_v1_0928	KFAV_v1_0928	conserved protein of unknown function	545	457	475	506
nusB	KFAV_v1_1216	transcription termination factor NusB	543	456	474	501
KFAV_v1_3014	KFAV_v1_3014	transposase	540	454	472	500
KFAV_v1_1150	KFAV_v1_1150	conserved protein of unknown function	540	451	472	499
KFAV_v1_3145	KFAV_v1_3145	conserved protein of unknown function	536	449	471	497
yacL	KFAV_v1_0138	putative membrane protein possibly involved in RNA binding	536	444	470	496
KFAV_v1_3086	KFAV_v1_3086	conserved protein of unknown function	535	440	469	495
KFAV_v1_2940	KFAV_v1_2940	conserved protein of unknown function	534	439	469	494
KFAV_v1_0410	KFAV_v1_0410	transposase	534	439	468	494
fapR	KFAV_v1_1980	transcription factor controlling fatty acid and phospholipid metabolism (FapR - malonyl-Coa)	532	437	467	493
KFAV_v1_0090	KFAV_v1_0090	conserved protein of unknown function	530	435	465	493

KFAV_v1_1644	KFAV_v1_1644	1-acyl-sn-glycerol-3-phosphate acyltransferase	528	435	465	492
KFAV_v1_1203	KFAV_v1_1203	AraC family transcriptional regulator	528	434	463	491
glpX_2	KFAV_v1_3542	fructose 1,6-bisphosphatase class II	525	431	461	491
ytkL	KFAV_v1_2647	putative metal-dependent hydrolase	524	428	460	490
bioK	KFAV_v1_2813	L-lysine-8-amino-7-oxononanoate aminotransferase	521	428	460	488
rpoE	KFAV_v1_3552	putative DNA-directed RNA polymerase subunit delta	518	427	456	482
coxG	KFAV_v1_2878	Putative carbon monoxide dehydrogenase subunit G	517	426	455	481
KFAV_v1_0591	KFAV_v1_0591	transposase	517	426	449	481
sufB	KFAV_v1_1042	FeS cluster formation scaffold protein	517	425	447	481
KFAV_v1_0919	KFAV_v1_0919	GntR family transcriptional regulator	516	424	447	478
KFAV_v1_1642	KFAV_v1_1642	protein of unknown function	516	422	446	476
ilvH	KFAV_v1_2492	acetohydroxy-acid synthase (small subunit)	514	422	446	476
KFAV_v1_0250	KFAV_v1_0250	transposase	510	421	445	475
KFAV_v1_0866	KFAV_v1_0866	Peroxiredoxin (modular protein)	507	420	441	475
plsX	KFAV_v1_1979	phosphate:acyl-ACP acyltransferase	507	419	440	474
KFAV_v1_3596	KFAV_v1_3596	conserved protein of unknown function	506	419	440	472
KFAV_v1_2331	KFAV_v1_2331	conserved exported protein of unknown function	504	418	439	471
KFAV_v1_0724	KFAV_v1_0724	conserved protein of unknown function	502	418	438	470
gltX_1	KFAV_v1_0141	glutamyl-tRNA synthetase	501	418	438	469
CcmE	KFAV_v1_2897	Cytochrome c biogenesis protein	500	418	437	468
frr	KFAV_v1_1879	ribosome recycling factor	500	417	437	467
trpB_1	KFAV_v1_0709	tryptophan synthase (beta subunit)	499	416	436	465
yqhY	KFAV_v1_1213	alkaline shock protein	499	416	435	464
prs	KFAV_v1_0074	phosphoribosylpyrophosphate synthetase	498	416	435	461
pdxS	KFAV_v1_0016	glutamine amidotransferase for pyridoxal phosphate synthesis; pyridoxal 5'-phosphate synthase complex, synthase subunit	496	415	433	460
rpmC	KFAV_v1_0170	ribosomal protein L29	496	414	433	459
KFAV_v1_1528	KFAV_v1_1528	Transcriptional regulator	496	414	431	457
KFAV_v1_0469	KFAV_v1_0469	protein of unknown function	495	413	427	455
KFAV_v1_2306	KFAV_v1_2306	conserved protein of unknown function	495	412	427	455
fabF_1	KFAV_v1_1975	beta-ketoacyl-acyl carrier protein synthase II (involved in pimelate synthesis)	492	411	426	453
maf	KFAV_v1_1151	nucleoside triphosphate pyrophosphatase; septum formation DNA-binding protein (multicopy associated filamentation)	491	408	425	452
KFAV_v1_1044	KFAV_v1_1044	Rrf2 family transcriptional regulator	488	407	421	447
KFAV_v1_1620	KFAV_v1_1620	Glutamate synthase	487	405	419	444
KFAV_v1_1506	KFAV_v1_1506	conserved protein of unknown function	485	405	419	444
KFAV_v1_2901	KFAV_v1_2901	conserved protein of unknown function	484	405	418	444
KFAV_v1_2160	KFAV_v1_2160	conserved protein of unknown function	484	403	418	442
KFAV_v1_0023	KFAV_v1_0023	conserved protein of unknown function	482	402	417	442
KFAV_v1_1936	KFAV_v1_1936	conserved protein of unknown function	482	397	416	441
rnjA	KFAV_v1_1846	ribonuclease J1	478	396	415	441
hemA	KFAV_v1_1131	glutamyl-tRNA reductase	477	396	415	440
oxaAA	KFAV_v1_3612	Sec-independent factor for membrane protein insertion (YidC/SpoIIIJ family)	476	396	414	440
bioFC	KFAV_v1_2812	8-amino-7-oxononanoate synthase (pimeloyl-CoA-dependent)	473	394	411	439
ssbA	KFAV_v1_3591	single-strand DNA-binding protein	472	394	410	437
ftsH_1	KFAV_v1_0104	ATP-dependent cytoplasmic membrane protease	472	394	410	436
acuC	KFAV_v1_1062	protein deacetylase (regulates AcsA activity)	472	393	409	433
KFAV_v1_2623	KFAV_v1_2623	conserved protein of unknown function	471	393	409	431
adh_4	KFAV_v1_1858	Alcohol dehydrogenase	471	392	409	430
KFAV_v1_1325	KFAV_v1_1325	Amidase	470	389	407	428
KFAV_v1_2873	KFAV_v1_2873	conserved protein of unknown function	470	387	407	427
KFAV_v1_1656	KFAV_v1_1656	Trp RNA-binding attenuation protein MtrB	470	385	406	427
pyrH	KFAV_v1_1880	uridylate kinase	470	385	405	427
KFAV_v1_3239	KFAV_v1_3239	Flagellar hook-associated protein FlgK	469	384	404	427

rplO	KFAV_v1_0181	ribosomal protein L15	468	384	403	427
thrB	KFAV_v1_1189	Homoserine kinase	468	383	401	426
KFAV_v1_2872	KFAV_v1_2872	Xanthine dehydrogenase	468	383	401	425
ftsA	KFAV_v1_1556	cell-division protein essential for Z-ring assembly	466	382	401	423
acsA_2	KFAV_v1_2382	Acetoacetyl-coenzyme A synthetase	465	382	401	423
KFAV_v1_2533	KFAV_v1_2533	conserved protein of unknown function	464	379	399	422
rpsF	KFAV_v1_3592	ribosomal protein S6 (BS9)	461	379	399	421
KFAV_v1_0435	KFAV_v1_0435	Putative sigma54 specific transcriptional regulator (fragment)	460	379	397	421
KFAV_v1_0929	KFAV_v1_0929	conserved protein of unknown function	459	377	396	421
dps	KFAV_v1_0656	DNA protection during starvation protein	457	376	396	419
KFAV_v1_3294	KFAV_v1_3294	conserved exported protein of unknown function	457	376	395	419
KFAV_v1_0498	KFAV_v1_0498	conserved membrane protein of unknown function	457	376	394	418
hofD	KFAV_v1_3385	Leader peptidase / N-methyltransferase	457	375	393	417
pgsA_1	KFAV_v1_1812	CDP-diacylglycerol-glycerol-3-phosphate 3-phosphatidyltransferase	457	375	393	415
rpsP	KFAV_v1_1966	ribosomal protein S16 (BS17)	456	374	392	415
KFAV_v1_3389	KFAV_v1_3389	conserved exported protein of unknown function	455	374	392	412
bcp	KFAV_v1_2596	peroxiredoxin with versatile activity	452	373	391	412
KFAV_v1_2889	KFAV_v1_2889	Acyl-CoA synthetase	447	372	391	412
mreB	KFAV_v1_1153	cell-shape determining protein	447	372	389	410
KFAV_v1_1324	KFAV_v1_1324	conserved protein of unknown function	446	371	388	410
KFAV_v1_3229	KFAV_v1_3229	transposase (fragment)	444	371	388	410
KFAV_v1_2340	KFAV_v1_2340	conserved protein of unknown function	443	370	387	410
glnA_2	KFAV_v1_1768	glutamine synthetase	439	370	387	410
pheA	KFAV_v1_2496	Prephenate dehydratase	439	366	385	409
clpQ	KFAV_v1_1921	two-component ATP-dependent protease (N-terminal serine protease)	436	366	385	409
rpsNA	KFAV_v1_0175	ribosomal protein S14	435	366	385	406
rpoC	KFAV_v1_0155	RNA polymerase (beta' subunit)	434	365	383	406
pstBB_1	KFAV_v1_2754	phosphate ABC transporter (ATP-binding protein)	431	364	381	406
trmD	KFAV_v1_1964	tRNA(m1G37)methyltransferase	431	363	381	402
pyrAB_2	KFAV_v1_2053	pyrimidine-specific carbamoyl-phosphate synthetase (large subunit)	431	363	380	402
thrC	KFAV_v1_1188	threonine synthase	430	363	379	400
aroX	KFAV_v1_1061	bifunctional chorismate mutase type II-isozyme 3 (regulatory domain); 3-deoxy-D-arabino-heptulosonate 7-phosphate synthase;	430	362	379	397
KFAV_v1_1414	KFAV_v1_1414	conserved protein of unknown function	430	361	379	395
KFAV_v1_1428	KFAV_v1_1428	conserved protein of unknown function	427	361	378	391
proA	KFAV_v1_2497	gamma-glutamyl phosphate reductase	426	360	377	391
nixA_1	KFAV_v1_1442	High-affinity nickel-transport protein, NixA	425	360	377	390
KFAV_v1_2551	KFAV_v1_2551	protein of unknown function	424	358	376	390
KFAV_v1_3152	KFAV_v1_3152	C-di-GMP phosphodiesterase	423	358	376	389
KFAV_v1_0934	KFAV_v1_0934	conserved protein of unknown function	423	357	374	387
KFAV_v1_1063	KFAV_v1_1063	Acetoin utilization protein AcuB	421	356	373	387
KFAV_v1_0389	KFAV_v1_0389	protein of unknown function	418	354	371	386
uppS	KFAV_v1_1878	undecaprenyl pyrophosphate synthase	417	354	370	385
atpE	KFAV_v1_3510	ATP synthase (subunit c, component F0)	416	353	370	383
rimM	KFAV_v1_1965	16S rRNA processing protein	416	352	369	383
KFAV_v1_0741	KFAV_v1_0741	Class I SAM-dependent methyltransferase	415	351	368	383
trpD	KFAV_v1_1690	anthranilate phosphoribosyltransferase	412	351	367	382
KFAV_v1_1411	KFAV_v1_1411	protein of unknown function	410	351	367	381
KFAV_v1_0917	KFAV_v1_0917	2-oxoacid ferredoxin oxidoreductase	408	350	367	377
prfB	KFAV_v1_3075	Peptide chain release factor 2	407	350	365	376
KFAV_v1_1877	KFAV_v1_1877	Phosphatidate cytidylyltransferase	406	349	364	374
KFAV_v1_0535	KFAV_v1_0535	RNA polymerase sigma factor	403	349	364	374
pdhD	KFAV_v1_0731	dihydrolipoamide dehydrogenase E3 subunit of both pyruvate dehydrogenase and 2-oxoglutarate dehydrogenase complexes	402	349	363	374

accC	KFAV_v1_1212	acetyl-CoA carboxylase subunit (biotin carboxylase subunit)	401	347	363	374
KFAV_v1_0699	KFAV_v1_0699	Acetolactate synthase	400	347	358	374
secA	KFAV_v1_3077	translocase binding subunit (ATPase)	399	347	357	372
ytcI	KFAV_v1_1655	putative acyl-coenzyme A synthetase	398	347	357	371
folD	KFAV_v1_1219	methylenetetrahydrofolate dehydrogenase; methenyltetrahydrofolate cyclohydrolase	397	345	356	369
rpoB	KFAV_v1_0154	RNA polymerase (beta subunit)	395	344	352	368
sufS	KFAV_v1_1040	cysteine desulfurase	395	344	352	367
KFAV_v1_2332	KFAV_v1_2332	conserved protein of unknown function	391	344	351	366
tsf	KFAV_v1_1881	Elongation factor Ts	391	343	350	364
psd	KFAV_v1_2354	Phosphatidylserine decarboxylase proenzyme	390	343	350	364
mdeA	KFAV_v1_1241	L-methionine gamma-lyase	390	342	349	364
KFAV_v1_3369	KFAV_v1_3369	Long-chain-fatty-acid--CoA ligase	389	341	349	363
recF	KFAV_v1_0005	RecA filament-DNA complex stabilisation, ssDNA and dsDNA binding, ATP binding	389	341	349	363
KFAV_v1_0909	KFAV_v1_0909	Glycosyl transferase family 2	389	340	349	362
adh_1	KFAV_v1_0360	Alcohol dehydrogenase	389	340	349	357
purC	KFAV_v1_0312	phosphoribosylaminoimidazole succinocarboxamide synthetase	388	339	348	357
lipM	KFAV_v1_1653	protein octanoyltransferase	388	338	347	357
citA	KFAV_v1_2602	citrate synthase I	387	337	346	356
KFAV_v1_0054	KFAV_v1_0054	AbrB/MazE/SpoVT family DNA-binding domain-containing protein	385	337	344	356
pyrB	KFAV_v1_2055	Aspartate carbamoyltransferase	385	336	344	355
serC	KFAV_v1_2383	phosphoserine aminotransferase	384	336	343	355
efp	KFAV_v1_1201	elongation factor P	383	336	342	353
bioD	KFAV_v1_2811	ATP-dependent dethiobiotin synthetase BioD	382	335	340	353
secG	KFAV_v1_0611	preprotein translocase subunit	380	334	340	352
dxs	KFAV_v1_1224	1-deoxyxylulose-5-phosphate synthase	380	334	339	351
queF	KFAV_v1_2574	NADPH-dependent 7-cyano-7-deazaguanine reductase	380	333	339	350
KFAV_v1_2398	KFAV_v1_2398	protein of unknown function	379	333	339	348
acs_2	KFAV_v1_1065	acetyl-CoA synthetase	377	333	337	346
hprT	KFAV_v1_0102	hypoxanthine-guanine phosphoribosyltransferase	375	332	337	345
KFAV_v1_1905	KFAV_v1_1905	conserved protein of unknown function	375	332	336	344
mreC	KFAV_v1_1154	Cell shape-determining protein MreC	374	332	334	344
KFAV_v1_0309	KFAV_v1_0309	protein of unknown function	372	331	334	343
KFAV_v1_1435	KFAV_v1_1435	conserved protein of unknown function	372	330	333	343
lutP	KFAV_v1_2864	L-lactate permease	371	329	332	343
sufD	KFAV_v1_1039	Fe-S cluster assembly protein SufD	369	329	331	341
KFAV_v1_1507	KFAV_v1_1507	Peptidase S16	369	328	330	341
KFAV_v1_3434	KFAV_v1_3434	protein of unknown function	368	325	330	341
spoVFA	KFAV_v1_1851	spore dipicolinate synthase subunit A	368	323	330	340
KFAV_v1_1705	KFAV_v1_1705	IDEAL domain protein (fragment)	367	323	329	339
argB	KFAV_v1_0490	N-acetylglutamate 5-phosphotransferase (acetylglutamate kinase)	365	322	328	339
sigH	KFAV_v1_0146	RNA polymerase sigma-30 factor (sigma(H))	364	321	328	335
sucC	KFAV_v1_1928	succinyl-CoA synthetase (beta subunit)	364	319	327	334
KFAV_v1_1033	KFAV_v1_1033	Group-specific protein	364	318	326	333
KFAV_v1_2536	KFAV_v1_2536	Thioesterase	364	315	326	332
novJ	KFAV_v1_0795	Short-chain reductase protein NovJ	364	315	323	332
KFAV_v1_1505	KFAV_v1_1505	conserved protein of unknown function	363	315	323	332
KFAV_v1_3413	KFAV_v1_3413	transposase	362	314	322	332
mdh_1	KFAV_v1_2633	malate dehydrogenase (NAD-dependent)	361	314	322	331
acs_1	KFAV_v1_0685	acetyl-CoA synthetase	360	314	321	330
KFAV_v1_2902	KFAV_v1_2902	Sodium ABC transporter ATP-binding protein	359	314	321	330
KFAV_v1_2899	KFAV_v1_2899	Cytochrome c-type biogenesis protein	357	314	321	329
lysCA	KFAV_v1_1115	aspartokinase II alpha subunit (aa 1->408)	356	314	319	328

nuoB_2	KFAV_v1_3501	NADH-quinone oxidoreductase subunit B	356	313	318	327
KFAV_v1_1190	KFAV_v1_1190	DNA replication protein	356	312	317	327
aroA	KFAV_v1_1696	3-phosphoshikimate 1-carboxyvinyltransferase (5-enolpyruvoylshikimate-3-phosphate synthase)	355	311	316	327
KFAV_v1_2788	KFAV_v1_2788	Acetamidase	353	310	316	326
yugH	KFAV_v1_1174	putative aspartate aminotransferase	353	309	315	324
KFAV_v1_1191	KFAV_v1_1191	conserved protein of unknown function	352	309	314	324
KFAV_v1_2790	KFAV_v1_2790	Radical SAM domain protein	351	309	314	323
KFAV_v1_0918	KFAV_v1_0918	2-oxoacid:acceptor oxidoreductase subunit alpha	350	308	311	323
KFAV_v1_2639	KFAV_v1_2639	Glutamate decarboxylase	350	307	310	323
pckA	KFAV_v1_2659	phosphoenolpyruvate carboxykinase	350	307	310	322
KFAV_v1_3539	KFAV_v1_3539	conserved protein of unknown function	350	306	309	322
KFAV_v1_0750	KFAV_v1_0750	conserved membrane protein of unknown function	348	306	308	321
KFAV_v1_0288	KFAV_v1_0288	conserved exported protein of unknown function	346	306	304	321
KFAV_v1_2622	KFAV_v1_2622	conserved protein of unknown function	346	306	304	321
KFAV_v1_2342	KFAV_v1_2342	Sortase family protein	345	305	304	320
rpmI	KFAV_v1_2528	ribosomal protein L35	345	304	303	319
KFAV_v1_3284	KFAV_v1_3284	conserved protein of unknown function	345	303	303	319
KFAV_v1_0719	KFAV_v1_0719	conserved protein of unknown function	344	300	302	319
KFAV_v1_0682	KFAV_v1_0682	conserved exported protein of unknown function	344	300	302	317
atpC	KFAV_v1_3503	ATP synthase (subunit epsilon, F1 subunit)	344	300	302	317
KFAV_v1_0887	KFAV_v1_0887	conserved protein of unknown function	343	299	301	316
trxBB_1	KFAV_v1_1218	ferredoxin-NADP+ reductase (flavodoxin)	342	297	300	315
KFAV_v1_0748	KFAV_v1_0748	conserved protein of unknown function	341	297	300	315
KFAV_v1_0118	KFAV_v1_0118	PaaI family thioesterase	341	297	299	315
narL	KFAV_v1_1413	DNA-binding response regulator in two-component regulatory system with NarX (or NarQ)	340	297	299	315
tilS	KFAV_v1_0101	tRNA(Ile)-lysidine synthase	340	296	298	313
sdhB	KFAV_v1_1120	succinate dehydrogenase (iron-sulfur protein)	339	295	298	313
hom	KFAV_v1_2499	Homoserine dehydrogenase	339	295	297	313
menG	KFAV_v1_1660	demethylmenaquinone methyltransferase	338	295	297	312
KFAV_v1_3238	KFAV_v1_3238	Flagellar biosynthesis protein FlgL	337	295	297	311
KFAV_v1_0794	KFAV_v1_0794	MaoC family dehydratase	336	294	297	311
KFAV_v1_3301	KFAV_v1_3301	Plasmid stabilization system	336	294	295	311
truA	KFAV_v1_0193	tRNA pseudouridine (38-40) synthase	335	294	295	311
spo0A	KFAV_v1_1230	response regulator, phosphorylated in response to complex YlbF/YmcA/YaaT	334	293	295	310
KFAV_v1_2216	KFAV_v1_2216	Transcriptional regulator containing PAS, AAA-type ATPase, and DNA-binding Fis domains	334	293	294	310
KFAV_v1_0232	KFAV_v1_0232	conserved protein of unknown function	333	293	294	310
yuiD	KFAV_v1_0930	Uncharacterized membrane protein YuiD	333	293	293	309
flgE	KFAV_v1_1906	flagellar hook protein	333	291	292	308
KFAV_v1_2941	KFAV_v1_2941	conserved protein of unknown function	332	290	292	307
KFAV_v1_1214	KFAV_v1_1214	conserved protein of unknown function	332	290	292	307
KFAV_v1_0301	KFAV_v1_0301	Cytochrome c class I	332	289	291	307
hemB	KFAV_v1_1136	delta-aminolevulinic acid dehydratase (porphobilinogen synthase)	331	289	291	307
pnpA	KFAV_v1_1860	polynucleotide phosphorylase (PNPase)	331	289	291	306
KFAV_v1_2102	KFAV_v1_2102	protein of unknown function	330	289	291	306
pyrP	KFAV_v1_2056	uracil permease	330	288	290	305
ffh	KFAV_v1_1967	signal recognition particle-like (SRP) GTPase	330	288	290	304
murAA_2	KFAV_v1_3487	UDP-N-acetylglucosamine 1-carboxyvinyltransferase	329	288	288	304
KFAV_v1_3066	KFAV_v1_3066	Undecaprenyl-phosphate alpha-N-acetylglucosaminyl 1-phosphate transferase	328	287	287	303
asd	KFAV_v1_1849	aspartate-semialdehyde dehydrogenase	327	286	287	303
fur_1	KFAV_v1_2073	transcriptional regulator for iron transport and metabolism	327	286	287	302
ylxM	KFAV_v1_1968	component of the signal recognition particle (SRP) protein-targeting pathway	326	286	287	301
lepB	KFAV_v1_1962	putative signal peptidase I-1	326	285	286	301

dapG	KFAV_v1_1848	aspartokinase I (alpha and beta subunits)	326	285	285	301
maeB	KFAV_v1_2640	NADP-dependent malic enzyme (conversion of malate into pyruvate, anabolic)	324	285	283	301
trpS	KFAV_v1_1105	tryptophanyl-tRNA synthetase	323	285	283	300
KFAV_v1_0014	KFAV_v1_0014	D-alanyl-D-alanine carboxypeptidase	323	283	283	300
KFAV_v1_0081	KFAV_v1_0081	Polysaccharide biosynthesis protein	322	282	283	299
KFAV_v1_0726	KFAV_v1_0726	protein of unknown function	321	281	283	298
pstC_1	KFAV_v1_2752	phosphate transporter subunit ; membrane component of ABC superfamily	321	281	283	297
glnA_1	KFAV_v1_0899	glutamine synthetase	320	281	282	297
glyA	KFAV_v1_3516	serine hydroxymethyltransferase	319	281	282	297
pyrG	KFAV_v1_3551	CTP synthetase	318	280	281	296
KFAV_v1_1941	KFAV_v1_1941	Transcription repressor NadR	317	279	280	295
KFAV_v1_2619	KFAV_v1_2619	protein of unknown function	317	279	280	294
KFAV_v1_1779	KFAV_v1_1779	Branched-chain amino acid ABC transporter	317	279	279	294
nuoH_2	KFAV_v1_3498	NADH:ubiquinone oxidoreductase, membrane subunit H	316	278	277	294
KFAV_v1_2392	KFAV_v1_2392	putative enzyme	315	278	276	292
lysS	KFAV_v1_0117	lysyl-tRNA synthetase	315	278	276	291
purU	KFAV_v1_0706	formyltetrahydrofolate hydrolase	315	278	275	291
KFAV_v1_1508	KFAV_v1_1508	conserved protein of unknown function	314	277	274	291
KFAV_v1_3225	KFAV_v1_3225	conserved protein of unknown function	313	277	274	290
KFAV_v1_1918	KFAV_v1_1918	Flagellar basal body rod protein FlgB	312	276	272	290
purB	KFAV_v1_0311	adenylosuccinate lyase	311	276	272	290
nadA	KFAV_v1_1940	quinolinate synthetase	311	276	272	289
KFAV_v1_2805	KFAV_v1_2805	conserved protein of unknown function	311	275	272	289
fbaA_2	KFAV_v1_3548	fructose-1,6-bisphosphate aldolase	311	275	272	289
pdhC_2	KFAV_v1_1095	pyruvate dehydrogenase (dihydrolipoamide acetyltransferase E2 subunit)	311	274	271	289
KFAV_v1_2863	KFAV_v1_2863	IclR family transcriptional regulator	310	274	271	287
KFAV_v1_2809	KFAV_v1_2809	conserved membrane protein of unknown function	310	273	270	287
hemL	KFAV_v1_1138	glutamate-1-semialdehyde 2,1-aminomutase	310	272	270	286
murI	KFAV_v1_1934	Glutamate racemase	310	271	270	286
KFAV_v1_0828	KFAV_v1_0828	protein of unknown function	310	271	269	284
cinA	KFAV_v1_1811	competence-damage inducible regulator	309	268	269	284
sufU	KFAV_v1_1041	iron-sulfur cluster assembly sulfur-transfer protein [Zn(2+)-dependent]	309	268	269	284
KFAV_v1_2219	KFAV_v1_2219	PaaI family thioesterase	308	266	269	284
dxr	KFAV_v1_1876	1-deoxy-D-xylulose-5-phosphate reductoisomerase	307	266	269	283
KFAV_v1_1031	KFAV_v1_1031	conserved exported protein of unknown function	307	265	269	282
fabD	KFAV_v1_1978	malonyl CoA:acyl carrier protein transacylase	306	265	267	280
KFAV_v1_2594	KFAV_v1_2594	conserved protein of unknown function	306	264	267	280
KFAV_v1_0493	KFAV_v1_0493	protein of unknown function	306	264	267	280
carAc	KFAV_v1_1043	Ferredoxin CarAc	304	264	267	279
KFAV_v1_3015	KFAV_v1_3015	conserved exported protein of unknown function	302	264	266	278
KFAV_v1_0284	KFAV_v1_0284	Aldo/keto reductase	302	263	266	278
KFAV_v1_2016	KFAV_v1_2016	conserved exported protein of unknown function	302	261	266	278
ldeF	KFAV_v1_0716	methylglutaconyl-CoA hydratase (leucine degradation)	301	261	265	278
KFAV_v1_0082	KFAV_v1_0082	Nucleoside triphosphate pyrophosphohydrolase	301	261	265	276
KFAV_v1_2817	KFAV_v1_2817	conserved membrane protein of unknown function	301	260	265	276
KFAV_v1_2355	KFAV_v1_2355	conserved protein of unknown function	300	260	264	276
nadK	KFAV_v1_1226	NAD kinase	300	260	264	276
KFAV_v1_3308	KFAV_v1_3308	3-hydroxybutyryl-CoA dehydratase	299	260	264	275
serA	KFAV_v1_1606	D-3-phosphoglycerate dehydrogenase	299	259	263	274
KFAV_v1_3446	KFAV_v1_3446	protein of unknown function	298	259	262	274
mrdA	KFAV_v1_1957	Penicillin-binding protein 2	298	258	262	273
prfA	KFAV_v1_3535	peptide chain release factor 1	298	257	262	272

ydcM_3	KFAV_v1_3013	putative transposase	297	257	261	272
KFAV_v1_0821	KFAV_v1_0821	protein of unknown function	296	255	260	271
resD	KFAV_v1_1597	two-component response regulator (ResD / ResE)	296	254	260	271
pstS	KFAV_v1_2758	Phosphate-binding protein PstS	294	253	260	270
KFAV_v1_1273	KFAV_v1_1273	protein of unknown function	294	253	259	269
accD	KFAV_v1_2638	acetyl-CoA carboxylase (carboxyltransferase beta subunit)	292	253	259	269
purR	KFAV_v1_0070	transcriptional regulator of the purine biosynthesis operon (PurR-pRpp)	291	253	259	268
KFAV_v1_3429	KFAV_v1_3429	conserved exported protein of unknown function	291	252	259	268
cydA_1	KFAV_v1_0816	cytochrome bb' ubiquinol oxidase (subunit I)	290	251	258	267
ctaB	KFAV_v1_0751	protoheme IX farnesyltransferase 2	290	251	257	267
tyrA	KFAV_v1_1695	prephenate dehydrogenase	289	251	256	267
KFAV_v1_0657	KFAV_v1_0657	Transcriptional regulator, XRE family	289	250	256	267
KFAV_v1_0941	KFAV_v1_0941	protein of unknown function	289	250	256	266
yrbC	KFAV_v1_2419	putative factor regulating gene expression	287	248	253	265
KFAV_v1_0721	KFAV_v1_0721	conserved protein of unknown function	287	248	252	265
KFAV_v1_2791	KFAV_v1_2791	Heme d1 biosynthesis radical SAM protein NirJ1	286	247	252	265
KFAV_v1_0715	KFAV_v1_0715	methylcrotonyl-CoA carboxylase; biotinylated subunit (leucine degradation) (modular protein)	285	247	251	264
groES	KFAV_v1_0242	chaperonin small subunit	284	245	251	264
KFAV_v1_0797	KFAV_v1_0797	Phosphotriesterase-related protein	283	245	251	263
KFAV_v1_1670	KFAV_v1_1670	Copper amine oxidase domain protein	283	245	250	262
eno	KFAV_v1_0610	enolase	283	245	249	262
nuoD_2	KFAV_v1_3499	NADH-quinone oxidoreductase subunit D	283	244	248	262
KFAV_v1_1278	KFAV_v1_1278	transposase	283	244	248	260
murAA_1	KFAV_v1_1551	UDP-N-acetylglucosamine 1-carboxyvinyltransferase	282	244	247	260
KFAV_v1_1830	KFAV_v1_1830	Transcriptional regulator, PadR-like family	282	244	247	260
KFAV_v1_0434	KFAV_v1_0434	Regulatory protein AtoC (fragment)	282	243	247	259
KFAV_v1_0740	KFAV_v1_0740	conserved protein of unknown function	281	243	247	258
KFAV_v1_0197	KFAV_v1_0197	conserved protein of unknown function	281	242	246	258
KFAV_v1_1630	KFAV_v1_1630	NDP-hexose 2,3-dehydratase	281	242	246	257
icd	KFAV_v1_2634	isocitrate dehydrogenase	280	241	246	257
yqxC	KFAV_v1_1225	putative 2'-O-ribose RNA methyltransferase	279	241	245	257
folK	KFAV_v1_0112	7,8-dihydro-6-hydroxymethylpterin pyrophosphokinase	278	241	245	257
KFAV_v1_1829	KFAV_v1_1829	conserved protein of unknown function	278	241	245	256
aspC	KFAV_v1_3565	Aspartate aminotransferase	277	241	244	255
fabI	KFAV_v1_2326	enoyl-acyl carrier protein reductase	276	241	244	254
proB	KFAV_v1_2498	gamma-glutamate kinase	276	241	244	253
ctsR	KFAV_v1_0131	transcriptional regulator of class III stress genes	275	241	244	253
KFAV_v1_0588	KFAV_v1_0588	conserved protein of unknown function	275	241	242	252
KFAV_v1_1116	KFAV_v1_1116	conserved protein of unknown function	275	239	242	252
KFAV_v1_1657	KFAV_v1_1657	conserved protein of unknown function	275	239	241	252
spoVFB	KFAV_v1_1850	spore dipicolinate synthase subunit B	274	238	241	252
KFAV_v1_0585	KFAV_v1_0585	4-hydroxybutyrate--CoA ligase 2	273	238	240	252
KFAV_v1_3300	KFAV_v1_3300	conserved protein of unknown function	272	238	240	251
ilvE_1	KFAV_v1_0261	putative branched-chain-amino-acid aminotransferase	272	237	239	250
fin	KFAV_v1_0077	protein required for the switch from F to G during sporulation (anti sigma F)	272	236	239	250
KFAV_v1_0344	KFAV_v1_0344	conserved protein of unknown function	270	236	238	249
yutF	KFAV_v1_0933	Acid sugar phosphatase	270	236	238	249
zapA	KFAV_v1_2520	regulator of cell division	270	235	238	249
KFAV_v1_2946	KFAV_v1_2946	conserved protein of unknown function	270	235	238	247
salA	KFAV_v1_0196	phosphorylation-dependent (Y327) transcriptional regulator	269	235	237	247
KFAV_v1_3411	KFAV_v1_3411	conserved protein of unknown function	269	234	237	247
trpC	KFAV_v1_1691	Indole-3-glycerol phosphate synthase	268	234	237	244

KFAV_v1_1488	KFAV_v1_1488	Glutamine amidotransferase	268	233	237	244
KFAV_v1_3299	KFAV_v1_3299	AMP-dependent synthetase	267	232	236	243
ridA	KFAV_v1_0071	aminoacrylate/iminopropionate hydrolase/deaminase	267	232	236	242
cobA	KFAV_v1_1135	Uroporphyrinogen-III C-methyltransferase	267	232	236	241
KFAV_v1_0742	KFAV_v1_0742	TlpA family protein disulfide reductase	266	231	236	241
citB	KFAV_v1_2761	aconitate hydratase	266	231	235	241
KFAV_v1_0793	KFAV_v1_0793	Dehydratase	265	231	233	241
aroB	KFAV_v1_1689	3-dehydroquinate synthase	265	231	233	241
valS	KFAV_v1_1144	valyl-tRNA synthetase	264	230	233	241
etfB	KFAV_v1_0857	electron transfer flavoprotein (beta subunit)	263	230	233	241
cydB	KFAV_v1_0817	Cytochrome d ubiquinol oxidase subunit II	263	230	233	241
smpB_1	KFAV_v1_0614	fragment of tmRNA-binding protein (part 1)	263	229	233	241
KFAV_v1_1493	KFAV_v1_1493	exported protein of unknown function	262	229	232	240
KFAV_v1_2546	KFAV_v1_2546	Integrase catalytic region	262	228	231	240
glnR	KFAV_v1_1769	transcriptional regulator (nitrogen metabolism)	261	228	230	240
KFAV_v1_1137	KFAV_v1_1137	Lrp/AsnC family transcriptional regulator	261	227	230	240
pdhA_2	KFAV_v1_1092	pyruvate dehydrogenase (E1 alpha subunit)	261	226	230	239
fliF	KFAV_v1_1915	Flagellar M-ring protein	259	226	230	239
KFAV_v1_1565	KFAV_v1_1565	conserved protein of unknown function	259	225	230	239
KFAV_v1_1046	KFAV_v1_1046	Phosphatase	259	225	230	237
lepA	KFAV_v1_2147	ribosomal elongation factor, GTPase	259	225	229	237
KFAV_v1_1659	KFAV_v1_1659	conserved protein of unknown function	258	225	229	237
der	KFAV_v1_1649	GTPase essential for ribosome 50S subunit assembly (maturation of the 50S subunit central protoberance)	258	224	228	236
KFAV_v1_1114	KFAV_v1_1114	conserved membrane protein of unknown function	257	224	228	236
rarA	KFAV_v1_2296	DNA-dependent ATPase active at replication forks	255	224	227	235
KFAV_v1_2488	KFAV_v1_2488	Decaprenyl-phosphate phosphoribosyltransferase	255	223	226	234
KFAV_v1_1617	KFAV_v1_1617	protein of unknown function	255	222	226	234
KFAV_v1_1159	KFAV_v1_1159	Rod shape-determining protein RodA	254	222	226	234
bshA	KFAV_v1_3404	N-acetyl-alpha-D-glucosaminyl L-malate synthase	254	221	226	233
KFAV_v1_1929	KFAV_v1_1929	conserved protein of unknown function	254	221	226	233
pyrE	KFAV_v1_2576	orotate phosphoribosyltransferase	254	220	226	232
KFAV_v1_2729	KFAV_v1_2729	conserved exported protein of unknown function	254	220	224	232
leuS	KFAV_v1_2162	leucyl-tRNA synthetase	253	220	224	231
KFAV_v1_2771	KFAV_v1_2771	conserved protein of unknown function	253	219	224	231
KFAV_v1_0422	KFAV_v1_0422	protein of unknown function	253	218	224	230
dapA_2	KFAV_v1_1847	4-hydroxy-tetrahydrodipicolinate synthase	252	218	223	230
rasP	KFAV_v1_1875	Zinc metalloprotease RasP	252	218	223	230
recA	KFAV_v1_1809	multifunctional SOS repair factor	252	218	222	229
gabD	KFAV_v1_0792	succinate-semialdehyde dehydrogenase	251	216	222	229
rocG_1	KFAV_v1_1388	glutamate dehydrogenase	251	216	222	229
ruvB	KFAV_v1_2415	Holliday junction DNA helicase, ATP-dependent component	251	215	221	228
KFAV_v1_1988	KFAV_v1_1988	conserved protein of unknown function	251	214	220	228
KFAV_v1_0755	KFAV_v1_0755	Topoisomerase	250	214	219	228
clpP_2	KFAV_v1_1511	ATP-dependent Clp protease proteolytic subunit; Maxwell's demon	249	214	219	228
KFAV_v1_3237	KFAV_v1_3237	conserved protein of unknown function	249	214	219	227
purS	KFAV_v1_0313	factor required for phosphoribosylformylglycinamidine synthetase activity	249	214	218	227
rnc	KFAV_v1_1974	ribonuclease III	249	214	218	227
murC	KFAV_v1_1146	UDP-N-acetylmuramate--L-alanine ligase	249	213	217	226
KFAV_v1_2082	KFAV_v1_2082	conserved protein of unknown function	249	213	217	226
KFAV_v1_0785	KFAV_v1_0785	Fis family transcriptional regulator	248	212	217	226
proS	KFAV_v1_1873	prolyl-tRNA synthetase	248	212	216	225
KFAV_v1_3436	KFAV_v1_3436	conserved protein of unknown function	247	212	216	225

KFAV_v1_1904	KFAV_v1_1904	Flagellar protein FliL	247	211	216	224
KFAV_v1_1854	KFAV_v1_1854	5'(3')-deoxyribonucleotidase	247	211	216	224
rocG_2	KFAV_v1_2577	glutamate dehydrogenase	247	211	214	224
yitK	KFAV_v1_1814	putative RNA or cyclic d-GMP binding protein	247	211	213	224
tyrZ	KFAV_v1_2516	tyrosyl-tRNA synthetase	247	211	213	224
trpA	KFAV_v1_1694	tryptophan synthase (alpha subunit)	246	210	213	224
KFAV_v1_2597	KFAV_v1_2597	AAA+ family ATPase	245	210	213	224
KFAV_v1_3088	KFAV_v1_3088	Flagellar hook-associated protein 2	245	210	212	223
hisJ	KFAV_v1_3083	putative histidine-binding protein	244	210	209	222
KFAV_v1_0361	KFAV_v1_0361	conserved exported protein of unknown function	244	210	209	222
KFAV_v1_3281	KFAV_v1_3281	Transcriptional regulator	244	209	209	222
lgt	KFAV_v1_0590	Prolipoprotein diacylglyceryl transferase	244	209	209	221
KFAV_v1_2818	KFAV_v1_2818	Sugar phosphate permease	242	207	208	221
smpB_2	KFAV_v1_0615	fragment of tmRNA-binding protein (part 2)	241	207	208	221
rbfA	KFAV_v1_1865	pre-ribosomal (17S) RNA binding factor A	241	207	208	221
acoR	KFAV_v1_2223	transcriptional regulator (AcoR-acetoin)	241	207	207	221
guaA	KFAV_v1_0292	GMP synthetase	239	207	207	221
divIVA	KFAV_v1_1567	cell-division initiation protein	239	206	207	220
KFAV_v1_2537	KFAV_v1_2537	protein of unknown function	239	205	206	220
fliS	KFAV_v1_3087	Flagellar secretion chaperone FliS	239	204	206	219
KFAV_v1_2643	KFAV_v1_2643	conserved protein of unknown function	239	204	206	219
KFAV_v1_1582	KFAV_v1_1582	conserved exported protein of unknown function	238	204	205	219
KFAV_v1_0931	KFAV_v1_0931	conserved protein of unknown function	238	204	204	219
KFAV_v1_3306	KFAV_v1_3306	Transcriptional regulator, AbrB family	238	204	204	219
acsA_1	KFAV_v1_2255	Acetoacetyl-coenzyme A synthetase	238	203	204	218
pbuG	KFAV_v1_0293	hypoxanthine/guanine permease	238	202	203	218
ytzG	KFAV_v1_2601	Uncharacterized RNA pseudouridine synthase YtzG	238	202	203	218
livF_2	KFAV_v1_1697	leucine/isoleucine/valine transporter subunit ; ATP-binding component of ABC superfamily	238	202	202	217
purQ	KFAV_v1_0314	phosphoribosylformylglycinamidine synthetase subunit I	238	202	202	217
yloU	KFAV_v1_1989	putative factor involved in malonyl-CoA synthesis	237	202	201	217
ccdA	KFAV_v1_0743	Cytochrome c-type biogenesis protein	237	201	201	217
KFAV_v1_2942	KFAV_v1_2942	conserved protein of unknown function	236	201	201	217
KFAV_v1_0393	KFAV_v1_0393	putative Glycosyltransferase	236	201	201	217
etfA_1	KFAV_v1_0858	electron transfer flavoprotein (alpha subunit)	236	201	201	217
KFAV_v1_2428	KFAV_v1_2428	transposase (fragment)	235	200	198	217
yugG	KFAV_v1_1175	putative transcriptional regulator (Lrp/AsnC family)	235	200	198	216
KFAV_v1_3296	KFAV_v1_3296	Branched-chain amino acid ABC transporter permease	235	200	198	216
aspS	KFAV_v1_2299	aspartyl-tRNA synthetase, promiscuous (also recognizes tRNAasn)	235	199	198	215
clpC_1	KFAV_v1_0134	class III stress response-related ATPase, AAA+ superfamily	234	199	198	214
hemC	KFAV_v1_1134	porphobilinogen deaminase (hydroxymethylbilane synthase)	233	198	198	214
KFAV_v1_2159	KFAV_v1_2159	conserved membrane protein of unknown function	233	198	198	213
acuA	KFAV_v1_1064	protein acetyltransferase (acetylates AcsA)	233	197	198	212
KFAV_v1_0654	KFAV_v1_0654	transposase	233	197	197	211
pgsA_2	KFAV_v1_3477	CDP-diacylglycerol--glycerol-3-phosphate 3-phosphatidyltransferase	232	197	197	211
acs_4	KFAV_v1_1272	Acetyl-coenzyme A synthetase	232	197	197	211
KFAV_v1_2421	KFAV_v1_2421	Acyltransferase	232	197	196	211
nphA	KFAV_v1_2423	NADH-dependent flavin reductase	232	196	196	211
fliM	KFAV_v1_1903	flagellar motor switching and energizing component	231	196	196	211
KFAV_v1_3245	KFAV_v1_3245	conserved protein of unknown function	231	196	196	211
KFAV_v1_2010	KFAV_v1_2010	conserved protein of unknown function	231	196	196	210
KFAV_v1_2122	KFAV_v1_2122	conserved protein of unknown function	230	196	195	210
KFAV_v1_0355	KFAV_v1_0355	conserved protein of unknown function	230	195	195	210

KFAV_v1_2367	KFAV_v1_2367	conserved protein of unknown function	228	195	194	210
holB	KFAV_v1_0050	DNA polymerase III subunit delta	227	195	194	210
KFAV_v1_2860	KFAV_v1_2860	conserved protein of unknown function	227	195	194	210
KFAV_v1_3328	KFAV_v1_3328	conserved protein of unknown function	227	195	194	210
KFAV_v1_3266	KFAV_v1_3266	conserved exported protein of unknown function	226	194	194	209
KFAV_v1_2381	KFAV_v1_2381	conserved protein of unknown function	225	194	193	208
engD	KFAV_v1_3597	potassium-dependent informational ATPase interacting with 70S ribosome; ROS stress regulator	225	194	193	208
nuoK_2	KFAV_v1_3495	NADH-quinone oxidoreductase subunit K	224	194	193	208
KFAV_v1_2515	KFAV_v1_2515	Heat-shock protein Hsp20	224	194	193	208
KFAV_v1_1864	KFAV_v1_1864	Phosphoesterase RecJ domain protein	224	193	192	208
spcB	KFAV_v1_1590	chromosome condensation and segregation factor	223	193	192	207
KFAV_v1_1049	KFAV_v1_1049	3-hydroxypropionyl-coenzyme A dehydratase	221	193	192	206
walJ	KFAV_v1_3580	putative hydrolase	221	193	192	206
dusB	KFAV_v1_0114	tRNA-dihydrouridine synthase B	220	193	192	206
upp	KFAV_v1_3515	uracil phosphoribosyltransferase	220	193	191	206
ileS	KFAV_v1_1569	isoleucyl-tRNA synthetase	220	193	191	206
acs_3	KFAV_v1_1113	acetyl-CoA synthetase	219	192	191	206
KFAV_v1_0658	KFAV_v1_0658	HEPN domain protein (modular protein)	219	192	191	205
KFAV_v1_0720	KFAV_v1_0720	conserved protein of unknown function	218	192	191	205
KFAV_v1_2329	KFAV_v1_2329	conserved protein of unknown function	218	191	190	205
argJ	KFAV_v1_0489	ornithine acetyltransferase; amino-acid acetyltransferase	218	191	190	205
KFAV_v1_1057	KFAV_v1_1057	Lytic transglycosylase catalytic	218	191	190	205
pyrD_2	KFAV_v1_2051	dihydroorotate dehydrogenase (catalytic subunit)	217	191	189	205
yccU	KFAV_v1_1602	putative CoA-binding protein	217	191	189	205
KFAV_v1_3151	KFAV_v1_3151	Copper amine oxidase	217	191	189	204
nocA	KFAV_v1_3608	DNA-binding protein Spo0J-like	216	190	189	203
KFAV_v1_0674	KFAV_v1_0674	conserved protein of unknown function	216	190	188	203
phoH	KFAV_v1_1256	phosphate starvation-induced protein	215	190	187	203
KFAV_v1_3268	KFAV_v1_3268	conserved exported protein of unknown function	215	190	187	202
KFAV_v1_0497	KFAV_v1_0497	conserved protein of unknown function	214	190	187	201
KFAV_v1_3595	KFAV_v1_3595	conserved protein of unknown function	214	190	187	201
parB	KFAV_v1_3606	site-specific DNA-binding protein	214	189	186	201
livG_3	KFAV_v1_3297	leucine/isoleucine/valine transporter subunit ; ATP-binding component of ABC superfamily	212	189	186	201
KFAV_v1_0097	KFAV_v1_0097	CMP deaminase	212	189	185	201
KFAV_v1_0586	KFAV_v1_0586	Class I SAM-dependent methyltransferase	211	189	185	200
accA	KFAV_v1_2637	acetyl-CoA carboxylase (carboxyltransferase alpha subunit)	211	188	185	200
argD	KFAV_v1_0492	N-acetylornithine aminotransferase	211	188	185	200
KFAV_v1_2214	KFAV_v1_2214	putative cyclase	211	187	185	200
pfkA	KFAV_v1_2636	6-phosphofructokinase	211	187	185	200
KFAV_v1_2291	KFAV_v1_2291	transposase	210	187	184	199
KFAV_v1_0392	KFAV_v1_0392	putative Glycosyltransferase	210	186	184	199
KFAV_v1_2944	KFAV_v1_2944	conserved protein of unknown function	210	186	184	199
xseA	KFAV_v1_1220	exodeoxyribonuclease VII (large subunit)	209	186	184	199
pth	KFAV_v1_0076	peptidyl-tRNA hydrolase	208	186	184	198
KFAV_v1_2391	KFAV_v1_2391	Carboxymethylenebutenolidase	208	185	184	198
KFAV_v1_0628	KFAV_v1_0628	DNA binding domain protein, excisionase family	208	185	184	198
namA	KFAV_v1_1167	NADPH dehydrogenase	208	185	183	197
infB	KFAV_v1_1866	initiation factor IF-2	208	185	182	197
queC	KFAV_v1_0888	7-cyano-7-deazaguanine synthase	207	185	182	197
yqfO	KFAV_v1_1275	putative GTP cyclohydrolase	207	184	182	197
trpF	KFAV_v1_1692	N-(5'-phosphoribosyl)anthranilate isomerase	207	184	182	196
KFAV_v1_1669	KFAV_v1_1669	conserved protein of unknown function	206	183	182	196

KFAV_v1_1672	KFAV_v1_1672	Nuclease	206	183	181	195
KFAV_v1_0898	KFAV_v1_0898	conserved protein of unknown function	206	183	181	195
KFAV_v1_1616	KFAV_v1_1616	Sirohydrochlorin cobaltochelatase	206	183	181	195
lipA	KFAV_v1_2083	lipoyl synthase (lipoic acid synthetase)	206	183	181	195
nuoA_2	KFAV_v1_3502	NADH-quinone oxidoreductase subunit A	205	183	181	194
KFAV_v1_0625	KFAV_v1_0625	protein of unknown function	205	183	181	194
KFAV_v1_1235	KFAV_v1_1235	conserved protein of unknown function	205	182	180	194
rpmJ	KFAV_v1_0187	ribosomal protein L36 (ribosomal protein B)	203	182	180	194
KFAV_v1_2948	KFAV_v1_2948	conserved protein of unknown function	203	182	179	194
KFAV_v1_3593	KFAV_v1_3593	protein of unknown function	203	182	179	194
rnpA	KFAV_v1_3614	Ribonuclease P protein component	203	182	179	193
spoIVB	KFAV_v1_1229	regulatory membrane-associated serine protease	203	182	179	193
rho	KFAV_v1_3541	transcriptional terminator Rho	202	182	178	193
moeB	KFAV_v1_2092	Molybdopterin-synthase adenylyltransferase	202	181	178	192
fadN	KFAV_v1_2320	bifunctional enoyl-CoA hydratase / 3-hydroxyacyl-CoA dehydrogenase	202	181	178	192
mtnA	KFAV_v1_1727	Methylthioribose-1-phosphate isomerase	202	181	177	192
KFAV_v1_2556	KFAV_v1_2556	Transcriptional regulator	201	181	177	192
KFAV_v1_2218	KFAV_v1_2218	protein of unknown function	201	181	177	192
KFAV_v1_2903	KFAV_v1_2903	conserved membrane protein of unknown function	200	181	176	191
KFAV_v1_3273	KFAV_v1_3273	conserved protein of unknown function	200	181	176	191
ywdK	KFAV_v1_1392	conserved membrane protein	200	181	176	191
KFAV_v1_2550	KFAV_v1_2550	conserved protein of unknown function	200	181	176	191
KFAV_v1_0059	KFAV_v1_0059	conserved protein of unknown function	199	181	176	191
spoVS	KFAV_v1_1805	regulator required for dehydratation of the spore core and assembly of the coat (stage V sporulation)	199	180	176	190
KFAV_v1_3277	KFAV_v1_3277	Thymidine kinase	199	180	176	190
KFAV_v1_2356	KFAV_v1_2356	Uracil-DNA glycosylase	199	180	176	190
KFAV_v1_0892	KFAV_v1_0892	conserved protein of unknown function	198	180	175	190
rsfS	KFAV_v1_2163	ribosomal silencing factor	198	180	175	189
ubiX	KFAV_v1_1661	Flavin prenyltransferase UbiX	197	179	175	189
metH	KFAV_v1_0746	Methionine synthase	197	179	175	189
KFAV_v1_1274	KFAV_v1_1274	conserved protein of unknown function	197	179	175	189
sdhA	KFAV_v1_1119	succinate dehydrogenase (flavoprotein subunit)	197	179	175	188
cymR	KFAV_v1_2295	transcriptional regulator of cysteine biosynthesis	197	179	174	188
KFAV_v1_1553	KFAV_v1_1553	conserved protein of unknown function	197	179	174	188
mtaD	KFAV_v1_1728	5-methylthioadenosine/S-adenosylhomocysteine deaminase	196	178	174	188
KFAV_v1_1973	KFAV_v1_1973	conserved protein of unknown function	196	178	173	188
KFAV_v1_0244	KFAV_v1_0244	protein of unknown function	196	178	173	187
KFAV_v1_0491	KFAV_v1_0491	conserved protein of unknown function	196	177	173	187
fliG	KFAV_v1_1914	flagellar motor switching and energizing component	195	177	172	187
papA	KFAV_v1_1200	aminopeptidase (Met-Xaa and Xaa-Pro, Xaa-Pro-Xaa)	195	177	172	186
dnaK	KFAV_v1_2143	molecular chaperone, ATP-dependent	194	176	172	186
KFAV_v1_1611	KFAV_v1_1611	Iron dicitrate transport regulator FecR	194	176	172	186
KFAV_v1_1908	KFAV_v1_1908	conserved protein of unknown function	194	176	172	186
KFAV_v1_0805	KFAV_v1_0805	Aminodeoxychorismate lyase	194	176	171	185
KFAV_v1_2945	KFAV_v1_2945	CRISPR-associated RAMP protein	194	175	171	185
greA_2	KFAV_v1_1419	transcription elongation factor resolving backtracking / stalling	194	175	171	185
KFAV_v1_2789	KFAV_v1_2789	Hemerythrin	194	175	171	185
hbd	KFAV_v1_2784	3-hydroxybutyryl-CoA dehydrogenase	193	175	171	185
xseB	KFAV_v1_1221	exodeoxyribonuclease VII (small subunit)	193	174	171	185
pssA	KFAV_v1_1395	CDP-diacylglycerol--serine O-phosphatidyltransferase	193	174	171	184
mqnC	KFAV_v1_1664	Cyclic dehypoxanthine futalosine synthase	193	174	170	184
ndk	KFAV_v1_1666	nucleoside diphosphate kinase	193	174	170	184

cheW	KFAV_v1_1890	modulation of CheA activity in response to attractants (chemotaxis)	193	173	170	184
KFAV_v1_2575	KFAV_v1_2575	protein of unknown function	193	173	170	184
KFAV_v1_2816	KFAV_v1_2816	conserved membrane protein of unknown function	193	173	170	183
ytjA	KFAV_v1_3613	membrane protein insertion efficiency factor	193	173	170	183
cat	KFAV_v1_0747	Succinyl-CoA:coenzyme A transferase	192	173	169	183
pycA	KFAV_v1_1519	pyruvate carboxylase	192	172	169	183
KFAV_v1_3484	KFAV_v1_3484	Chemotaxis protein	191	172	169	182
KFAV_v1_2290	KFAV_v1_2290	transposase	191	172	168	182
KFAV_v1_3488	KFAV_v1_3488	conserved exported protein of unknown function	191	172	168	182
KFAV_v1_3348	KFAV_v1_3348	conserved protein of unknown function	191	172	168	182
gatB	KFAV_v1_3063	glutamyl-tRNA(Gln) amidotransferase (subunit B)	190	172	168	182
KFAV_v1_0601	KFAV_v1_0601	conserved protein of unknown function	190	172	168	182
KFAV_v1_1601	KFAV_v1_1601	conserved protein of unknown function	190	171	168	182
ribF	KFAV_v1_1862	Riboflavin kinase / FMN adenylyltransferase	189	171	168	182
KFAV_v1_2947	KFAV_v1_2947	conserved protein of unknown function	189	171	167	181
KFAV_v1_2274	KFAV_v1_2274	conserved protein of unknown function	188	171	167	181
mqnE_2	KFAV_v1_1933	Aminodeoxyfutalosine synthase	188	171	167	181
KFAV_v1_3295	KFAV_v1_3295	conserved membrane protein of unknown function	187	171	167	181
tal	KFAV_v1_3547	transaldolase	187	170	167	181
KFAV_v1_0126	KFAV_v1_0126	transposase	186	170	167	181
KFAV_v1_1202	KFAV_v1_1202	conserved membrane protein of unknown function	186	170	166	180
KFAV_v1_2089	KFAV_v1_2089	conserved protein of unknown function	186	170	166	180
KFAV_v1_2327	KFAV_v1_2327	NUDIX hydrolase (fragment)	186	169	165	180
ebfC	KFAV_v1_0039	nucleoid associated protein	185	169	165	180
nuoC_2	KFAV_v1_3500	NADH-quinone oxidoreductase subunit C	185	169	165	180
parA	KFAV_v1_3607	chromosome partitioning protein; transcriptional regulator	185	169	165	180
KFAV_v1_0717	KFAV_v1_0717	Methyl-accepting chemotaxis protein	184	168	165	180
pucH	KFAV_v1_1332	allantoinase	184	168	165	180
fadH	KFAV_v1_2325	putative 2,4-dienoyl-CoA reductase	184	168	165	180
KFAV_v1_3421	KFAV_v1_3421	Multidrug ABC transporter	184	167	164	179
KFAV_v1_0772	KFAV_v1_0772	protein of unknown function	183	167	164	179
KFAV_v1_0950	KFAV_v1_0950	conserved protein of unknown function	183	167	164	178
fliI	KFAV_v1_1912	flagellar-specific ATPase subunit of export apparatus	183	166	164	178
recR	KFAV_v1_0040	recA filament-DNA complex stabilisation factor	183	166	163	178
KFAV_v1_1734	KFAV_v1_1734	ATPase	183	166	163	178
rimF	KFAV_v1_2275	pre-16S ribosomal RNA maturation enzyme	183	165	163	177
KFAV_v1_1994	KFAV_v1_1994	Protein serine/threonine phosphatase	183	164	163	177
KFAV_v1_1624	KFAV_v1_1624	Phosphohydrolase	183	164	163	177
KFAV_v1_2969	KFAV_v1_2969	CRISPR-associated protein	183	164	163	177
KFAV_v1_3553	KFAV_v1_3553	putative enzyme	183	163	162	176
rlbA	KFAV_v1_0004	RNA binding protein involved in ribosome maturation	182	163	162	176
KFAV_v1_0283	KFAV_v1_0283	Cupin	182	163	162	176
KFAV_v1_0982	KFAV_v1_0982	Amino acid ABC transporter substrate-binding protein, PAAT family	182	162	162	176
hisF	KFAV_v1_0576	imidazole glycerol phosphate synthase subunit	182	162	161	175
gmhB	KFAV_v1_0937	D-glycero-beta-D-manno-heptose-1,7-bisphosphate 7-phosphatase	182	161	161	174
fliP	KFAV_v1_1899	flagellar biosynthesis protein	182	161	161	173
KFAV_v1_1443	KFAV_v1_1443	conserved membrane protein of unknown function	181	161	160	173
pyrK	KFAV_v1_2052	dihydroorotate dehydrogenase (electron transfer subunit)	181	160	160	173
cheA	KFAV_v1_1891	chemotactic two-component sensor histidine kinase	181	160	160	173
KFAV_v1_1104	KFAV_v1_1104	Methylenetetrahydrofolate reductase	181	160	160	172
spoVT	KFAV_v1_0080	transcriptional regulator of sporulation / germination	181	160	160	172
KFAV_v1_0769	KFAV_v1_0769	conserved protein of unknown function	181	159	160	171

KFAV_v1_0414	KFAV_v1_0414	putative UDP-glucose 4-epimerase	181	159	160	171
KFAV_v1_2943	KFAV_v1_2943	conserved protein of unknown function	181	159	160	171
pheT	KFAV_v1_2521	phenylalanyl-tRNA synthetase (beta subunit)	180	158	160	170
KFAV_v1_1402	KFAV_v1_1402	conserved membrane protein of unknown function	180	158	160	170
KFAV_v1_1568	KFAV_v1_1568	protein of unknown function	179	158	160	170
KFAV_v1_0354	KFAV_v1_0354	conserved protein of unknown function	179	158	160	169
pilT	KFAV_v1_1147	Twitching mobility protein	179	158	159	169
recX	KFAV_v1_1808	Regulatory protein RecX	178	157	159	169
kduD	KFAV_v1_2785	2-dehydro-3-deoxy-D-gluconate 5-dehydrogenase	178	157	159	169
KFAV_v1_1907	KFAV_v1_1907	Flagellar hook capping protein	178	157	159	169
pstA_1	KFAV_v1_2751	phosphate transporter subunit ; membrane component of ABC superfamily	178	157	159	169
rnrY	KFAV_v1_1258	endonuclease involved in 70S ribosomes quality control	177	157	159	169
rsh	KFAV_v1_2394	GTP pyrophosphokinase (RelA/SpoT)	177	157	158	169
KFAV_v1_1591	KFAV_v1_1591	conserved protein of unknown function	176	157	158	168
KFAV_v1_1165	KFAV_v1_1165	conserved protein of unknown function	176	156	157	168
KFAV_v1_0796	KFAV_v1_0796	Long-chain fatty acid--CoA ligase	175	156	157	168
KFAV_v1_1032	KFAV_v1_1032	conserved membrane protein of unknown function	175	156	157	168
KFAV_v1_2505	KFAV_v1_2505	conserved protein of unknown function	175	156	157	168
KFAV_v1_3032	KFAV_v1_3032	DEAD/DEAH box helicase	175	156	157	168
hpaH	KFAV_v1_1845	4-hydroxyphenylacetate 3-monooxygenase oxygenase component	175	155	156	167
hrcA	KFAV_v1_2145	transcriptional regulator of heat-shock genes	175	155	156	167
ku	KFAV_v1_2158	Non-homologous end joining protein Ku	174	155	156	166
remA	KFAV_v1_2003	essential sporulation DNA binding protein; regulator of biofilm formation	173	155	155	166
glcD_2	KFAV_v1_2865	glycolate oxidase subunit, FAD-linked	173	154	155	166
KFAV_v1_0019	KFAV_v1_0019	conserved membrane protein of unknown function	173	154	155	164
KFAV_v1_0060	KFAV_v1_0060	conserved protein of unknown function	172	154	155	164
KFAV_v1_1045	KFAV_v1_1045	protein of unknown function	172	154	155	164
KFAV_v1_1735	KFAV_v1_1735	DNA replication protein DnaD	172	153	154	164
KFAV_v1_2401	KFAV_v1_2401	Amidohydrolase 2	172	153	154	163
KFAV_v1_1784	KFAV_v1_1784	Gamma-glutamyltransferase	172	153	154	163
KFAV_v1_2544	KFAV_v1_2544	conserved exported protein of unknown function	172	152	154	163
KFAV_v1_2287	KFAV_v1_2287	conserved protein of unknown function	172	152	154	163
hisC	KFAV_v1_1060	Histidinol-phosphate aminotransferase	171	152	154	163
KFAV_v1_0044	KFAV_v1_0044	Pro-sigmaK processing inhibitor BofA	171	152	154	162
KFAV_v1_2524	KFAV_v1_2524	rRNA methyltransferase	171	151	154	162
KFAV_v1_0110	KFAV_v1_0110	Dihydropteroate synthase	171	151	153	162
argC	KFAV_v1_0488	N-acetylglutamate gamma-semialdehyde dehydrogenase	171	151	153	161
nuoL_2	KFAV_v1_3494	NADH-quinone oxidoreductase subunit L	171	151	153	161
KFAV_v1_3025	KFAV_v1_3025	transposase	171	151	153	161
nadE	KFAV_v1_2420	NH(3)-dependent NAD(+) synthetase	171	151	152	161
KFAV_v1_0784	KFAV_v1_0784	transposase (fragment)	171	151	152	160
KFAV_v1_1427	KFAV_v1_1427	protein of unknown function	171	150	152	160
KFAV_v1_1529	KFAV_v1_1529	conserved protein of unknown function	171	150	152	160
KFAV_v1_3599	KFAV_v1_3599	conserved protein of unknown function	171	150	152	160
purF	KFAV_v1_0316	glutamine phosphoribosylpyrophosphate amidotransferase	170	150	152	160
ppk_1	KFAV_v1_2485	Polyphosphate kinase	170	150	152	160
KFAV_v1_2370	KFAV_v1_2370	Cysteine desulfurase	170	150	151	160
KFAV_v1_1938	KFAV_v1_1938	conserved protein of unknown function	170	150	151	159
KFAV_v1_2586	KFAV_v1_2586	Site-2 protease family protein	169	150	151	159
ysmB	KFAV_v1_1122	putative transcriptional regulator (mother cell's gene expression during sporulation)	169	149	151	159
ispA	KFAV_v1_1222	farnesyl diphosphate synthase	169	149	151	159
citZ	KFAV_v1_2570	citrate synthase II	169	149	151	158

apt	KFAV_v1_2395	adenine phosphoribosyltransferase	169	149	150	158
KFAV_v1_0626	KFAV_v1_0626	transposase (fragment)	169	149	150	158
rbgA	KFAV_v1_1961	ribosome biogenesis GTPase A	168	149	150	158
purL	KFAV_v1_0315	phosphoribosylformylglycinamidine synthetase subunit II	168	148	150	158
KFAV_v1_1658	KFAV_v1_1658	conserved protein of unknown function	168	148	150	158
thiG	KFAV_v1_1605	hydroxyethylthiazole phosphate synthetase (thiamine biosynthesis)	168	148	150	158
KFAV_v1_1145	KFAV_v1_1145	Bifunctional folylpolyglutamate synthase/dihydrofolate synthase	167	148	149	158
mqnE_1	KFAV_v1_0943	Aminodeoxyfutalosine synthase	167	148	149	157
tgt	KFAV_v1_2410	tRNA-guanine transglycosylase	167	148	149	157
KFAV_v1_3303	KFAV_v1_3303	conserved membrane protein of unknown function	166	148	149	157
gyrA	KFAV_v1_0008	DNA gyrase (subunit A)	166	148	149	157
KFAV_v1_0198	KFAV_v1_0198	conserved membrane protein of unknown function	166	147	149	157
KFAV_v1_2234	KFAV_v1_2234	2-keto-gluconate dehydrogenase (fragment)	166	147	149	157
KFAV_v1_2487	KFAV_v1_2487	Uncharacterized HIT-like protein aq_141	166	147	149	157
KFAV_v1_3353	KFAV_v1_3353	conserved protein of unknown function	166	147	148	157
KFAV_v1_0037	KFAV_v1_0037	PAS domain-containing sensor histidine kinase	166	146	148	157
nusA	KFAV_v1_1869	transcription translation coupling factor involved in Rho-dependent transcription termination	166	146	148	157
pyrAB_1	KFAV_v1_0541	pyrimidine-specific carbamoyl-phosphate synthetase (large subunit)	166	146	147	156
engB	KFAV_v1_1130	GTPase involved in ribosome 50S subunit assembly (maturation of the central 50S protuberance)	165	146	147	156
KFAV_v1_0567	KFAV_v1_0567	conserved protein of unknown function	165	146	147	156
KFAV_v1_2519	KFAV_v1_2519	conserved membrane protein of unknown function	165	145	147	156
KFAV_v1_0391	KFAV_v1_0391	UDP-galactopyranose mutase	164	145	147	156
ycsA_2	KFAV_v1_3342	putative tartrate dehydrogenase	164	145	147	155
KFAV_v1_1512	KFAV_v1_1512	conserved protein of unknown function	164	145	146	155
KFAV_v1_2093	KFAV_v1_2093	1-acyl-sn-glycerol-3-phosphate acyltransferase	164	145	146	155
KFAV_v1_3008	KFAV_v1_3008	conserved protein of unknown function	164	145	146	155
nadC	KFAV_v1_0107	nicotinate-nucleotide pyrophosphorylase (quinolinate phosphoribosyltransferase)	164	145	146	155
nadB	KFAV_v1_0106	L-aspartate oxidase	164	144	146	155
KFAV_v1_1091	KFAV_v1_1091	2-hydroxy-acid oxidase	164	144	146	155
KFAV_v1_1588	KFAV_v1_1588	Peptidase M50	164	144	146	155
asnB	KFAV_v1_1185	Asparagine synthetase [glutamine-hydrolyzing] 1	163	144	146	155
braG	KFAV_v1_3298	High-affinity branched-chain amino acid transport ATP-binding protein BraG	163	143	145	154
KFAV_v1_1415	KFAV_v1_1415	transposase (fragment)	163	143	145	154
yhfP	KFAV_v1_3409	putative oxidoreductase	163	143	145	154
KFAV_v1_3423	KFAV_v1_3423	Efflux transporter, RND family, MFP subunit	163	143	144	154
mqnA	KFAV_v1_1663	Chorismate dehydratase	162	143	144	153
KFAV_v1_1889	KFAV_v1_1889	CheY-P-specific phosphatase CheC	162	143	144	153
KFAV_v1_0342	KFAV_v1_0342	transposase	162	142	144	153
ylmE	KFAV_v1_1563	putative PLP-containing enzyme	162	142	144	153
KFAV_v1_2815	KFAV_v1_2815	conserved membrane protein of unknown function	161	142	144	153
pabA	KFAV_v1_1485	4-amino-4-deoxychorismate synthase; anthranilate synthase (subunit II)	161	141	144	153
plsY	KFAV_v1_1650	Glycerol-3-phosphate acyltransferase	161	140	144	153
KFAV_v1_1399	KFAV_v1_1399	conserved protein of unknown function	161	140	144	152
pucG	KFAV_v1_2330	(S)-ureidoglycine--glyoxylate transaminase	161	140	144	152
spoVG	KFAV_v1_0072	regulator required for spore cortex synthesis (stage V sporulation)	161	140	144	152
fliY	KFAV_v1_1902	flagellar motor switching and energizing phosphatase	161	140	143	152
sasP	KFAV_v1_2569	Small, acid-soluble spore protein 1	161	140	143	152
KFAV_v1_0752	KFAV_v1_0752	conserved exported protein of unknown function	160	139	143	152
sdhC	KFAV_v1_1118	succinate dehydrogenase (cytochrome b558 subunit)	160	139	143	151
KFAV_v1_1756	KFAV_v1_1756	conserved protein of unknown function	159	139	143	151
queD	KFAV_v1_0890	6-carboxytetrahydropterin synthase QueD	159	139	143	151
ribBA	KFAV_v1_1586	fused 3,4-dihydroxy-2-butanone 4-phosphate synthase and GTP cyclohydrolase II	159	138	143	151

KFAV_v1_0745	KFAV_v1_0745	conserved membrane protein of unknown function	159	138	143	151
minD	KFAV_v1_1157	ATPase activator of MinC	159	138	143	150
KFAV_v1_0659	KFAV_v1_0659	putative transcriptional regulator, XRE family	159	137	142	150
cbiJ	KFAV_v1_0827	Cobalt-precorrin-6A reductase	159	137	142	150
bkdR	KFAV_v1_2088	transcriptional regulator	159	137	142	150
trpB_2	KFAV_v1_1693	tryptophan synthase (beta subunit)	158	137	142	150
queE	KFAV_v1_0889	7-carboxy-7-deazaguanine synthase	158	137	141	149
fer	KFAV_v1_0893	Ferredoxin	158	137	141	149
sps	KFAV_v1_1993	putative serine/threonine-protein kinase Sps1	158	136	141	149
KFAV_v1_1872	KFAV_v1_1872	Glycosyl transferase family 2	158	136	141	149
KFAV_v1_0103	KFAV_v1_0103	protein of unknown function	158	136	141	149
gpmI	KFAV_v1_0609	phosphoglycerate mutase	158	136	141	149
KFAV_v1_1501	KFAV_v1_1501	Zinc finger protein 62	158	136	141	148
KFAV_v1_1481	KFAV_v1_1481	conserved protein of unknown function	158	135	141	148
greA_1	KFAV_v1_0116	transcription elongation factor resolving backtracking / stalling	157	135	141	148
rdgB	KFAV_v1_1124	deoxyinosine/deoxyxanthosine triphosphate pyrophosphatase, promiscuous (subunit A)	157	135	141	148
KFAV_v1_0099	KFAV_v1_0099	conserved membrane protein of unknown function	157	135	141	147
KFAV_v1_1052	KFAV_v1_1052	conserved membrane protein of unknown function	157	135	141	147
moaC	KFAV_v1_2091	molybdenum cofactor biosynthesis protein C	157	135	141	147
KFAV_v1_1426	KFAV_v1_1426	2-keto-4-pentenoate hydratase/2-oxohepta-3-ene-1,7-dioic acid hydratase (Catechol pathway)	157	135	141	147
KFAV_v1_1956	KFAV_v1_1956	conserved protein of unknown function	157	135	140	147
mnmA	KFAV_v1_2293	tRNA-specific 2-thiouridylase	156	134	140	147
pyk	KFAV_v1_2635	pyruvate kinase	156	134	140	147
hemX	KFAV_v1_1132	Protein HemX	156	134	140	146
KFAV_v1_1171	KFAV_v1_1171	Nucleotidyltransferase	155	134	140	146
yyaT	KFAV_v1_0875	putative acetyltransferase (polyamine degradation)	155	134	140	146
hisIE	KFAV_v1_0577	bifunctional phosphoribosyl-AMP cyclohydrolase; phosphoribosyl-ATP pyrophosphohydrolase	155	133	140	146
KFAV_v1_0495	KFAV_v1_0495	RluA family pseudouridine synthase	155	133	140	145
KFAV_v1_3339	KFAV_v1_3339	Acetylornithine deacetylase or succinyl-diaminopimelate desuccinylase	155	133	140	145
csx	KFAV_v1_3029	Type I-U CRISPR-associated protein Csx17	155	133	140	145
pdhA_1	KFAV_v1_0728	pyruvate dehydrogenase (E1 alpha subunit)	155	133	140	145
KFAV_v1_2289	KFAV_v1_2289	MFS transporter, OFA family, oxalate/formate antiporter	155	133	140	145
secDF_1	KFAV_v1_2402	fragment of protein-export membrane protein (part 2)	155	133	140	145
KFAV_v1_2964	KFAV_v1_2964	CRISPR-associated endoribonuclease Cas2 (modular protein)	154	133	139	145
fmt	KFAV_v1_1997	methionyl-tRNA formyltransferase	154	133	139	145
KFAV_v1_1237	KFAV_v1_1237	conserved protein of unknown function	153	133	139	145
pdhB_1	KFAV_v1_0729	pyruvate dehydrogenase (E1 beta subunit)	153	132	139	145
bkdB	KFAV_v1_2084	branched-chain alpha-keto acid dehydrogenase E2 subunit (lipoamide acyltransferase)	153	132	139	144
ispD	KFAV_v1_0139	2-C-methyl-D-erythritol 4-phosphate cytidylyltransferase, nonmevalonate isoprenoid pathway	153	132	138	144
KFAV_v1_2259	KFAV_v1_2259	GAF modulated sigma54 specific transcriptional regulator, Fis family	153	132	138	144
KFAV_v1_1243	KFAV_v1_1243	conserved membrane protein of unknown function	152	132	138	143
KFAV_v1_3383	KFAV_v1_3383	Type II secretion system F domain protein	152	132	138	143
KFAV_v1_3110	KFAV_v1_3110	conserved protein of unknown function	152	132	137	143
KFAV_v1_0018	KFAV_v1_0018	conserved protein of unknown function	152	132	137	143
KFAV_v1_0041	KFAV_v1_0041	conserved protein of unknown function	152	132	137	143
pdhB_3	KFAV_v1_1094	fragment of pyruvate dehydrogenase (E1 beta subunit) (part 2)	152	132	137	142
pncB	KFAV_v1_1098	Nicotinate phosphoribosyltransferase	152	131	137	142
KFAV_v1_1867	KFAV_v1_1867	Ribosomal protein L7Ae	152	131	137	142
KFAV_v1_3368	KFAV_v1_3368	putative enzyme	152	131	136	142
KFAV_v1_2276	KFAV_v1_2276	conserved protein of unknown function	151	131	136	142
KFAV_v1_0770	KFAV_v1_0770	conserved protein of unknown function	151	131	135	142
dapI	KFAV_v1_3544	N-acetyl-diaminopimelate deacetylase	151	131	135	142

livH_2	KFAV_v1_1701	leucine/isoleucine/valine transporter subunit ; membrane component of ABC superfamily	151	130	135	142
trxB	KFAV_v1_1236	thioredoxin reductase	151	130	135	142
KFAV_v1_3410	KFAV_v1_3410	conserved protein of unknown function	151	130	135	141
KFAV_v1_0260	KFAV_v1_0260	conserved protein of unknown function	151	130	135	141
thiE	KFAV_v1_1603	Thiamine-phosphate synthase	151	130	134	141
KFAV_v1_3605	KFAV_v1_3605	Cysteine desulfurase	150	130	134	141
KFAV_v1_0849	KFAV_v1_0849	conserved membrane protein of unknown function	150	130	134	141
KFAV_v1_1133	KFAV_v1_1133	conserved protein of unknown function	150	130	134	141
ypwA	KFAV_v1_1394	Carboxypeptidase 1	150	129	134	141
KFAV_v1_0429	KFAV_v1_0429	PilT protein domain protein	149	129	134	140
pdhC_1	KFAV_v1_0730	pyruvate dehydrogenase (dihydrolipoamide acetyltransferase E2 subunit)	149	129	134	140
KFAV_v1_0105	KFAV_v1_0105	conserved protein of unknown function	149	129	134	140
KFAV_v1_3112	KFAV_v1_3112	ATPase (AAA+ superfamily)-like protein	149	129	134	140
ppk_2	KFAV_v1_2545	Polyphosphate kinase 2	149	129	134	140
KFAV_v1_0534	KFAV_v1_0534	conserved protein of unknown function	148	129	133	140
KFAV_v1_1289	KFAV_v1_1289	AbrB family transcriptional regulator	148	129	133	140
KFAV_v1_1853	KFAV_v1_1853	Thioredoxin-disulfide reductase	148	128	133	140
defA	KFAV_v1_1998	peptide deformylase	148	128	132	140
KFAV_v1_2157	KFAV_v1_2157	conserved exported protein of unknown function	148	128	132	139
KFAV_v1_2523	KFAV_v1_2523	conserved membrane protein of unknown function	148	128	132	139
dapH	KFAV_v1_3545	tetrahydrodipicolinate N-acetyltransferase	148	128	132	139
fabG_7	KFAV_v1_2819	3-oxoacyl-[acyl-carrier-protein] reductase FabG	148	128	132	139
pepF	KFAV_v1_2667	oligoendopeptidase F	147	128	132	139
KFAV_v1_3280	KFAV_v1_3280	protein of unknown function	147	128	132	138
KFAV_v1_3420	KFAV_v1_3420	conserved protein of unknown function	147	128	132	138
KFAV_v1_2600	KFAV_v1_2600	conserved protein of unknown function	147	128	132	138
mccB	KFAV_v1_0681	cystathionine gamma-lyase and homocysteine gamma-lyase for reverse transsulfuration pathway	147	127	132	138
KFAV_v1_0620	KFAV_v1_0620	XRE family transcriptional regulator (fragment)	146	127	131	137
KFAV_v1_2684	KFAV_v1_2684	Sigma-54-dependent Fis family transcriptional regulator	146	127	131	137
yfnA	KFAV_v1_0345	Uncharacterized amino acid permease YfnA	146	127	131	137
KFAV_v1_1422	KFAV_v1_1422	ABC transporter permease	146	127	131	136
htrC	KFAV_v1_3578	sporulation membrane serine protease	146	127	130	136
KFAV_v1_3293	KFAV_v1_3293	Carnitine dehydratase	146	126	130	136
KFAV_v1_3336	KFAV_v1_3336	ECF transporter S component	146	126	130	136
hslO	KFAV_v1_0109	disulfide bond chaperone (heat shock protein HSP33)	146	126	130	136
mce	KFAV_v1_2080	Methylmalonyl-CoA epimerase	145	125	129	136
hisG	KFAV_v1_0571	ATP phosphoribosyltransferase	145	125	129	136
KFAV_v1_1050	KFAV_v1_1050	conserved protein of unknown function	145	125	129	136
KFAV_v1_1322	KFAV_v1_1322	NADPH:quinone oxidoreductase family protein	145	125	129	136
codV	KFAV_v1_1922	site-specific tyrosine recombinase for chromosome partitioning	145	125	129	135
KFAV_v1_1911	KFAV_v1_1911	conserved protein of unknown function	145	124	129	135
KFAV_v1_3393	KFAV_v1_3393	Diguanylate phosphodiesterase	145	124	129	135
KFAV_v1_3337	KFAV_v1_3337	Energy-coupling factor transporter transmembrane protein EcfT	145	124	129	135
argS	KFAV_v1_3560	arginyl-tRNA synthetase	145	124	128	135
dacF	KFAV_v1_2067	D-alanyl-D-alanine carboxypeptidase (penicilin binding protein)	145	124	128	135
KFAV_v1_1160	KFAV_v1_1160	conserved protein of unknown function	145	124	128	135
ALDH_1	KFAV_v1_0280	Aldehyde dehydrogenase	144	124	128	135
KFAV_v1_1416	KFAV_v1_1416	protein of unknown function	144	124	128	135
efpC	KFAV_v1_2305	first step modification enzyme of EF-P (addition of the amino- group of 5-aminopentanone)	144	123	127	134
KFAV_v1_2937	KFAV_v1_2937	protein of unknown function	144	123	127	134
KFAV_v1_3235	KFAV_v1_3235	Flagellar assembly factor FliW (fragment)	144	123	127	134
KFAV_v1_1423	KFAV_v1_1423	ABC transporter ATP-binding protein	144	123	127	134

glnQ_1	KFAV_v1_0129	glutamine ABC transporter (ATP-binding protein)	144	123	127	134
gmk	KFAV_v1_2002	guanylate kinase	144	123	127	134
htpX	KFAV_v1_2557	Protease HtpX homolog	143	122	127	134
KFAV_v1_2806	KFAV_v1_2806	Addiction module toxin, RelE/StbE family (fragment)	143	122	127	134
KFAV_v1_1682	KFAV_v1_1682	Long-chain fatty acid--CoA ligase	143	122	127	134
KFAV_v1_3399	KFAV_v1_3399	conserved membrane protein of unknown function	143	122	126	133
pdhB_2	KFAV_v1_1093	fragment of pyruvate dehydrogenase (E1 beta subunit) (part 1)	143	122	126	133
wecB	KFAV_v1_2302	UDP-N-acetylglucosamine 2-epimerase	143	122	126	133
KFAV_v1_1607	KFAV_v1_1607	protein of unknown function	143	122	126	133
KFAV_v1_2702	KFAV_v1_2702	conserved protein of unknown function	143	121	126	133
ktrB	KFAV_v1_2526	potassium transporter ATPase	143	121	126	133
KFAV_v1_0660	KFAV_v1_0660	conserved protein of unknown function	142	121	126	133
glmS	KFAV_v1_0209	L-glutamine-D-fructose-6-phosphate amidotransferase	142	121	126	133
KFAV_v1_1176	KFAV_v1_1176	conserved protein of unknown function	142	121	125	132
yqhS	KFAV_v1_1199	3-dehydroquinate dehydratase, type II	142	121	125	132
folB	KFAV_v1_0111	dihydroneopterin aldolase	142	121	125	132
KFAV_v1_3009	KFAV_v1_3009	conserved protein of unknown function	142	120	125	132
dayD	KFAV_v1_0056	D-amino acyl-tRNA deacylase	142	120	125	132
KFAV_v1_0612	KFAV_v1_0612	Carboxylesterase	142	120	124	132
KFAV_v1_2357	KFAV_v1_2357	Acyl-CoA thioesterase	142	120	124	132
cspC_2	KFAV_v1_3080	cold-shock protein	142	119	124	131
leuD	KFAV_v1_2582	3-isopropylmalate dehydratase (small subunit)	141	119	124	131
KFAV_v1_1239	KFAV_v1_1239	3-oxoacyl-ACP reductase	141	119	123	131
KFAV_v1_0700	KFAV_v1_0700	conserved protein of unknown function	141	119	123	131
KFAV_v1_1764	KFAV_v1_1764	conserved protein of unknown function	141	119	123	131
KFAV_v1_0240	KFAV_v1_0240	5-formyltetrahydrofolate cyclo-ligase	140	119	123	131
thiD	KFAV_v1_2861	phosphomethylpyrimidine kinase, 4-amino-5-hydroxymethyl-2-methylpyrimidine and 4-amino-5-hydroxymethyl-2-methylpyrimidine pyrophosphate kinase	140	118	123	131
KFAV_v1_0665	KFAV_v1_0665	PIN domain nuclease	140	118	123	131
aceA	KFAV_v1_2386	isocitrate lyase	140	118	122	130
ypjQ	KFAV_v1_2641	putative phosphatidylglycerophosphatase	140	118	122	130
KFAV_v1_2936	KFAV_v1_2936	conserved protein of unknown function	140	118	122	130
ecfA	KFAV_v1_3338	Energy-coupling factor transporter ATP-binding protein EcfA	140	117	122	130
KFAV_v1_1935	KFAV_v1_1935	conserved protein of unknown function	140	117	122	130
nuoN_2	KFAV_v1_3492	NADH-quinone oxidoreductase subunit N	140	117	121	129
KFAV_v1_2314	KFAV_v1_2314	conserved membrane protein of unknown function	139	117	121	129
KFAV_v1_1826	KFAV_v1_1826	Class III poly(R)-hydroxyalkanoic acid synthase subunit PhaC	139	117	121	129
KFAV_v1_1520	KFAV_v1_1520	conserved protein of unknown function	139	117	121	129
pdtaR	KFAV_v1_2845	putative transcriptional regulatory protein pdtaR	139	117	121	128
KFAV_v1_1987	KFAV_v1_1987	DNA helicase RecG	139	116	121	128
folEA	KFAV_v1_1623	GTP cyclohydrolase I	139	116	120	128
iadA	KFAV_v1_2888	Isoaspartyl dipeptidase	139	116	120	128
KFAV_v1_0708	KFAV_v1_0708	Iron transporter	139	116	120	128
polC	KFAV_v1_1871	DNA polymerase III (alpha subunit)	139	116	120	128
KFAV_v1_1089	KFAV_v1_1089	transposase	139	116	120	128
KFAV_v1_2823	KFAV_v1_2823	conserved protein of unknown function	138	115	119	128
accB	KFAV_v1_1211	acetyl-CoA carboxylase subunit (biotin carboxyl carrier subunit)	138	115	119	127
KFAV_v1_2153	KFAV_v1_2153	transposase (fragment)	138	115	118	127
KFAV_v1_1913	KFAV_v1_1913	Flagellar assembly protein FliH/Type III secretion system HrpE	138	115	118	127
KFAV_v1_1018	KFAV_v1_1018	RNA polymerase subunit sigma-24	138	115	118	127
KFAV_v1_1059	KFAV_v1_1059	protein of unknown function	138	115	118	127
KFAV_v1_1552	KFAV_v1_1552	Cell division protein FtsQ	138	115	118	127
KFAV_v1_2422	KFAV_v1_2422	conserved protein of unknown function	138	114	118	127

nfrA	KFAV_v1_3000	FMN reductase (NADPH)	138	114	118	126
speB_2	KFAV_v1_3561	agmatinase	138	114	118	126
spoVR_1	KFAV_v1_0870	involved in spore cortex synthesis (stage V sporulation, conserved in non sporulating bacteria	137	114	118	126
KFAV_v1_2155	KFAV_v1_2155	DNA polymerase III subunit delta	137	114	117	126
speE_1	KFAV_v1_0859	Polyamine aminopropyltransferase	137	114	117	126
xylE_3	KFAV_v1_2468	Metapyrocatechase	137	114	117	126
KFAV_v1_3341	KFAV_v1_3341	putative aldehyde-dehydrogenase-like protein y4uC	137	113	117	126
KFAV_v1_2418	KFAV_v1_2418	conserved exported protein of unknown function	137	113	117	126
KFAV_v1_3209	KFAV_v1_3209	conserved protein of unknown function	137	113	117	125
fadA_1	KFAV_v1_0970	3-ketoacyl-CoA thiolase	136	113	117	125
KFAV_v1_0971	KFAV_v1_0971	2,3-dihydroxybenzoate-AMP ligase	136	113	117	125
KFAV_v1_3185	KFAV_v1_3185	conserved protein of unknown function	135	113	117	125
KFAV_v1_3240	KFAV_v1_3240	conserved protein of unknown function	135	113	116	125
KFAV_v1_0546	KFAV_v1_0546	conserved exported protein of unknown function	135	113	116	125
KFAV_v1_0208	KFAV_v1_0208	conserved protein of unknown function	134	113	116	125
KFAV_v1_3419	KFAV_v1_3419	conserved membrane protein of unknown function	134	113	116	125
cysE	KFAV_v1_0142	serine O-acetyltransferase	134	112	115	124
nifW	KFAV_v1_1759	Putative nitrogenase stabilizing/protective protein	134	112	115	124
KFAV_v1_3272	KFAV_v1_3272	2-nitropropane dioxygenase	134	112	115	124
tsaD	KFAV_v1_0239	tRNA(NNU) t(6)A37 threonylcarbamoyladenosine modification; glycation binding protein	134	112	115	124
mutL	KFAV_v1_1788	DNA mismatch repair protein MutL	134	111	115	124
resE	KFAV_v1_1598	two-component sensor histidine kinase (ResD / ResE)	134	111	115	124
spcP	KFAV_v1_0088	spore protein involved in the shaping of the spore coat	133	111	115	124
fucA	KFAV_v1_3291	L-fuculose phosphate aldolase	133	111	115	124
KFAV_v1_0739	KFAV_v1_0739	conserved protein of unknown function	133	111	115	124
coaE	KFAV_v1_2628	dephosphocoenzyme A kinase	133	111	114	124
ypjC	KFAV_v1_1711	putative integral inner membrane protein	133	111	114	123
KFAV_v1_2486	KFAV_v1_2486	CoA transferase	132	111	114	123
nadD	KFAV_v1_2165	putative nicotinate-nucleotide adenylyltransferase	132	111	114	123
KFAV_v1_1424	KFAV_v1_1424	conserved protein of unknown function	132	111	114	123
KFAV_v1_3401	KFAV_v1_3401	conserved membrane protein of unknown function	132	110	114	123
dapF	KFAV_v1_2005	Diaminopimelate epimerase	132	110	114	123
KFAV_v1_3227	KFAV_v1_3227	protein of unknown function	132	110	114	123
KFAV_v1_1625	KFAV_v1_1625	conserved protein of unknown function	131	110	113	123
acdA_2	KFAV_v1_3554	acyl-CoA dehydrogenase	131	110	113	123
hisS	KFAV_v1_2300	histidyl-tRNA synthetase	131	110	113	122
efpB	KFAV_v1_1770	putative C-S lyase involved in a first step of EF-P modification	131	110	113	122
KFAV_v1_1799	KFAV_v1_1799	transposase	131	110	113	122
KFAV_v1_2337	KFAV_v1_2337	protein of unknown function	131	110	113	122
KFAV_v1_2588	KFAV_v1_2588	Dihydrolipoamide acetyltransferase component of pyruvate dehydrogenase complex	131	110	112	122
KFAV_v1_0115	KFAV_v1_0115	Quinate 5-dehydrogenase	130	110	112	122
KFAV_v1_2660	KFAV_v1_2660	conserved protein of unknown function	130	110	112	122
KFAV_v1_3486	KFAV_v1_3486	conserved protein of unknown function	130	110	112	122
KFAV_v1_3392	KFAV_v1_3392	conserved protein of unknown function	130	110	112	122
KFAV_v1_0850	KFAV_v1_0850	conserved protein of unknown function	130	110	112	121
mlpA	KFAV_v1_1856	specific processing protease	130	110	111	121
yqxK	KFAV_v1_2076	putative nucleotide binding protein	129	109	111	121
metS	KFAV_v1_0055	methionyl-tRNA synthetase	129	109	111	121
KFAV_v1_0254	KFAV_v1_0254	Polysaccharide deacetylase	129	109	111	121
cysS	KFAV_v1_0143	dual cysteinyl-tRNA synthetase; cysteine persulfide synthase	129	109	111	121
frvX	KFAV_v1_0911	putative fructose-lysine aminopeptidase	129	109	111	121
KFAV_v1_2967	KFAV_v1_2967	conserved protein of unknown function	129	109	111	121

KFAV_v1_2970	KFAV_v1_2970	conserved protein of unknown function	129	109	111	121
copA_2	KFAV_v1_2918	copper [Cu(I)] transporter ATPase	129	109	111	121
argR	KFAV_v1_1227	transcriptional regulator (AhrC(ArgR)-arginine)	128	109	110	121
ispG	KFAV_v1_1874	4-hydroxy-3-methylbut-2-en-1-yl diphosphate synthase (1-hydroxy-2-methyl-2-(E)-butenyl 4-diphosphate synthase)	128	109	110	121
yrbF	KFAV_v1_2409	component of the preprotein translocase	128	109	110	121
KFAV_v1_2807	KFAV_v1_2807	conserved protein of unknown function	128	109	110	120
ttuB	KFAV_v1_2868	putative tartrate transporter	128	108	110	120
KFAV_v1_3104	KFAV_v1_3104	conserved protein of unknown function	128	108	110	120
rlmN	KFAV_v1_1995	23S rRNA m2A2503 methyltransferase and tRNA A37 C2 methyltransferase	128	108	110	120
recN	KFAV_v1_1228	DNA repair protein RecN	128	108	110	120
KFAV_v1_1562	KFAV_v1_1562	Polyphenol oxidase	128	108	110	120
cas5u6u	KFAV_v1_3027	Type I-U CRISPR-associated protein Cas5/Cas6	128	108	109	120
ltrA_1	KFAV_v1_0517	Group II intron reverse transcriptase/maturase	127	108	109	120
KFAV_v1_0664	KFAV_v1_0664	AbrB family transcriptional regulator	127	108	109	120
fabG_2	KFAV_v1_1827	beta-ketoacyl-acyl carrier protein reductase	127	108	108	119
mmgA_1	KFAV_v1_3514	degradative acetoacetyl-CoA thiolase	127	108	108	119
KFAV_v1_3526	KFAV_v1_3526	protein of unknown function	127	107	108	119
KFAV_v1_2971	KFAV_v1_2971	CRISPR-associated protein Cas6	127	107	108	119
KFAV_v1_0531	KFAV_v1_0531	protein of unknown function	126	107	108	118
thiC	KFAV_v1_0771	phosphomethylpyrimidine synthase	126	107	108	118
cas_1	KFAV_v1_2966	CRISPR-associated protein Cas4	126	107	108	118
bipA	KFAV_v1_1531	ribosome-associated GTPase	126	107	108	118
fadF	KFAV_v1_3558	putative iron-sulphur-binding reductase	126	107	108	118
KFAV_v1_2919	KFAV_v1_2919	Radical SAM protein	126	107	108	118
yrkJ	KFAV_v1_2690	putative membrane transporter protein YrkJ	125	106	108	118
ribH	KFAV_v1_1587	6,7-dimethyl-8-ribityllumazine synthase, beta subunit	125	106	108	117
KFAV_v1_2009	KFAV_v1_2009	conserved membrane protein of unknown function	125	106	108	117
feoB	KFAV_v1_2796	Fe(2+) transporter FeoB	125	106	107	117
recD_1	KFAV_v1_2665	ATP-dependent RecD-like DNA helicase	125	106	107	117
aroF	KFAV_v1_1688	chorismate synthase	124	106	107	117
KFAV_v1_1554	KFAV_v1_1554	conserved protein of unknown function	124	105	107	117
aceB_2	KFAV_v1_2385	malate synthase A	124	105	107	117
hupN_2	KFAV_v1_2598	HU-related DNA-binding protein; phage SPbeta	124	105	106	117
KFAV_v1_1179	KFAV_v1_1179	conserved protein of unknown function	124	105	106	117
KFAV_v1_2313	KFAV_v1_2313	conserved protein of unknown function	123	105	106	117
KFAV_v1_1530	KFAV_v1_1530	HAD family hydrolase	123	105	106	116
miaB	KFAV_v1_1791	enzyme for ms(2)i(6)A formation for tRNA modification	123	105	106	116
KFAV_v1_2118	KFAV_v1_2118	Na/Pi-cotransporter II-related protein	123	104	106	116
alaS	KFAV_v1_2277	alanyl-tRNA synthetase	123	104	106	116
priA	KFAV_v1_1999	primosomal replication factor Y (primosomal protein N')	123	104	106	116
KFAV_v1_0560	KFAV_v1_0560	OmpA/MotB domain protein	123	104	105	116
KFAV_v1_0341	KFAV_v1_0341	conserved protein of unknown function	123	104	105	116
rsgA	KFAV_v1_1992	GTPase involved in ribosome biogenesis	122	104	105	115
KFAV_v1_3046	KFAV_v1_3046	protein of unknown function	122	104	105	115
pgi	KFAV_v1_1500	glucose-6-phosphate isomerase	122	103	105	115
KFAV_v1_0702	KFAV_v1_0702	Cation transporter	122	103	105	115
dut	KFAV_v1_1855	Deoxyuridine 5'-triphosphate nucleotidohydrolase	122	103	105	115
scpA	KFAV_v1_1589	chromosome condensation and partitioning factor	122	103	104	115
KFAV_v1_1257	KFAV_v1_1257	Metal-dependent phosphohydrolase	122	103	104	114
dnaX	KFAV_v1_0038	DNA polymerase III subunit gamma/tau	122	103	104	114
hfq	KFAV_v1_1785	RNA-binding protein Hfq	122	103	104	114
paaA	KFAV_v1_2137	putative multicomponent oxygenase/reductase subunit for phenylacetic acid degradation	122	103	104	114

cas7u	KFAV_v1_3028	Type I-U CRISPR-associated protein Cas7	122	103	104	114
gpsA	KFAV_v1_1651	NADPH-dependent glycerol-3-phosphate dehydrogenase	121	103	104	114
acdA_1	KFAV_v1_2664	Acyl-CoA dehydrogenase	121	103	103	114
argF	KFAV_v1_0542	ornithine carbamoyltransferase	121	103	103	114
ytnA	KFAV_v1_3283	putative amino acid permease	121	102	103	114
KFAV_v1_2657	KFAV_v1_2657	conserved protein of unknown function	121	102	103	113
hisD	KFAV_v1_0572	histidinol dehydrogenase	120	102	103	113
metQ	KFAV_v1_1408	methionine ABC transporter, substrate binding lipoprotein	120	102	103	113
KFAV_v1_2123	KFAV_v1_2123	IclR family transcriptional regulator	120	102	102	113
glnQ_2	KFAV_v1_3081	glutamine ABC transporter (ATP-binding protein)	120	102	102	113
hisH	KFAV_v1_0574	imidazole glycerol phosphate synthase, glutamine amidotransferase subunit	120	102	102	113
KFAV_v1_1781	KFAV_v1_1781	Branched-chain amino acid ABC transporter permease	120	102	102	113
yerC	KFAV_v1_3070	transcriptional repressor - histidine operons	119	102	102	112
KFAV_v1_0357	KFAV_v1_0357	Copper oxidase	119	102	102	112
fabZ	KFAV_v1_3476	beta-hydroxyacyl-[acyl carrier protein] dehydratase	119	101	102	112
bkdAB_2	KFAV_v1_2589	branched-chain alpha-keto acid dehydrogenase E1 subunit	119	101	102	112
KFAV_v1_0089	KFAV_v1_0089	conserved membrane protein of unknown function	119	101	102	112
rpoZ	KFAV_v1_2001	omega subunit of RNA polymerase	119	101	102	112
KFAV_v1_2254	KFAV_v1_2254	GAF modulated sigma54 specific transcriptional regulator, Fis family	119	101	102	112
KFAV_v1_3253	KFAV_v1_3253	Resolvase domain protein	118	101	102	112
rpiB_2	KFAV_v1_3518	D-ribose 5-phosphate isomerase	118	101	102	112
nuoM_2	KFAV_v1_3493	Proton-translocating NADH-quinone oxidoreductase subunit M	118	101	102	112
KFAV_v1_3076	KFAV_v1_3076	protein of unknown function	118	100	102	111
KFAV_v1_3254	KFAV_v1_3254	conserved protein of unknown function	118	100	101	111
KFAV_v1_2592	KFAV_v1_2592	Sigma-54-dependent Fis family transcriptional regulator	118	100	101	111
KFAV_v1_1700	KFAV_v1_1700	Inner-membrane translocator	118	100	101	111
KFAV_v1_3269	KFAV_v1_3269	Phosphatidylglycerol lysyltransferase (fragment)	118	100	101	111
KFAV_v1_1733	KFAV_v1_1733	conserved protein of unknown function	118	100	101	111
KFAV_v1_3089	KFAV_v1_3089	conserved protein of unknown function	118	100	101	111
KFAV_v1_2373	KFAV_v1_2373	conserved protein of unknown function	117	100	101	111
coxS_1	KFAV_v1_2539	Carbon monoxide dehydrogenase small chain	117	100	101	111
KFAV_v1_0500	KFAV_v1_0500	protein of unknown function	117	100	101	110
cynS	KFAV_v1_1013	Cyanate hydratase	117	100	101	110
KFAV_v1_1197	KFAV_v1_1197	Histidine phosphatase family protein	117	99	100	110
KFAV_v1_0680	KFAV_v1_0680	conserved membrane protein of unknown function	117	99	100	110
KFAV_v1_3144	KFAV_v1_3144	protein of unknown function	117	99	100	110
KFAV_v1_3512	KFAV_v1_3512	conserved membrane protein of unknown function	117	99	100	110
lysA	KFAV_v1_1581	meso-2,6-diaminopimelate decarboxylase	117	99	100	110
KFAV_v1_0049	KFAV_v1_0049	conserved protein of unknown function	116	99	100	110
moaE	KFAV_v1_2571	molybdopterin synthase (large subunit)	116	99	100	109
KFAV_v1_2563	KFAV_v1_2563	conserved protein of unknown function	116	99	100	109
KFAV_v1_3312	KFAV_v1_3312	conserved membrane protein of unknown function	116	99	100	109
KFAV_v1_0624	KFAV_v1_0624	Integral membrane protein TerC	116	99	100	109
KFAV_v1_1487	KFAV_v1_1487	conserved protein of unknown function	116	99	100	109
gatC	KFAV_v1_3065	glutamyl-tRNA(Gln) amidotransferase (subunit C)	116	98	100	109
iscSA	KFAV_v1_2294	cysteine desulfurase involved in U34 tRNA thiolation	115	98	99	109
KFAV_v1_2284	KFAV_v1_2284	conserved protein of unknown function	115	98	99	109
KFAV_v1_1778	KFAV_v1_1778	protein of unknown function	115	98	99	109
rpoN	KFAV_v1_0604	RNA polymerase sigma-54 factor	115	98	99	108
ylbK	KFAV_v1_1983	putative hydrolase	115	98	99	108
KFAV_v1_2580	KFAV_v1_2580	conserved protein of unknown function	115	98	99	108
KFAV_v1_2374	KFAV_v1_2374	Serine hydrolase	115	98	99	108

KFAV_v1_2387	KFAV_v1_2387	LysR family transcriptional regulator	115	98	99	108
pcrA	KFAV_v1_3068	ATP-dependent DNA helicase	115	98	99	108
yitW_2	KFAV_v1_1037	Fe-S protein maturation auxiliary factor YitW	114	98	99	108
KFAV_v1_0760	KFAV_v1_0760	conserved protein of unknown function	114	97	98	107
KFAV_v1_2917	KFAV_v1_2917	conserved protein of unknown function	114	97	98	107
argH	KFAV_v1_0544	argininosuccinate lyase	114	97	98	107
trmFO	KFAV_v1_1923	tRNA:m(5)U-54 methyltransferase	114	97	98	107
mtaB	KFAV_v1_2120	tRNA N(6)-threonylcarbamoyladenosine (t(6)A) methylthiotransferase	114	97	98	107
gmuF	KFAV_v1_1169	putative mannose-6-phosphate isomerase GmuF	114	97	98	107
coaX	KFAV_v1_0108	pantothenate kinase type III	113	97	98	106
pxpA_2	KFAV_v1_0787	oxoprolinase subunit A	113	97	97	106
uvrC	KFAV_v1_1108	excinuclease ABC (subunit C)	113	97	97	106
KFAV_v1_2517	KFAV_v1_2517	Glycosyl transferase	113	96	97	106
KFAV_v1_2412	KFAV_v1_2412	conserved protein of unknown function	113	96	97	106
KFAV_v1_1841	KFAV_v1_1841	2-hydroxyhepta-2,4-diene-1,7-dioate isomerase	113	96	97	106
KFAV_v1_0394	KFAV_v1_0394	Glycosyltransferase involved in cell wall bisynthesis	113	96	97	106
KFAV_v1_0024	KFAV_v1_0024	Cupin domain-containing protein	113	96	97	106
KFAV_v1_0783	KFAV_v1_0783	protein of unknown function	113	96	97	105
cdaA	KFAV_v1_0205	diadenylate cyclase	112	96	96	105
KFAV_v1_1180	KFAV_v1_1180	conserved protein of unknown function	112	96	96	105
acoA_2	KFAV_v1_2590	fragment of acetoin dehydrogenase E1 component (TPP-dependent alpha subunit) (part 2)	112	96	96	105
KFAV_v1_2699	KFAV_v1_2699	transposase	112	96	96	105
KFAV_v1_1952	KFAV_v1_1952	conserved membrane protein of unknown function	112	95	96	105
KFAV_v1_0912	KFAV_v1_0912	conserved protein of unknown function	112	95	96	105
KFAV_v1_3208	KFAV_v1_3208	protein of unknown function	112	95	96	105
rnhB	KFAV_v1_1958	Ribonuclease HII	112	95	96	105
yfdE	KFAV_v1_3309	Acetyl-CoA:oxalate CoA-transferase	112	95	95	105
KFAV_v1_1319	KFAV_v1_1319	NADPH:quinone reductase	112	95	95	105
KFAV_v1_2963	KFAV_v1_2963	transposase	112	95	95	105
KFAV_v1_3031	KFAV_v1_3031	CRISPR-associated endonuclease Cas3	112	95	95	105
KFAV_v1_0896	KFAV_v1_0896	MBL fold metallo-hydrolase	111	94	95	105
cheY	KFAV_v1_1901	regulator of chemotaxis and motility	111	94	95	105
KFAV_v1_2825	KFAV_v1_2825	transposase (fragment)	111	94	95	105
nagA	KFAV_v1_1034	N-acetylglucosamine-6-phosphate deacetylase	111	94	95	105
udk	KFAV_v1_2229	uridine kinase	111	94	95	104
coxL_1	KFAV_v1_2538	Carbon monoxide dehydrogenase large chain	111	94	95	104
KFAV_v1_2081	KFAV_v1_2081	Methylmalonyl-CoA mutase	111	94	95	104
yqhH	KFAV_v1_1192	putative RNA polymerase-associated helicase protein	111	94	95	104
gabT	KFAV_v1_0791	4-aminobutyrate aminotransferase	111	94	94	104
yeaC	KFAV_v1_0289	putative MoxR-like ATPase	111	94	94	104
KFAV_v1_1323	KFAV_v1_1323	quinone oxidoreductase, NADPH-dependent (fragment)	111	94	94	104
KFAV_v1_3400	KFAV_v1_3400	Glycosyl transferase family 2	111	94	94	103
KFAV_v1_0063	KFAV_v1_0063	5-carboxymethyl-2-hydroxymuconate isomerase	110	94	94	103
KFAV_v1_3214	KFAV_v1_3214	transposase	110	94	94	103
KFAV_v1_2164	KFAV_v1_2164	Metal-dependent phosphohydrolase	110	94	94	103
KFAV_v1_0415	KFAV_v1_0415	Sugar transferase	110	94	94	103
KFAV_v1_1242	KFAV_v1_1242	conserved protein of unknown function	110	94	93	103
KFAV_v1_1897	KFAV_v1_1897	conserved membrane protein of unknown function	109	94	93	103
KFAV_v1_0652	KFAV_v1_0652	Copper amine oxidase domain protein	109	94	93	103
KFAV_v1_0455	KFAV_v1_0455	conserved exported protein of unknown function	109	94	93	103
csaB	KFAV_v1_3380	Polysaccharide pyruvyl transferase CsaB	109	93	93	103
KFAV_v1_2801	KFAV_v1_2801	HAD family hydrolase	109	93	93	103

tsaC_1	KFAV_v1_3340	4-formylbenzenesulfonate dehydrogenase TsaC1/TsaC2	109	93	93	102
kapD	KFAV_v1_1353	putative 3'-5' exonuclease KapD	109	93	93	102
KFAV_v1_3109	KFAV_v1_3109	Helicase domain protein	108	93	93	102
recJ	KFAV_v1_2887	Single-stranded-DNA-specific exonuclease RecJ	108	93	93	102
dnaEC	KFAV_v1_2642	DNA polymerase III (alpha subunit), DnaE3	108	93	92	102
KFAV_v1_3352	KFAV_v1_3352	conserved protein of unknown function	108	93	92	102
serS	KFAV_v1_0923	Serine--tRNA ligase	108	93	92	102
KFAV_v1_2846	KFAV_v1_2846	Histidine kinase	107	93	92	102
KFAV_v1_0079	KFAV_v1_0079	Peptidylprolyl isomerase	107	93	92	102
KFAV_v1_1662	KFAV_v1_1662	Thiamine biosynthesis protein ThiH-like protein	107	93	92	101
rlmI	KFAV_v1_2117	Ribosomal RNA large subunit methyltransferase I	107	93	92	101
gltX_2	KFAV_v1_1085	Glutamate--tRNA ligase	107	92	92	101
KFAV_v1_0257	KFAV_v1_0257	Addiction module toxin, RelE/StbE family	107	92	92	101
aroK	KFAV_v1_1184	Shikimate kinase	107	92	91	101
dapA_1	KFAV_v1_1842	4-hydroxy-tetrahydrodipicolinate synthase	107	92	91	101
KFAV_v1_1930	KFAV_v1_1930	L-threonine dehydratase catabolic TdcB	107	92	91	101
KFAV_v1_2723	KFAV_v1_2723	conserved protein of unknown function	107	92	91	100
nth	KFAV_v1_2775	endonuclease III, apurinic apyrimidic DNA lyase	107	92	91	100
KFAV_v1_2227	KFAV_v1_2227	conserved protein of unknown function	107	92	91	100
KFAV_v1_2968	KFAV_v1_2968	CRISPR-associated protein Cas5	107	91	91	100
ktrC	KFAV_v1_2525	potassium uptake protein	107	91	91	100
KFAV_v1_3403	KFAV_v1_3403	conserved protein of unknown function	106	91	91	100
KFAV_v1_0282	KFAV_v1_0282	conserved protein of unknown function	106	91	91	100
ldeE_1	KFAV_v1_1537	methylcrotonoyl-CoA carboxylase subunit (leucine degradation)	106	91	91	100
hmuV	KFAV_v1_2661	Hemin import ATP-binding protein HmuV	106	91	91	100
KFAV_v1_2018	KFAV_v1_2018	Peroxiredoxin	106	91	91	100
KFAV_v1_3396	KFAV_v1_3396	putative enzyme	106	91	91	100
ytkK	KFAV_v1_1396	putative oxidoreductase YtkK	106	91	90	100
murD	KFAV_v1_1547	UDP-N-acetylmuramoylalanyl-D-glutamate ligase	106	90	90	100
KFAV_v1_3603	KFAV_v1_3603	conserved protein of unknown function	106	90	90	100
KFAV_v1_1782	KFAV_v1_1782	ABC transporter substrate-binding protein	106	90	90	100
KFAV_v1_2233	KFAV_v1_2233	conserved protein of unknown function	106	90	90	100
yjbI	KFAV_v1_2890	putative thiol management oxidoreductase component	106	90	90	100
queA	KFAV_v1_2411	S-adenosylmethionine tRNA ribosyltransferase-isomerase	105	90	90	100
sigF	KFAV_v1_1574	RNA polymerase sporulation-specific sigma factor (sigma-F)	105	90	90	100
coaBC	KFAV_v1_2000	coenzyme A biosynthesis bifunctional protein CoaBC; phosphopantothenoylcysteine synthetase/decarboxylase	105	90	90	100
rsmA	KFAV_v1_0058	dimethyladenosine 16S ribosomal RNA transferase	105	90	90	99
KFAV_v1_3260	KFAV_v1_3260	protein of unknown function	105	90	90	99
rulR	KFAV_v1_1868	molecular ruler co-factor for RNA; new fold	105	90	90	99
KFAV_v1_2849	KFAV_v1_2849	Pirin family protein	105	90	89	99
KFAV_v1_1622	KFAV_v1_1622	conserved exported protein of unknown function	105	90	89	99
KFAV_v1_0356	KFAV_v1_0356	Copper oxidase	105	89	89	99
KFAV_v1_0548	KFAV_v1_0548	conserved membrane protein of unknown function	104	89	89	99
KFAV_v1_1667	KFAV_v1_1667	Patatin	104	89	89	99
KFAV_v1_3287	KFAV_v1_3287	conserved protein of unknown function	104	89	89	99
spoVD	KFAV_v1_1544	transpeptidase penicillin-binding protein (forespore targeted)	104	89	89	99
KFAV_v1_1071	KFAV_v1_1071	conserved protein of unknown function	104	89	89	99
rnr	KFAV_v1_0613	ribonuclease R	104	89	89	99
KFAV_v1_1302	KFAV_v1_1302	conserved protein of unknown function	104	89	89	99
KFAV_v1_2500	KFAV_v1_2500	conserved protein of unknown function	104	89	89	99
KFAV_v1_1843	KFAV_v1_1843	GntR family transcriptional regulator	104	88	88	98
KFAV_v1_2572	KFAV_v1_2572	Molybdopterin synthase sulfur carrier subunit (fragment)	104	88	88	98

KFAV_v1_3259	KFAV_v1_3259	conserved protein of unknown function	104	88	88	98
lonA	KFAV_v1_1129	class III heat-shock ATP-dependent LonA protease	103	88	88	98
KFAV_v1_0233	KFAV_v1_0233	conserved membrane protein of unknown function	103	88	88	98
lcfA	KFAV_v1_2599	Long-chain-fatty-acid--CoA ligase	103	88	88	98
yjlD	KFAV_v1_2156	NADH dehydrogenase-like protein YjlD	103	88	88	98
KFAV_v1_0662	KFAV_v1_0662	conserved protein of unknown function	103	88	88	98
trpE	KFAV_v1_1486	Anthranilate synthase component 1	103	88	88	98
KFAV_v1_1321	KFAV_v1_1321	Putative alcohol dehydrogenase	103	87	88	98
KFAV_v1_0113	KFAV_v1_0113	Transcriptional regulator	103	87	88	97
KFAV_v1_3236	KFAV_v1_3236	Flagellar assembly factor FliW (fragment)	103	87	88	97
truB	KFAV_v1_1863	tRNA pseudouridine synthase B	102	87	88	97
KFAV_v1_2938	KFAV_v1_2938	conserved protein of unknown function	102	87	88	97
panD	KFAV_v1_1721	aspartate 1-decarboxylase	102	87	87	97
flhA	KFAV_v1_1895	component of the flagellar export machinery	102	87	87	97
KFAV_v1_3252	KFAV_v1_3252	transposase	102	87	87	96
mreD	KFAV_v1_1155	Rod shape-determining protein MreD	102	87	87	96
KFAV_v1_0650	KFAV_v1_0650	transposase (fragment)	102	87	87	96
KFAV_v1_0701	KFAV_v1_0701	2-hydroxy-acid oxidase	102	87	87	96
KFAV_v1_3543	KFAV_v1_3543	conserved exported protein of unknown function	102	87	87	96
KFAV_v1_2014	KFAV_v1_2014	EamA family transporter	102	86	87	96
ctpA	KFAV_v1_0557	Carboxy-terminal processing protease CtpA	102	86	87	96
KFAV_v1_0627	KFAV_v1_0627	transposase (fragment)	101	86	86	96
KFAV_v1_1382	KFAV_v1_1382	Pyridine nucleotide-disulfide oxidoreductase	101	86	86	95
KFAV_v1_1492	KFAV_v1_1492	protein of unknown function	101	86	86	95
KFAV_v1_2228	KFAV_v1_2228	protein of unknown function	101	86	86	95
KFAV_v1_2369	KFAV_v1_2369	Ribulose phosphate epimerase	101	86	86	95
KFAV_v1_2822	KFAV_v1_2822	protein of unknown function	101	86	86	95
flgG	KFAV_v1_3478	Flagellar basal body protein	101	86	86	95
yqjD	KFAV_v1_2079	putative propionyl-CoA carboxylase beta chain	101	86	86	95
mmgB_2	KFAV_v1_3556	3-hydroxybutyryl-CoA dehydrogenase	101	86	86	95
tmk	KFAV_v1_0047	Thymidylate kinase	101	85	85	95
KFAV_v1_2358	KFAV_v1_2358	ATPase	101	85	85	95
KFAV_v1_1006	KFAV_v1_1006	putative spermidine/putrescine transport system substrate-binding protein	101	85	85	95
KFAV_v1_2583	KFAV_v1_2583	Spore coat protein	100	85	85	95
fliO	KFAV_v1_1900	Flagellar protein	100	85	85	95
gyrB	KFAV_v1_0007	DNA gyrase (subunit B)	100	85	85	94
KFAV_v1_0291	KFAV_v1_0291	Transglutaminase domain protein	100	85	85	94
KFAV_v1_1996	KFAV_v1_1996	16S rRNA (Cytosine(967)-C(5))-methyltransferase RsmB	100	85	85	94
KFAV_v1_0998	KFAV_v1_0998	protein of unknown function	99	85	85	94
KFAV_v1_3045	KFAV_v1_3045	transposase (fragment)	99	85	85	94
yuiH	KFAV_v1_0945	Uncharacterized oxidoreductase YuiH	99	84	85	94
KFAV_v1_0524	KFAV_v1_0524	conserved protein of unknown function	99	84	85	94
KFAV_v1_0228	KFAV_v1_0228	conserved protein of unknown function	99	84	84	94
panB	KFAV_v1_1719	ketopantoate hydroxymethyltransferase	99	84	84	94
KFAV_v1_1883	KFAV_v1_1883	conserved protein of unknown function	99	84	84	93
pyrAA_1	KFAV_v1_0540	pyrimidine-specific carbamoyl-phosphate synthetase (small subunit, glutaminase subunit)	98	84	84	93
KFAV_v1_3466	KFAV_v1_3466	transposase	98	84	84	93
topA	KFAV_v1_1924	DNA topoisomerase I	98	84	84	93
purN	KFAV_v1_0318	Phosphoribosylglycinamide formyltransferase	98	84	84	93
pgoN_2	KFAV_v1_0754	promiscuous glyoxal/methylglyoxal reductase	97	84	84	93
gatA_2	KFAV_v1_3064	glutamyl-tRNA(Gln) amidotransferase (subunit A)	97	84	84	93
asnO	KFAV_v1_0707	Asparagine synthetase [glutamine-hydrolyzing] 3	97	84	84	93

scoB	KFAV_v1_0980	acetoacetyl CoA-transferase (subunit B)	97	84	83	93
mntH	KFAV_v1_2685	proton-coupled manganese transporter	97	84	83	93
KFAV_v1_0430	KFAV_v1_0430	Cobalamin synthesis protein P47K	97	83	83	93
gcvT	KFAV_v1_1193	aminomethyltransferase (glycine cleavage system protein T)	97	83	83	93
KFAV_v1_0457	KFAV_v1_0457	Helicase SNF2	97	83	83	93
yngB	KFAV_v1_0332	putative UTP-glucose-1-phosphate uridylyltransferase	97	83	83	93
ftsH_2	KFAV_v1_1937	ATP-dependent zinc metalloprotease FtsH	97	82	83	93
ddl	KFAV_v1_2334	D-alanine--D-alanine ligase	97	82	83	93
ligA	KFAV_v1_3067	DNA ligase (NAD-dependent)	97	82	83	93
KFAV_v1_0969	KFAV_v1_0969	conserved protein of unknown function	96	82	83	93
KFAV_v1_1074	KFAV_v1_1074	conserved protein of unknown function	96	82	83	92
KFAV_v1_1960	KFAV_v1_1960	protein of unknown function	96	82	82	92
ndx	KFAV_v1_2119	Diadenosine hexaphosphate hydrolase	96	82	82	92
KFAV_v1_2333	KFAV_v1_2333	conserved exported protein of unknown function	96	82	82	92
KFAV_v1_2787	KFAV_v1_2787	Nitroreductase	96	82	82	92
glcF_2	KFAV_v1_2866	glycolate oxidase (iron-sulfur subunit)	96	82	82	92
KFAV_v1_0556	KFAV_v1_0556	cell-division ABC transporter (modular protein)	96	82	81	92
thrZ	KFAV_v1_2530	threonyl-tRNA synthetase	96	82	81	92
KFAV_v1_1954	KFAV_v1_1954	NAD(P)H dehydrogenase (quinone)	96	82	81	92
yhbH_1	KFAV_v1_0869	factor involved in shape determination	96	81	81	92
KFAV_v1_0446	KFAV_v1_0446	Aliphatic sulfonates family ABC transporter, periplasmic ligand-binding protein	96	81	81	92
KFAV_v1_3062	KFAV_v1_3062	conserved protein of unknown function	96	81	81	92
KFAV_v1_0087	KFAV_v1_0087	conserved membrane protein of unknown function	95	81	81	92
KFAV_v1_1763	KFAV_v1_1763	conserved protein of unknown function	95	81	81	92
KFAV_v1_1955	KFAV_v1_1955	conserved protein of unknown function	95	81	81	92
KFAV_v1_0528	KFAV_v1_0528	conserved protein of unknown function	95	81	81	91
KFAV_v1_1004	KFAV_v1_1004	Enoyl-CoA hydratase	95	81	81	91
KFAV_v1_0496	KFAV_v1_0496	conserved protein of unknown function	95	81	80	91
KFAV_v1_2904	KFAV_v1_2904	Cytochrome C assembly protein	95	81	80	91
spoVE_1	KFAV_v1_0897	Stage V sporulation protein E	95	80	80	91
KFAV_v1_2207	KFAV_v1_2207	conserved protein of unknown function	95	80	80	91
gcvPB	KFAV_v1_1195	glycine decarboxylase (subunit 2) (glycine cleavage system protein P)	95	80	80	91
fabHA	KFAV_v1_0043	beta-ketoacyl-acyl carrier protein synthase III 1	95	80	80	91
KFAV_v1_3412	KFAV_v1_3412	conserved protein of unknown function	95	80	80	91
KFAV_v1_2675	KFAV_v1_2675	transposase	95	80	80	91
fliE	KFAV_v1_1916	Flagellar hook-basal body complex protein FliE	94	80	80	90
KFAV_v1_3111	KFAV_v1_3111	conserved protein of unknown function	94	80	80	90
KFAV_v1_0533	KFAV_v1_0533	conserved protein of unknown function	94	80	80	90
frlP	KFAV_v1_2584	fructose-amino acid ABC transporter (ATP-binding subunit)	94	80	80	90
KFAV_v1_1627	KFAV_v1_1627	DNA helicase	94	80	80	90
KFAV_v1_1217	KFAV_v1_1217	N(6)-L-threonylcarbamoyladenine synthase	94	80	80	90
KFAV_v1_1790	KFAV_v1_1790	conserved protein of unknown function	94	80	80	90
sseA	KFAV_v1_2668	putative 3-mercaptopyruvate sulfurtransferase	94	79	80	90
chaA	KFAV_v1_1056	H+/Ca2+ antiporter	94	79	79	89
fmdA	KFAV_v1_3101	Formamidase	94	79	79	89
KFAV_v1_1198	KFAV_v1_1198	protein of unknown function	93	79	79	89
KFAV_v1_1909	KFAV_v1_1909	putative kinesin-like protein (fragment)	93	79	79	89
KFAV_v1_2803	KFAV_v1_2803	D-isomer specific 2-hydroxyacid dehydrogenase NAD-binding protein	93	79	79	89
KFAV_v1_3334	KFAV_v1_3334	putative enzyme	93	79	79	89
KFAV_v1_0045	KFAV_v1_0045	NAD-dependent malic enzyme	93	79	79	89
KFAV_v1_3418	KFAV_v1_3418	conserved protein of unknown function	93	79	79	89
KFAV_v1_3279	KFAV_v1_3279	2-dehydropantoate 2-reductase	93	79	78	89

KFAV_v1_3406	KFAV_v1_3406	conserved exported protein of unknown function	93	79	78	89
KFAV_v1_3598	KFAV_v1_3598	protein of unknown function	93	79	78	89
aroE	KFAV_v1_2166	Shikimate dehydrogenase (NADP(+))	93	78	78	89
vapC	KFAV_v1_3457	Ribonuclease VapC	92	78	78	88
KFAV_v1_3234	KFAV_v1_3234	Translational regulator CsrA (modular protein)	92	78	77	88
alr	KFAV_v1_3278	Alanine racemase	92	78	77	88
KFAV_v1_0824	KFAV_v1_0824	RelA/SpoT domain protein	92	78	77	88
KFAV_v1_2666	KFAV_v1_2666	conserved protein of unknown function	92	78	77	88
mcsA	KFAV_v1_0132	activator of protein kinase McsB	92	78	77	88
ispH	KFAV_v1_0886	1-hydroxy-2-methyl-2-(E)-butenyl 4-diphosphate reductase	92	77	77	88
KFAV_v1_2116	KFAV_v1_2116	protein of unknown function	92	77	77	88
KFAV_v1_3244	KFAV_v1_3244	Metal-dependent phosphohydrolase	92	77	77	87
yeeE	KFAV_v1_3520	conserved hypothetical protein; putative inner membrane protein	91	77	77	87
KFAV_v1_1893	KFAV_v1_1893	Site-determining protein	91	77	77	87
KFAV_v1_2726	KFAV_v1_2726	HAD family hydrolase	91	77	77	87
KFAV_v1_0425	KFAV_v1_0425	transposase	91	77	77	87
KFAV_v1_2008	KFAV_v1_2008	conserved membrane protein of unknown function	91	77	77	87
KFAV_v1_1284	KFAV_v1_1284	transposase	91	76	77	87
KFAV_v1_1196	KFAV_v1_1196	conserved protein of unknown function	91	76	77	87
fabL	KFAV_v1_2850	enoyl-acyl carrier protein reductase III	91	76	77	87
KFAV_v1_3390	KFAV_v1_3390	Tfp pilus assembly protein ATPase PilM-like protein	91	76	77	87
KFAV_v1_2442	KFAV_v1_2442	Uncharacterized 15.0 kDa protein in dhaT-dhaS intergenic region	90	76	77	87
KFAV_v1_0303	KFAV_v1_0303	conserved protein of unknown function	90	76	76	86
KFAV_v1_2867	KFAV_v1_2867	conserved protein of unknown function	90	76	76	86
KFAV_v1_1232	KFAV_v1_1232	conserved protein of unknown function	90	76	76	86
spoIIR	KFAV_v1_3533	Stage II sporulation protein R	90	76	76	86
KFAV_v1_0333	KFAV_v1_0333	conserved exported protein of unknown function	90	76	76	86
KFAV_v1_2021	KFAV_v1_2021	Radical SAM protein (fragment)	90	76	76	86
KFAV_v1_1774	KFAV_v1_1774	putative enzyme	90	76	76	86
hpcC	KFAV_v1_1840	5-carboxymethyl-2-hydroxymuconate semialdehyde dehydrogenase	90	76	76	86
KFAV_v1_1073	KFAV_v1_1073	protein of unknown function	90	75	76	85
KFAV_v1_3246	KFAV_v1_3246	conserved protein of unknown function	90	75	76	85
KFAV_v1_3265	KFAV_v1_3265	conserved protein of unknown function	89	75	75	85
KFAV_v1_0100	KFAV_v1_0100	conserved membrane protein of unknown function	89	75	75	85
yjkA	KFAV_v1_2993	putative ABC transporter (permease)	89	75	75	85
spoVE_2	KFAV_v1_1548	factor for spore cortex peptidoglycan synthesis (stage V sporulation)	89	75	75	85
codA	KFAV_v1_3311	cytosine deaminase	89	75	75	85
dprE	KFAV_v1_3416	Decaprenylphosphoryl-beta-D-ribose oxidase	89	75	75	84
purE	KFAV_v1_0310	N5-carboxyaminoimidazole ribonucleotide mutase	89	75	75	84
KFAV_v1_0122	KFAV_v1_0122	conserved protein of unknown function	89	75	75	84
KFAV_v1_2013	KFAV_v1_2013	conserved protein of unknown function	89	75	75	84
proH	KFAV_v1_1097	Pyrroline-5-carboxylate reductase 1	89	75	75	84
KFAV_v1_1282	KFAV_v1_1282	conserved protein of unknown function	89	75	75	84
KFAV_v1_2727	KFAV_v1_2727	Phosphorylase	89	75	75	84
KFAV_v1_2452	KFAV_v1_2452	putative transcriptional regulator, PucR family	89	74	75	84
KFAV_v1_2626	KFAV_v1_2626	conserved protein of unknown function	89	74	75	84
KFAV_v1_0786	KFAV_v1_0786	Biotin carboxyl carrier protein of acetyl-CoA carboxylase	89	74	75	84
yjbE_2	KFAV_v1_2999	Uncharacterized membrane protein YjbE	88	74	74	84
pepA_1	KFAV_v1_1613	fragment of cytosol aminopeptidase (part 1)	88	74	74	84
KFAV_v1_0416	KFAV_v1_0416	DNA-binding protein	88	74	74	84
KFAV_v1_1628	KFAV_v1_1628	protein of unknown function	88	74	74	83
KFAV_v1_1698	KFAV_v1_1698	High-affinity branched-chain amino acid transport ATP-binding protein LivG (fragment)	88	74	74	83

KFAV_v1_0206	KFAV_v1_0206	conserved protein of unknown function	87	74	74	83
KFAV_v1_2859	KFAV_v1_2859	conserved membrane protein of unknown function	87	74	74	83
KFAV_v1_1910	KFAV_v1_1910	protein of unknown function	87	74	74	83
ruvA	KFAV_v1_2416	Holliday junction ATP-dependent DNA helicase RuvA	87	73	74	83
KFAV_v1_1103	KFAV_v1_1103	conserved exported protein of unknown function	87	73	74	83
paaG_2	KFAV_v1_3071	fragment of acyl-CoA hydratase (part 2)	87	73	74	83
KFAV_v1_3251	KFAV_v1_3251	Amidophosphoribosyltransferase-like protein	87	73	74	83
nodI	KFAV_v1_0767	Nod factor export ATP-binding protein I	87	73	73	83
rpe	KFAV_v1_1991	ribulose-5-phosphate 3-epimerase	87	73	73	83
KFAV_v1_0366	KFAV_v1_0366	protein of unknown function	87	73	73	83
arsF_1	KFAV_v1_0947	fragment of arsenite/antimonite/H+ antiporter (part 3)	87	73	73	83
ruvC	KFAV_v1_2417	component of RuvABC resolvasome, endonuclease	87	73	73	82
KFAV_v1_0398	KFAV_v1_0398	conserved membrane protein of unknown function	86	73	73	82
KFAV_v1_1090	KFAV_v1_1090	transposase	86	73	73	82
KFAV_v1_0758	KFAV_v1_0758	protein of unknown function	86	73	73	82
KFAV_v1_1729	KFAV_v1_1729	protein of unknown function	86	73	73	82
KFAV_v1_1787	KFAV_v1_1787	SAM-dependent methyltransferase	86	72	73	82
KFAV_v1_3030	KFAV_v1_3030	protein of unknown function	86	72	73	82
KFAV_v1_3407	KFAV_v1_3407	Endonuclease, Uma2 family (Restriction endonuclease fold)	86	72	73	81
KFAV_v1_1352	KFAV_v1_1352	Acyl-CoA dehydrogenase	86	72	73	81
floA	KFAV_v1_1252	flotillin-like protein involved in membrane lipid rafts	86	72	72	81
ftsY	KFAV_v1_1969	signal recognition particle (docking protein)	86	72	72	81
rnhA	KFAV_v1_2768	Ribonuclease H	86	72	72	81
KFAV_v1_2004	KFAV_v1_2004	YicC family protein	85	71	72	81
mutS	KFAV_v1_1789	DNA mismatch repair recognition factor	85	71	72	81
KFAV_v1_0390	KFAV_v1_0390	O-antigen transporter	85	71	72	81
ymfH	KFAV_v1_2308	putative processing protease	85	71	72	81
fabF_2	KFAV_v1_2322	3-oxoacyl-[acyl-carrier-protein] synthase 2	85	71	72	81
coxM_1	KFAV_v1_2540	Putative carbon monoxide dehydrogenase medium chain	85	71	72	81
pyrF	KFAV_v1_2050	orotidine 5'-phosphate decarboxylase	85	71	72	81
KFAV_v1_1183	KFAV_v1_1183	conserved protein of unknown function	85	71	72	81
KFAV_v1_2508	KFAV_v1_2508	protein of unknown function	85	71	72	80
ftsE	KFAV_v1_0555	cell-division signal transducer (ATP-binding protein)	85	71	72	80
KFAV_v1_0623	KFAV_v1_0623	protein of unknown function	85	71	72	80
purM	KFAV_v1_0317	phosphoribosylaminoimidazole synthetase	85	71	72	80
ripX	KFAV_v1_2070	site-specific tyrosine recombinase for chromosome partitioning	85	71	72	80
murE	KFAV_v1_1545	UDP-N-acetylmuramoylalanyl-D-glutamate-2,6-diaminopimelate ligase	85	70	72	80
KFAV_v1_1177	KFAV_v1_1177	conserved membrane protein of unknown function	84	70	72	80
tatC	KFAV_v1_0241	Sec-independent protein translocase protein TatCd	84	70	72	80
KFAV_v1_0677	KFAV_v1_0677	transposase	84	70	71	80
KFAV_v1_2662	KFAV_v1_2662	Iron ABC transporter	84	70	71	80
baeR	KFAV_v1_1453	DNA-binding response regulator in two-component regulatory system with BaeS	84	70	71	80
lutB	KFAV_v1_2610	component of an iron-sulfur oxidase linked to L-lactate utilization	84	70	71	80
yfcH	KFAV_v1_1499	Epimerase family protein YfcH	84	70	71	79
KFAV_v1_0914	KFAV_v1_0914	Alanine racemase	84	70	71	79
KFAV_v1_1959	KFAV_v1_1959	protein of unknown function	84	70	71	79
dnaJ_2	KFAV_v1_2142	fragment of co-factor of molecular chaperone (part 1)	84	70	71	79
KFAV_v1_1684	KFAV_v1_1684	conserved protein of unknown function	84	70	71	79
secDF_2	KFAV_v1_2403	fragment of protein-export membrane protein (part 1)	84	70	71	79
KFAV_v1_3546	KFAV_v1_3546	conserved membrane protein of unknown function	83	70	71	79
KFAV_v1_0558	KFAV_v1_0558	conserved membrane protein of unknown function	83	70	71	79
KFAV_v1_0860	KFAV_v1_0860	conserved membrane protein of unknown function	83	70	71	79

KFAV_v1_2932	KFAV_v1_2932	conserved protein of unknown function	83	69	71	79
pxpB_2	KFAV_v1_3085	L-5-oxoprolinase (ATP-dependent) subunit B	83	69	70	78
KFAV_v1_1381	KFAV_v1_1381	protein of unknown function	83	69	70	78
KFAV_v1_3391	KFAV_v1_3391	Fimbrial assembly family protein	83	69	70	78
KFAV_v1_0275	KFAV_v1_0275	DNA-binding transcriptional regulator, GntR family	83	69	70	78
ampS	KFAV_v1_0584	Aminopeptidase AmpS	83	69	70	78
ymfI	KFAV_v1_1534	Uncharacterized oxidoreductase YmfI	82	69	70	78
ccpN	KFAV_v1_1265	negative regulator of gluconeogenesis	82	69	70	78
KFAV_v1_2542	KFAV_v1_2542	ATPase	82	69	70	78
KFAV_v1_2952	KFAV_v1_2952	protein of unknown function	82	69	70	77
KFAV_v1_2894	KFAV_v1_2894	putative 4-hydroxy-4-methyl-2-oxoglutarate aldolase	82	69	70	77
KFAV_v1_1051	KFAV_v1_1051	protein of unknown function	81	69	69	77
KFAV_v1_1168	KFAV_v1_1168	Gfo/Idh/MocA family oxidoreductase	81	69	69	77
cas1_1	KFAV_v1_2965	CRISPR-associated endonuclease Cas1 2	81	69	69	77
mcsB	KFAV_v1_0133	protein arginine kinase	81	69	69	77
dnaC	KFAV_v1_3586	replicative DNA helicase	81	69	69	77
ldeJ_1	KFAV_v1_1271	isovaleryl-CoA dehydrogenase (leucine degradation)	81	69	69	77
KFAV_v1_0021	KFAV_v1_0021	Phospholipid phosphatase	81	69	69	77
qoxD	KFAV_v1_2235	cytochrome aa3-600 quinol oxidase (subunit IV)	81	69	69	77
KFAV_v1_3006	KFAV_v1_3006	conserved protein of unknown function	81	68	69	77
KFAV_v1_3074	KFAV_v1_3074	conserved protein of unknown function	81	68	69	77
yfkJ	KFAV_v1_2804	Low molecular weight protein-tyrosine-phosphatase YfkJ	80	68	69	77
KFAV_v1_0130	KFAV_v1_0130	TetR/AcrR family transcriptional regulator	80	68	68	76
fadR	KFAV_v1_0856	transcriptional regulator of fatty acids degradation [FadR-long-chain (C14-C20) acyl-CoAs]	80	68	68	76
rlmG	KFAV_v1_0153	23S rRNA m2G1835 methyltransferase	80	68	68	76
KFAV_v1_3069	KFAV_v1_3069	conserved protein of unknown function	80	68	68	76
KFAV_v1_1722	KFAV_v1_1722	conserved protein of unknown function	79	68	68	76
prkA_1	KFAV_v1_0868	serine protein kinase (involved in sporulation)	79	68	68	76
KFAV_v1_2972	KFAV_v1_2972	protein of unknown function	79	68	68	76
hisB	KFAV_v1_0573	imidazoleglycerol-phosphate dehydratase [Mn(II)-dependent]	79	68	68	75
pdp	KFAV_v1_2011	pyrimidine-nucleoside phosphorylase	79	68	68	75
KFAV_v1_1972	KFAV_v1_1972	conserved exported protein of unknown function	79	67	68	75
cheR	KFAV_v1_1668	methyl-accepting chemotaxis proteins (MCPs) methyltransferase	79	67	68	75
metN	KFAV_v1_1410	methionine ABC transporter (ATP-binding protein)	79	67	68	75
ldeE_3	KFAV_v1_1681	fragment of methylcrotonoyl-CoA carboxylase subunit (leucine degradation) (part 2)	79	67	67	75
panC	KFAV_v1_1720	pantothenate synthetase	79	67	67	75
mutSB	KFAV_v1_2518	homologous recombination factor	79	67	67	75
KFAV_v1_3414	KFAV_v1_3414	membrane protein of unknown function	79	67	67	75
KFAV_v1_1253	KFAV_v1_1253	conserved protein of unknown function	78	67	67	75
KFAV_v1_2821	KFAV_v1_2821	protein of unknown function	78	67	67	75
pgdA	KFAV_v1_0432	Peptidoglycan deacetylase	78	67	67	75
mltG	KFAV_v1_2230	Endolytic murein transglycosylase	78	67	67	75
KFAV_v1_0227	KFAV_v1_0227	conserved protein of unknown function	78	67	67	75
dtd	KFAV_v1_2393	gly-tRNA(Ala) deacylase / D-Tyr-tRNATyr deacylase	78	66	67	75
KFAV_v1_3408	KFAV_v1_3408	conserved exported protein of unknown function	78	66	67	75
rplI	KFAV_v1_3587	ribosomal protein L9	78	66	67	74
KFAV_v1_1238	KFAV_v1_1238	conserved protein of unknown function	78	66	67	74
KFAV_v1_0477	KFAV_v1_0477	conserved protein of unknown function	78	66	67	74
KFAV_v1_0759	KFAV_v1_0759	Aldo/keto reductase	78	66	66	74
KFAV_v1_0895	KFAV_v1_0895	AEC family transporter	78	66	66	74
ahcY	KFAV_v1_0225	Adenosylhomocysteinase	78	66	66	74
KFAV_v1_1251	KFAV_v1_1251	conserved membrane protein of unknown function	78	66	66	74

pheS	KFAV_v1_2522	phenylalanyl-tRNA synthetase (alpha subunit)	78	66	66	74
icmF	KFAV_v1_2323	Isobutyryl-CoA mutase / P-loop GTPase	78	66	66	74
KFAV_v1_0634	KFAV_v1_0634	Class II aldolase/adducin family protein	78	65	66	74
fadE	KFAV_v1_2318	acyl-CoA dehydrogenase (FAD dependent)	78	65	66	74
KFAV_v1_3582	KFAV_v1_3582	conserved protein of unknown function	78	65	66	73
KFAV_v1_3602	KFAV_v1_3602	conserved protein of unknown function	78	65	66	73
smc	KFAV_v1_1970	Chromosome partition protein Smc	77	65	66	73
thiO	KFAV_v1_0924	Glycine oxidase ThiO	77	65	66	72
ltrA_7	KFAV_v1_2504	Group II intron reverse transcriptase/maturase	77	65	65	72
KFAV_v1_1181	KFAV_v1_1181	protein of unknown function	77	65	65	72
KFAV_v1_1389	KFAV_v1_1389	CoA-binding protein	77	65	65	72
KFAV_v1_1839	KFAV_v1_1839	ABC transporter substrate-binding protein	77	65	65	72
KFAV_v1_2414	KFAV_v1_2414	conserved protein of unknown function	77	65	65	72
KFAV_v1_2555	KFAV_v1_2555	Thymidylate synthase	77	65	65	72
dnaN	KFAV_v1_0003	DNA polymerase III (beta subunit)	77	65	65	72
KFAV_v1_1683	KFAV_v1_1683	AMP-dependent synthetase	76	65	65	72
KFAV_v1_0290	KFAV_v1_0290	conserved protein of unknown function	76	65	65	72
KFAV_v1_0085	KFAV_v1_0085	Flippase	76	65	65	72
hepT	KFAV_v1_1665	heptaprenyl diphosphate synthase component II	76	64	64	72
KFAV_v1_1765	KFAV_v1_1765	protein of unknown function	76	64	64	72
KFAV_v1_2335	KFAV_v1_2335	UDP-N-acetylmuramoyl-tripeptide--D-alanyl-D-alanine ligase	76	64	64	72
ykoX	KFAV_v1_0020	Uncharacterized membrane protein YkoX	76	64	64	72
KFAV_v1_1780	KFAV_v1_1780	Branched-chain amino acid ABC transporter permease	75	64	64	72
mfd	KFAV_v1_0078	transcription-repair coupling factor	75	64	64	72
KFAV_v1_1178	KFAV_v1_1178	conserved protein of unknown function	75	64	64	72
yfkA	KFAV_v1_1231	putative protein YfkA	75	64	64	72
KFAV_v1_0977	KFAV_v1_0977	Aminotransferase class-III	75	64	64	71
KFAV_v1_0915	KFAV_v1_0915	conserved protein of unknown function	75	64	63	71
KFAV_v1_1387	KFAV_v1_1387	conserved membrane protein of unknown function	75	64	63	71
KFAV_v1_1566	KFAV_v1_1566	RNA-binding protein	75	63	63	71
KFAV_v1_2703	KFAV_v1_2703	conserved protein of unknown function	74	63	63	71
KFAV_v1_2146	KFAV_v1_2146	Oxygen-independent coproporphyrinogen-III oxidase-like protein	74	63	63	71
KFAV_v1_2891	KFAV_v1_2891	Transporter (fragment)	74	63	63	71
KFAV_v1_3471	KFAV_v1_3471	Lysine decarboxylase (fragment)	74	63	63	71
KFAV_v1_2701	KFAV_v1_2701	Sulfur reduction protein DsrE	74	63	63	71
KFAV_v1_2762	KFAV_v1_2762	Coenzyme A pyrophosphatase	74	63	63	70
KFAV_v1_2797	KFAV_v1_2797	conserved protein of unknown function	74	63	63	70
KFAV_v1_3464	KFAV_v1_3464	transposase	74	63	63	70
KFAV_v1_3292	KFAV_v1_3292	PAS domain S-box-containing protein	74	63	63	70
KFAV_v1_0481	KFAV_v1_0481	conserved membrane protein of unknown function	74	63	63	70
KFAV_v1_2336	KFAV_v1_2336	Alpha/beta hydrolase fold protein	74	62	63	70
KFAV_v1_2045	KFAV_v1_2045	putative metabolite transport protein HI_1104	74	62	63	70
nudF	KFAV_v1_2077	isopentenyl pyrophosphate and dimethylallyl pyrophosphate diphosphatase (moonlighting ADP-ribose pyrophosphatase)	74	62	63	70
KFAV_v1_2368	KFAV_v1_2368	conserved protein of unknown function	74	62	63	70
KFAV_v1_0732	KFAV_v1_0732	ATP-dependent acyl-CoA ligase	74	62	63	70
polA	KFAV_v1_2631	DNA polymerase I	73	62	63	70
KFAV_v1_0704	KFAV_v1_0704	protein of unknown function	73	62	63	70
speA	KFAV_v1_0902	arginine decarboxylase	73	62	62	70
trmL	KFAV_v1_2728	tRNA (cytidine(34)-2'-O)-methyltransferase	73	62	62	70
KFAV_v1_1012	KFAV_v1_1012	Sarcosine oxidase subunit beta	73	62	62	70
KFAV_v1_0764	KFAV_v1_0764	Glycosyl transferase family 1	73	62	62	70
KFAV_v1_0766	KFAV_v1_0766	GntR family transcriptional regulator	73	62	62	69

flhB	KFAV_v1_1896	component of the flagellar export machinery	73	62	62	69
KFAV_v1_2828	KFAV_v1_2828	protein of unknown function	73	62	62	69
ydaO	KFAV_v1_3249	Uncharacterized amino acid permease YdaO	73	62	62	69
KFAV_v1_2767	KFAV_v1_2767	Epoxyqueuosine reductase	72	62	62	69
KFAV_v1_2501	KFAV_v1_2501	conserved protein of unknown function	72	62	62	69
pgk_1	KFAV_v1_0607	phosphoglycerate kinase	72	61	62	69
coaD	KFAV_v1_1984	phosphopantetheine adenylyltransferase	72	61	62	69
KFAV_v1_1712	KFAV_v1_1712	conserved membrane protein of unknown function	72	61	62	69
cwlM	KFAV_v1_2312	N-acetylmuramoyl-L-alanine amidase CwlM	72	61	62	69
KFAV_v1_0035	KFAV_v1_0035	conserved protein of unknown function	72	61	62	69
mhpD	KFAV_v1_2447	2-keto-4-pentenoate hydratase	72	61	62	69
KFAV_v1_0878	KFAV_v1_0878	Ribonucleoside-diphosphate reductase	72	61	62	68
KFAV_v1_1857	KFAV_v1_1857	conserved protein of unknown function	72	61	61	68
tpiA	KFAV_v1_0608	triose phosphate isomerase	72	61	61	68
KFAV_v1_3199	KFAV_v1_3199	transposase (fragment)	72	61	61	68
KFAV_v1_0675	KFAV_v1_0675	protein of unknown function	71	61	61	68
KFAV_v1_1077	KFAV_v1_1077	conserved protein of unknown function	71	61	61	68
KFAV_v1_1986	KFAV_v1_1986	conserved protein of unknown function	71	61	61	68
ltrA_4	KFAV_v1_2360	Group II intron reverse transcriptase/maturase	71	61	61	68
KFAV_v1_2534	KFAV_v1_2534	conserved protein of unknown function	71	61	61	68
KFAV_v1_2213	KFAV_v1_2213	transposase	71	61	61	68
KFAV_v1_1244	KFAV_v1_1244	conserved exported protein of unknown function	71	61	61	68
KFAV_v1_1075	KFAV_v1_1075	Pyridine nucleotide-disulfide oxidoreductase	71	61	61	68
iscSB	KFAV_v1_1515	cysteine desulfurase	71	61	61	68
cheD	KFAV_v1_1888	sequence specific deamidase required for methylation of methyl-accepting chemotaxis proteins (MCPs) by CheR	71	61	61	68
gluQ	KFAV_v1_2658	Glutamyl-Q tRNA(Asp) synthetase	71	61	61	68
KFAV_v1_0789	KFAV_v1_0789	Allophanate hydrolase	71	61	61	68
pupG	KFAV_v1_2068	purine nucleoside phosphorylase	71	61	61	68
ugtP	KFAV_v1_3102	Processive diacylglycerol beta-glucosyltransferase	71	60	61	68
metP	KFAV_v1_1409	methionine ABC transporter, permease component	71	60	61	67
KFAV_v1_2558	KFAV_v1_2558	conserved protein of unknown function	71	60	61	67
KFAV_v1_0825	KFAV_v1_0825	B12-binding domain-containing radical SAM protein (modular protein)	71	60	61	67
KFAV_v1_0583	KFAV_v1_0583	protein of unknown function	70	60	61	67
KFAV_v1_1149	KFAV_v1_1149	conserved protein of unknown function	70	60	61	67
bkdAB_1	KFAV_v1_2085	branched-chain alpha-keto acid dehydrogenase E1 subunit	70	60	60	67
artQ	KFAV_v1_0983	high affinity arginine ABC transporter (permease)	70	60	60	67
KFAV_v1_2278	KFAV_v1_2278	AI-2E family transporter	70	60	60	67
KFAV_v1_3480	KFAV_v1_3480	Flagellar basal body protein	70	60	60	67
KFAV_v1_3221	KFAV_v1_3221	conserved protein of unknown function	70	60	60	67
KFAV_v1_3453	KFAV_v1_3453	transposase (fragment)	70	60	60	67
KFAV_v1_1240	KFAV_v1_1240	conserved protein of unknown function	70	60	60	67
KFAV_v1_2245	KFAV_v1_2245	AMP-dependent synthetase and ligase	70	60	60	67
KFAV_v1_0096	KFAV_v1_0096	conserved protein of unknown function	70	60	60	66
KFAV_v1_2436	KFAV_v1_2436	putative Transcriptional regulator, LysR family	70	60	60	66
KFAV_v1_0380	KFAV_v1_0380	conserved protein of unknown function	70	60	60	66
KFAV_v1_0532	KFAV_v1_0532	Sporulation protein (fragment)	70	60	60	66
KFAV_v1_0744	KFAV_v1_0744	conserved protein of unknown function	70	59	60	66
KFAV_v1_2585	KFAV_v1_2585	conserved protein of unknown function	70	59	60	66
KFAV_v1_3382	KFAV_v1_3382	Type II secretion system F domain protein (fragment)	70	59	60	66
speE_2	KFAV_v1_3562	spermidine synthase; polyamine metabolism	70	59	60	66
lytT_2	KFAV_v1_1112	fragment of two-component response regulator [LytS] (part 2)	69	59	60	66
KFAV_v1_1798	KFAV_v1_1798	protein of unknown function	69	59	60	66

ytvI	KFAV_v1_2852	Sporulation integral membrane protein YtvI	69	59	60	66
mntR	KFAV_v1_2161	Transcriptional regulator MntR	69	59	59	66
KFAV_v1_1009	KFAV_v1_1009	putative spermidine/putrescine transport system permease protein	69	59	59	66
KFAV_v1_2925	KFAV_v1_2925	protein of unknown function	69	59	59	66
KFAV_v1_0661	KFAV_v1_0661	conserved protein of unknown function	69	59	59	65
KFAV_v1_2724	KFAV_v1_2724	conserved exported protein of unknown function	69	59	59	65
KFAV_v1_0482	KFAV_v1_0482	conserved protein of unknown function	68	58	59	65
rimI	KFAV_v1_0238	Ribosomal-protein-alanine N-acetyltransferase	68	58	59	65
KFAV_v1_0622	KFAV_v1_0622	AAA family ATPase	68	58	59	65
whiA	KFAV_v1_0594	putative morphogen	68	58	59	65
KFAV_v1_0378	KFAV_v1_0378	Diguanylate phosphodiesterase	68	58	59	65
KFAV_v1_3290	KFAV_v1_3290	conserved protein of unknown function	68	58	59	65
ppdK	KFAV_v1_1267	Pyruvate, phosphate dikinase	68	58	59	65
KFAV_v1_0285	KFAV_v1_0285	protein of unknown function	68	58	59	65
KFAV_v1_0456	KFAV_v1_0456	Amidohydrolase	68	58	59	65
KFAV_v1_0944	KFAV_v1_0944	ABC transporter	67	58	58	65
KFAV_v1_2627	KFAV_v1_2627	Lytic transglycosylase	67	58	58	65
KFAV_v1_0350	KFAV_v1_0350	SNF2-related protein	67	58	58	65
ldeG	KFAV_v1_1536	hydroxymethylglutaryl-CoA lyase (leucine degradation)	67	58	58	65
KFAV_v1_1017	KFAV_v1_1017	conserved protein of unknown function	67	58	58	65
KFAV_v1_2404	KFAV_v1_2404	conserved protein of unknown function	67	57	58	65
KFAV_v1_3398	KFAV_v1_3398	conserved membrane protein of unknown function	67	57	58	64
ald_1	KFAV_v1_2072	L-alanine dehydrogenase (NAD-dependent)	67	57	57	64
KFAV_v1_0046	KFAV_v1_0046	Arginine decarboxylase	67	57	57	64
mhpE	KFAV_v1_2445	4-hyroxy-2-oxovalerate/4-hydroxy-2-oxopentanoic acid aldolase, class I	67	57	57	64
KFAV_v1_1234	KFAV_v1_1234	DedA family protein	67	57	57	64
KFAV_v1_1008	KFAV_v1_1008	putative spermidine/putrescine transport system permease protein	67	57	57	64
KFAV_v1_0806	KFAV_v1_0806	conserved membrane protein of unknown function	67	57	57	64
xpsE	KFAV_v1_3381	Type II secretion system protein E	66	57	57	64
minC	KFAV_v1_1156	putative septum site-determining protein MinC	66	57	57	64
leuC	KFAV_v1_2581	3-isopropylmalate dehydratase (large subunit)	66	57	57	64
KFAV_v1_2451	KFAV_v1_2451	Oxidoreductase FAD/NAD(P)-binding domain protein	66	57	57	64
KFAV_v1_3604	KFAV_v1_3604	conserved membrane protein of unknown function	66	57	57	64
KFAV_v1_3525	KFAV_v1_3525	protein of unknown function	66	57	57	64
glxK	KFAV_v1_3540	D-glycerate kinase	66	57	57	63
motA	KFAV_v1_0559	motility protein A; MotA component of the H+-coupled stator flagellum complex	66	57	56	63
KFAV_v1_1816	KFAV_v1_1816	conserved protein of unknown function	66	57	56	63
mog	KFAV_v1_2090	Molybdopterin adenylyltransferase	66	56	56	63
KFAV_v1_1080	KFAV_v1_1080	DNA-binding transcriptional regulator, MurR/RpiR family, contains HTH and SIS domains	66	56	56	63
KFAV_v1_1828	KFAV_v1_1828	conserved protein of unknown function	66	56	56	63
prmC	KFAV_v1_3534	Release factor glutamine methyltransferase	66	56	56	63
KFAV_v1_3397	KFAV_v1_3397	conserved protein of unknown function	65	56	56	63
KFAV_v1_0124	KFAV_v1_0124	conserved protein of unknown function	65	56	56	63
KFAV_v1_1615	KFAV_v1_1615	conserved protein of unknown function	65	56	56	63
KFAV_v1_2466	KFAV_v1_2466	protein of unknown function	65	56	56	62
KFAV_v1_3463	KFAV_v1_3463	conserved protein of unknown function	65	56	56	62
KFAV_v1_3267	KFAV_v1_3267	conserved exported protein of unknown function	65	56	56	62
KFAV_v1_1726	KFAV_v1_1726	Glycosidase	65	56	56	62
KFAV_v1_0483	KFAV_v1_0483	conserved protein of unknown function	65	56	56	62
ldeHA	KFAV_v1_0714	biotin carboxylase for subunit LdeHB of methylcrotonyl-CoA carboxylase	65	56	56	62
KFAV_v1_3417	KFAV_v1_3417	conserved membrane protein of unknown function	64	56	56	62
KFAV_v1_1301	KFAV_v1_1301	SAM-dependent methyltransferase	64	56	56	62

nuoJ_2	KFAV_v1_3496	NADH-quinone oxidoreductase subunit J	64	56	56	62
gcvPA	KFAV_v1_1194	glycine decarboxylase (subunit 1) (glycine cleavage system protein P)	64	56	56	62
KFAV_v1_0529	KFAV_v1_0529	conserved protein of unknown function	64	56	56	62
yyaC	KFAV_v1_3601	Spore protease YyaC	64	56	56	61
KFAV_v1_2375	KFAV_v1_2375	conserved protein of unknown function	64	56	55	61
argI	KFAV_v1_0201	arginase	64	55	55	61
KFAV_v1_1259	KFAV_v1_1259	Diacylglycerol kinase	64	55	55	61
KFAV_v1_3524	KFAV_v1_3524	Low molecular weight phosphatase family protein	64	55	55	61
KFAV_v1_0256	KFAV_v1_0256	Antitoxin	63	55	55	61
KFAV_v1_3589	KFAV_v1_3589	Pyruvate oxidase	63	55	55	61
KFAV_v1_0525	KFAV_v1_0525	ATPase	63	55	55	61
KFAV_v1_1078	KFAV_v1_1078	Sensor histidine kinase	63	55	55	61
KFAV_v1_1307	KFAV_v1_1307	conserved protein of unknown function	63	55	55	61
mtnE	KFAV_v1_2997	methionine-glutamine aminotransferase	63	55	55	61
KFAV_v1_2341	KFAV_v1_2341	conserved exported protein of unknown function	63	55	55	61
KFAV_v1_2786	KFAV_v1_2786	conserved protein of unknown function	63	55	55	61
flhF	KFAV_v1_1894	Flagellar biosynthesis protein FlhF	63	55	55	61
mmgA_2	KFAV_v1_3557	degradative acetoacetyl-CoA thiolase	63	55	55	61
KFAV_v1_0808	KFAV_v1_0808	ABC transporter	63	55	55	61
potA_1	KFAV_v1_1007	Spermidine/putrescine import ATP-binding protein PotA	63	55	55	60
phoP_2	KFAV_v1_2755	Alkaline phosphatase synthesis transcriptional regulatory protein PhoP	63	55	55	60
remB	KFAV_v1_0006	regulator of extracellular matrix formation	63	55	55	60
rsmG	KFAV_v1_3609	7-methylguanosine methyltransferase (16S rRNA, nucleotide G527)	63	54	54	60
KFAV_v1_0848	KFAV_v1_0848	Cysteine hydrolase	62	54	54	60
KFAV_v1_1522	KFAV_v1_1522	conserved protein of unknown function	62	54	54	60
rluD	KFAV_v1_2059	pseudouridylate synthase	62	54	54	60
KFAV_v1_0069	KFAV_v1_0069	Nucleotide-diphospho-sugar transferase	62	54	54	60
sigI	KFAV_v1_2413	RNA polymerase sigma factor (heat stress responsive)	62	54	54	59
nuoI_2	KFAV_v1_3497	NADH-quinone oxidoreductase subunit I	62	54	54	59
KFAV_v1_1832	KFAV_v1_1832	conserved protein of unknown function	62	54	54	59
KFAV_v1_2766	KFAV_v1_2766	conserved protein of unknown function	62	54	54	59
yhbJ_3	KFAV_v1_3427	putative membrane fusion protein; putative exporter subunit (benzoate transcriptome)	62	54	54	59
yfnC	KFAV_v1_3491	Uncharacterized MFS-type transporter YfnC	62	54	54	59
KFAV_v1_1521	KFAV_v1_1521	conserved exported protein of unknown function	61	54	54	59
KFAV_v1_2399	KFAV_v1_2399	DinB family protein	61	54	53	59
KFAV_v1_2951	KFAV_v1_2951	conserved protein of unknown function	61	54	53	59
KFAV_v1_0334	KFAV_v1_0334	protein of unknown function	61	54	53	59
KFAV_v1_2066	KFAV_v1_2066	conserved protein of unknown function	61	53	53	59
KFAV_v1_2773	KFAV_v1_2773	conserved protein of unknown function	61	53	53	59
glyS	KFAV_v1_1264	glycyl-tRNA synthetase (beta subunit)	61	53	53	59
KFAV_v1_1699	KFAV_v1_1699	leucine/isoleucine/valine transporter subunit ; ATP-binding component of ABC superfamily (fragment)	61	53	53	59
KFAV_v1_0386	KFAV_v1_0386	conserved protein of unknown function	61	53	53	59
KFAV_v1_2625	KFAV_v1_2625	Sugar kinase	61	53	53	59
ftsL	KFAV_v1_1542	Cell division protein FtsL	61	53	53	59
KFAV_v1_2929	KFAV_v1_2929	conserved protein of unknown function	61	53	53	58
KFAV_v1_0598	KFAV_v1_0598	conserved protein of unknown function	61	53	53	58
KFAV_v1_1884	KFAV_v1_1884	conserved protein of unknown function	61	53	53	58
rnmV	KFAV_v1_0057	ribonuclease M5	61	53	53	58
coxE	KFAV_v1_2541	Putative carbon monoxygenase dehydrogenase accessory protein	60	53	53	58
yecS	KFAV_v1_3082	putative transporter subunit: permease component of ABC superfamily transporter	60	53	53	58
KFAV_v1_1817	KFAV_v1_1817	conserved protein of unknown function	60	53	53	58
KFAV_v1_0418	KFAV_v1_0418	protein of unknown function	60	53	53	58

sdpR	KFAV_v1_0538	transcriptional regulator of SdpC synthesis operon (ArsR family)	60	53	53	58
ureG	KFAV_v1_0552	Urease accessory protein UreG	60	53	53	58
KFAV_v1_0936	KFAV_v1_0936	protein of unknown function	60	53	53	58
KFAV_v1_1182	KFAV_v1_1182	conserved protein of unknown function	60	52	52	58
KFAV_v1_1397	KFAV_v1_1397	conserved protein of unknown function	60	52	52	58
ydeD_1	KFAV_v1_2994	fragment of putative permease (part 2)	60	52	52	58
spo0F	KFAV_v1_3549	two-component response regulator of sporulation initiation	60	52	52	58
cysI	KFAV_v1_2243	Sulfite reductase	60	52	52	58
tcyB	KFAV_v1_0128	cystine ABC transporter (permease)	60	52	52	58
KFAV_v1_0617	KFAV_v1_0617	AMP-dependent synthetase	60	52	52	57
KFAV_v1_0433	KFAV_v1_0433	Asparaginase/glutaminase	60	52	52	57
KFAV_v1_2283	KFAV_v1_2283	conserved protein of unknown function	60	52	52	57
dmpH	KFAV_v1_2444	4-oxalocrotonate decarboxylase	60	52	52	57
KFAV_v1_0473	KFAV_v1_0473	ArsR family transcriptional regulator	60	52	52	57
KFAV_v1_3327	KFAV_v1_3327	Glutamate synthase	60	52	52	57
KFAV_v1_2280	KFAV_v1_2280	4-hydroxybenzoate octaprenyltransferase	60	52	52	57
KFAV_v1_1011	KFAV_v1_1011	Thioredoxin reductase	59	52	52	57
KFAV_v1_2663	KFAV_v1_2663	ABC transporter substrate-binding protein	59	51	52	57
KFAV_v1_1172	KFAV_v1_1172	Glycosyl transferase family 1	59	51	52	57
KFAV_v1_0951	KFAV_v1_0951	transposase (fragment)	59	51	52	56
KFAV_v1_1717	KFAV_v1_1717	CCA-adding enzyme	59	51	52	56
ytiB	KFAV_v1_2022	carbonic anhydrase	59	51	52	56
KFAV_v1_3302	KFAV_v1_3302	protein of unknown function	59	51	52	56
KFAV_v1_3430	KFAV_v1_3430	protein of unknown function	59	51	52	56
KFAV_v1_3585	KFAV_v1_3585	conserved protein of unknown function	59	51	51	56
aldHT_1	KFAV_v1_0279	Aldehyde dehydrogenase, thermostable	59	51	51	56
KFAV_v1_0788	KFAV_v1_0788	Allophanate hydrolase	59	51	51	56
KFAV_v1_1148	KFAV_v1_1148	conserved exported protein of unknown function	59	51	51	56
KFAV_v1_1015	KFAV_v1_1015	Phosphatase PAP2 family protein	59	51	51	56
hyuC_2	KFAV_v1_3313	N-carbamoyl-L-amino-acid hydrolase	59	51	51	56
KFAV_v1_1102	KFAV_v1_1102	Dioxygenase	59	51	51	55
KFAV_v1_0379	KFAV_v1_0379	protein of unknown function	58	51	51	55
KFAV_v1_1317	KFAV_v1_1317	conserved protein of unknown function	58	51	51	55
KFAV_v1_1636	KFAV_v1_1636	protein of unknown function	58	51	51	55
KFAV_v1_2798	KFAV_v1_2798	conserved protein of unknown function	58	50	51	55
KFAV_v1_2248	KFAV_v1_2248	Methyl-accepting chemotaxis sensory transducer	58	50	51	55
tcrA	KFAV_v1_2912	Transcriptional regulatory protein TcrA	58	50	51	55
KFAV_v1_0221	KFAV_v1_0221	conserved exported protein of unknown function	58	50	51	55
yitW_1	KFAV_v1_0871	Fe-S protein maturation auxiliary factor YitW	58	50	51	55
ydcM_4	KFAV_v1_3523	putative transposase	58	50	51	55
fabG_1	KFAV_v1_0465	putative enzyme	58	50	51	55
obgE	KFAV_v1_1166	ppGpp-binding GTPase involved in cell portioning, DNA repair and ribosome assembly	58	50	51	55
era	KFAV_v1_1260	maturation of 16S RNA and assembly of 30S ribosomal subunit GTPase	58	50	51	55
cggR	KFAV_v1_0605	transcriptional regulator of gapA	57	50	51	54
KFAV_v1_3454	KFAV_v1_3454	transposase (fragment)	57	50	50	54
tsaB	KFAV_v1_0237	tRNA (Adenosine(37)-N6)-threonylcarbamoyltransferase complex dimerization subunit type 1 TsaB	57	50	50	54
KFAV_v1_3485	KFAV_v1_3485	conserved protein of unknown function	57	50	50	54
KFAV_v1_0447	KFAV_v1_0447	putative ABC transporter permease protein BruAb2_1124	57	50	50	54
KFAV_v1_0712	KFAV_v1_0712	Sigma-54-dependent Fis family transcriptional regulator	57	50	50	54
KFAV_v1_0409	KFAV_v1_0409	protein of unknown function	57	50	50	54
murG	KFAV_v1_1549	UDP-N-acetylglucosamine-N-acetylmuramyl-(pentapeptide)pyrophosphoryl-undecaprenol N-acetylglucosamine transferase	57	50	50	54
KFAV_v1_2249	KFAV_v1_2249	Peptidoglycan-binding protein	57	50	50	54

KFAV_v1_2315	KFAV_v1_2315	GNAT family N-acetyltransferase	57	49	50	54
KFAV_v1_3047	KFAV_v1_3047	protein of unknown function	57	49	50	54
KFAV_v1_1357	KFAV_v1_1357	FadR family transcriptional regulator	56	49	50	54
KFAV_v1_1942	KFAV_v1_1942	conserved membrane protein of unknown function	56	49	49	54
KFAV_v1_0262	KFAV_v1_0262	Phospholipase	56	49	49	54
purH	KFAV_v1_0319	fused phosphoribosylaminoimidazole carboxy formyl formyltransferase; inosine-monophosphate cyclohydrolase	56	49	49	54
KFAV_v1_0988	KFAV_v1_0988	Sarcosine oxidase subunit alpha	56	49	49	54
KFAV_v1_2361	KFAV_v1_2361	conserved protein of unknown function	56	49	49	54
KFAV_v1_2980	KFAV_v1_2980	conserved protein of unknown function	56	49	49	54
KFAV_v1_3061	KFAV_v1_3061	protein of unknown function	56	49	49	53
KFAV_v1_0804	KFAV_v1_0804	conserved membrane protein of unknown function	56	49	49	53
KFAV_v1_2273	KFAV_v1_2273	DNA polymerase beta domain protein region	56	49	49	53
KFAV_v1_0595	KFAV_v1_0595	conserved protein of unknown function	56	49	49	53
KFAV_v1_1455	KFAV_v1_1455	conserved protein of unknown function	56	48	49	53
KFAV_v1_2301	KFAV_v1_2301	protein of unknown function	56	48	49	53
copA_1	KFAV_v1_2808	Copper-exporting P-type ATPase	56	48	49	53
KFAV_v1_2264	KFAV_v1_2264	conserved protein of unknown function	56	48	49	53
KFAV_v1_0348	KFAV_v1_0348	Sigma-54-dependent Fis family transcriptional regulator	56	48	49	53
KFAV_v1_3536	KFAV_v1_3536	conserved protein of unknown function	56	48	48	53
KFAV_v1_3588	KFAV_v1_3588	conserved protein of unknown function	56	48	48	53
KFAV_v1_0807	KFAV_v1_0807	Glycerophosphodiester phosphodiesterase	55	48	48	53
KFAV_v1_2514	KFAV_v1_2514	conserved protein of unknown function	55	48	48	53
adh_6	KFAV_v1_2351	Alcohol dehydrogenase	55	48	48	53
KFAV_v1_2138	KFAV_v1_2138	Alpha/beta hydrolase fold protein	55	48	48	53
KFAV_v1_0676	KFAV_v1_0676	DNA binding domain protein, excisionase family (fragment)	55	48	48	52
rex	KFAV_v1_1723	Redox-sensing transcriptional repressor Rex	55	48	48	52
dnaJ_1	KFAV_v1_2141	fragment of co-factor of molecular chaperone (part 2)	55	48	48	52
KFAV_v1_2307	KFAV_v1_2307	conserved protein of unknown function	55	48	48	52
KFAV_v1_2851	KFAV_v1_2851	conserved protein of unknown function	55	48	48	52
KFAV_v1_2853	KFAV_v1_2853	conserved protein of unknown function	55	48	48	52
KFAV_v1_3007	KFAV_v1_3007	conserved protein of unknown function	55	47	48	52
KFAV_v1_3459	KFAV_v1_3459	Uma2 family endonuclease	55	47	48	52
KFAV_v1_2020	KFAV_v1_2020	Radical SAM protein (fragment)	55	47	48	52
KFAV_v1_3257	KFAV_v1_3257	conserved protein of unknown function	55	47	48	52
KFAV_v1_0880	KFAV_v1_0880	conserved membrane protein of unknown function	55	47	48	52
KFAV_v1_0335	KFAV_v1_0335	conserved membrane protein of unknown function	55	47	48	52
KFAV_v1_1290	KFAV_v1_1290	conserved protein of unknown function	55	47	48	52
uppP_1	KFAV_v1_0798	Undecaprenyl-diphosphatase	54	47	48	52
aldHT_2	KFAV_v1_0985	Aldehyde dehydrogenase, thermostable	54	47	48	52
deoB	KFAV_v1_2069	1,5-phosphopentomutase	54	47	48	51
gyaR	KFAV_v1_0810	Glyoxylate reductase	54	46	48	51
KFAV_v1_3426	KFAV_v1_3426	Efflux transporter, RND family, MFP subunit	54	46	47	51
gapA_1	KFAV_v1_0606	glyceraldehyde-3-phosphate dehydrogenase	54	46	47	51
glyQ	KFAV_v1_1263	glycyl-tRNA synthetase (alpha subunit)	54	46	47	51
tkt_2	KFAV_v1_2896	fragment of transketolase (part 1)	54	46	47	51
KFAV_v1_0692	KFAV_v1_0692	conserved protein of unknown function	54	46	47	51
KFAV_v1_2855	KFAV_v1_2855	conserved protein of unknown function	54	46	47	51
KFAV_v1_2975	KFAV_v1_2975	conserved protein of unknown function	54	46	47	51
KFAV_v1_0663	KFAV_v1_0663	conserved protein of unknown function	54	46	47	51
KFAV_v1_0648	KFAV_v1_0648	Resolvase domain protein	54	46	47	51
KFAV_v1_3415	KFAV_v1_3415	putative enzyme	54	46	47	51
chrA_1	KFAV_v1_0276	chromate transporter subunit C	54	46	47	51

mraZ	KFAV_v1_1540	inhibitor of RsmH and transcriptional regulator	54	46	47	50
KFAV_v1_2209	KFAV_v1_2209	Aminoglycoside phosphotransferase	53	46	47	50
KFAV_v1_1143	KFAV_v1_1143	conserved membrane protein of unknown function	53	46	47	50
KFAV_v1_0768	KFAV_v1_0768	Transport permease protein	53	46	47	50
artR	KFAV_v1_0984	high affinity arginine ABC transporter (ATP-binding protein)	53	46	47	50
KFAV_v1_1101	KFAV_v1_1101	conserved membrane protein of unknown function	53	46	47	50
KFAV_v1_3559	KFAV_v1_3559	conserved protein of unknown function	53	46	47	50
lspA	KFAV_v1_2060	signal peptidase II	53	46	46	50
ywaE	KFAV_v1_2985	Uncharacterized HTH-type transcriptional regulator YwaE	53	46	46	50
KFAV_v1_0385	KFAV_v1_0385	AAA family ATPase	53	46	46	50
KFAV_v1_0847	KFAV_v1_0847	Threonine-phosphate decarboxylase	53	45	46	49
KFAV_v1_1173	KFAV_v1_1173	putative Glycosyltransferase	53	45	46	49
KFAV_v1_3307	KFAV_v1_3307	protein of unknown function	53	45	46	49
KFAV_v1_3579	KFAV_v1_3579	conserved protein of unknown function	53	45	46	49
KFAV_v1_3331	KFAV_v1_3331	Amino acid transporter	53	45	46	49
birA	KFAV_v1_1718	biotin acetyl-CoA-carboxylase ligase and biotin regulon repressor (BirA-biotinoyl-5'-AMP)	53	45	46	49
ymdB	KFAV_v1_2390	conserved hypothetical protein	53	45	46	49
bshC	KFAV_v1_2400	putative cysteine ligase BshC	53	45	46	49
KFAV_v1_1725	KFAV_v1_1725	DNA polymerase domain-containing protein	53	45	46	49
KFAV_v1_0474	KFAV_v1_0474	MBL fold metallo-hydrolase	52	45	46	49
yoqW	KFAV_v1_3394	putative stress-associated peptidase; putative general secretion pathway protein; phage SPbeta	52	45	46	49
mhpF	KFAV_v1_2446	acetaldehyde dehydrogenase II	52	45	45	49
KFAV_v1_0666	KFAV_v1_0666	Carbohydrate ABC transporter permease	52	45	45	49
KFAV_v1_1777	KFAV_v1_1777	LLM class flavin-dependent oxidoreductase	52	45	45	48
scoA	KFAV_v1_0979	acetoacetyl CoA-transferase (subunit A)	52	45	45	48
KFAV_v1_1276	KFAV_v1_1276	conserved protein of unknown function	52	45	45	48
KFAV_v1_2167	KFAV_v1_2167	conserved protein of unknown function	52	45	45	48
KFAV_v1_2913	KFAV_v1_2913	conserved protein of unknown function	52	45	45	48
mdtG	KFAV_v1_2062	Multidrug resistance protein MdtG	52	45	45	48
KFAV_v1_1123	KFAV_v1_1123	conserved protein of unknown function	52	45	45	48
KFAV_v1_3354	KFAV_v1_3354	Nitrilase/cyanide hydratase and apolipoprotein N-acyltransferase	52	45	45	48
ribD	KFAV_v1_1584	fused diaminohydroxyphosphoribosylaminopyrimidine deaminase; 5-amino-6-(5-phosphoribosylamino) uracil reductase	52	45	45	48
KFAV_v1_0253	KFAV_v1_0253	Group II intron reverse transcriptase/maturase (fragment)	52	45	45	48
KFAV_v1_2115	KFAV_v1_2115	transposase (fragment)	52	45	45	48
nreC	KFAV_v1_1079	Oxygen regulatory protein NreC	52	45	45	48
KFAV_v1_2893	KFAV_v1_2893	conserved protein of unknown function	52	45	45	48
trmG	KFAV_v1_1516	persulfide ATP pyrophosphatase involved in tRNA modification	52	45	45	48
KFAV_v1_2350	KFAV_v1_2350	R2-like ligand binding oxidase	52	44	45	48
KFAV_v1_0894	KFAV_v1_0894	RNA 2',3'-cyclic phosphodiesterase	51	44	45	48
KFAV_v1_0061	KFAV_v1_0061	conserved protein of unknown function	51	44	45	48
KFAV_v1_1139	KFAV_v1_1139	conserved protein of unknown function	51	44	45	48
KFAV_v1_0396	KFAV_v1_0396	protein of unknown function	51	44	45	48
KFAV_v1_3376	KFAV_v1_3376	conserved protein of unknown function	51	44	45	48
KFAV_v1_2253	KFAV_v1_2253	Acetone carboxylase subunit alpha	51	44	44	48
KFAV_v1_0431	KFAV_v1_0431	conserved protein of unknown function	51	44	44	48
yjkB	KFAV_v1_2992	putative ABC transporter (ATP-binding protein)	51	44	44	48
yvcJ	KFAV_v1_0592	GTPase possibly involved in regulator sRNA degradation	51	44	44	48
rsmI	KFAV_v1_0053	16S rRNA 2'-O-ribose C1402 methyltransferase	51	44	44	47
KFAV_v1_2215	KFAV_v1_2215	protein of unknown function	51	44	44	47
aceB_1	KFAV_v1_2384	malate synthase A	51	44	44	47
KFAV_v1_2858	KFAV_v1_2858	conserved protein of unknown function	51	44	44	47
KFAV_v1_3095	KFAV_v1_3095	Sigma-54-dependent Fis family transcriptional regulator	51	44	44	47

KFAV_v1_3033	KFAV_v1_3033	conserved protein of unknown function	51	44	44	47
KFAV_v1_0300	KFAV_v1_0300	Arylformamidase	50	44	44	47
ldeJ_3	KFAV_v1_1686	isovaleryl-CoA dehydrogenase (leucine degradation)	50	44	44	47
ldeJ_2	KFAV_v1_1685	isovaleryl-CoA dehydrogenase (leucine degradation)	50	43	44	47
KFAV_v1_1599	KFAV_v1_1599	conserved exported protein of unknown function	50	43	44	47
KFAV_v1_0478	KFAV_v1_0478	Glycosyl transferase	50	43	44	47
KFAV_v1_0401	KFAV_v1_0401	protein of unknown function	50	43	44	47
KFAV_v1_0420	KFAV_v1_0420	protein of unknown function	50	43	43	47
allB	KFAV_v1_2044	Allantoinase	50	43	43	47
mutY	KFAV_v1_2856	Adenine DNA glycosylase	50	43	43	47
ldeE_2	KFAV_v1_1680	fragment of methylcrotonoyl-CoA carboxylase subunit (leucine degradation) (part 1)	50	43	43	47
KFAV_v1_0400	KFAV_v1_0400	transposase (fragment)	50	43	43	47
KFAV_v1_3363	KFAV_v1_3363	conserved protein of unknown function	50	43	43	46
KFAV_v1_0901	KFAV_v1_0901	conserved protein of unknown function	50	42	43	46
rimP	KFAV_v1_1870	ribosome maturation factor	50	42	43	46
uvrB	KFAV_v1_0564	excinuclease ABC (subunit B)	50	42	43	46
KFAV_v1_0547	KFAV_v1_0547	Long-chain-fatty-acid--CoA ligase	50	42	43	46
KFAV_v1_0484	KFAV_v1_0484	conserved membrane protein of unknown function	50	42	43	46
KFAV_v1_1732	KFAV_v1_1732	protein of unknown function	49	42	43	46
KFAV_v1_3479	KFAV_v1_3479	protein of unknown function	49	42	43	46
KFAV_v1_2624	KFAV_v1_2624	conserved protein of unknown function	49	42	43	46
cadA	KFAV_v1_0673	putative cadmium-transporting ATPase	49	42	43	46
sutR	KFAV_v1_0212	HTH-type transcriptional regulator SutR	49	42	43	46
KFAV_v1_0596	KFAV_v1_0596	conserved protein of unknown function	49	42	43	46
KFAV_v1_2507	KFAV_v1_2507	protein of unknown function	49	42	43	46
KFAV_v1_3184	KFAV_v1_3184	Toxin-antitoxin system HicB family antitoxin	49	42	43	46
KFAV_v1_0961	KFAV_v1_0961	ABC transporter permease	49	42	42	46
KFAV_v1_0908	KFAV_v1_0908	conserved protein of unknown function	49	42	42	45
KFAV_v1_0959	KFAV_v1_0959	ABC transporter substrate-binding protein	49	42	42	45
KFAV_v1_0428	KFAV_v1_0428	Antitoxin	49	42	42	45
rsmH	KFAV_v1_1541	16S rRNA m4C1402 methyltransferase	49	42	42	45
grpE	KFAV_v1_2144	nucleotide exchange factor for DnaK activity	49	42	42	45
yrkI	KFAV_v1_2705	putative sulfur-carrier protein	49	42	42	45
KFAV_v1_2795	KFAV_v1_2795	FeoA family protein	49	42	42	45
KFAV_v1_2914	KFAV_v1_2914	conserved protein of unknown function	49	42	42	45
KFAV_v1_3310	KFAV_v1_3310	MFS transporter	49	42	42	45
KFAV_v1_1350	KFAV_v1_1350	DedA family protein	49	41	42	45
KFAV_v1_2007	KFAV_v1_2007	conserved protein of unknown function	49	41	42	45
yjgC	KFAV_v1_2285	putative molybdoenzyme; putative formate dehydrogenase	49	41	42	45
KFAV_v1_0978	KFAV_v1_0978	Transcriptional regulator, IclR family	48	41	42	45
KFAV_v1_1815	KFAV_v1_1815	conserved exported protein of unknown function	48	41	42	45
KFAV_v1_0210	KFAV_v1_0210	Branched-chain amino acid ABC transporter permease	48	41	42	45
KFAV_v1_0958	KFAV_v1_0958	protein of unknown function	48	41	42	45
KFAV_v1_1504	KFAV_v1_1504	conserved protein of unknown function	48	41	42	45
mraY	KFAV_v1_1546	phospho-N-acetylmuramoyl-pentapeptide undecaprenyl phosphate (C55P) transferase	48	41	42	45
ribE	KFAV_v1_1585	riboflavin synthase (alpha subunit)	48	41	42	45
KFAV_v1_2252	KFAV_v1_2252	Hydantoinase/oxoprolinase	48	41	41	45
mnmE	KFAV_v1_3611	tRNA modification GTPase and tRNA-U34 5-formylation enzyme	48	41	41	45
KFAV_v1_0756	KFAV_v1_0756	Glycoside hydrolase family 2 sugar binding protein	48	41	41	45
KFAV_v1_0925	KFAV_v1_0925	Thiamine-phosphate synthase (modular protein)	48	41	41	45
KFAV_v1_1425	KFAV_v1_1425	protein of unknown function	48	41	41	45
lutA	KFAV_v1_2611	iron-sulfur oxidase subunit used in L-lactate utilization	48	41	41	45

KFAV_v1_1305	KFAV_v1_1305	transposase (fragment)	48	41	41	45
KFAV_v1_1524	KFAV_v1_1524	protein of unknown function	48	41	41	44
KFAV_v1_2303	KFAV_v1_2303	conserved exported protein of unknown function	47	41	41	44
hisZ	KFAV_v1_0570	histidyl-tRNA synthetase-like subunit of ATP phophoribosyltransferase	47	41	41	44
puuE	KFAV_v1_0981	GABA aminotransferase, PLP-dependent	47	41	41	44
KFAV_v1_2178	KFAV_v1_2178	Branched-chain amino acid ABC transporter permease	47	41	41	44
KFAV_v1_3271	KFAV_v1_3271	conserved protein of unknown function	47	40	41	44
KFAV_v1_2998	KFAV_v1_2998	Amidase	47	40	41	44
KFAV_v1_3202	KFAV_v1_3202	conserved protein of unknown function	47	40	41	44
KFAV_v1_1002	KFAV_v1_1002	conserved membrane protein of unknown function	47	40	41	44
KFAV_v1_1361	KFAV_v1_1361	conserved protein of unknown function	47	40	41	44
KFAV_v1_0475	KFAV_v1_0475	MFS transporter	47	40	41	44
KFAV_v1_0587	KFAV_v1_0587	conserved protein of unknown function	47	40	41	44
KFAV_v1_3428	KFAV_v1_3428	protein of unknown function	47	40	40	43
KFAV_v1_0842	KFAV_v1_0842	conserved protein of unknown function	47	40	40	43
KFAV_v1_2107	KFAV_v1_2107	CoA transferase	47	40	40	43
lipL	KFAV_v1_1249	Octanoyl-[GcvH]:protein N-octanoyltransferase	47	40	40	43
KFAV_v1_2114	KFAV_v1_2114	transposase (fragment)	47	40	40	43
KFAV_v1_3201	KFAV_v1_3201	Restriction endonuclease subunit S	47	40	40	43
KFAV_v1_2359	KFAV_v1_2359	Peptidoglycan-binding protein	46	40	40	43
KFAV_v1_2365	KFAV_v1_2365	conserved protein of unknown function	46	40	40	43
KFAV_v1_2467	KFAV_v1_2467	IclR family transcriptional regulator	46	40	40	43
KFAV_v1_2646	KFAV_v1_2646	conserved protein of unknown function	46	40	40	43
KFAV_v1_2078	KFAV_v1_2078	conserved protein of unknown function	46	40	40	43
livF_3	KFAV_v1_1835	leucine/isoleucine/valine transporter subunit ; ATP-binding component of ABC superfamily	46	40	40	43
KFAV_v1_2669	KFAV_v1_2669	conserved protein of unknown function	46	40	40	43
KFAV_v1_1318	KFAV_v1_1318	Serine/threonine dehydratase	46	40	40	43
mmgB_1	KFAV_v1_2125	3-hydroxybutyryl-CoA dehydrogenase	46	39	40	43
KFAV_v1_0761	KFAV_v1_0761	protein of unknown function	45	39	40	43
KFAV_v1_0876	KFAV_v1_0876	conserved protein of unknown function	45	39	40	43
raeA	KFAV_v1_0145	ribosome-dependent mRNA endonuclease	45	39	39	43
KFAV_v1_1760	KFAV_v1_1760	NifZ family protein	45	39	39	42
ykcC	KFAV_v1_0322	putative glycosyltransferase	45	39	39	42
KFAV_v1_2469	KFAV_v1_2469	Ferredoxin	45	39	39	42
KFAV_v1_2799	KFAV_v1_2799	conserved membrane protein of unknown function	45	39	39	42
KFAV_v1_0986	KFAV_v1_0986	Glycine/D-amino acid oxidase	45	39	39	42
mmgC_1	KFAV_v1_2124	propionyl-CoA dehydrogenase subunit	45	39	39	42
spoIIM	KFAV_v1_2075	Stage II sporulation protein M	45	39	39	42
KFAV_v1_0962	KFAV_v1_0962	ABC transporter ATP-binding protein	45	39	39	42
KFAV_v1_1106	KFAV_v1_1106	Radical SAM protein	45	39	39	42
actP_2	KFAV_v1_1109	Cation acetate symporter	45	39	39	42
KFAV_v1_2854	KFAV_v1_2854	conserved protein of unknown function	45	38	39	42
KFAV_v1_2113	KFAV_v1_2113	conserved protein of unknown function	45	38	39	42
uvrA	KFAV_v1_0565	excinuclease ABC (subunit A)	44	38	39	42
KFAV_v1_0372	KFAV_v1_0372	protein of unknown function	44	38	39	42
KFAV_v1_0537	KFAV_v1_0537	conserved membrane protein of unknown function	44	38	39	42
KFAV_v1_2181	KFAV_v1_2181	conserved protein of unknown function	44	38	39	42
KFAV_v1_2879	KFAV_v1_2879	conserved protein of unknown function	44	38	39	42
KFAV_v1_3474	KFAV_v1_3474	conserved protein of unknown function	44	38	39	42
KFAV_v1_2696	KFAV_v1_2696	Biotin/lipoate A/B protein ligase	44	38	39	42
rsmF	KFAV_v1_0885	Ribosomal RNA small subunit methyltransferase F	44	38	39	41
KFAV_v1_0507	KFAV_v1_0507	ABC transporter substrate-binding protein	44	38	38	41

KFAV_v1_1731	KFAV_v1_1731	conserved protein of unknown function	44	37	38	41
purD	KFAV_v1_0320	phosphoribosylglycinamide synthetase	43	37	38	41
KFAV_v1_1292	KFAV_v1_1292	protein of unknown function	43	37	38	41
KFAV_v1_1982	KFAV_v1_1982	Endopeptidase La	43	37	38	41
KFAV_v1_0501	KFAV_v1_0501	conserved protein of unknown function	43	37	38	41
KFAV_v1_0561	KFAV_v1_0561	protein of unknown function	43	37	38	41
KFAV_v1_2244	KFAV_v1_2244	conserved protein of unknown function	43	37	38	41
KFAV_v1_2700	KFAV_v1_2700	Ligase	43	37	38	41
rlmB	KFAV_v1_0144	23S rRNA (Gm2251)-methyltransferase	43	37	38	41
ltrA_6	KFAV_v1_2502	Group II intron reverse transcriptase/maturase	43	37	38	41
KFAV_v1_0258	KFAV_v1_0258	conserved protein of unknown function	43	37	38	41
KFAV_v1_0935	KFAV_v1_0935	protein of unknown function	43	37	38	41
KFAV_v1_3044	KFAV_v1_3044	protein of unknown function	43	37	38	41
KFAV_v1_3490	KFAV_v1_3490	conserved protein of unknown function	43	37	38	41
tsaC_2	KFAV_v1_3532	tRNA(NNU) t(6)A37 threonylcarbamoyladenosine modification; threonine-dependent ADP-forming ATPase	43	37	38	41
dnaA	KFAV_v1_0002	chromosomal replication initiator informational ATPase	43	37	38	40
KFAV_v1_1140	KFAV_v1_1140	conserved protein of unknown function	43	37	37	40
ppsR	KFAV_v1_1266	bifunctional ADP-dependent kinase-Pi-dependent pyrophosphorylase / positive regulator of gluconeogenesis	43	37	37	40
KFAV_v1_1648	KFAV_v1_1648	conserved protein of unknown function	43	37	37	40
KFAV_v1_3026	KFAV_v1_3026	protein of unknown function	42	37	37	40
KFAV_v1_3305	KFAV_v1_3305	Transcriptional regulator, GntR family	42	37	37	40
KFAV_v1_1313	KFAV_v1_1313	conserved protein of unknown function	42	36	37	40
glcF_1	KFAV_v1_1360	glycolate oxidase (iron-sulfur subunit)	42	36	37	40
gudB	KFAV_v1_1635	cryptic glutamate dehydrogenase (active after removal of a 9 bp insert)	42	36	37	40
cdd	KFAV_v1_2012	Cytidine deaminase	42	36	37	40
KFAV_v1_2127	KFAV_v1_2127	conserved protein of unknown function	42	36	37	40
suhB	KFAV_v1_2232	Inositol-1-monophosphatase	42	36	37	40
ssuB	KFAV_v1_0448	aliphatic sulfonate ABC transporter (ATP-binding protein)	42	36	37	40
KFAV_v1_0476	KFAV_v1_0476	Ubiquinone biosynthesis protein UbiE	42	36	37	40
KFAV_v1_0989	KFAV_v1_0989	Sarcosine oxidase subunit alpha	41	36	37	40
KFAV_v1_0851	KFAV_v1_0851	conserved membrane protein of unknown function	41	36	37	40
KFAV_v1_3146	KFAV_v1_3146	conserved protein of unknown function	41	36	37	40
KFAV_v1_3203	KFAV_v1_3203	Restriction modification system DNA specificity domain-containing protein	41	36	37	40
KFAV_v1_0402	KFAV_v1_0402	Type II secretory pathway, component ExeA (Predicted ATPase) (fragment)	41	36	37	40
KFAV_v1_1358	KFAV_v1_1358	conserved protein of unknown function	41	36	36	40
KFAV_v1_0217	KFAV_v1_0217	Transcriptional regulator	41	36	36	40
KFAV_v1_0321	KFAV_v1_0321	conserved membrane protein of unknown function	41	36	36	40
pepA_2	KFAV_v1_1614	fragment of cytosol aminopeptidase (part 2)	41	36	36	40
KFAV_v1_1892	KFAV_v1_1892	Protein-glutamate methylesterase	41	36	36	40
KFAV_v1_2907	KFAV_v1_2907	protein of unknown function	41	36	36	39
KFAV_v1_1363	KFAV_v1_1363	Pyridinium-3,5-bisthiocarboxylic acid mononucleotide nickel insertion protein (fragment)	41	36	36	39
qoxA	KFAV_v1_2238	Quinol oxidase subunit 2	41	36	36	39
todF	KFAV_v1_2453	2-hydroxy-6-oxo-2,4-heptadienoate hydrolase	41	36	36	39
xamoE	KFAV_v1_2465	Alkene monooxygenase system, oxygenase component subunit beta	41	35	36	39
KFAV_v1_3205	KFAV_v1_3205	protein of unknown function	40	35	36	39
KFAV_v1_0444	KFAV_v1_0444	Amidase, hydantoinase/carbamoylase family	40	35	36	39
KFAV_v1_2396	KFAV_v1_2396	protein of unknown function	40	35	36	39
adh_2	KFAV_v1_0757	Alcohol dehydrogenase	40	35	36	39
KFAV_v1_3060	KFAV_v1_3060	putative enzyme	40	35	36	39
acxC_1	KFAV_v1_2251	Acetone carboxylase gamma subunit	40	35	36	39
galE_2	KFAV_v1_0377	UDP-glucose 4-epimerase	40	35	36	39
KFAV_v1_0015	KFAV_v1_0015	conserved protein of unknown function	40	35	36	39

KFAV_v1_1286	KFAV_v1_1286	transposase (fragment)	40	35	36	39
KFAV_v1_1673	KFAV_v1_1673	conserved protein of unknown function	40	35	35	39
KFAV_v1_2930	KFAV_v1_2930	conserved protein of unknown function	40	35	35	39
KFAV_v1_3248	KFAV_v1_3248	protein of unknown function	40	35	35	39
KFAV_v1_0443	KFAV_v1_0443	Transcriptional regulator, RpiR family	40	35	35	39
pitB	KFAV_v1_2559	putative low-affinity inorganic phosphate transporter	40	35	35	39
trmF	KFAV_v1_3610	tRNA uridine 5-carboxymethylaminomethyl modification enzyme	40	35	35	39
cobI	KFAV_v1_0832	Precorrin-2 C(20)-methyltransferase	40	35	35	39
KFAV_v1_0597	KFAV_v1_0597	conserved protein of unknown function	40	35	35	38
mntP	KFAV_v1_3531	manganese efflux pump	40	35	35	38
KFAV_v1_1885	KFAV_v1_1885	RNA polymerase sigma factor	40	35	35	38
iscS	KFAV_v1_1939	Cysteine desulfurase IscS 1	40	35	35	38
KFAV_v1_0672	KFAV_v1_0672	ArsR family transcriptional regulator	40	34	35	38
ureC	KFAV_v1_0550	urease (alpha subunit)	39	34	35	38
KFAV_v1_1467	KFAV_v1_1467	protein of unknown function	39	34	35	38
gmuE	KFAV_v1_2019	putative fructokinase	39	34	35	38
KFAV_v1_3169	KFAV_v1_3169	conserved protein of unknown function	39	34	35	38
KFAV_v1_1837	KFAV_v1_1837	Branched-chain amino acid ABC transporter permease	39	34	35	38
KFAV_v1_0340	KFAV_v1_0340	protein of unknown function	39	34	35	38
KFAV_v1_3103	KFAV_v1_3103	MFS transporter	39	34	35	38
spoIIAB	KFAV_v1_1573	anti-sigma factor (antagonist of sigma(F)) and serine kinase	39	34	35	38
prtG	KFAV_v1_0062	sporulation-specific protease	39	34	34	38
KFAV_v1_0749	KFAV_v1_0749	protein of unknown function	39	34	34	38
KFAV_v1_3170	KFAV_v1_3170	conserved protein of unknown function	39	34	34	37
fabG_6	KFAV_v1_2654	3-oxoacyl-[acyl-carrier-protein] reductase FabG	38	34	34	37
KFAV_v1_0599	KFAV_v1_0599	conserved protein of unknown function	38	34	34	37
lytS	KFAV_v1_1110	Sensor protein LytS	38	34	34	37
KFAV_v1_1757	KFAV_v1_1757	Protein HesA, heterocyst (fragment)	38	34	34	37
KFAV_v1_3270	KFAV_v1_3270	Phosphatidylglycerol lysyltransferase (fragment)	38	34	34	37
larB	KFAV_v1_1362	Pyridinium-3,5-biscarboxylic acid mononucleotide synthase	38	34	34	37
KFAV_v1_1330	KFAV_v1_1330	Nitrogen regulatory protein P-II (modular protein)	38	34	34	37
radA	KFAV_v1_0135	DNA repair protein; 6-O-methylguanine-DNA methyltransferase	38	34	34	37
KFAV_v1_0405	KFAV_v1_0405	transposase	38	34	33	37
tkt_1	KFAV_v1_2895	fragment of transketolase (part 2)	38	34	33	37
KFAV_v1_3004	KFAV_v1_3004	protein of unknown function	38	34	33	37
KFAV_v1_3043	KFAV_v1_3043	protein of unknown function	38	33	33	37
KFAV_v1_3262	KFAV_v1_3262	N-acetylglucosaminyldiphosphoundecaprenol N-acetyl-beta-D-mannosaminyltransferase	38	33	33	37
KFAV_v1_3242	KFAV_v1_3242	protein of unknown function	38	33	33	37
gabP	KFAV_v1_2996	GABA permease	37	33	33	36
KFAV_v1_3460	KFAV_v1_3460	protein of unknown function	37	33	33	36
KFAV_v1_3462	KFAV_v1_3462	PIN domain nuclease (fragment)	37	33	33	36
ppx	KFAV_v1_2484	Exopolyphosphatase	37	33	33	36
KFAV_v1_0775	KFAV_v1_0775	Acyl-CoA dehydrogenase (modular protein)	37	33	33	36
livG_2	KFAV_v1_1836	leucine/isoleucine/valine transporter subunit ; ATP-binding component of ABC superfamily	37	33	33	36
KFAV_v1_1404	KFAV_v1_1404	SMC domain-containing protein	37	33	33	36
miaA	KFAV_v1_1786	tRNA isopentenylpyrophosphate transferase	37	33	33	36
flgC	KFAV_v1_1917	flagellar component of cell-proximal portion of basal-body rod	37	33	33	36
KFAV_v1_2154	KFAV_v1_2154	Alpha/beta hydrolase	37	33	33	36
KFAV_v1_2649	KFAV_v1_2649	conserved protein of unknown function	37	33	33	36
KFAV_v1_1626	KFAV_v1_1626	conserved protein of unknown function	37	33	33	36
KFAV_v1_2710	KFAV_v1_2710	Electron transfer flavoprotein subunit alpha/FixB family protein	37	33	33	36
KFAV_v1_1687	KFAV_v1_1687	conserved protein of unknown function	37	33	33	36

pstBB_2	KFAV_v1_3116	phosphate ABC transporter (ATP-binding protein)	37	33	33	36
KFAV_v1_0736	KFAV_v1_0736	conserved protein of unknown function	37	33	33	36
KFAV_v1_1776	KFAV_v1_1776	conserved protein of unknown function	37	33	33	36
KFAV_v1_0365	KFAV_v1_0365	IclR family transcriptional regulator	36	33	33	36
gpuA	KFAV_v1_1824	Guanidinopropionase	36	32	32	36
KFAV_v1_0776	KFAV_v1_0776	protein of unknown function	36	32	32	36
KFAV_v1_0840	KFAV_v1_0840	Cobyric acid synthase (fragment)	36	32	32	36
paaI	KFAV_v1_2135	Phenylacetate-CoA oxygenase subunit PaaI	36	32	32	36
KFAV_v1_0504	KFAV_v1_0504	transposase	36	32	32	36
pyrD_1	KFAV_v1_2047	dihydroorotate dehydrogenase (catalytic subunit)	36	32	32	36
KFAV_v1_2978	KFAV_v1_2978	ABC transporter related protein	36	32	32	36
KFAV_v1_0725	KFAV_v1_0725	conserved protein of unknown function	36	32	32	35
KFAV_v1_2406	KFAV_v1_2406	conserved membrane protein of unknown function	36	32	32	35
tcaB	KFAV_v1_2006	P-type calcium transport ATPase (sporulation)	36	32	32	35
KFAV_v1_1571	KFAV_v1_1571	Nitrate ABC transporter ATP-binding protein	36	32	32	35
KFAV_v1_2175	KFAV_v1_2175	Hydroxyacid dehydrogenase	36	32	32	35
glcD_1	KFAV_v1_1359	glycolate oxidase subunit	36	32	32	35
dapB	KFAV_v1_1716	(4S)-4-hydroxy-2,3,4,5-tetrahydro-(2S)-dipicolinic acid (HTPA) dehydratase reductase	36	32	32	35
KFAV_v1_1391	KFAV_v1_1391	FMN-binding glutamate synthase family protein	36	32	32	35
KFAV_v1_2231	KFAV_v1_2231	Patatin	36	31	32	35
fdhD	KFAV_v1_2288	Sulfur carrier protein FdhD	36	31	32	35
KFAV_v1_0042	KFAV_v1_0042	Pseudouridine synthase	36	31	32	35
KFAV_v1_2949	KFAV_v1_2949	protein of unknown function	36	31	32	35
KFAV_v1_0879	KFAV_v1_0879	DNA-binding response regulator	36	31	32	35
darA	KFAV_v1_0048	signal transduction receptor, cyclic di-AMP binding	35	31	32	35
KFAV_v1_0738	KFAV_v1_0738	Rhodanese-like domain-containing protein	35	31	32	35
KFAV_v1_1646	KFAV_v1_1646	conserved membrane protein of unknown function	35	31	32	35
KFAV_v1_2239	KFAV_v1_2239	protein of unknown function	35	31	32	35
KFAV_v1_2364	KFAV_v1_2364	Sporulation protein	35	31	32	35
KFAV_v1_2881	KFAV_v1_2881	conserved protein of unknown function	35	31	32	35
KFAV_v1_1087	KFAV_v1_1087	protein of unknown function	35	31	32	35
ctaE_2	KFAV_v1_2236	cytochrome caa3 oxidase (subunit III)	35	31	32	35
KFAV_v1_1543	KFAV_v1_1543	conserved exported protein of unknown function	35	31	32	34
yhfA_1	KFAV_v1_0264	fragment of putative transporter (part 2)	35	31	31	34
KFAV_v1_0713	KFAV_v1_0713	conserved protein of unknown function	35	31	31	34
KFAV_v1_3113	KFAV_v1_3113	Alpha/beta hydrolase	35	31	31	34
KFAV_v1_0910	KFAV_v1_0910	conserved protein of unknown function	35	31	31	34
KFAV_v1_2094	KFAV_v1_2094	MFS transporter	35	31	31	34
KFAV_v1_0219	KFAV_v1_0219	conserved protein of unknown function	35	31	31	34
ydeD_2	KFAV_v1_2995	fragment of putative permease (part 1)	35	31	31	34
KFAV_v1_3425	KFAV_v1_3425	Drug resistance transporter, EmrB/QacA subfamily	35	31	31	34
KFAV_v1_0384	KFAV_v1_0384	transposase (fragment)	34	30	31	34
KFAV_v1_3537	KFAV_v1_3537	conserved exported protein of unknown function	34	30	31	34
hisA	KFAV_v1_0575	phosphoribosylformimino-5-aminoimidazole carboxamide ribotide isomerase	34	30	31	34
KFAV_v1_1971	KFAV_v1_1971	protein of unknown function	34	30	31	34
KFAV_v1_2136	KFAV_v1_2136	Phenylacetic acid degradation B	34	30	31	34
KFAV_v1_2210	KFAV_v1_2210	Acyl-CoA dehydrogenase domain protein	34	30	31	34
ygjG	KFAV_v1_1825	putrescine:2-oxoglutaric acid aminotransferase, PLP-dependent	34	30	31	34
KFAV_v1_3449	KFAV_v1_3449	Antitoxin	34	30	31	34
cobD	KFAV_v1_0843	Cobalamin biosynthesis protein CobD	34	30	31	34
KFAV_v1_1818	KFAV_v1_1818	conserved membrane protein of unknown function	34	30	30	34
KFAV_v1_1677	KFAV_v1_1677	conserved protein of unknown function	34	30	30	34

yvlD	KFAV_v1_0566	Uncharacterized membrane protein YvlD	34	30	30	33
KFAV_v1_0691	KFAV_v1_0691	conserved membrane protein of unknown function	34	30	30	33
cbiG	KFAV_v1_0834	Cobalt-precorrin-5A hydrolase	34	30	30	33
KFAV_v1_1014	KFAV_v1_1014	protein of unknown function	34	30	30	33
KFAV_v1_2298	KFAV_v1_2298	protein of unknown function	34	30	30	33
KFAV_v1_2565	KFAV_v1_2565	protein of unknown function	34	30	30	33
KFAV_v1_2910	KFAV_v1_2910	protein of unknown function	34	29	30	33
KFAV_v1_0296	KFAV_v1_0296	PAS modulated sigma54 specific transcriptional regulator, Fis family	34	29	30	33
pstC_2	KFAV_v1_3118	phosphate transporter subunit ; membrane component of ABC superfamily	34	29	30	33
phnPP	KFAV_v1_3364	Phosphoribosyl 1,2-cyclic phosphate 1,2-diphosphodiesterase	34	29	30	33
KFAV_v1_1255	KFAV_v1_1255	conserved protein of unknown function	34	29	30	33
fadA_2	KFAV_v1_2126	acetyl-CoA C-acyltransferase	34	29	30	33
KFAV_v1_0453	KFAV_v1_0453	Uma2 family endonuclease	34	29	30	33
rnz	KFAV_v1_0562	Ribonuclease Z	34	29	30	33
KFAV_v1_0441	KFAV_v1_0441	conserved protein of unknown function	34	29	30	33
KFAV_v1_2324	KFAV_v1_2324	TetR family transcriptional regulator	33	29	30	33
KFAV_v1_0022	KFAV_v1_0022	conserved membrane protein of unknown function	33	29	30	33
KFAV_v1_1730	KFAV_v1_1730	conserved protein of unknown function	33	29	30	32
KFAV_v1_2832	KFAV_v1_2832	protein of unknown function	33	29	30	32
KFAV_v1_3196	KFAV_v1_3196	protein of unknown function	33	29	30	32
KFAV_v1_3212	KFAV_v1_3212	transposase (fragment)	33	29	30	32
KFAV_v1_3470	KFAV_v1_3470	Constitutive lysine decarboxylase (fragment)	33	29	29	32
prmA	KFAV_v1_2140	Ribosomal protein L11 methyltransferase	33	29	29	32
KFAV_v1_0678	KFAV_v1_0678	ABC transporter ATP-binding protein	33	29	29	32
KFAV_v1_2111	KFAV_v1_2111	conserved protein of unknown function	33	29	29	32
spoIIAA	KFAV_v1_1572	anti-anti-sigma factor (antagonist of SpoIIAB)	33	29	29	32
KFAV_v1_1490	KFAV_v1_1490	protein of unknown function	33	29	29	32
KFAV_v1_0373	KFAV_v1_0373	conserved protein of unknown function	33	28	29	32
KFAV_v1_2916	KFAV_v1_2916	conserved protein of unknown function	33	28	29	32
wtpC	KFAV_v1_1740	Molybdate/tungstate import ATP-binding protein WtpC	33	28	29	32
sbp	KFAV_v1_1555	putative integral inner membrane protein (small basic protein)	33	28	29	32
KFAV_v1_2794	KFAV_v1_2794	Transcriptional regulator, Crp/Fnr family	33	28	29	32
modB	KFAV_v1_1739	Molybdate ABC transporter permease subunit	33	28	29	32
fpaP	KFAV_v1_2031	Proline iminopeptidase	32	28	29	32
hyuC_1	KFAV_v1_3163	N-carbamoyl-L-amino-acid hydrolase	32	28	29	31
ureF	KFAV_v1_0551	Urease accessory protein UreF	32	28	29	31
KFAV_v1_3529	KFAV_v1_3529	ZIP family metal transporter (fragment)	32	28	29	31
qoxB	KFAV_v1_2237	cytochrome aa3-600 quinol oxidase (subunit I)	32	28	29	31
KFAV_v1_0762	KFAV_v1_0762	transposase	32	28	29	31
KFAV_v1_1724	KFAV_v1_1724	DNA ligase	32	28	29	31
KFAV_v1_2772	KFAV_v1_2772	protein of unknown function	32	28	29	31
ahpA_1	KFAV_v1_0330	biofilm-specific peroxidase; 2-cys peroxiredoxin	32	28	28	31
KFAV_v1_2595	KFAV_v1_2595	Glycerol uptake facilitator protein	32	28	28	31
KFAV_v1_3563	KFAV_v1_3563	protein of unknown function	32	28	28	31
KFAV_v1_3600	KFAV_v1_3600	conserved membrane protein of unknown function	32	28	28	31
KFAV_v1_1005	KFAV_v1_1005	putative HTH-type transcriptional repressor YvoA	32	28	28	31
disA	KFAV_v1_0136	diadenylate cyclase; DNA integrity scanning protein; cell cycle checkpoint DNA scanning protein	32	28	28	31
KFAV_v1_1678	KFAV_v1_1678	conserved protein of unknown function	32	28	28	31
ydfM	KFAV_v1_0222	Uncharacterized transporter YdfM	32	28	28	31
KFAV_v1_2168	KFAV_v1_2168	Ribosome biogenesis GTPase YqeH	32	28	28	31
KFAV_v1_2030	KFAV_v1_2030	Acetamidase	32	28	28	31
xylE_1	KFAV_v1_2271	Metapyrocatechase	32	28	28	31

KFAV_v1_0337	KFAV_v1_0337	DNA polymerase IV	32	27	28	31
KFAV_v1_0234	KFAV_v1_0234	Thiamine-monophosphate kinase (fragment)	32	27	28	31
ispE	KFAV_v1_0068	4-(cytidine 5'-diphospho)-2-C-methyl-D-erythritol kinase	31	27	28	31
KFAV_v1_0251	KFAV_v1_0251	protein of unknown function	31	27	28	31
KFAV_v1_0703	KFAV_v1_0703	DNA-binding response regulator MtrA (fragment)	31	27	28	31
KFAV_v1_3049	KFAV_v1_3049	conserved protein of unknown function	31	27	28	31
KFAV_v1_2309	KFAV_v1_2309	conserved protein of unknown function	31	27	28	31
murB	KFAV_v1_1550	UDP-N-acetylenolpyruvoylglucosamine reductase	31	27	28	31
ALDH_2	KFAV_v1_2128	Aldehyde dehydrogenase	31	27	28	31
tsaE	KFAV_v1_0236	tRNA(NNU) t(6)A37 threonylcarbamoyladenosine modification; ADP binding protein	31	27	28	31
KFAV_v1_0259	KFAV_v1_0259	Uncharacterized oxidoreductase TM_0019	31	27	28	30
KFAV_v1_1384	KFAV_v1_1384	protein of unknown function	31	27	27	30
KFAV_v1_1608	KFAV_v1_1608	conserved protein of unknown function	31	27	27	30
KFAV_v1_2015	KFAV_v1_2015	protein of unknown function	31	27	27	30
KFAV_v1_0753	KFAV_v1_0753	conserved protein of unknown function	31	27	27	30
pdaB	KFAV_v1_0199	Polysaccharide deacetylase family sporulation protein PdaB	31	27	27	30
tcdA	KFAV_v1_0891	tRNA threonylcarbamoyladenosine dehydratase (t(6)A37 dehydratase)	31	27	27	30
KFAV_v1_0999	KFAV_v1_0999	RNA polymerase subunit sigma-24	31	27	27	30
KFAV_v1_2906	KFAV_v1_2906	TlpA family protein disulfide reductase	31	27	27	30
KFAV_v1_3197	KFAV_v1_3197	protein of unknown function	31	26	27	30
KFAV_v1_3275	KFAV_v1_3275	conserved protein of unknown function	31	26	27	30
recD_2	KFAV_v1_3255	ATP-dependent RecD-like DNA helicase	30	26	27	30
KFAV_v1_2607	KFAV_v1_2607	conserved exported protein of unknown function	30	26	27	30
splB	KFAV_v1_0419	spore photoproduct (thymine dimer) lyase	30	26	27	30
KFAV_v1_2722	KFAV_v1_2722	conserved membrane protein of unknown function	30	26	27	30
KFAV_v1_0093	KFAV_v1_0093	transposase (fragment)	30	26	27	30
KFAV_v1_2177	KFAV_v1_2177	ABC transporter ATP-binding protein	30	26	27	30
glnT	KFAV_v1_3330	Glutamine synthetase	30	26	27	30
KFAV_v1_2321	KFAV_v1_2321	AMP-dependent synthetase	30	26	27	30
KFAV_v1_3243	KFAV_v1_3243	Uma2 family endonuclease	30	26	27	30
KFAV_v1_1088	KFAV_v1_1088	Integrase core domain-containing protein (fragment)	30	26	27	30
uppP_2	KFAV_v1_1713	Undecaprenyl-diphosphatase	30	26	27	30
KFAV_v1_2343	KFAV_v1_2343	conserved protein of unknown function	30	26	27	29
KFAV_v1_2554	KFAV_v1_2554	conserved protein of unknown function	30	26	27	29
KFAV_v1_2883	KFAV_v1_2883	conserved membrane protein of unknown function	30	26	27	29
KFAV_v1_0351	KFAV_v1_0351	conserved protein of unknown function	30	26	27	29
KFAV_v1_2180	KFAV_v1_2180	conserved exported protein of unknown function	30	26	27	29
KFAV_v1_1464	KFAV_v1_1464	conserved protein of unknown function	30	26	26	29
KFAV_v1_2029	KFAV_v1_2029	Amino acid permease	30	26	26	29
efpG	KFAV_v1_3517	involved in 5-aminopentanol modification of EF-P	29	26	26	29
KFAV_v1_0449	KFAV_v1_0449	D-isomer specific 2-hydroxyacid dehydrogenase NAD-binding protein	29	26	26	29
KFAV_v1_1773	KFAV_v1_1773	Metal-dependent hydrolase	29	26	26	29
pgcA	KFAV_v1_0460	phosphoglucomutase	29	25	26	29
KFAV_v1_0581	KFAV_v1_0581	protein of unknown function	29	25	26	28
ytrA	KFAV_v1_0802	transcriptional regulator (GntR family, cell wall antibiotics)	29	25	26	28
KFAV_v1_1583	KFAV_v1_1583	protein of unknown function	29	25	26	28
spoIIIE	KFAV_v1_1819	spore DNA directional translocase (motor ATPase)	29	25	26	28
KFAV_v1_2928	KFAV_v1_2928	protein of unknown function	29	25	26	28
KFAV_v1_2939	KFAV_v1_2939	protein of unknown function	29	25	26	28
KFAV_v1_3226	KFAV_v1_3226	protein of unknown function	29	25	26	28
KFAV_v1_2048	KFAV_v1_2048	Dihydroorotate dehydrogenase electron transfer subunit	29	25	26	28
hpaD	KFAV_v1_1844	3,4-dihydroxyphenylacetate 2,3-dioxygenase	29	25	26	28

ureA	KFAV_v1_0549	Urease subunit gamma	29	25	26	28
menB	KFAV_v1_2656	dihydroxynapthoic acid synthetase	29	25	26	28
KFAV_v1_0920	KFAV_v1_0920	transposase (fragment)	29	25	26	28
ydcM_1	KFAV_v1_1306	putative transposase	29	25	26	28
KFAV_v1_0774	KFAV_v1_0774	APC family permease	29	25	26	28
KFAV_v1_0095	KFAV_v1_0095	conserved protein of unknown function	29	25	26	28
KFAV_v1_1035	KFAV_v1_1035	conserved protein of unknown function	29	25	26	28
KFAV_v1_1048	KFAV_v1_1048	protein of unknown function	29	25	26	28
thiS	KFAV_v1_1604	sulfur carrier for synthesis of hydroxyethylthiazole phosphate	29	24	26	28
KFAV_v1_2261	KFAV_v1_2261	conserved protein of unknown function	29	24	26	28
KFAV_v1_2615	KFAV_v1_2615	protein of unknown function	29	24	26	28
KFAV_v1_2311	KFAV_v1_2311	conserved membrane protein of unknown function	29	24	26	27
KFAV_v1_0530	KFAV_v1_0530	transposase (fragment)	29	24	26	27
KFAV_v1_0872	KFAV_v1_0872	conserved membrane protein of unknown function	29	24	26	27
KFAV_v1_2348	KFAV_v1_2348	conserved membrane protein of unknown function	29	24	26	27
pdeB	KFAV_v1_1806	2'3' and 3'5' cyclic nucleotide monophosphates phosphodiesterase involved in biofilm formation	28	24	26	27
KFAV_v1_0025	KFAV_v1_0025	IMP dehydrogenase	28	24	25	27
KFAV_v1_1887	KFAV_v1_1887	protein of unknown function	28	24	25	27
KFAV_v1_2778	KFAV_v1_2778	Transport permease protein	28	24	25	27
KFAV_v1_0376	KFAV_v1_0376	conserved protein of unknown function	28	24	25	27
KFAV_v1_3329	KFAV_v1_3329	AsnC family transcriptional regulator (modular protein)	28	24	25	27
KFAV_v1_2263	KFAV_v1_2263	Oxidoreductase	28	24	25	27
KFAV_v1_0323	KFAV_v1_0323	protein of unknown function	28	24	25	27
KFAV_v1_2046	KFAV_v1_2046	conserved protein of unknown function	28	24	25	27
yvqK_2	KFAV_v1_2857	putative ATP:cob(I)alamin adenosyltransferase	28	24	25	27
KFAV_v1_1761	KFAV_v1_1761	transposase	28	24	25	27
KFAV_v1_2982	KFAV_v1_2982	Cytochrome P450	28	24	25	27
KFAV_v1_3024	KFAV_v1_3024	protein of unknown function	28	24	25	27
spoIIE	KFAV_v1_0098	Stage II sporulation protein E	28	24	25	26
KFAV_v1_2174	KFAV_v1_2174	Peptidase M20	28	23	25	26
KFAV_v1_0926	KFAV_v1_0926	conserved protein of unknown function	28	23	25	26
KFAV_v1_3052	KFAV_v1_3052	conserved protein of unknown function	28	23	25	26
KFAV_v1_3172	KFAV_v1_3172	protein of unknown function	28	23	25	26
KFAV_v1_3335	KFAV_v1_3335	protein of unknown function	28	23	25	26
KFAV_v1_3437	KFAV_v1_3437	protein of unknown function	28	23	25	26
KFAV_v1_0255	KFAV_v1_0255	3'-5' exoribonuclease YhaM (fragment)	27	23	25	26
acoA_3	KFAV_v1_2591	fragment of acetoin dehydrogenase E1 component (TPP-dependent alpha subunit) (part 1)	27	23	25	26
KFAV_v1_2670	KFAV_v1_2670	conserved membrane protein of unknown function	27	23	25	26
KFAV_v1_2587	KFAV_v1_2587	conserved protein of unknown function	27	23	25	26
sepF	KFAV_v1_1564	cell division machinery factor	27	23	25	26
KFAV_v1_1758	KFAV_v1_1758	Protein hesA (fragment)	27	23	25	26
KFAV_v1_2776	KFAV_v1_2776	membrane protein of unknown function	27	23	25	26
KFAV_v1_3073	KFAV_v1_3073	XRE family transcriptional regulator	27	23	25	26
KFAV_v1_3447	KFAV_v1_3447	Nucleic acid binding protein	27	23	25	25
KFAV_v1_3159	KFAV_v1_3159	conserved protein of unknown function	27	23	24	25
dhaS	KFAV_v1_2349	3-hydroxypropionaldehyde dehydrogenase	27	23	24	25
KFAV_v1_0442	KFAV_v1_0442	conserved protein of unknown function	27	23	24	25
KFAV_v1_1772	KFAV_v1_1772	AAA family ATPase	27	23	24	25
KFAV_v1_1000	KFAV_v1_1000	conserved protein of unknown function	27	23	24	25
KFAV_v1_2935	KFAV_v1_2935	conserved protein of unknown function	27	23	24	25
nnr	KFAV_v1_0220	ADP-dependent (S)-NAD(P)H-hydrate dehydratase / NAD(P)H-hydrate epimerase	27	23	24	25
KFAV_v1_0461	KFAV_v1_0461	conserved protein of unknown function	27	23	24	25

KFAV_v1_0554	KFAV_v1_0554	conserved protein of unknown function	27	23	24	25
KFAV_v1_1022	KFAV_v1_1022	Predicted RNA binding protein YcfA, dsRBD-like fold, HicA-like mRNA interferase family	27	23	24	25
KFAV_v1_1288	KFAV_v1_1288	protein of unknown function	27	23	24	25
KFAV_v1_1365	KFAV_v1_1365	(S)-sulfolactate dehydrogenase	27	22	24	25
dmpN_1	KFAV_v1_2266	Phenol hydroxylase P3 protein	27	22	24	25
KFAV_v1_1538	KFAV_v1_1538	conserved protein of unknown function	27	22	24	25
KFAV_v1_1679	KFAV_v1_1679	Dehydratase	27	22	24	25
KFAV_v1_2272	KFAV_v1_2272	HEPN domain protein	27	22	24	25
odhB	KFAV_v1_2025	2-oxoglutarate dehydrogenase complex (dihydrolipoamide transsuccinylase, E2 subunit)	27	22	24	25
KFAV_v1_0913	KFAV_v1_0913	protein of unknown function	26	22	24	25
KFAV_v1_1838	KFAV_v1_1838	Branched-chain amino acid ABC transporter permease	26	22	24	25
KFAV_v1_2104	KFAV_v1_2104	protein of unknown function	26	22	24	25
glxD	KFAV_v1_3326	Glutamate synthase large subunit-like protein	26	22	24	25
KFAV_v1_1331	KFAV_v1_1331	conserved membrane protein of unknown function	26	22	24	25
KFAV_v1_2438	KFAV_v1_2438	Glycerol-3-phosphate dehydrogenase	26	22	24	25
KFAV_v1_2648	KFAV_v1_2648	conserved exported protein of unknown function	26	22	24	25
KFAV_v1_1016	KFAV_v1_1016	conserved membrane protein of unknown function	26	22	24	25
KFAV_v1_3568	KFAV_v1_3568	conserved protein of unknown function	26	22	24	25
KFAV_v1_0454	KFAV_v1_0454	putative nitronate monooxygenase	26	22	24	25
arsF_2	KFAV_v1_0948	fragment of arsenite/antimonite/H+ antiporter (part 2)	26	22	23	25
KFAV_v1_1600	KFAV_v1_1600	protein of unknown function	26	22	23	24
KFAV_v1_0523	KFAV_v1_0523	DNA helicase	26	22	23	24
acxB_1	KFAV_v1_2257	Acetone carboxylase alpha subunit	26	22	23	24
KFAV_v1_2651	KFAV_v1_2651	Cyclohexanecarboxyl-CoA dehydrogenase (fragment)	26	22	23	24
metXA	KFAV_v1_1117	Homoserine O-acetyltransferase	26	21	23	24
KFAV_v1_2620	KFAV_v1_2620	conserved membrane protein of unknown function	26	21	23	24
KFAV_v1_0381	KFAV_v1_0381	protein of unknown function	26	21	23	24
KFAV_v1_2208	KFAV_v1_2208	conserved protein of unknown function	26	21	23	24
KFAV_v1_2908	KFAV_v1_2908	conserved membrane protein of unknown function	26	21	23	24
KFAV_v1_3378	KFAV_v1_3378	conserved protein of unknown function	26	21	23	24
KFAV_v1_3528	KFAV_v1_3528	protein of unknown function	25	21	23	24
KFAV_v1_3357	KFAV_v1_3357	Dihydropyrimidine dehydrogenase	25	21	23	24
gerE	KFAV_v1_1121	transcriptional regulator required for the expression of late spore coat genes	25	21	23	24
gerW	KFAV_v1_1592	germination-associated protein	25	21	23	24
adh_5	KFAV_v1_2105	Zinc-binding alcohol dehydrogenase	25	21	23	24
yhfA_2	KFAV_v1_0265	fragment of putative transporter (part 1)	25	21	23	24
mmgC_3	KFAV_v1_3555	propionyl-CoA dehydrogenase subunit	25	21	23	24
KFAV_v1_3402	KFAV_v1_3402	ABC transporter permease (fragment)	25	21	23	24
KFAV_v1_0421	KFAV_v1_0421	putative transcriptional regulator	25	21	23	24
KFAV_v1_2372	KFAV_v1_2372	conserved protein of unknown function	25	21	23	24
KFAV_v1_1823	KFAV_v1_1823	Fis family transcriptional regulator	25	21	23	24
KFAV_v1_2246	KFAV_v1_2246	Acyl-CoA dehydrogenase	25	21	23	23
KFAV_v1_2106	KFAV_v1_2106	Acyl-CoA dehydrogenase	25	21	23	23
nrdR	KFAV_v1_0877	negative regulator of transcription of ribonucleotide reductase nrd genes and operons	25	21	23	23
spoVR_2	KFAV_v1_0884	involved in spore cortex synthesis (stage V sporulation, conserved in non sporulating bacteria	25	21	23	23
KFAV_v1_3050	KFAV_v1_3050	Diguanylate phosphodiesterase (fragment)	25	21	22	23
KFAV_v1_0651	KFAV_v1_0651	conserved protein of unknown function	25	21	22	23
KFAV_v1_1316	KFAV_v1_1316	GDSL family lipase	25	21	22	23
KFAV_v1_2609	KFAV_v1_2609	Lactate utilization protein C	25	21	22	23
KFAV_v1_0029	KFAV_v1_0029	MerR family transcriptional regulator	25	21	22	23
fadA_3	KFAV_v1_2319	acetyl-CoA C-acyltransferase	25	20	22	23
KFAV_v1_0479	KFAV_v1_0479	conserved protein of unknown function	24	20	22	23

KFAV_v1_0689	KFAV_v1_0689	EamA family transporter	24	20	22	23
KFAV_v1_0773	KFAV_v1_0773	protein of unknown function	24	20	22	23
KFAV_v1_2672	KFAV_v1_2672	conserved protein of unknown function	24	20	22	23
hprK	KFAV_v1_0266	HPr kinase/phosphorylase	24	20	22	23
KFAV_v1_1463	KFAV_v1_1463	Integrase	24	20	22	23
livF_4	KFAV_v1_2176	leucine/isoleucine/valine transporter subunit ; ATP-binding component of ABC superfamily	24	20	22	23
KFAV_v1_0991	KFAV_v1_0991	GNAT family N-acetyltransferase	24	20	22	23
sigE_2	KFAV_v1_2225	RNA polymerase sporulation-specific sigma-29 factor (sigma-E)	24	20	22	23
KFAV_v1_1804	KFAV_v1_1804	conserved protein of unknown function	24	20	22	23
KFAV_v1_0278	KFAV_v1_0278	CDP-alcohol phosphatidyltransferase	24	20	22	23
KFAV_v1_2443	KFAV_v1_2443	Tautomerase	24	20	22	23
ydcM_2	KFAV_v1_2926	putative transposase	24	20	22	23
tdcB	KFAV_v1_1058	L-threonine ammonia-lyase	24	20	21	23
gbh	KFAV_v1_0297	Guanidinobutyrase	24	20	21	22
KFAV_v1_1308	KFAV_v1_1308	Sigma-54-dependent Fis family transcriptional regulator	24	20	21	22
KFAV_v1_1406	KFAV_v1_1406	conserved protein of unknown function	24	20	21	22
KFAV_v1_0397	KFAV_v1_0397	protein of unknown function	24	20	21	22
KFAV_v1_1036	KFAV_v1_1036	protein of unknown function	24	20	21	22
KFAV_v1_1076	KFAV_v1_1076	putative sulfur carrier protein AF_0556	24	20	21	22
sigE_1	KFAV_v1_1559	RNA polymerase sporulation-specific sigma-29 factor (sigma-E)	24	20	21	22
KFAV_v1_3182	KFAV_v1_3182	protein of unknown function	24	20	21	22
dmpC	KFAV_v1_2448	2-hydroxymuconic semialdehyde dehydrogenase	24	20	21	22
KFAV_v1_1570	KFAV_v1_1570	Sulfonate ABC transporter permease	24	20	21	22
KFAV_v1_0235	KFAV_v1_0235	Thiamine-monophosphate kinase (fragment)	24	20	21	22
KFAV_v1_2212	KFAV_v1_2212	Acyl-CoA synthetase	24	20	21	22
cbiET	KFAV_v1_0831	Cobalt-precorrin-7 C(5)-methyltransferase / Cobalt-precorrin-6B C(15)-methyltransferase (decarboxylating)	24	19	21	22
KFAV_v1_2991	KFAV_v1_2991	4-hydroxyphenylacetate 3-monooxygenase oxygenase subunit	24	19	21	22
rluB	KFAV_v1_1596	23S rRNA pseudouridine 2633 (=2605 standard) pseudouridine synthase	23	19	21	22
KFAV_v1_2454	KFAV_v1_2454	conserved protein of unknown function	23	19	21	22
KFAV_v1_3108	KFAV_v1_3108	protein of unknown function	23	19	21	22
KFAV_v1_3256	KFAV_v1_3256	Small acid-soluble spore protein	23	19	21	21
KFAV_v1_3439	KFAV_v1_3439	protein of unknown function	23	19	21	21
KFAV_v1_2121	KFAV_v1_2121	Ribosomal RNA small subunit methyltransferase E	23	19	21	21
acxC_2	KFAV_v1_2256	Acetone carboxylase gamma subunit	23	19	21	21
KFAV_v1_2774	KFAV_v1_2774	conserved protein of unknown function	23	19	21	21
KFAV_v1_2915	KFAV_v1_2915	conserved protein of unknown function	23	19	21	21
KFAV_v1_2933	KFAV_v1_2933	conserved protein of unknown function	23	19	21	21
KFAV_v1_0653	KFAV_v1_0653	conserved protein of unknown function	23	19	21	21
KFAV_v1_3452	KFAV_v1_3452	DNA-binding transcriptional repressor PuuR (fragment)	23	19	21	21
KFAV_v1_2911	KFAV_v1_2911	FAD:protein FMN transferase	23	19	21	21
KFAV_v1_3204	KFAV_v1_3204	DEAD/DEAH box helicase	23	19	21	21
KFAV_v1_2407	KFAV_v1_2407	protein of unknown function	23	19	21	21
KFAV_v1_1775	KFAV_v1_1775	Fumarylacetoacetase	23	19	21	21
KFAV_v1_2388	KFAV_v1_2388	conserved membrane protein of unknown function	23	19	21	21
KFAV_v1_2909	KFAV_v1_2909	conserved protein of unknown function	23	19	21	21
KFAV_v1_3153	KFAV_v1_3153	AAA family ATPase	23	19	20	21
KFAV_v1_1020	KFAV_v1_1020	MFS transporter	23	19	20	21
KFAV_v1_2173	KFAV_v1_2173	Uncharacterized aminotransferase BpOF4_10225	23	19	20	21
acxB_2	KFAV_v1_2681	Acetone carboxylase alpha subunit	22	19	20	21
shiA	KFAV_v1_0734	Shikimate transporter	22	19	20	21
KFAV_v1_0336	KFAV_v1_0336	conserved protein of unknown function	22	18	20	21
tlp	KFAV_v1_0563	Protein Tlp homolog	22	18	20	20

KFAV_v1_3456	KFAV_v1_3456	Antitoxin	22	18	20	20
KFAV_v1_0274	KFAV_v1_0274	Creatinine amidohydrolase	22	18	20	20
KFAV_v1_1070	KFAV_v1_1070	Shikimate/quinate 5-dehydrogenase (fragment)	22	18	20	20
KFAV_v1_2097	KFAV_v1_2097	transposase (fragment)	22	18	20	20
KFAV_v1_3173	KFAV_v1_3173	protein of unknown function	22	18	20	20
KFAV_v1_1261	KFAV_v1_1261	DNA repair protein RecO (fragment)	22	18	20	20
KFAV_v1_2108	KFAV_v1_2108	Enoyl-CoA hydratase	22	18	20	20
KFAV_v1_2652	KFAV_v1_2652	Cyclohexanecarboxyl-CoA dehydrogenase (fragment)	22	18	20	20
KFAV_v1_3094	KFAV_v1_3094	protein of unknown function	22	18	20	20
ycsA_1	KFAV_v1_2110	putative tartrate dehydrogenase	22	18	20	20
KFAV_v1_2240	KFAV_v1_2240	conserved membrane protein of unknown function	22	18	20	20
KFAV_v1_0638	KFAV_v1_0638	Glycosyl transferase family 2	22	18	20	20
amaB_2	KFAV_v1_2049	N-carbamoyl-L-amino acid hydrolase	22	18	20	20
arsB	KFAV_v1_1385	arsenite efflux transporter; skin element	22	18	20	20
bpsA	KFAV_v1_2671	promiscuous alkylpyrone synthase BpsA (polyketide synthesis)	22	17	20	20
KFAV_v1_3444	KFAV_v1_3444	Integrase	22	17	20	20
KFAV_v1_3440	KFAV_v1_3440	AbrB family transcriptional regulator	21	17	20	20
KFAV_v1_0371	KFAV_v1_0371	MFS transporter	21	17	20	20
chrA_2	KFAV_v1_0486	chromate transporter subunit C	21	17	20	20
KFAV_v1_2098	KFAV_v1_2098	protein of unknown function	21	17	19	19
cobJ	KFAV_v1_0837	Precorrin-3B C(17)-methyltransferase	21	17	19	19
KFAV_v1_1047	KFAV_v1_1047	protein of unknown function	21	17	19	19
lytT_1	KFAV_v1_1111	fragment of two-component response regulator [LytS] (part 1)	21	17	19	19
KFAV_v1_2765	KFAV_v1_2765	putative isocitrate dehydrogenase	21	17	19	19
KFAV_v1_2779	KFAV_v1_2779	Multidrug ABC transporter ATP-binding protein	21	17	19	19
KFAV_v1_2653	KFAV_v1_2653	Cyclohexanecarboxylate-CoA ligase	21	17	19	19
KFAV_v1_1943	KFAV_v1_1943	putative glycosyltransferase	21	17	19	19
KFAV_v1_3178	KFAV_v1_3178	PilT protein domain protein (fragment)	21	16	19	19
hutU	KFAV_v1_0450	urocanase	21	16	19	19
ureD	KFAV_v1_0553	Urease accessory protein UreD	21	16	19	19
cbiX	KFAV_v1_0836	Sirohydrochlorin cobaltochelatase	21	16	19	19
KFAV_v1_1372	KFAV_v1_1372	2-aminoethylphosphonate ABC transporter substrate-binding protein	21	16	19	19
KFAV_v1_0710	KFAV_v1_0710	RNA polymerase subunit sigma	21	16	19	19
KFAV_v1_2512	KFAV_v1_2512	Acyl-CoA synthetase (fragment)	21	16	19	19
KFAV_v1_2650	KFAV_v1_2650	conserved protein of unknown function	21	16	19	19
modA	KFAV_v1_1738	Molybdate ABC transporter substrate-binding protein	21	16	19	19
KFAV_v1_3304	KFAV_v1_3304	conserved protein of unknown function	21	16	19	19
cbaA_1	KFAV_v1_0031	Cytochrome c oxidase subunit 1	21	16	19	19
yvqK_1	KFAV_v1_0932	putative ATP:cob(I)alamin adenosyltransferase	20	16	19	19
KFAV_v1_3222	KFAV_v1_3222	Adenine-specific DNA methylase (fragment)	20	16	19	19
KFAV_v1_0480	KFAV_v1_0480	conserved membrane protein of unknown function	20	15	19	19
KFAV_v1_1762	KFAV_v1_1762	conserved protein of unknown function	20	15	19	19
KFAV_v1_2268	KFAV_v1_2268	Toluene hydroxylase	20	15	19	19
KFAV_v1_2931	KFAV_v1_2931	protein of unknown function	20	15	19	18
KFAV_v1_3186	KFAV_v1_3186	transposase	20	15	19	18
KFAV_v1_1754	KFAV_v1_1754	transposase	20	15	19	18
KFAV_v1_1294	KFAV_v1_1294	conserved protein of unknown function	20	15	19	18
KFAV_v1_2267	KFAV_v1_2267	Monooxygenase	20	15	19	18
flgM	KFAV_v1_3241	Flagellar biosynthesis anti-sigma factor FlgM	20	15	18	18
KFAV_v1_3115	KFAV_v1_3115	Diguanylate cyclase/phosphodiesterase (modular protein)	20	15	18	18
KFAV_v1_0281	KFAV_v1_0281	conserved protein of unknown function	20	15	18	18
KFAV_v1_0823	KFAV_v1_0823	Nickel/cobalt efflux system (fragment)	20	15	18	18

KFAV_v1_2179	KFAV_v1_2179	Amino acid ABC transporter permease	20	15	18	18
cas_2	KFAV_v1_3017	CRISPR-associated endoribonuclease Cas2 3	20	15	18	18
KFAV_v1_1447	KFAV_v1_1447	Spore germination protein	20	15	18	18
rsmD	KFAV_v1_1985	16S rRNA (Guanine(966)-N(2))-methyltransferase RsmD	20	15	18	18
iolA	KFAV_v1_3361	methylmalonate-semialdehyde dehydrogenase	20	15	18	18
spoIVA	KFAV_v1_1654	morphogenetic stage IV sporulation protein	20	15	18	18
KFAV_v1_0200	KFAV_v1_0200	conserved membrane protein of unknown function	19	15	18	18
KFAV_v1_0229	KFAV_v1_0229	conserved membrane protein of unknown function	19	15	18	18
KFAV_v1_0690	KFAV_v1_0690	RNA polymerase sigma factor	19	14	18	18
KFAV_v1_1254	KFAV_v1_1254	conserved protein of unknown function	19	14	18	18
uxaA_1	KFAV_v1_1368	fragment of altronate hydrolase (part 1)	19	14	18	18
KFAV_v1_2676	KFAV_v1_2676	conserved protein of unknown function	19	14	18	18
KFAV_v1_0267	KFAV_v1_0267	putative Catabolite repression HPr-like protein	19	14	18	18
KFAV_v1_2892	KFAV_v1_2892	Transporter (fragment)	19	14	18	18
KFAV_v1_2715	KFAV_v1_2715	Heterodisulfide reductase subunit B	19	14	18	17
ALDH_3	KFAV_v1_2839	Acetaldehyde dehydrogenase	19	14	18	17
KFAV_v1_0990	KFAV_v1_0990	conserved protein of unknown function	19	14	18	17
xylE_2	KFAV_v1_2459	Metapyrocatechase	19	14	18	17
KFAV_v1_2920	KFAV_v1_2920	conserved protein of unknown function	19	14	17	17
KFAV_v1_2621	KFAV_v1_2621	MFS transporter	19	14	17	17
KFAV_v1_1466	KFAV_v1_1466	conserved protein of unknown function	19	14	17	17
KFAV_v1_2709	KFAV_v1_2709	Fe-S oxidoreductase	19	14	17	17
KFAV_v1_2139	KFAV_v1_2139	conserved exported protein of unknown function	19	14	17	17
KFAV_v1_0820	KFAV_v1_0820	conserved protein of unknown function	19	14	17	17
fadM_1	KFAV_v1_0347	Proline dehydrogenase 1	19	14	17	17
KFAV_v1_0603	KFAV_v1_0603	protein of unknown function	19	14	17	17
cbiN	KFAV_v1_0965	Cobalt transport protein CbiN	19	14	17	17
KFAV_v1_2950	KFAV_v1_2950	protein of unknown function	19	14	17	17
ydcW	KFAV_v1_1822	gamma-aminobutyraldehyde dehydrogenase	19	14	17	17
KFAV_v1_1523	KFAV_v1_1523	conserved membrane protein of unknown function	19	14	17	17
KFAV_v1_2560	KFAV_v1_2560	DUF47 domain-containing protein	19	13	17	17
KFAV_v1_0668	KFAV_v1_0668	ABC transporter permease	18	13	17	17
odhA	KFAV_v1_2024	2-oxoglutarate dehydrogenase (E1 subunit)	18	13	17	17
KFAV_v1_1309	KFAV_v1_1309	conserved protein of unknown function	18	13	17	16
dapL	KFAV_v1_2769	LL-diaminopimelate aminotransferase	18	13	17	16
KFAV_v1_3054	KFAV_v1_3054	Efflux RND transporter periplasmic adaptor subunit	18	13	17	16
KFAV_v1_0001	KFAV_v1_0001	protein of unknown function	18	13	16	16
mdh_2	KFAV_v1_2833	malate dehydrogenase (NAD-dependent)	18	13	16	16
hutI	KFAV_v1_0451	imidazolone-5-propionate hydrolase	18	13	16	16
KFAV_v1_1491	KFAV_v1_1491	protein of unknown function	18	13	16	16
KFAV_v1_0536	KFAV_v1_0536	protein of unknown function	18	13	16	16
KFAV_v1_0688	KFAV_v1_0688	Transcriptional regulator	18	13	16	16
KFAV_v1_1580	KFAV_v1_1580	protein of unknown function	18	13	16	16
KFAV_v1_1852	KFAV_v1_1852	conserved protein of unknown function	18	13	16	16
KFAV_v1_2074	KFAV_v1_2074	RNHCP domain-containing protein	18	13	16	16
KFAV_v1_2437	KFAV_v1_2437	3-hydroxybutyryl-CoA dehydrogenase	18	13	16	16
KFAV_v1_0406	KFAV_v1_0406	conserved protein of unknown function	18	13	16	16
KFAV_v1_2441	KFAV_v1_2441	conserved protein of unknown function	18	13	16	16
KFAV_v1_0273	KFAV_v1_0273	Ferredoxin	17	13	16	16
KFAV_v1_1081	KFAV_v1_1081	Glutamate-1-semialdehyde 2,1-aminomutase	17	13	16	16
KFAV_v1_1293	KFAV_v1_1293	transposase (fragment)	17	13	16	16
KFAV_v1_2112	KFAV_v1_2112	GntR family transcriptional regulator	17	13	16	16

KFAV_v1_2260	KFAV_v1_2260	Plasmid stabilization system	17	13	16	16
KFAV_v1_2924	KFAV_v1_2924	protein of unknown function	17	13	16	16
paaG_3	KFAV_v1_3072	fragment of acyl-CoA hydratase (part 1)	17	12	16	16
pxpC	KFAV_v1_3084	L-5-oxoprolinase (ATP-dependent) subunit C	17	12	16	16
KFAV_v1_2759	KFAV_v1_2759	conserved membrane protein of unknown function	17	12	16	16
patA_2	KFAV_v1_2479	Putrescine aminotransferase	17	12	16	16
KFAV_v1_3016	KFAV_v1_3016	conserved membrane protein of unknown function	17	12	16	15
KFAV_v1_2242	KFAV_v1_2242	CoA-binding domain protein	17	12	16	15
KFAV_v1_0327	KFAV_v1_0327	transposase (fragment)	17	12	16	15
KFAV_v1_3187	KFAV_v1_3187	conserved protein of unknown function	17	12	16	15
KFAV_v1_2379	KFAV_v1_2379	Error-prone DNA polymerase (fragment)	17	12	16	15
KFAV_v1_2886	KFAV_v1_2886	Sugar fermentation stimulation protein homolog (fragment)	17	12	15	15
KFAV_v1_2802	KFAV_v1_2802	conserved protein of unknown function	17	12	15	15
yvyE	KFAV_v1_3473	IMPACT family member YvyE	17	12	15	15
KFAV_v1_3096	KFAV_v1_3096	Aliphatic amidase	17	12	15	15
KFAV_v1_0252	KFAV_v1_0252	protein of unknown function	16	12	15	15
KFAV_v1_0993	KFAV_v1_0993	Lantibiotic ABC transporter ATP-binding protein	16	12	15	15
KFAV_v1_2882	KFAV_v1_2882	Chloride channel protein	16	12	15	15
KFAV_v1_1741	KFAV_v1_1741	FAD dependent oxidoreductase	16	12	15	15
KFAV_v1_3366	KFAV_v1_3366	Phosphonate C-P lyase system protein PhnL	16	12	15	14
hyuB	KFAV_v1_3164	putative D-/L-hydantoinase subunit B	16	12	15	14
KFAV_v1_0815	KFAV_v1_0815	conserved protein of unknown function	16	12	15	14
KFAV_v1_3395	KFAV_v1_3395	Aminopeptidase	16	12	15	14
KFAV_v1_3344	KFAV_v1_3344	Sigma-54-dependent Fis family transcriptional regulator	16	12	15	14
KFAV_v1_0218	KFAV_v1_0218	conserved membrane protein of unknown function	16	12	15	14
KFAV_v1_2432	KFAV_v1_2432	conserved protein of unknown function	16	12	15	14
KFAV_v1_0226	KFAV_v1_0226	putative enzyme	16	12	15	14
KFAV_v1_1024	KFAV_v1_1024	Copper resistance protein CopC	15	11	15	14
KFAV_v1_2193	KFAV_v1_2193	conserved protein of unknown function	15	11	15	14
KFAV_v1_2211	KFAV_v1_2211	2-phosphosulfolactate phosphatase	15	11	15	14
prkA_2	KFAV_v1_0881	serine protein kinase (involved in sporulation)	15	11	15	14
KFAV_v1_0841	KFAV_v1_0841	Cobyric acid synthase (fragment)	15	11	15	14
KFAV_v1_0526	KFAV_v1_0526	DNA modification methylase	15	11	15	14
KFAV_v1_0485	KFAV_v1_0485	Transporter	15	11	15	14
KFAV_v1_0994	KFAV_v1_0994	Lantibiotic immunity ABC transporter MutE/EpiE family permease subunit	15	11	15	14
uxaA_2	KFAV_v1_1369	fragment of altronate hydrolase (part 2)	15	11	15	14
KFAV_v1_1371	KFAV_v1_1371	DeoR/GlpR transcriptional regulator	15	11	14	14
cobB	KFAV_v1_2973	NAD-dependent protein deacetylase	15	11	14	14
KFAV_v1_2979	KFAV_v1_2979	Transport permease protein	15	11	14	14
KFAV_v1_2716	KFAV_v1_2716	Heterodisulfide reductase subunit C	15	11	14	14
KFAV_v1_0737	KFAV_v1_0737	Sulfurtransferase	15	11	14	14
KFAV_v1_1084	KFAV_v1_1084	protein of unknown function	15	11	14	14
KFAV_v1_2535	KFAV_v1_2535	Glyoxalase	15	11	14	14
sigG	KFAV_v1_1560	RNA polymerase sporulation-specific sigma factor (sigma-G)	15	11	14	14
KFAV_v1_0593	KFAV_v1_0593	Gluconeogenesis factor	15	11	14	14
KFAV_v1_0946	KFAV_v1_0946	Gamma-glutamylcyclotransferase	15	11	14	14
pxpA_1	KFAV_v1_0370	oxoprolinase subunit A	14	11	14	14
mtnU	KFAV_v1_3379	Hydrolase MtnU	14	11	14	13
KFAV_v1_3482	KFAV_v1_3482	M23 family peptidase	14	11	14	13
KFAV_v1_0404	KFAV_v1_0404	Type II secretory pathway, component ExeA (Predicted ATPase)	14	10	14	13
KFAV_v1_2376	KFAV_v1_2376	conserved protein of unknown function	14	10	14	13
KFAV_v1_0408	KFAV_v1_0408	transposase (fragment)	14	10	14	13

KFAV_v1_2408	KFAV_v1_2408	conserved membrane protein of unknown function	14	10	14	13
KFAV_v1_3119	KFAV_v1_3119	phosphate transporter subunit ; periplasmic-binding component of ABC superfamily (fragment)	14	10	14	13
KFAV_v1_0955	KFAV_v1_0955	transposase (fragment)	14	10	14	13
KFAV_v1_2617	KFAV_v1_2617	conserved exported protein of unknown function	14	10	14	13
KFAV_v1_2829	KFAV_v1_2829	Amino acid transporter	14	10	14	13
cbiF	KFAV_v1_0833	Cobalt-precorrin-4 C(11)-methyltransferase	14	10	14	13
KFAV_v1_1386	KFAV_v1_1386	Transcriptional regulator	14	10	14	13
KFAV_v1_2605	KFAV_v1_2605	conserved membrane protein of unknown function	14	10	13	13
KFAV_v1_3114	KFAV_v1_3114	Aminotransferase, class IV	14	10	13	13
KFAV_v1_2564	KFAV_v1_2564	conserved membrane protein of unknown function	14	10	13	13
KFAV_v1_1797	KFAV_v1_1797	protein of unknown function	14	10	13	13
yybF	KFAV_v1_3092	putative permease	14	10	13	13
KFAV_v1_1021	KFAV_v1_1021	conserved protein of unknown function	14	10	13	12
KFAV_v1_1083	KFAV_v1_1083	Major facilitator superfamily MFS_1	14	10	13	12
lonB	KFAV_v1_1128	spore-specific ATP-dependent protease LonB	14	10	13	12
nfo	KFAV_v1_1446	putative endonuclease 4	14	10	13	12
KFAV_v1_2847	KFAV_v1_2847	Ethanolamine utilization protein EutP	14	10	13	12
pgoN_1	KFAV_v1_0028	promiscuous glyoxal/methylglyoxal reductase	14	10	13	12
KFAV_v1_0967	KFAV_v1_0967	ABC transporter ATP-binding protein	13	9	13	12
KFAV_v1_1364	KFAV_v1_1364	Putative nickel insertion protein (fragment)	13	9	13	12
KFAV_v1_2352	KFAV_v1_2352	conserved protein of unknown function	13	9	13	12
KFAV_v1_0669	KFAV_v1_0669	conserved protein of unknown function	13	9	13	12
spoVAB	KFAV_v1_1576	stage V sporulation protein AB	13	9	13	12
KFAV_v1_2977	KFAV_v1_2977	Multidrug transporter	13	9	13	12
gcvH_3	KFAV_v1_2708	Glycine cleavage system H protein 2	13	9	13	12
KFAV_v1_1285	KFAV_v1_1285	protein of unknown function	13	9	13	12
KFAV_v1_0382	KFAV_v1_0382	Diguanylate phosphodiesterase	13	9	13	12
KFAV_v1_0822	KFAV_v1_0822	Nickel/cobalt efflux system (fragment)	13	9	13	12
KFAV_v1_3002	KFAV_v1_3002	conserved membrane protein of unknown function	13	9	13	12
cphA	KFAV_v1_0067	Cyanophycin synthetase	13	9	13	12
KFAV_v1_0445	KFAV_v1_0445	Amidohydrolase	13	9	12	12
KFAV_v1_0458	KFAV_v1_0458	CoA-disulfide reductase	13	9	12	12
fadM_2	KFAV_v1_2644	Long-chain acyl-CoA thioesterase FadM	13	9	12	11
KFAV_v1_2777	KFAV_v1_2777	MFS transporter (fragment)	13	9	12	11
KFAV_v1_1310	KFAV_v1_1310	transposase	13	9	12	11
KFAV_v1_3195	KFAV_v1_3195	conserved protein of unknown function	13	9	12	11
phnX	KFAV_v1_1375	Phosphonoacetaldehyde hydrolase	13	9	12	11
KFAV_v1_0619	KFAV_v1_0619	MerR family transcriptional regulator	13	9	12	11
KFAV_v1_2756	KFAV_v1_2756	conserved protein of unknown function	12	8	12	11
KFAV_v1_2134	KFAV_v1_2134	Phenylacetate-CoA oxygenase, PaaJ subunit	12	8	12	11
KFAV_v1_1631	KFAV_v1_1631	conserved membrane protein of unknown function	12	8	12	11
KFAV_v1_3010	KFAV_v1_3010	Dipeptidase	12	8	12	11
KFAV_v1_3093	KFAV_v1_3093	conserved protein of unknown function	12	8	12	11
KFAV_v1_0873	KFAV_v1_0873	Glutamine synthetase	12	8	12	11
KFAV_v1_2677	KFAV_v1_2677	conserved protein of unknown function	12	8	12	11
KFAV_v1_1558	KFAV_v1_1558	conserved membrane protein of unknown function	12	8	12	11
cbaB_1	KFAV_v1_0032	Cytochrome c oxidase subunit 2	12	8	12	11
ispF	KFAV_v1_0140	2-C-methyl-D-erythritol-2,4-cyclodiphosphate synthase	12	8	12	11
KFAV_v1_0711	KFAV_v1_0711	conserved protein of unknown function	12	8	12	11
KFAV_v1_0800	KFAV_v1_0800	membrane protein of unknown function	12	8	12	11
KFAV_v1_3107	KFAV_v1_3107	Spore gernimation protein	12	8	12	11
KFAV_v1_0801	KFAV_v1_0801	putative integral inner membrane protein (fragment)	12	8	12	11

KFAV_v1_0997	KFAV_v1_0997	Sensor histidine kinase	12	8	12	11
KFAV_v1_2481	KFAV_v1_2481	conserved membrane protein of unknown function	12	8	12	11
KFAV_v1_1295	KFAV_v1_1295	conserved protein of unknown function	12	7	12	11
puuC	KFAV_v1_2983	gamma-Glu-gamma-aminobutyraldehyde dehydrogenase, NAD(P)H-dependent	12	7	12	11
KFAV_v1_0818	KFAV_v1_0818	Drug resistance transporter, EmrB/QacA subfamily (fragment)	12	7	11	11
KFAV_v1_2027	KFAV_v1_2027	Sigma-54-dependent Fis family transcriptional regulator	12	7	11	11
KFAV_v1_0580	KFAV_v1_0580	Gfo/Idh/MocA family oxidoreductase	12	7	11	10
tmoA	KFAV_v1_2461	Toluene-4-monooxygenase system, hydroxylase component subunit alpha	12	7	11	10
acsA_3	KFAV_v1_2679	Acetyl-coenzyme A synthetase	12	7	11	10
KFAV_v1_2689	KFAV_v1_2689	Formate dehydrogenase	12	7	11	10
KFAV_v1_2064	KFAV_v1_2064	conserved protein of unknown function	12	7	11	10
acxC_3	KFAV_v1_2680	Acetone carboxylase gamma subunit	12	7	11	10
bkdAA	KFAV_v1_2086	branched-chain alpha-keto acid dehydrogenase E1 subunit	11	7	11	10
dmpN_2	KFAV_v1_2455	Phenol hydroxylase P3 protein	11	7	11	10
KFAV_v1_2606	KFAV_v1_2606	conserved protein of unknown function	11	7	11	10
KFAV_v1_2840	KFAV_v1_2840	conserved protein of unknown function	11	7	11	10
KFAV_v1_2426	KFAV_v1_2426	Flavin-dependent oxidoreductase, luciferase family (Includes alkanesulfonate monooxygenase SsuD and methylene tetrahydromethanopterin reductase) (fragment)	11	7	11	10
KFAV_v1_3442	KFAV_v1_3442	conserved protein of unknown function	11	7	11	10
cotE	KFAV_v1_1803	morphogenic spore protein	11	7	11	10
yqeG	KFAV_v1_2169	phosphatase (active on GMP and Glc-6-P)	11	7	11	10
KFAV_v1_0679	KFAV_v1_0679	conserved membrane protein of unknown function	11	6	11	10
KFAV_v1_1367	KFAV_v1_1367	Malate/lactate/ureidoglycolate dehydrogenase, LDH2 family	11	6	11	10
rlmH	KFAV_v1_3576	23S rRNA (pseudouridine1915-N3)-methyltransferase	11	6	11	10
KFAV_v1_2344	KFAV_v1_2344	conserved exported protein of unknown function	11	6	11	10
KFAV_v1_0632	KFAV_v1_0632	Polysaccharide deacetylase	11	6	11	10
spmB	KFAV_v1_1595	spore maturation protein	11	6	11	10
bcd	KFAV_v1_2087	branched-chain amino acid dehydrogenase	11	6	10	10
clpC_2	KFAV_v1_3097	fragment of class III stress response-related ATPase, AAA+ superfamily (part 1)	11	6	10	10
KFAV_v1_3323	KFAV_v1_3323	Myristoyl transferase	11	6	10	10
KFAV_v1_0705	KFAV_v1_0705	conserved protein of unknown function	11	6	10	10
KFAV_v1_0268	KFAV_v1_0268	membrane protein of unknown function	10	6	10	10
KFAV_v1_2061	KFAV_v1_2061	Conjugal transfer protein TraR	10	6	10	10
KFAV_v1_2457	KFAV_v1_2457	Methane/phenol/toluene hydroxylase	10	6	10	9
KFAV_v1_2197	KFAV_v1_2197	conserved protein of unknown function	10	6	10	9
KFAV_v1_0367	KFAV_v1_0367	Acetolactate synthase	10	6	10	9
KFAV_v1_3206	KFAV_v1_3206	protein of unknown function	10	6	10	9
KFAV_v1_1476	KFAV_v1_1476	Peptidase U35	10	6	10	9
KFAV_v1_1706	KFAV_v1_1706	conserved protein of unknown function	10	6	10	9
yhcA_1	KFAV_v1_2472	fragment of putative exporter (benzoate transcriptome) (part 3)	10	6	10	9
dhaT	KFAV_v1_1312	1,3-propanediol dehydrogenase	10	6	10	9
KFAV_v1_2814	KFAV_v1_2814	RsfA family transcriptional regulator	10	6	10	9
dprA	KFAV_v1_1926	DNA-protecting protein DprA	10	5	10	9
KFAV_v1_2133	KFAV_v1_2133	Transcriptional regulator, TetR family	10	5	10	9
KFAV_v1_2347	KFAV_v1_2347	ABC transporter	10	5	10	9
KFAV_v1_1518	KFAV_v1_1518	conserved protein of unknown function	10	5	10	9
KFAV_v1_3264	KFAV_v1_3264	protein of unknown function	10	5	10	9
KFAV_v1_2695	KFAV_v1_2695	Radical SAM protein	10	5	9	9
KFAV_v1_0026	KFAV_v1_0026	TetR family transcriptional regulator	10	5	9	9
KFAV_v1_0812	KFAV_v1_0812	Multidrug transporter	10	5	9	9
KFAV_v1_1351	KFAV_v1_1351	Adenine deaminase	10	5	9	9
KFAV_v1_1315	KFAV_v1_1315	membrane protein of unknown function	10	4	9	9
KFAV_v1_1477	KFAV_v1_1477	HK97 family phage portal protein	10	4	9	9

KFAV_v1_2434	KFAV_v1_2434	Renal dipeptidase family protein	10	4	9	9
KFAV_v1_2770	KFAV_v1_2770	conserved protein of unknown function	10	3	9	9
cutR	KFAV_v1_1030	transcriptional regulator of copper intake (CutR-Cu(+))	10	0	9	9
KFAV_v1_3573	KFAV_v1_3573	ABC transporter related protein	10	0	9	9
KFAV_v1_1209	KFAV_v1_1209	conserved membrane protein of unknown function	9	0	9	9
potA_2	KFAV_v1_1373	Spermidine/putrescine import ATP-binding protein PotA	9	0	9	9
KFAV_v1_1771	KFAV_v1_1771	conserved protein of unknown function	9	0	9	9
KFAV_v1_3377	KFAV_v1_3377	protein of unknown function	9	0	9	9
pabB	KFAV_v1_2262	Aminodeoxychorismate synthase, component I	9	0	9	9
KFAV_v1_1026	KFAV_v1_1026	conserved membrane protein of unknown function	9	0	9	8
clpC_3	KFAV_v1_3098	fragment of class III stress response-related ATPase, AAA+ superfamily (part 2)	9	0	9	8
ylbJ	KFAV_v1_1714	Sporulation integral membrane protein YlbJ	9	0	9	8
KFAV_v1_2282	KFAV_v1_2282	Molybdenum cofactor guanylyltransferase	9	0	9	8
KFAV_v1_1141	KFAV_v1_1141	conserved protein of unknown function	9	0	9	8
KFAV_v1_1233	KFAV_v1_1233	Peptidase S8 and S53 subtilisin kexin sedolisin	9	0	9	8
KFAV_v1_0671	KFAV_v1_0671	Glycerol-3-phosphate responsive antiterminator	9	0	9	8
KFAV_v1_2712	KFAV_v1_2712	conserved protein of unknown function	9	0	9	8
KFAV_v1_0270	KFAV_v1_0270	conserved membrane protein of unknown function	9	0	9	8
KFAV_v1_0331	KFAV_v1_0331	Pyridine nucleotide-disulfide oxidoreductase	9	0	8	8
KFAV_v1_1019	KFAV_v1_1019	protein of unknown function	9	0	8	8
KFAV_v1_3445	KFAV_v1_3445	transposase	9	0	8	8
KFAV_v1_1333	KFAV_v1_1333	Acetylornithine deacetylase or succinyl-diaminopimelate desuccinylase	9	0	8	8
eutL	KFAV_v1_2841	putative carboxysome-related structural protein with putative role in ethanolamine utilization	9	0	8	8
yjbE_1	KFAV_v1_0487	Uncharacterized membrane protein YjbE	9	0	8	8
KFAV_v1_1796	KFAV_v1_1796	conserved protein of unknown function	9	0	8	8
KFAV_v1_2202	KFAV_v1_2202	Glycosyl transferase family 2	9	0	8	8
KFAV_v1_1210	KFAV_v1_1210	conserved protein of unknown function	8	0	8	8
eutB_1	KFAV_v1_2831	ethanolamine ammonia-lyase, large subunit, heavy chain	8	0	8	8
KFAV_v1_2981	KFAV_v1_2981	L-glutamine synthetase	8	0	8	8
KFAV_v1_3057	KFAV_v1_3057	DNA-binding protein	8	0	8	8
KFAV_v1_1320	KFAV_v1_1320	conserved exported protein of unknown function	8	0	8	8
KFAV_v1_1028	KFAV_v1_1028	Putative Cytochrome C	8	0	8	8
fumC	KFAV_v1_0938	Fumarate hydratase class II	8	0	8	8
cas_3	KFAV_v1_3019	CRISPR-associated protein Cas4	8	0	8	7
KFAV_v1_0972	KFAV_v1_0972	conserved protein of unknown function	8	0	8	7
spaR	KFAV_v1_0996	Transcriptional regulatory protein SpaR	8	0	8	7
KFAV_v1_3355	KFAV_v1_3355	D-hydantoinase	8	0	8	7
KFAV_v1_1448	KFAV_v1_1448	conserved protein of unknown function	8	0	8	7
KFAV_v1_3055	KFAV_v1_3055	Hemolysin D	8	0	8	7
KFAV_v1_1454	KFAV_v1_1454	Two-component sensor histidine kinase	8	0	8	7
KFAV_v1_1674	KFAV_v1_1674	conserved protein of unknown function	8	0	8	7
KFAV_v1_2028	KFAV_v1_2028	Glutamate--ammonia ligase	8	0	8	7
KFAV_v1_0733	KFAV_v1_0733	Transcriptional regulator	8	0	8	7
yhxA	KFAV_v1_3359	putative aminotransferase	8	0	8	7
KFAV_v1_0649	KFAV_v1_0649	transposase (fragment)	8	0	8	7
KFAV_v1_2424	KFAV_v1_2424	Glutamyl-tRNA amidotransferase	8	0	8	7
acxA_2	KFAV_v1_2682	Acetone carboxylase beta subunit	8	0	8	7
KFAV_v1_3174	KFAV_v1_3174	Aspartate aminotransferase family protein	8	0	8	7
cbiA	KFAV_v1_0835	Cobyrinate a,c-diamide synthase	8	0	8	7
KFAV_v1_1366	KFAV_v1_1366	Transcription regulator HTH, GntR	8	0	8	7
sleB_1	KFAV_v1_1449	Spore cortex-lytic enzyme	8	0	7	7
acxA_1	KFAV_v1_2258	Acetone carboxylase beta subunit	8	0	7	6

KFAV_v1_0636	KFAV_v1_0636	Drug resistance transporter, EmrB/QacA subfamily	8	0	7	6
fabG_3	KFAV_v1_1831	beta-ketoacyl-acyl carrier protein reductase	8	0	7	6
KFAV_v1_1329	KFAV_v1_1329	conserved protein of unknown function	8	0	7	6
livF_1	KFAV_v1_0781	leucine/isoleucine/valine transporter subunit ; ATP-binding component of ABC superfamily	8	0	7	6
cbiM	KFAV_v1_0964	Cobalt transport protein CbiM	8	0	7	6
fabG_5	KFAV_v1_2433	3-oxoacyl-[acyl-carrier-protein] reductase FabG	8	0	7	6
actP_3	KFAV_v1_2440	Cation acetate symporter	8	0	7	6
KFAV_v1_3056	KFAV_v1_3056	MFS transporter	8	0	7	6
KFAV_v1_2764	KFAV_v1_2764	IclR family transcriptional regulator	8	0	7	6
KFAV_v1_3263	KFAV_v1_3263	conserved protein of unknown function	8	0	7	6
KFAV_v1_1460	KFAV_v1_1460	conserved protein of unknown function	8	0	7	6
KFAV_v1_3358	KFAV_v1_3358	NCS1 nucleoside transporter family protein	8	0	7	6
nupO	KFAV_v1_1341	guanosine ABC transporter (ATP-binding protein)	8	0	7	6
KFAV_v1_2711	KFAV_v1_2711	Electron transfer flavoprotein subunit beta	8	0	7	6
putC_1	KFAV_v1_0346	1-pyrroline-5-carboxylate dehydrogenase	7	0	7	6
putC_2	KFAV_v1_0940	1-pyrroline-5-carboxylate dehydrogenase	7	0	7	6
tepA	KFAV_v1_1821	protein export-enhancing protease (spore outgrowth)	7	0	7	6
KFAV_v1_2792	KFAV_v1_2792	membrane protein of unknown function	7	0	7	6
KFAV_v1_2203	KFAV_v1_2203	conserved protein of unknown function	7	0	7	6
KFAV_v1_2688	KFAV_v1_2688	4Fe-4S ferredoxin	7	0	7	6
aqpM	KFAV_v1_0463	putative aquaporin AqpM	7	0	7	6
yfkQ_1	KFAV_v1_0248	putative spore germination protein	7	0	7	6
yfkQ_2	KFAV_v1_0641	Uncharacterized membrane protein YfkQ	7	0	6	6
cbiQ	KFAV_v1_0966	Cobalt ECF transporter T component CbiQ	7	0	6	6
KFAV_v1_1676	KFAV_v1_1676	Enoyl-CoA hydratase/isomerase family protein	7	0	6	6
KFAV_v1_0269	KFAV_v1_0269	Transcriptional regulator	7	0	6	6
paaG_1	KFAV_v1_2130	acyl-CoA hydratase	7	0	6	6
KFAV_v1_0765	KFAV_v1_0765	conserved protein of unknown function	7	0	6	6
KFAV_v1_2673	KFAV_v1_2673	NLP/P60 protein	7	0	6	5
KFAV_v1_2984	KFAV_v1_2984	Acetoacetate decarboxylase	7	0	6	5
KFAV_v1_2844	KFAV_v1_2844	Ethanolamine utilization protein	7	0	6	5
KFAV_v1_2986	KFAV_v1_2986	4-hydroxyphenylacetate degradation bifunctional isomerase/decarboxylase subunit HpaG2	7	0	6	5
KFAV_v1_1335	KFAV_v1_1335	Transcriptional regulator, PucR family	7	0	6	5
KFAV_v1_2632	KFAV_v1_2632	Peptide ABC transporter substrate-binding protein	7	0	6	5
KFAV_v1_2612	KFAV_v1_2612	Manganese ABC transporter permease	7	0	6	5
KFAV_v1_2655	KFAV_v1_2655	IclR family transcriptional regulator	7	0	6	5
KFAV_v1_3168	KFAV_v1_3168	Fis family transcriptional regulator	7	0	6	5
KFAV_v1_0299	KFAV_v1_0299	Carbon-nitrogen hydrolase family protein	7	0	6	5
mutM	KFAV_v1_2629	formamidopyrimidine-DNA glycosylase; 5-hydroxymethyl-uracil DNA-glycosylase	7	0	6	5
yhdF	KFAV_v1_2686	putative NAD(P)-dependent dehydrogenase	7	0	6	5
KFAV_v1_0631	KFAV_v1_0631	Polysaccharide deacetylase family protein	7	0	6	5
alsR	KFAV_v1_1378	HTH-type transcriptional regulator AlsR	6	0	6	5
yhcA_4	KFAV_v1_3372	putative exporter (benzoate transcriptome)	6	0	6	5
KFAV_v1_1161	KFAV_v1_1161	conserved membrane protein of unknown function	6	0	6	5
yybE	KFAV_v1_3091	putative transcriptional regulator (LysR family)	6	0	6	5
KFAV_v1_3322	KFAV_v1_3322	ABC transporter permease	6	0	6	5
cyeB	KFAV_v1_3090	cysteine and O-acetylserine efflux permease	6	0	6	4
KFAV_v1_0803	KFAV_v1_0803	conserved protein of unknown function	6	0	6	4
KFAV_v1_2189	KFAV_v1_2189	NAD-dependent epimerase/dehydratase	6	0	6	4
KFAV_v1_2614	KFAV_v1_2614	Zinc ABC transporter substrate-binding protein	6	0	5	4
KFAV_v1_1327	KFAV_v1_1327	conserved protein of unknown function	6	0	5	4
KFAV_v1_3040	KFAV_v1_3040	Glutathione ABC transporter permease GsiC	6	0	5	4

livH_1	KFAV_v1_0778	leucine/isoleucine/valine transporter subunit ; membrane component of ABC superfamily	6	0	5	4
KFAV_v1_2693	KFAV_v1_2693	conserved membrane protein of unknown function	6	0	5	4
galE_1	KFAV_v1_0359	UDP-glucose 4-epimerase	6	0	5	4
KFAV_v1_3058	KFAV_v1_3058	conserved protein of unknown function	6	0	5	4
KFAV_v1_1484	KFAV_v1_1484	conserved protein of unknown function	6	0	5	4
KFAV_v1_2694	KFAV_v1_2694	Radical SAM protein	6	0	5	4
pucL_2	KFAV_v1_1337	fragment of urate oxidase with peroxide reductase N-terminal domain (part 2)	6	0	5	4
pdhB_4	KFAV_v1_1833	pyruvate dehydrogenase (E1 beta subunit)	6	0	5	3
KFAV_v1_2187	KFAV_v1_2187	NAD-dependent dehydratase	6	0	5	3
KFAV_v1_1794	KFAV_v1_1794	conserved protein of unknown function	6	0	5	3
KFAV_v1_3189	KFAV_v1_3189	conserved membrane protein of unknown function	6	0	5	0
KFAV_v1_0939	KFAV_v1_0939	conserved protein of unknown function	6	0	5	0
KFAV_v1_1099	KFAV_v1_1099	conserved protein of unknown function	6	0	5	0
hyuA	KFAV_v1_3165	putative D-/L-hydantoinase subunit A	6	0	5	0
ltrA_3	KFAV_v1_0916	Group II intron reverse transcriptase/maturase	6	0	5	0
KFAV_v1_2192	KFAV_v1_2192	conserved protein of unknown function	6	0	4	0
KFAV_v1_3575	KFAV_v1_3575	Extracellular solute-binding protein family 1	6	0	4	0
gpr	KFAV_v1_2150	spore germination protease	5	0	4	0
KFAV_v1_2706	KFAV_v1_2706	Biotin/lipoate A/B protein ligase	5	0	4	0
adh_7	KFAV_v1_2460	Alcohol dehydrogenase	5	0	4	0
pdhA_3	KFAV_v1_1834	pyruvate dehydrogenase (E1 alpha subunit)	5	0	4	0
KFAV_v1_1029	KFAV_v1_1029	conserved membrane protein of unknown function	5	0	4	0
KFAV_v1_2149	KFAV_v1_2149	conserved exported protein of unknown function	5	0	4	0
ald_2	KFAV_v1_3360	L-alanine dehydrogenase (NAD-dependent)	5	0	4	0
KFAV_v1_2714	KFAV_v1_2714	Pyridine nucleotide-disulfide oxidoreductase	5	0	4	0
KFAV_v1_0306	KFAV_v1_0306	Spore gernimation protein	5	0	4	0
KFAV_v1_0349	KFAV_v1_0349	Fic family protein	5	0	4	0
phnW	KFAV_v1_1377	2-aminoethylphosphonate--pyruvate transaminase	5	0	4	0
mmgC_2	KFAV_v1_2510	Acyl-CoA dehydrogenase	5	0	4	0
speB_1	KFAV_v1_1326	putative agmatinase 2	5	0	4	0
KFAV_v1_3218	KFAV_v1_3218	conserved protein of unknown function	5	0	3	0
amiE	KFAV_v1_3003	Aliphatic amidase	5	0	3	0
cbiD	KFAV_v1_0830	Cobalt-precorrin-5B C(1)-methyltransferase	5	0	3	0
KFAV_v1_0813	KFAV_v1_0813	ABC transporter permease	5	0	3	0
KFAV_v1_3247	KFAV_v1_3247	Spore gernimation protein	5	0	3	0
KFAV_v1_1737	KFAV_v1_1737	conserved protein of unknown function	5	0	2	0
rocD	KFAV_v1_1429	ornithine aminotransferase	5	0	0	0
KFAV_v1_1342	KFAV_v1_1342	Inner-membrane translocator	5	0	0	0
KFAV_v1_0777	KFAV_v1_0777	Extracellular ligand-binding receptor	5	0	0	0
KFAV_v1_3036	KFAV_v1_3036	Zn-dependent hydrolase	5	0	0	0
KFAV_v1_2482	KFAV_v1_2482	conserved protein of unknown function	5	0	0	0
gltP	KFAV_v1_1082	proton/glutamate symport protein	5	0	0	0
KFAV_v1_2435	KFAV_v1_2435	Rieske (2Fe-2S) protein	5	0	0	0
KFAV_v1_1370	KFAV_v1_1370	conserved membrane protein of unknown function	5	0	0	0
preA_2	KFAV_v1_3356	NAD-dependent dihydropyrimidine dehydrogenase subunit PreA	5	0	0	0
aprX	KFAV_v1_0723	Serine protease AprX	4	0	0	0
dadD	KFAV_v1_1339	5'-deoxyadenosine deaminase	4	0	0	0
KFAV_v1_2439	KFAV_v1_2439	Major facilitator superfamily MFS_1	4	0	0	0
KFAV_v1_2471	KFAV_v1_2471	MFS transporter	4	0	0	0
KFAV_v1_0436	KFAV_v1_0436	Major facilitator superfamily MFS_1	4	0	0	0
KFAV_v1_1802	KFAV_v1_1802	conserved protein of unknown function	4	0	0	0
sleC	KFAV_v1_1638	spore germination membrane component	4	0	0	0

KFAV_v1_0779	KFAV_v1_0779	Inner-membrane translocator	4	0	0	0
KFAV_v1_1801	KFAV_v1_1801	conserved protein of unknown function	4	0	0	0
eutB_2	KFAV_v1_2843	ethanolamine ammonia-lyase, large subunit, heavy chain	4	0	0	0
sudA	KFAV_v1_3318	Sulfide dehydrogenase subunit alpha	4	0	0	0
spoIIIAE	KFAV_v1_1208	Stage III sporulation protein AE	4	0	0	0
KFAV_v1_2987	KFAV_v1_2987	conserved membrane protein of unknown function	4	0	0	0
ykcB_2	KFAV_v1_0328	fragment of putative membrane glycosyl transferase (part 1)	4	0	0	0
KFAV_v1_0214	KFAV_v1_0214	Spore germination protein	4	0	0	0
KFAV_v1_0687	KFAV_v1_0687	Amino acid permease	4	0	0	0
KFAV_v1_2196	KFAV_v1_2196	Ferredoxin-dependent glutamate synthase	4	0	0	0
KFAV_v1_2353	KFAV_v1_2353	conserved protein of unknown function	4	0	0	0
KFAV_v1_2036	KFAV_v1_2036	Hydantoinase subunit beta	4	0	0	0
KFAV_v1_1328	KFAV_v1_1328	Amino acid permease-associated region (modular protein)	4	0	0	0
KFAV_v1_1944	KFAV_v1_1944	conserved protein of unknown function	4	0	0	0
spoVB	KFAV_v1_2405	Stage V sporulation protein B	3	0	0	0
KFAV_v1_1703	KFAV_v1_1703	Helicase	3	0	0	0
KFAV_v1_1374	KFAV_v1_1374	Phosphonate ABC transporter permease (modular protein)	3	0	0	0
yndD	KFAV_v1_0305	Uncharacterized membrane protein YndD	3	0	0	0
prkA_3	KFAV_v1_2721	serine protein kinase (involved in sporulation)	3	0	0	0
KFAV_v1_1527	KFAV_v1_1527	conserved protein of unknown function	2	0	0	0
KFAV_v1_0646	KFAV_v1_0646	Putative nitric-oxide reductase	2	0	0	0
KFAV_v1_0963	KFAV_v1_0963	conserved exported protein of unknown function	2	0	0	0
KFAV_v1_0027	KFAV_v1_0027	protein of unknown function	0	0	0	0
KFAV_v1_0030	KFAV_v1_0030	Putative cytochrome c oxidase subunit IIa	0	0	0	0
KFAV_v1_0033	KFAV_v1_0033	conserved protein of unknown function	0	0	0	0
KFAV_v1_0034	KFAV_v1_0034	conserved exported protein of unknown function	0	0	0	0
sspF	KFAV_v1_0065	small acid-soluble spore protein (alpha/beta-type SASP)	0	0	0	0
cphB	KFAV_v1_0066	Cyanophycinase	0	0	0	0
KFAV_v1_0086	KFAV_v1_0086	protein of unknown function	0	0	0	0
KFAV_v1_0092	KFAV_v1_0092	protein of unknown function	0	0	0	0
KFAV_v1_0094	KFAV_v1_0094	protein of unknown function	0	0	0	0
KFAV_v1_0123	KFAV_v1_0123	conserved protein of unknown function	0	0	0	0
KFAV_v1_0125	KFAV_v1_0125	protein of unknown function	0	0	0	0
KFAV_v1_0211	KFAV_v1_0211	AzlD domain-containing protein	0	0	0	0
KFAV_v1_0215	KFAV_v1_0215	conserved membrane protein of unknown function	0	0	0	0
KFAV_v1_0216	KFAV_v1_0216	conserved protein of unknown function	0	0	0	0
KFAV_v1_0230	KFAV_v1_0230	conserved protein of unknown function	0	0	0	0
KFAV_v1_0231	KFAV_v1_0231	protein of unknown function	0	0	0	0
KFAV_v1_0245	KFAV_v1_0245	transposase (fragment)	0	0	0	0
KFAV_v1_0246	KFAV_v1_0246	protein of unknown function	0	0	0	0
KFAV_v1_0247	KFAV_v1_0247	protein of unknown function	0	0	0	0
KFAV_v1_0249	KFAV_v1_0249	Spore gernimation protein	0	0	0	0
KFAV_v1_0263	KFAV_v1_0263	protein of unknown function	0	0	0	0
KFAV_v1_0271	KFAV_v1_0271	conserved membrane protein of unknown function	0	0	0	0
chrB	KFAV_v1_0272	chromate transporter subunit N	0	0	0	0
KFAV_v1_0277	KFAV_v1_0277	2Fe-2S iron-sulfur cluster binding domain-containing protein (modular protein)	0	0	0	0
KFAV_v1_0286	KFAV_v1_0286	conserved protein of unknown function	0	0	0	0
KFAV_v1_0294	KFAV_v1_0294	Transcriptional regulator, PadR-like family	0	0	0	0
KFAV_v1_0295	KFAV_v1_0295	protein of unknown function	0	0	0	0
KFAV_v1_0298	KFAV_v1_0298	Cytosine permease	0	0	0	0
KFAV_v1_0304	KFAV_v1_0304	conserved membrane protein of unknown function	0	0	0	0
KFAV_v1_0307	KFAV_v1_0307	Ger(X)C family spore germination protein	0	0	0	0

KFAV_v1_0308	KFAV_v1_0308	conserved protein of unknown function	0	0	0	0
ykcB_1	KFAV_v1_0324	fragment of putative membrane glycosyl transferase (part 2)	0	0	0	0
KFAV_v1_0325	KFAV_v1_0325	protein of unknown function	0	0	0	0
KFAV_v1_0326	KFAV_v1_0326	transposase (fragment)	0	0	0	0
KFAV_v1_0329	KFAV_v1_0329	conserved protein of unknown function	0	0	0	0
KFAV_v1_0338	KFAV_v1_0338	conserved protein of unknown function	0	0	0	0
KFAV_v1_0339	KFAV_v1_0339	protein of unknown function	0	0	0	0
KFAV_v1_0352	KFAV_v1_0352	protein of unknown function	0	0	0	0
KFAV_v1_0358	KFAV_v1_0358	protein of unknown function	0	0	0	0
KFAV_v1_0362	KFAV_v1_0362	conserved protein of unknown function	0	0	0	0
KFAV_v1_0363	KFAV_v1_0363	transposase (fragment)	0	0	0	0
KFAV_v1_0364	KFAV_v1_0364	exported protein of unknown function	0	0	0	0
pxpB_1	KFAV_v1_0368	L-5-oxoprolinase (ATP-dependent) subunit B	0	0	0	0
KFAV_v1_0369	KFAV_v1_0369	KipI antagonist	0	0	0	0
KFAV_v1_0374	KFAV_v1_0374	protein of unknown function	0	0	0	0
KFAV_v1_0375	KFAV_v1_0375	protein of unknown function	0	0	0	0
KFAV_v1_0383	KFAV_v1_0383	protein of unknown function	0	0	0	0
KFAV_v1_0387	KFAV_v1_0387	protein of unknown function	0	0	0	0
KFAV_v1_0388	KFAV_v1_0388	transposase (fragment)	0	0	0	0
KFAV_v1_0395	KFAV_v1_0395	protein of unknown function	0	0	0	0
KFAV_v1_0399	KFAV_v1_0399	protein of unknown function	0	0	0	0
KFAV_v1_0403	KFAV_v1_0403	conserved protein of unknown function	0	0	0	0
KFAV_v1_0407	KFAV_v1_0407	protein of unknown function	0	0	0	0
KFAV_v1_0411	KFAV_v1_0411	conserved protein of unknown function	0	0	0	0
KFAV_v1_0412	KFAV_v1_0412	conserved protein of unknown function	0	0	0	0
KFAV_v1_0413	KFAV_v1_0413	putative glycosyltransferase	0	0	0	0
KFAV_v1_0417	KFAV_v1_0417	conserved protein of unknown function	0	0	0	0
KFAV_v1_0426	KFAV_v1_0426	transposase (fragment)	0	0	0	0
KFAV_v1_0427	KFAV_v1_0427	transposase (fragment)	0	0	0	0
KFAV_v1_0437	KFAV_v1_0437	exported protein of unknown function	0	0	0	0
KFAV_v1_0438	KFAV_v1_0438	conserved protein of unknown function	0	0	0	0
KFAV_v1_0439	KFAV_v1_0439	protein of unknown function	0	0	0	0
KFAV_v1_0440	KFAV_v1_0440	protein of unknown function	0	0	0	0
KFAV_v1_0452	KFAV_v1_0452	protein of unknown function	0	0	0	0
KFAV_v1_0459	KFAV_v1_0459	protein of unknown function	0	0	0	0
yycR	KFAV_v1_0462	putative dehydrogenase	0	0	0	0
KFAV_v1_0464	KFAV_v1_0464	protein of unknown function	0	0	0	0
KFAV_v1_0466	KFAV_v1_0466	protein of unknown function	0	0	0	0
KFAV_v1_0467	KFAV_v1_0467	protein of unknown function	0	0	0	0
KFAV_v1_0494	KFAV_v1_0494	conserved protein of unknown function	0	0	0	0
KFAV_v1_0499	KFAV_v1_0499	Cation transporter (fragment)	0	0	0	0
KFAV_v1_0502	KFAV_v1_0502	Resolvase domain protein (fragment)	0	0	0	0
KFAV_v1_0503	KFAV_v1_0503	conserved protein of unknown function	0	0	0	0
KFAV_v1_0506	KFAV_v1_0506	conserved protein of unknown function	0	0	0	0
ytlC	KFAV_v1_0508	putative ABC anion transporter component, ATP-binding	0	0	0	0
KFAV_v1_0509	KFAV_v1_0509	Sulfonate ABC transporter permease	0	0	0	0
KFAV_v1_0511	KFAV_v1_0511	conserved protein of unknown function	0	0	0	0
KFAV_v1_0512	KFAV_v1_0512	Group II intron reverse transcriptase/maturase (fragment)	0	0	0	0
KFAV_v1_0513	KFAV_v1_0513	protein of unknown function	0	0	0	0
KFAV_v1_0514	KFAV_v1_0514	Group II intron reverse transcriptase/maturase (fragment)	0	0	0	0
KFAV_v1_0515	KFAV_v1_0515	conserved protein of unknown function	0	0	0	0
KFAV_v1_0516	KFAV_v1_0516	protein of unknown function	0	0	0	0

KFAV_v1_0518	KFAV_v1_0518	protein of unknown function	0	0	0	0
KFAV_v1_0519	KFAV_v1_0519	protein of unknown function	0	0	0	0
ltrA_2	KFAV_v1_0520	Group II intron reverse transcriptase/maturase	0	0	0	0
KFAV_v1_0521	KFAV_v1_0521	protein of unknown function	0	0	0	0
KFAV_v1_0522	KFAV_v1_0522	protein of unknown function	0	0	0	0
KFAV_v1_0527	KFAV_v1_0527	protein of unknown function	0	0	0	0
KFAV_v1_0539	KFAV_v1_0539	protein of unknown function	0	0	0	0
KFAV_v1_0545	KFAV_v1_0545	conserved protein of unknown function	0	0	0	0
KFAV_v1_0569	KFAV_v1_0569	conserved protein of unknown function	0	0	0	0
KFAV_v1_0578	KFAV_v1_0578	protein of unknown function	0	0	0	0
KFAV_v1_0579	KFAV_v1_0579	conserved protein of unknown function	0	0	0	0
KFAV_v1_0582	KFAV_v1_0582	protein of unknown function	0	0	0	0
KFAV_v1_0600	KFAV_v1_0600	protein of unknown function	0	0	0	0
KFAV_v1_0602	KFAV_v1_0602	conserved protein of unknown function	0	0	0	0
KFAV_v1_0618	KFAV_v1_0618	Enoyl-CoA hydratase	0	0	0	0
KFAV_v1_0621	KFAV_v1_0621	conserved protein of unknown function	0	0	0	0
KFAV_v1_0629	KFAV_v1_0629	protein of unknown function	0	0	0	0
KFAV_v1_0630	KFAV_v1_0630	protein of unknown function	0	0	0	0
KFAV_v1_0633	KFAV_v1_0633	protein of unknown function	0	0	0	0
KFAV_v1_0635	KFAV_v1_0635	Transcriptional regulator, PadR-like family	0	0	0	0
KFAV_v1_0637	KFAV_v1_0637	conserved protein of unknown function	0	0	0	0
KFAV_v1_0639	KFAV_v1_0639	conserved protein of unknown function	0	0	0	0
KFAV_v1_0640	KFAV_v1_0640	Small, acid-soluble spore protein 1 (fragment)	0	0	0	0
KFAV_v1_0642	KFAV_v1_0642	Spore germination protein	0	0	0	0
KFAV_v1_0643	KFAV_v1_0643	protein of unknown function	0	0	0	0
KFAV_v1_0644	KFAV_v1_0644	protein of unknown function	0	0	0	0
KFAV_v1_0645	KFAV_v1_0645	conserved protein of unknown function	0	0	0	0
KFAV_v1_0647	KFAV_v1_0647	RNA polymerase, sigma-24 subunit, ECF subfamily	0	0	0	0
KFAV_v1_0655	KFAV_v1_0655	protein of unknown function	0	0	0	0
KFAV_v1_0667	KFAV_v1_0667	protein of unknown function	0	0	0	0
KFAV_v1_0670	KFAV_v1_0670	ABC transporter substrate-binding protein	0	0	0	0
KFAV_v1_0683	KFAV_v1_0683	protein of unknown function	0	0	0	0
KFAV_v1_0684	KFAV_v1_0684	protein of unknown function	0	0	0	0
KFAV_v1_0686	KFAV_v1_0686	protein of unknown function	0	0	0	0
KFAV_v1_0693	KFAV_v1_0693	transposase (fragment)	0	0	0	0
KFAV_v1_0694	KFAV_v1_0694	protein of unknown function	0	0	0	0
KFAV_v1_0695	KFAV_v1_0695	transposase (fragment)	0	0	0	0
KFAV_v1_0698	KFAV_v1_0698	Sodium:proton exchanger	0	0	0	0
KFAV_v1_0722	KFAV_v1_0722	protein of unknown function	0	0	0	0
KFAV_v1_0735	KFAV_v1_0735	protein of unknown function	0	0	0	0
KFAV_v1_0763	KFAV_v1_0763	conserved protein of unknown function	0	0	0	0
livG_1	KFAV_v1_0780	leucine/isoleucine/valine transporter subunit ; ATP-binding component of ABC superfamily	0	0	0	0
KFAV_v1_0782	KFAV_v1_0782	protein of unknown function	0	0	0	0
KFAV_v1_0790	KFAV_v1_0790	conserved protein of unknown function	0	0	0	0
KFAV_v1_0809	KFAV_v1_0809	protein of unknown function	0	0	0	0
KFAV_v1_0811	KFAV_v1_0811	conserved protein of unknown function	0	0	0	0
yisU	KFAV_v1_0814	putative amino-acid transporter YisU	0	0	0	0
KFAV_v1_0819	KFAV_v1_0819	protein of unknown function	0	0	0	0
KFAV_v1_0826	KFAV_v1_0826	conserved protein of unknown function	0	0	0	0
cbiC	KFAV_v1_0829	Cobalt-precorrin-8 methylmutase	0	0	0	0
KFAV_v1_0838	KFAV_v1_0838	Nicotinate-nucleotide--dimethylbenzimidazole phosphoribosyltransferase (fragment)	0	0	0	0
KFAV_v1_0839	KFAV_v1_0839	Nicotinate-nucleotide--dimethylbenzimidazole phosphoribosyltransferase (fragment)	0	0	0	0

KFAV_v1_0844	KFAV_v1_0844	protein of unknown function	0	0	0	0
KFAV_v1_0845	KFAV_v1_0845	Adenosylcobinamide-GDP ribazoletransferase (fragment)	0	0	0	0
KFAV_v1_0846	KFAV_v1_0846	Adenosylcobinamide-GDP ribazoletransferase (fragment)	0	0	0	0
KFAV_v1_0853	KFAV_v1_0853	Putative cytochrome c oxidase subunit IIa	0	0	0	0
KFAV_v1_0874	KFAV_v1_0874	Hemerythrin	0	0	0	0
KFAV_v1_0882	KFAV_v1_0882	protein of unknown function	0	0	0	0
KFAV_v1_0883	KFAV_v1_0883	factor involved in shape determination (fragment)	0	0	0	0
KFAV_v1_0900	KFAV_v1_0900	YdcF family protein	0	0	0	0
KFAV_v1_0903	KFAV_v1_0903	Spore germination protein	0	0	0	0
KFAV_v1_0904	KFAV_v1_0904	conserved membrane protein of unknown function	0	0	0	0
KFAV_v1_0905	KFAV_v1_0905	protein of unknown function	0	0	0	0
KFAV_v1_0906	KFAV_v1_0906	conserved protein of unknown function	0	0	0	0
KFAV_v1_0907	KFAV_v1_0907	conserved membrane protein of unknown function	0	0	0	0
KFAV_v1_0921	KFAV_v1_0921	protein of unknown function	0	0	0	0
KFAV_v1_0922	KFAV_v1_0922	protein of unknown function	0	0	0	0
KFAV_v1_0927	KFAV_v1_0927	conserved protein of unknown function	0	0	0	0
KFAV_v1_0942	KFAV_v1_0942	conserved protein of unknown function	0	0	0	0
arsF_3	KFAV_v1_0949	fragment of arsenite/antimonite/H+ antiporter (part 1)	0	0	0	0
KFAV_v1_0952	KFAV_v1_0952	transposase (fragment)	0	0	0	0
KFAV_v1_0953	KFAV_v1_0953	conserved protein of unknown function	0	0	0	0
KFAV_v1_0954	KFAV_v1_0954	Integrase catalytic region (fragment)	0	0	0	0
KFAV_v1_0956	KFAV_v1_0956	AAA family ATPase	0	0	0	0
KFAV_v1_0957	KFAV_v1_0957	conserved protein of unknown function	0	0	0	0
KFAV_v1_0960	KFAV_v1_0960	conserved exported protein of unknown function	0	0	0	0
bluB	KFAV_v1_0968	5,6-dimethylbenzimidazole synthase	0	0	0	0
KFAV_v1_0973	KFAV_v1_0973	protein of unknown function	0	0	0	0
KFAV_v1_0974	KFAV_v1_0974	protein of unknown function	0	0	0	0
KFAV_v1_0975	KFAV_v1_0975	protein of unknown function	0	0	0	0
KFAV_v1_0976	KFAV_v1_0976	protein of unknown function	0	0	0	0
KFAV_v1_0987	KFAV_v1_0987	BFD-like [2Fe-2S] binding domain-containing protein	0	0	0	0
KFAV_v1_0992	KFAV_v1_0992	protein of unknown function	0	0	0	0
KFAV_v1_0995	KFAV_v1_0995	Lantibiotic immunity ABC transporter MutG family permease subunit	0	0	0	0
KFAV_v1_1001	KFAV_v1_1001	protein of unknown function	0	0	0	0
KFAV_v1_1003	KFAV_v1_1003	protein of unknown function	0	0	0	0
hcnA	KFAV_v1_1010	Hydrogen cyanide synthase subunit HcnA	0	0	0	0
KFAV_v1_1023	KFAV_v1_1023	protein of unknown function	0	0	0	0
cutI	KFAV_v1_1025	putative membrane protein involved in copper intake	0	0	0	0
cydA_2	KFAV_v1_1027	cytochrome bb' ubiquinol oxidase (subunit I)	0	0	0	0
KFAV_v1_1066	KFAV_v1_1066	conserved protein of unknown function	0	0	0	0
patA_1	KFAV_v1_1067	Putrescine aminotransferase	0	0	0	0
KFAV_v1_1068	KFAV_v1_1068	conserved protein of unknown function	0	0	0	0
KFAV_v1_1069	KFAV_v1_1069	protein of unknown function	0	0	0	0
KFAV_v1_1072	KFAV_v1_1072	conserved protein of unknown function	0	0	0	0
KFAV_v1_1086	KFAV_v1_1086	Integrase catalytic region (fragment)	0	0	0	0
KFAV_v1_1100	KFAV_v1_1100	conserved protein of unknown function	0	0	0	0
KFAV_v1_1107	KFAV_v1_1107	conserved membrane protein of unknown function	0	0	0	0
KFAV_v1_1142	KFAV_v1_1142	conserved protein of unknown function	0	0	0	0
KFAV_v1_1158	KFAV_v1_1158	protein of unknown function	0	0	0	0
KFAV_v1_1170	KFAV_v1_1170	protein of unknown function	0	0	0	0
KFAV_v1_1186	KFAV_v1_1186	protein of unknown function	0	0	0	0
spoIIIAA	KFAV_v1_1204	ATP-binding stage III sporulation protein	0	0	0	0
spoIIIAB	KFAV_v1_1205	Stage III sporulation protein AB	0	0	0	0

spoIIIAC	KFAV_v1_1206	stage III sporulation protein (feeding tube apparatus)	0	0	0	0
spoIIIAD	KFAV_v1_1207	stage III sporulation protein (feeding tube apparatus)	0	0	0	0
KFAV_v1_1223	KFAV_v1_1223	protein of unknown function	0	0	0	0
KFAV_v1_1250	KFAV_v1_1250	protein of unknown function	0	0	0	0
KFAV_v1_1262	KFAV_v1_1262	protein of unknown function	0	0	0	0
KFAV_v1_1268	KFAV_v1_1268	protein of unknown function	0	0	0	0
KFAV_v1_1277	KFAV_v1_1277	protein of unknown function	0	0	0	0
KFAV_v1_1279	KFAV_v1_1279	protein of unknown function	0	0	0	0
KFAV_v1_1280	KFAV_v1_1280	conserved protein of unknown function	0	0	0	0
KFAV_v1_1281	KFAV_v1_1281	protein of unknown function	0	0	0	0
KFAV_v1_1283	KFAV_v1_1283	protein of unknown function	0	0	0	0
KFAV_v1_1287	KFAV_v1_1287	transposase (fragment)	0	0	0	0
KFAV_v1_1291	KFAV_v1_1291	protein of unknown function	0	0	0	0
KFAV_v1_1296	KFAV_v1_1296	protein of unknown function	0	0	0	0
KFAV_v1_1297	KFAV_v1_1297	protein of unknown function	0	0	0	0
KFAV_v1_1298	KFAV_v1_1298	transposase (fragment)	0	0	0	0
KFAV_v1_1299	KFAV_v1_1299	transposase (fragment)	0	0	0	0
KFAV_v1_1300	KFAV_v1_1300	conserved protein of unknown function	0	0	0	0
KFAV_v1_1303	KFAV_v1_1303	transposase	0	0	0	0
KFAV_v1_1304	KFAV_v1_1304	transposase (fragment)	0	0	0	0
KFAV_v1_1311	KFAV_v1_1311	protein of unknown function	0	0	0	0
adh_3	KFAV_v1_1314	Alcohol dehydrogenase	0	0	0	0
amaB_1	KFAV_v1_1334	N-carbamoyl-L-amino acid hydrolase	0	0	0	0
pucL_1	KFAV_v1_1336	fragment of urate oxidase with peroxide reductase N-terminal domain (part 1)	0	0	0	0
pucM	KFAV_v1_1338	5-hydroxyisourate hydrolase; also uricase subunit	0	0	0	0
KFAV_v1_1340	KFAV_v1_1340	Purine-binding protein BAB2_0673	0	0	0	0
KFAV_v1_1343	KFAV_v1_1343	Inner-membrane translocator	0	0	0	0
KFAV_v1_1344	KFAV_v1_1344	Molybdopterin dehydrogenase FAD-binding protein	0	0	0	0
pucD_1	KFAV_v1_1345	fragment of xanthine dehydrogenase, substrate and molybdenum cofactor subunit (part 1)	0	0	0	0
pucD_2	KFAV_v1_1346	fragment of xanthine dehydrogenase, substrate and molybdenum cofactor subunit (part 2)	0	0	0	0
pucE	KFAV_v1_1347	xanthine dehydrogenase, iron-sulfur subunit	0	0	0	0
KFAV_v1_1348	KFAV_v1_1348	Adenosine deaminase	0	0	0	0
KFAV_v1_1349	KFAV_v1_1349	8-oxoguanine deaminase	0	0	0	0
KFAV_v1_1354	KFAV_v1_1354	UbiA prenyltransferase	0	0	0	0
KFAV_v1_1355	KFAV_v1_1355	conserved membrane protein of unknown function	0	0	0	0
KFAV_v1_1356	KFAV_v1_1356	protein of unknown function	0	0	0	0
KFAV_v1_1376	KFAV_v1_1376	Aspartate aminotransferase family protein	0	0	0	0
KFAV_v1_1379	KFAV_v1_1379	Holin-like protein CidA (modular protein)	0	0	0	0
cidB	KFAV_v1_1380	metabolic regulator controlling activity of murein hydrolases	0	0	0	0
arsC	KFAV_v1_1383	thioredoxin-coupled arsenate reductase; skin element	0	0	0	0
KFAV_v1_1390	KFAV_v1_1390	conserved protein of unknown function	0	0	0	0
KFAV_v1_1403	KFAV_v1_1403	protein of unknown function	0	0	0	0
KFAV_v1_1405	KFAV_v1_1405	protein of unknown function	0	0	0	0
KFAV_v1_1407	KFAV_v1_1407	conserved protein of unknown function	0	0	0	0
KFAV_v1_1417	KFAV_v1_1417	Polysaccharide deacetylase family protein	0	0	0	0
KFAV_v1_1418	KFAV_v1_1418	protein of unknown function	0	0	0	0
KFAV_v1_1444	KFAV_v1_1444	conserved exported protein of unknown function	0	0	0	0
KFAV_v1_1445	KFAV_v1_1445	protein of unknown function	0	0	0	0
cotJC	KFAV_v1_1450	enzyme component of the inner spore coat	0	0	0	0
cotJB	KFAV_v1_1451	component of the inner spore coat	0	0	0	0
cotJA	KFAV_v1_1452	component of the inner spore coat	0	0	0	0
KFAV_v1_1456	KFAV_v1_1456	conserved protein of unknown function	0	0	0	0

spoVAC_1	KFAV_v1_1457	mechanosensitive channel; stage V sporulation protein AC	0	0	0	0
spoVAD_1	KFAV_v1_1458	stage V sporulation protein AD (uptake of pyridine-2,6-dicarboxylic acid)	0	0	0	0
KFAV_v1_1459	KFAV_v1_1459	protein of unknown function	0	0	0	0
KFAV_v1_1465	KFAV_v1_1465	protein of unknown function	0	0	0	0
KFAV_v1_1468	KFAV_v1_1468	protein of unknown function	0	0	0	0
KFAV_v1_1469	KFAV_v1_1469	putative Phage transcriptional regulator, RinA family protein	0	0	0	0
KFAV_v1_1470	KFAV_v1_1470	conserved protein of unknown function	0	0	0	0
KFAV_v1_1471	KFAV_v1_1471	conserved protein of unknown function	0	0	0	0
KFAV_v1_1472	KFAV_v1_1472	conserved protein of unknown function	0	0	0	0
KFAV_v1_1473	KFAV_v1_1473	conserved protein of unknown function	0	0	0	0
KFAV_v1_1474	KFAV_v1_1474	DNA packaging protein	0	0	0	0
KFAV_v1_1475	KFAV_v1_1475	Phage major capsid protein, HK97 family	0	0	0	0
KFAV_v1_1478	KFAV_v1_1478	exported protein of unknown function	0	0	0	0
KFAV_v1_1479	KFAV_v1_1479	conserved protein of unknown function	0	0	0	0
KFAV_v1_1480	KFAV_v1_1480	Phage/plasmid primase, P4 family	0	0	0	0
KFAV_v1_1482	KFAV_v1_1482	protein of unknown function	0	0	0	0
KFAV_v1_1483	KFAV_v1_1483	Transcriptional regulator	0	0	0	0
KFAV_v1_1489	KFAV_v1_1489	XRE family transcriptional regulator	0	0	0	0
KFAV_v1_1494	KFAV_v1_1494	protein of unknown function	0	0	0	0
KFAV_v1_1495	KFAV_v1_1495	Integrase family protein	0	0	0	0
KFAV_v1_1496	KFAV_v1_1496	protein of unknown function	0	0	0	0
KFAV_v1_1497	KFAV_v1_1497	conserved exported protein of unknown function	0	0	0	0
KFAV_v1_1498	KFAV_v1_1498	protein of unknown function	0	0	0	0
KFAV_v1_1513	KFAV_v1_1513	protein of unknown function	0	0	0	0
KFAV_v1_1517	KFAV_v1_1517	conserved protein of unknown function	0	0	0	0
KFAV_v1_1525	KFAV_v1_1525	protein of unknown function	0	0	0	0
KFAV_v1_1526	KFAV_v1_1526	exported protein of unknown function	0	0	0	0
KFAV_v1_1535	KFAV_v1_1535	protein of unknown function	0	0	0	0
KFAV_v1_1539	KFAV_v1_1539	protein of unknown function	0	0	0	0
KFAV_v1_1561	KFAV_v1_1561	YlmC/YmxH family sporulation protein	0	0	0	0
spoVAA	KFAV_v1_1575	stage V sporulation protein AA	0	0	0	0
spoVAC_2	KFAV_v1_1577	mechanosensitive channel; stage V sporulation protein AC	0	0	0	0
spoVAD_2	KFAV_v1_1578	stage V sporulation protein AD (uptake of pyridine-2,6-dicarboxylic acid)	0	0	0	0
spoVAEB	KFAV_v1_1579	spore germinant protein	0	0	0	0
KFAV_v1_1593	KFAV_v1_1593	D-alanyl-D-alanine carboxypeptidase	0	0	0	0
spmA	KFAV_v1_1594	spore maturation protein	0	0	0	0
KFAV_v1_1618	KFAV_v1_1618	conserved protein of unknown function	0	0	0	0
KFAV_v1_1619	KFAV_v1_1619	Peptidase A2	0	0	0	0
comA	KFAV_v1_1632	Phosphosulfolactate synthase	0	0	0	0
yitG	KFAV_v1_1633	putative efflux transporter	0	0	0	0
KFAV_v1_1634	KFAV_v1_1634	protein of unknown function	0	0	0	0
sleB_2	KFAV_v1_1637	spore germination cortex-lytic enzyme	0	0	0	0
KFAV_v1_1639	KFAV_v1_1639	N-acetylmuramoyl-L-alanine amidase	0	0	0	0
KFAV_v1_1640	KFAV_v1_1640	protein of unknown function	0	0	0	0
KFAV_v1_1641	KFAV_v1_1641	conserved protein of unknown function	0	0	0	0
KFAV_v1_1647	KFAV_v1_1647	conserved protein of unknown function	0	0	0	0
KFAV_v1_1675	KFAV_v1_1675	protein of unknown function	0	0	0	0
KFAV_v1_1704	KFAV_v1_1704	conserved protein of unknown function	0	0	0	0
ypjD	KFAV_v1_1715	oxidized nucleotide pyrophosphohydrolase	0	0	0	0
nifB	KFAV_v1_1742	FeMo cofactor biosynthesis protein NifB	0	0	0	0
nifV	KFAV_v1_1743	Homocitrate synthase 1	0	0	0	0
KFAV_v1_1744	KFAV_v1_1744	Cysteine desulfurase NifS	0	0	0	0

KFAV_v1_1745	KFAV_v1_1745	conserved protein of unknown function	0	0	0	0
nifH	KFAV_v1_1746	Nitrogenase iron protein	0	0	0	0
nifD	KFAV_v1_1747	Nitrogenase molybdenum-iron protein alpha chain	0	0	0	0
nifK	KFAV_v1_1748	Nitrogenase molybdenum-iron protein beta chain	0	0	0	0
nifE	KFAV_v1_1749	Nitrogenase iron-molybdenum cofactor biosynthesis protein	0	0	0	0
nifN	KFAV_v1_1750	Putative nitrogenase iron-molybdenum cofactor biosynthesis protein	0	0	0	0
nifX	KFAV_v1_1751	Protein NifX	0	0	0	0
KFAV_v1_1752	KFAV_v1_1752	Resolvase domain protein (fragment)	0	0	0	0
KFAV_v1_1753	KFAV_v1_1753	protein of unknown function	0	0	0	0
KFAV_v1_1792	KFAV_v1_1792	conserved protein of unknown function	0	0	0	0
KFAV_v1_1793	KFAV_v1_1793	protein of unknown function	0	0	0	0
KFAV_v1_1795	KFAV_v1_1795	conserved protein of unknown function	0	0	0	0
KFAV_v1_1800	KFAV_v1_1800	conserved protein of unknown function	0	0	0	0
KFAV_v1_1820	KFAV_v1_1820	conserved protein of unknown function	0	0	0	0
KFAV_v1_1859	KFAV_v1_1859	protein of unknown function	0	0	0	0
KFAV_v1_1886	KFAV_v1_1886	conserved protein of unknown function	0	0	0	0
fliQ	KFAV_v1_1898	component of the flagellar export machinery	0	0	0	0
KFAV_v1_1925	KFAV_v1_1925	protein of unknown function	0	0	0	0
KFAV_v1_1931	KFAV_v1_1931	protein of unknown function	0	0	0	0
KFAV_v1_1945	KFAV_v1_1945	conserved protein of unknown function	0	0	0	0
KFAV_v1_1951	KFAV_v1_1951	protein of unknown function	0	0	0	0
KFAV_v1_1953	KFAV_v1_1953	protein of unknown function	0	0	0	0
KFAV_v1_2017	KFAV_v1_2017	protein of unknown function	0	0	0	0
KFAV_v1_2023	KFAV_v1_2023	protein of unknown function	0	0	0	0
KFAV_v1_2026	KFAV_v1_2026	ABC transporter permease (fragment)	0	0	0	0
KFAV_v1_2032	KFAV_v1_2032	conserved protein of unknown function	0	0	0	0
KFAV_v1_2033	KFAV_v1_2033	Cytosine permease (fragment)	0	0	0	0
KFAV_v1_2034	KFAV_v1_2034	protein of unknown function	0	0	0	0
KFAV_v1_2035	KFAV_v1_2035	conserved protein of unknown function	0	0	0	0
KFAV_v1_2043	KFAV_v1_2043	protein of unknown function	0	0	0	0
KFAV_v1_2057	KFAV_v1_2057	protein of unknown function	0	0	0	0
KFAV_v1_2063	KFAV_v1_2063	conserved protein of unknown function	0	0	0	0
KFAV_v1_2065	KFAV_v1_2065	protein of unknown function	0	0	0	0
KFAV_v1_2071	KFAV_v1_2071	conserved protein of unknown function	0	0	0	0
KFAV_v1_2096	KFAV_v1_2096	protein of unknown function	0	0	0	0
KFAV_v1_2099	KFAV_v1_2099	Endonuclease, Uma2 family (Restriction endonuclease fold) (fragment)	0	0	0	0
KFAV_v1_2109	KFAV_v1_2109	protein of unknown function	0	0	0	0
KFAV_v1_2129	KFAV_v1_2129	putative esterase AF_2264	0	0	0	0
KFAV_v1_2131	KFAV_v1_2131	Ethyl tert-butyl ether degradation EthD	0	0	0	0
KFAV_v1_2148	KFAV_v1_2148	conserved protein of unknown function	0	0	0	0
KFAV_v1_2152	KFAV_v1_2152	protein of unknown function	0	0	0	0
KFAV_v1_2170	KFAV_v1_2170	protein of unknown function	0	0	0	0
KFAV_v1_2171	KFAV_v1_2171	protein of unknown function	0	0	0	0
KFAV_v1_2172	KFAV_v1_2172	conserved protein of unknown function	0	0	0	0
KFAV_v1_2182	KFAV_v1_2182	protein of unknown function	0	0	0	0
KFAV_v1_2183	KFAV_v1_2183	conserved protein of unknown function	0	0	0	0
KFAV_v1_2184	KFAV_v1_2184	conserved protein of unknown function	0	0	0	0
KFAV_v1_2185	KFAV_v1_2185	protein of unknown function	0	0	0	0
KFAV_v1_2186	KFAV_v1_2186	Nucleotide sugar dehydrogenase	0	0	0	0
KFAV_v1_2188	KFAV_v1_2188	Nucleoside-diphosphate-sugar pyrophosphorylase	0	0	0	0
KFAV_v1_2190	KFAV_v1_2190	Glycosyl transferase family 2	0	0	0	0
KFAV_v1_2191	KFAV_v1_2191	Group 1 glycosyl transferase	0	0	0	0

KFAV_v1_2194	KFAV_v1_2194	protein of unknown function	0	0	0	0
KFAV_v1_2195	KFAV_v1_2195	conserved protein of unknown function	0	0	0	0
KFAV_v1_2198	KFAV_v1_2198	conserved protein of unknown function	0	0	0	0
KFAV_v1_2199	KFAV_v1_2199	protein of unknown function	0	0	0	0
KFAV_v1_2200	KFAV_v1_2200	conserved protein of unknown function	0	0	0	0
KFAV_v1_2201	KFAV_v1_2201	conserved protein of unknown function	0	0	0	0
KFAV_v1_2204	KFAV_v1_2204	Glycosyl transferase family 1	0	0	0	0
KFAV_v1_2205	KFAV_v1_2205	Glycosyl transferase family 2	0	0	0	0
KFAV_v1_2206	KFAV_v1_2206	conserved protein of unknown function	0	0	0	0
KFAV_v1_2224	KFAV_v1_2224	conserved protein of unknown function	0	0	0	0
acyP	KFAV_v1_2241	Acylphosphatase	0	0	0	0
KFAV_v1_2247	KFAV_v1_2247	Transcriptional regulator, MerR family	0	0	0	0
KFAV_v1_2250	KFAV_v1_2250	conserved protein of unknown function	0	0	0	0
KFAV_v1_2265	KFAV_v1_2265	conserved protein of unknown function	0	0	0	0
KFAV_v1_2269	KFAV_v1_2269	protein of unknown function	0	0	0	0
nahT_1	KFAV_v1_2270	Ferredoxin, plant-type	0	0	0	0
KFAV_v1_2279	KFAV_v1_2279	protein of unknown function	0	0	0	0
KFAV_v1_2281	KFAV_v1_2281	conserved protein of unknown function	0	0	0	0
KFAV_v1_2286	KFAV_v1_2286	conserved protein of unknown function	0	0	0	0
KFAV_v1_2297	KFAV_v1_2297	protein of unknown function	0	0	0	0
KFAV_v1_2316	KFAV_v1_2316	conserved protein of unknown function	0	0	0	0
KFAV_v1_2317	KFAV_v1_2317	exported protein of unknown function	0	0	0	0
KFAV_v1_2328	KFAV_v1_2328	Dihydroneopterin triphosphate diphosphatase (fragment)	0	0	0	0
KFAV_v1_2345	KFAV_v1_2345	conserved membrane protein of unknown function	0	0	0	0
KFAV_v1_2346	KFAV_v1_2346	conserved protein of unknown function	0	0	0	0
KFAV_v1_2363	KFAV_v1_2363	protein of unknown function	0	0	0	0
KFAV_v1_2371	KFAV_v1_2371	conserved protein of unknown function	0	0	0	0
KFAV_v1_2377	KFAV_v1_2377	conserved protein of unknown function	0	0	0	0
KFAV_v1_2378	KFAV_v1_2378	protein of unknown function	0	0	0	0
KFAV_v1_2380	KFAV_v1_2380	conserved protein of unknown function	0	0	0	0
KFAV_v1_2389	KFAV_v1_2389	conserved membrane protein of unknown function	0	0	0	0
KFAV_v1_2397	KFAV_v1_2397	conserved protein of unknown function	0	0	0	0
KFAV_v1_2425	KFAV_v1_2425	protein of unknown function	0	0	0	0
KFAV_v1_2427	KFAV_v1_2427	protein of unknown function	0	0	0	0
KFAV_v1_2429	KFAV_v1_2429	conserved protein of unknown function	0	0	0	0
KFAV_v1_2430	KFAV_v1_2430	conserved protein of unknown function	0	0	0	0
KFAV_v1_2431	KFAV_v1_2431	Luciferase-like monooxygenase (fragment)	0	0	0	0
KFAV_v1_2449	KFAV_v1_2449	protein of unknown function	0	0	0	0
KFAV_v1_2450	KFAV_v1_2450	protein of unknown function	0	0	0	0
KFAV_v1_2456	KFAV_v1_2456	Monooxygenase component MmoB/DmpM	0	0	0	0
nahT_2	KFAV_v1_2458	Ferredoxin, plant-type	0	0	0	0
tmoB	KFAV_v1_2462	Toluene-4-monooxygenase system, hydroxylase component subunit gamma	0	0	0	0
KFAV_v1_2463	KFAV_v1_2463	Rieske (2Fe-2S) region	0	0	0	0
tmoD	KFAV_v1_2464	Toluene-4-monooxygenase system, effector component	0	0	0	0
KFAV_v1_2470	KFAV_v1_2470	protein of unknown function	0	0	0	0
yhcA_2	KFAV_v1_2473	fragment of putative exporter (benzoate transcriptome) (part 2)	0	0	0	0
yhcA_3	KFAV_v1_2474	fragment of putative exporter (benzoate transcriptome) (part 1)	0	0	0	0
KFAV_v1_2475	KFAV_v1_2475	protein of unknown function	0	0	0	0
yhbJ_1	KFAV_v1_2476	putative membrane fusion protein; putative exporter subunit (benzoate transcriptome)	0	0	0	0
ltrA_5	KFAV_v1_2477	Group II intron reverse transcriptase/maturase	0	0	0	0
KFAV_v1_2478	KFAV_v1_2478	conserved protein of unknown function	0	0	0	0
KFAV_v1_2480	KFAV_v1_2480	protein of unknown function	0	0	0	0

KFAV_v1_2483	KFAV_v1_2483	transposase	0	0	0	0
KFAV_v1_2503	KFAV_v1_2503	conserved protein of unknown function	0	0	0	0
KFAV_v1_2511	KFAV_v1_2511	Long-chain-fatty-acid--CoA ligase (fragment)	0	0	0	0
yunB	KFAV_v1_2513	Sporulation protein YunB	0	0	0	0
KFAV_v1_2543	KFAV_v1_2543	protein of unknown function	0	0	0	0
KFAV_v1_2549	KFAV_v1_2549	protein of unknown function	0	0	0	0
KFAV_v1_2561	KFAV_v1_2561	protein of unknown function	0	0	0	0
lytH	KFAV_v1_2562	L-Ala--D-Glu endopeptidase	0	0	0	0
KFAV_v1_2566	KFAV_v1_2566	conserved membrane protein of unknown function	0	0	0	0
yhcV	KFAV_v1_2567	putative CBS containing enzyme (sporulation- / germination-related)	0	0	0	0
KFAV_v1_2568	KFAV_v1_2568	conserved protein of unknown function	0	0	0	0
KFAV_v1_2578	KFAV_v1_2578	conserved protein of unknown function	0	0	0	0
KFAV_v1_2603	KFAV_v1_2603	protein of unknown function	0	0	0	0
KFAV_v1_2608	KFAV_v1_2608	protein of unknown function	0	0	0	0
KFAV_v1_2613	KFAV_v1_2613	conserved protein of unknown function	0	0	0	0
fur_2	KFAV_v1_2616	Ferric uptake regulation protein	0	0	0	0
KFAV_v1_2645	KFAV_v1_2645	conserved protein of unknown function	0	0	0	0
KFAV_v1_2674	KFAV_v1_2674	protein of unknown function	0	0	0	0
ogt	KFAV_v1_2678	Methylated-DNA--protein-cysteine methyltransferase, constitutive	0	0	0	0
KFAV_v1_2683	KFAV_v1_2683	protein of unknown function	0	0	0	0
KFAV_v1_2687	KFAV_v1_2687	protein of unknown function	0	0	0	0
KFAV_v1_2691	KFAV_v1_2691	Bifunctional metallophosphatase/5'-nucleotidase	0	0	0	0
KFAV_v1_2692	KFAV_v1_2692	conserved exported protein of unknown function	0	0	0	0
KFAV_v1_2697	KFAV_v1_2697	Glycine cleavage system H protein (modular protein)	0	0	0	0
KFAV_v1_2698	KFAV_v1_2698	protein of unknown function	0	0	0	0
KFAV_v1_2704	KFAV_v1_2704	conserved protein of unknown function	0	0	0	0
gcvH_2	KFAV_v1_2707	Glycine cleavage system H protein 3	0	0	0	0
KFAV_v1_2713	KFAV_v1_2713	conserved protein of unknown function	0	0	0	0
KFAV_v1_2717	KFAV_v1_2717	protein of unknown function	0	0	0	0
KFAV_v1_2718	KFAV_v1_2718	protein of unknown function	0	0	0	0
spoVR_3	KFAV_v1_2719	involved in spore cortex synthesis (stage V sporulation, conserved in non sporulating bacteria	0	0	0	0
yhbH_2	KFAV_v1_2720	factor involved in shape determination	0	0	0	0
KFAV_v1_2725	KFAV_v1_2725	protein of unknown function	0	0	0	0
KFAV_v1_2750	KFAV_v1_2750	protein of unknown function	0	0	0	0
KFAV_v1_2757	KFAV_v1_2757	protein of unknown function	0	0	0	0
KFAV_v1_2760	KFAV_v1_2760	conserved protein of unknown function	0	0	0	0
KFAV_v1_2763	KFAV_v1_2763	conserved protein of unknown function	0	0	0	0
KFAV_v1_2780	KFAV_v1_2780	MarR family transcriptional regulator	0	0	0	0
KFAV_v1_2781	KFAV_v1_2781	protein of unknown function	0	0	0	0
crcBB	KFAV_v1_2782	subunit of fluoride efflux transporter	0	0	0	0
crcB	KFAV_v1_2783	putative fluoride ion transporter CrcB 2	0	0	0	0
KFAV_v1_2793	KFAV_v1_2793	conserved membrane protein of unknown function	0	0	0	0
KFAV_v1_2820	KFAV_v1_2820	protein of unknown function	0	0	0	0
KFAV_v1_2824	KFAV_v1_2824	protein of unknown function	0	0	0	0
KFAV_v1_2826	KFAV_v1_2826	protein of unknown function	0	0	0	0
KFAV_v1_2827	KFAV_v1_2827	protein of unknown function	0	0	0	0
eutC	KFAV_v1_2830	Ethanolamine ammonia-lyase light chain	0	0	0	0
KFAV_v1_2834	KFAV_v1_2834	protein of unknown function	0	0	0	0
cchA_1	KFAV_v1_2835	putative carboxysome-like ethanolaminosome structural protein, ethanolamine utilization protein	0	0	0	0
ccmL	KFAV_v1_2836	Carbon dioxide concentrating mechanism protein CcmL	0	0	0	0
KFAV_v1_2837	KFAV_v1_2837	conserved protein of unknown function	0	0	0	0
cchA_2	KFAV_v1_2838	putative carboxysome-like ethanolaminosome structural protein, ethanolamine utilization protein	0	0	0	0

KFAV_v1_2842	KFAV_v1_2842	Ethanolamine ammonia-lyase light chain (modular protein)	0	0	0	0
ypfE	KFAV_v1_2848	putative carboxysome-like ethanolaminosome structural protein with putative role in ethanol utilization	0	0	0	0
KFAV_v1_2880	KFAV_v1_2880	protein of unknown function	0	0	0	0
KFAV_v1_2884	KFAV_v1_2884	protein of unknown function	0	0	0	0
KFAV_v1_2885	KFAV_v1_2885	protein of unknown function	0	0	0	0
KFAV_v1_2905	KFAV_v1_2905	conserved protein of unknown function	0	0	0	0
KFAV_v1_2921	KFAV_v1_2921	conserved protein of unknown function	0	0	0	0
KFAV_v1_2923	KFAV_v1_2923	transposase	0	0	0	0
KFAV_v1_2927	KFAV_v1_2927	transposase (fragment)	0	0	0	0
KFAV_v1_2934	KFAV_v1_2934	protein of unknown function	0	0	0	0
KFAV_v1_2953	KFAV_v1_2953	protein of unknown function	0	0	0	0
KFAV_v1_2954	KFAV_v1_2954	protein of unknown function	0	0	0	0
KFAV_v1_2955	KFAV_v1_2955	conserved protein of unknown function	0	0	0	0
KFAV_v1_2956	KFAV_v1_2956	protein of unknown function	0	0	0	0
KFAV_v1_2957	KFAV_v1_2957	protein of unknown function	0	0	0	0
KFAV_v1_2958	KFAV_v1_2958	protein of unknown function	0	0	0	0
KFAV_v1_2959	KFAV_v1_2959	protein of unknown function	0	0	0	0
KFAV_v1_2960	KFAV_v1_2960	protein of unknown function	0	0	0	0
KFAV_v1_2961	KFAV_v1_2961	protein of unknown function	0	0	0	0
KFAV_v1_2962	KFAV_v1_2962	protein of unknown function	0	0	0	0
KFAV_v1_2974	KFAV_v1_2974	protein of unknown function	0	0	0	0
KFAV_v1_2976	KFAV_v1_2976	TetR/AcrR family transcriptional regulator	0	0	0	0
KFAV_v1_2988	KFAV_v1_2988	putative glutamine amidotransferase	0	0	0	0
KFAV_v1_2989	KFAV_v1_2989	protein of unknown function	0	0	0	0
KFAV_v1_2990	KFAV_v1_2990	Extradiol ring-cleavage dioxygenase class III	0	0	0	0
KFAV_v1_3001	KFAV_v1_3001	conserved protein of unknown function	0	0	0	0
KFAV_v1_3005	KFAV_v1_3005	protein of unknown function	0	0	0	0
KFAV_v1_3012	KFAV_v1_3012	protein of unknown function	0	0	0	0
cas1_2	KFAV_v1_3018	CRISPR-associated endonuclease Cas1 1	0	0	0	0
KFAV_v1_3020	KFAV_v1_3020	protein of unknown function	0	0	0	0
KFAV_v1_3021	KFAV_v1_3021	protein of unknown function	0	0	0	0
KFAV_v1_3022	KFAV_v1_3022	conserved protein of unknown function	0	0	0	0
KFAV_v1_3023	KFAV_v1_3023	protein of unknown function	0	0	0	0
gatA_1	KFAV_v1_3034	Glutamyl-tRNA(Gln) amidotransferase subunit A	0	0	0	0
KFAV_v1_3035	KFAV_v1_3035	protein of unknown function	0	0	0	0
appF	KFAV_v1_3037	oligopeptide ABC transporter (ATP-binding protein)	0	0	0	0
dppD	KFAV_v1_3038	dipeptide ABC transporter (ATP-binding subunit)	0	0	0	0
yliD	KFAV_v1_3039	putative peptide transporter permease subunit: membrane component of ABC superfamily	0	0	0	0
KFAV_v1_3041	KFAV_v1_3041	Peptide ABC transporter substrate-binding protein	0	0	0	0
KFAV_v1_3042	KFAV_v1_3042	protein of unknown function	0	0	0	0
KFAV_v1_3048	KFAV_v1_3048	protein of unknown function	0	0	0	0
KFAV_v1_3051	KFAV_v1_3051	protein of unknown function	0	0	0	0
KFAV_v1_3053	KFAV_v1_3053	protein of unknown function	0	0	0	0
KFAV_v1_3059	KFAV_v1_3059	protein of unknown function	0	0	0	0
KFAV_v1_3079	KFAV_v1_3079	protein of unknown function	0	0	0	0
amiS	KFAV_v1_3099	putative transporter protein AmiS2	0	0	0	0
KFAV_v1_3100	KFAV_v1_3100	protein of unknown function	0	0	0	0
KFAV_v1_3105	KFAV_v1_3105	protein of unknown function	0	0	0	0
KFAV_v1_3106	KFAV_v1_3106	protein of unknown function	0	0	0	0
pstA_2	KFAV_v1_3117	phosphate transporter subunit ; membrane component of ABC superfamily	0	0	0	0
KFAV_v1_3120	KFAV_v1_3120	Phosphate-binding protein PstS (fragment)	0	0	0	0
KFAV_v1_3147	KFAV_v1_3147	protein of unknown function	0	0	0	0

KFAV_v1_3148	KFAV_v1_3148	conserved protein of unknown function	0	0	0	0
KFAV_v1_3149	KFAV_v1_3149	conserved protein of unknown function	0	0	0	0
KFAV_v1_3150	KFAV_v1_3150	conserved protein of unknown function	0	0	0	0
KFAV_v1_3154	KFAV_v1_3154	conserved protein of unknown function	0	0	0	0
KFAV_v1_3155	KFAV_v1_3155	conserved protein of unknown function	0	0	0	0
KFAV_v1_3156	KFAV_v1_3156	XRE family transcriptional regulator (fragment)	0	0	0	0
KFAV_v1_3157	KFAV_v1_3157	conserved protein of unknown function	0	0	0	0
KFAV_v1_3158	KFAV_v1_3158	conserved protein of unknown function	0	0	0	0
KFAV_v1_3160	KFAV_v1_3160	transposase (fragment)	0	0	0	0
KFAV_v1_3161	KFAV_v1_3161	transposase (fragment)	0	0	0	0
KFAV_v1_3162	KFAV_v1_3162	conserved protein of unknown function	0	0	0	0
KFAV_v1_3166	KFAV_v1_3166	conserved membrane protein of unknown function	0	0	0	0
KFAV_v1_3167	KFAV_v1_3167	protein of unknown function	0	0	0	0
KFAV_v1_3171	KFAV_v1_3171	conserved protein of unknown function	0	0	0	0
KFAV_v1_3175	KFAV_v1_3175	MFS transporter	0	0	0	0
gltR	KFAV_v1_3176	transcriptional regulator (LysR family)	0	0	0	0
KFAV_v1_3177	KFAV_v1_3177	Antitoxin	0	0	0	0
KFAV_v1_3179	KFAV_v1_3179	transposase (fragment)	0	0	0	0
KFAV_v1_3180	KFAV_v1_3180	protein of unknown function	0	0	0	0
KFAV_v1_3181	KFAV_v1_3181	protein of unknown function	0	0	0	0
KFAV_v1_3183	KFAV_v1_3183	conserved protein of unknown function	0	0	0	0
KFAV_v1_3188	KFAV_v1_3188	protein of unknown function	0	0	0	0
KFAV_v1_3190	KFAV_v1_3190	protein of unknown function	0	0	0	0
KFAV_v1_3191	KFAV_v1_3191	transposase (fragment)	0	0	0	0
KFAV_v1_3192	KFAV_v1_3192	protein of unknown function	0	0	0	0
KFAV_v1_3193	KFAV_v1_3193	conserved protein of unknown function	0	0	0	0
KFAV_v1_3194	KFAV_v1_3194	transposase (fragment)	0	0	0	0
KFAV_v1_3198	KFAV_v1_3198	protein of unknown function	0	0	0	0
KFAV_v1_3200	KFAV_v1_3200	protein of unknown function	0	0	0	0
KFAV_v1_3207	KFAV_v1_3207	protein of unknown function	0	0	0	0
KFAV_v1_3210	KFAV_v1_3210	conserved protein of unknown function	0	0	0	0
KFAV_v1_3211	KFAV_v1_3211	protein of unknown function	0	0	0	0
KFAV_v1_3213	KFAV_v1_3213	protein of unknown function	0	0	0	0
KFAV_v1_3215	KFAV_v1_3215	conserved protein of unknown function	0	0	0	0
KFAV_v1_3216	KFAV_v1_3216	conserved protein of unknown function	0	0	0	0
KFAV_v1_3217	KFAV_v1_3217	conserved protein of unknown function	0	0	0	0
KFAV_v1_3219	KFAV_v1_3219	transposase (fragment)	0	0	0	0
KFAV_v1_3220	KFAV_v1_3220	conserved protein of unknown function	0	0	0	0
KFAV_v1_3223	KFAV_v1_3223	transposase (fragment)	0	0	0	0
KFAV_v1_3224	KFAV_v1_3224	transposase (fragment)	0	0	0	0
KFAV_v1_3228	KFAV_v1_3228	protein of unknown function	0	0	0	0
KFAV_v1_3231	KFAV_v1_3231	conserved protein of unknown function	0	0	0	0
KFAV_v1_3258	KFAV_v1_3258	protein of unknown function	0	0	0	0
KFAV_v1_3274	KFAV_v1_3274	protein of unknown function	0	0	0	0
KFAV_v1_3282	KFAV_v1_3282	protein of unknown function	0	0	0	0
KFAV_v1_3285	KFAV_v1_3285	putative enzyme	0	0	0	0
KFAV_v1_3286	KFAV_v1_3286	protein of unknown function	0	0	0	0
KFAV_v1_3288	KFAV_v1_3288	conserved protein of unknown function	0	0	0	0
KFAV_v1_3289	KFAV_v1_3289	protein of unknown function	0	0	0	0
KFAV_v1_3314	KFAV_v1_3314	Acyltransferase (fragment)	0	0	0	0
KFAV_v1_3315	KFAV_v1_3315	Beta-ureidopropionase (fragment)	0	0	0	0
KFAV_v1_3316	KFAV_v1_3316	D-hydantoinase	0	0	0	0

preA_1	KFAV_v1_3317	NAD-dependent dihydropyrimidine dehydrogenase subunit PreA	0	0	0	0
KFAV_v1_3319	KFAV_v1_3319	protein of unknown function	0	0	0	0
KFAV_v1_3320	KFAV_v1_3320	conserved protein of unknown function	0	0	0	0
KFAV_v1_3321	KFAV_v1_3321	ABC transporter permease	0	0	0	0
KFAV_v1_3324	KFAV_v1_3324	GNAT family acetyltransferase	0	0	0	0
KFAV_v1_3325	KFAV_v1_3325	Glycine/D-amino acid oxidase, deaminating	0	0	0	0
KFAV_v1_3332	KFAV_v1_3332	protein of unknown function	0	0	0	0
KFAV_v1_3333	KFAV_v1_3333	protein of unknown function	0	0	0	0
KFAV_v1_3343	KFAV_v1_3343	protein of unknown function	0	0	0	0
KFAV_v1_3345	KFAV_v1_3345	protein of unknown function	0	0	0	0
KFAV_v1_3346	KFAV_v1_3346	protein of unknown function	0	0	0	0
KFAV_v1_3347	KFAV_v1_3347	Uncharacterized aminotransferase AF_1815 (fragment)	0	0	0	0
KFAV_v1_3349	KFAV_v1_3349	conserved protein of unknown function	0	0	0	0
KFAV_v1_3350	KFAV_v1_3350	conserved protein of unknown function	0	0	0	0
KFAV_v1_3351	KFAV_v1_3351	protein of unknown function	0	0	0	0
tpx	KFAV_v1_3362	Thiol peroxidase	0	0	0	0
KFAV_v1_3365	KFAV_v1_3365	conserved protein of unknown function	0	0	0	0
KFAV_v1_3367	KFAV_v1_3367	carbon-phosphorus lyase complex subunit (fragment)	0	0	0	0
yfkQ_3	KFAV_v1_3370	Uncharacterized membrane protein YfkQ	0	0	0	0
KFAV_v1_3371	KFAV_v1_3371	conserved protein of unknown function	0	0	0	0
yhbJ_2	KFAV_v1_3373	putative membrane fusion protein; putative exporter subunit (benzoate transcriptome)	0	0	0	0
KFAV_v1_3374	KFAV_v1_3374	conserved protein of unknown function	0	0	0	0
KFAV_v1_3375	KFAV_v1_3375	conserved exported protein of unknown function	0	0	0	0
KFAV_v1_3405	KFAV_v1_3405	protein of unknown function	0	0	0	0
KFAV_v1_3422	KFAV_v1_3422	protein of unknown function	0	0	0	0
KFAV_v1_3424	KFAV_v1_3424	protein of unknown function	0	0	0	0
KFAV_v1_3432	KFAV_v1_3432	exported protein of unknown function	0	0	0	0
KFAV_v1_3433	KFAV_v1_3433	Glycosyl transferase group 1 (fragment)	0	0	0	0
KFAV_v1_3435	KFAV_v1_3435	conserved protein of unknown function	0	0	0	0
KFAV_v1_3438	KFAV_v1_3438	Transcriptional regulator/antitoxin, MazE	0	0	0	0
KFAV_v1_3441	KFAV_v1_3441	protein of unknown function	0	0	0	0
KFAV_v1_3443	KFAV_v1_3443	Transcriptional regulator, AbrB family	0	0	0	0
KFAV_v1_3448	KFAV_v1_3448	protein of unknown function	0	0	0	0
KFAV_v1_3450	KFAV_v1_3450	Antitoxin (fragment)	0	0	0	0
KFAV_v1_3451	KFAV_v1_3451	Ribonuclease VapC (fragment)	0	0	0	0
KFAV_v1_3455	KFAV_v1_3455	Helix-turn-helix domain protein (fragment)	0	0	0	0
KFAV_v1_3461	KFAV_v1_3461	protein of unknown function	0	0	0	0
KFAV_v1_3465	KFAV_v1_3465	protein of unknown function	0	0	0	0
KFAV_v1_3467	KFAV_v1_3467	protein of unknown function	0	0	0	0
KFAV_v1_3468	KFAV_v1_3468	protein of unknown function	0	0	0	0
KFAV_v1_3469	KFAV_v1_3469	protein of unknown function	0	0	0	0
KFAV_v1_3472	KFAV_v1_3472	conserved protein of unknown function	0	0	0	0
KFAV_v1_3475	KFAV_v1_3475	conserved protein of unknown function	0	0	0	0
spoIIID	KFAV_v1_3481	transcriptional regulator (stage III sporulation)	0	0	0	0
spoIID	KFAV_v1_3483	Stage II sporulation protein D	0	0	0	0
KFAV_v1_3489	KFAV_v1_3489	conserved protein of unknown function	0	0	0	0
KFAV_v1_3521	KFAV_v1_3521	protein of unknown function	0	0	0	0
KFAV_v1_3522	KFAV_v1_3522	transposase	0	0	0	0
KFAV_v1_3527	KFAV_v1_3527	protein of unknown function	0	0	0	0
KFAV_v1_3530	KFAV_v1_3530	protein of unknown function	0	0	0	0
KFAV_v1_3550	KFAV_v1_3550	protein of unknown function	0	0	0	0
KFAV_v1_3564	KFAV_v1_3564	Penicillin-binding protein	0	0	0	0

KFAV_v1_3566	KFAV_v1_3566	conserved protein of unknown function	0	0	0	0
KFAV_v1_3567	KFAV_v1_3567	protein of unknown function	0	0	0	0
KFAV_v1_3569	KFAV_v1_3569	conserved protein of unknown function	0	0	0	0
KFAV_v1_3570	KFAV_v1_3570	protein of unknown function	0	0	0	0
KFAV_v1_3571	KFAV_v1_3571	protein of unknown function	0	0	0	0
KFAV_v1_3572	KFAV_v1_3572	conserved protein of unknown function	0	0	0	0
KFAV_v1_3574	KFAV_v1_3574	Binding-protein-dependent transport systems inner membrane component	0	0	0	0
KFAV_v1_3577	KFAV_v1_3577	CxxH/CxxC protein, BA_5709 family (modular protein)	0	0	0	0
KFAV_v1_3581	KFAV_v1_3581	protein of unknown function	0	0	0	0
KFAV_v1_3594	KFAV_v1_3594	conserved protein of unknown function	0	0	0	0
